# Supplementary material for: Mapping AMPA and NMDA receptors at mouse brain hippocampal synapses by cryo-ET
Source: bioRxiv. 2026 Nov 3:2026.11.02.763697. Preprint. [Version 1] doi: 10.64898/2026.11.02.763697 (PMC13734303; doi:10.64898/2026.11.02.763697)

Tomogram ID: 20231017_EGmilled24-2_68	SET: anti-GluA2 15F1 "C-term"
Cleft ID: 0	Type: tissue

Denoised Tomogram Slice

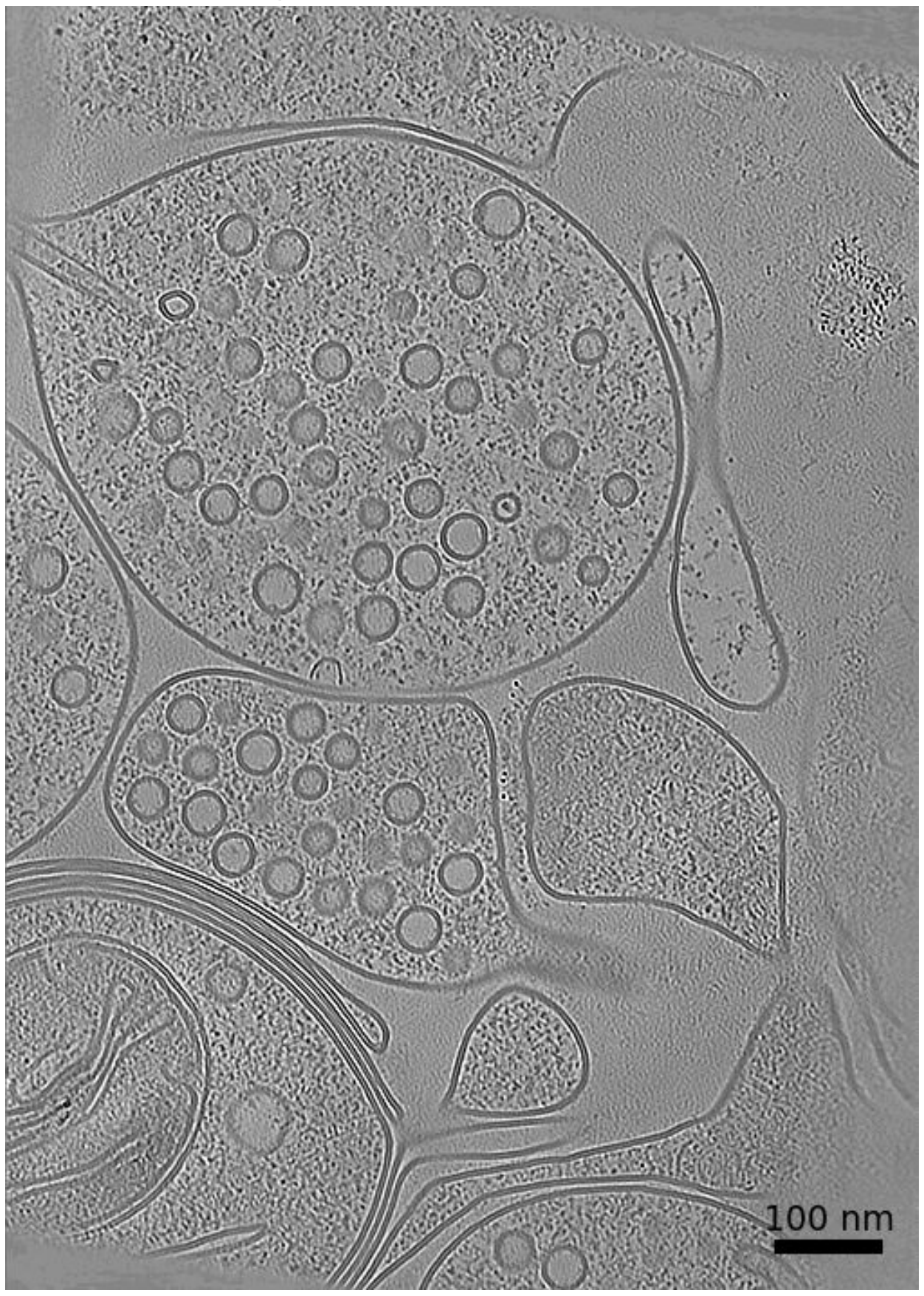

Membrane Segmentation & Cleft Position

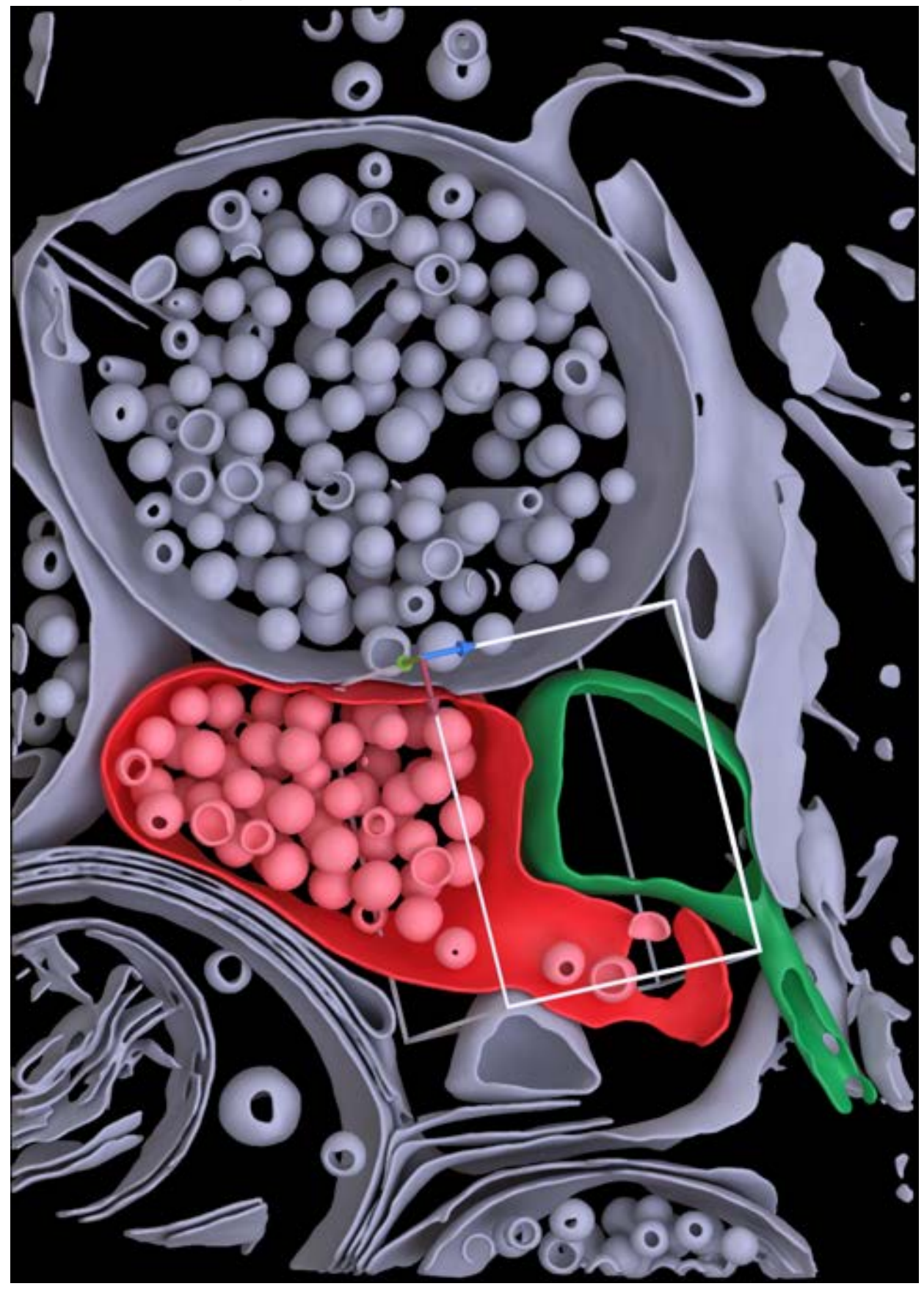

Cleft MIP

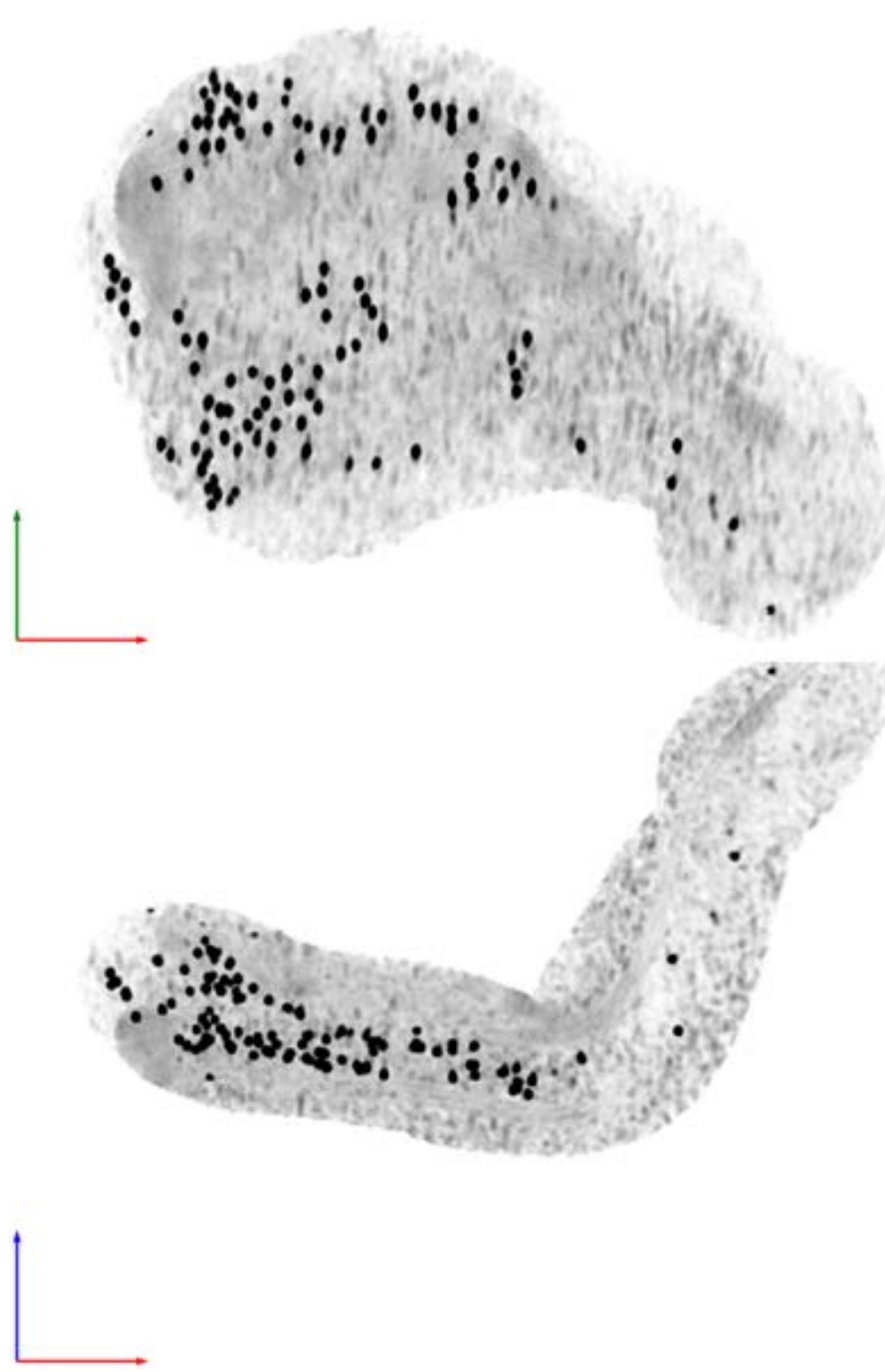

Tomogram ID: 20231017_HippAu_159 **SET: anti-GluA2 15F1 "C-term"**

Cleft ID: 0 Type: tissue

Denoised Tomogram Slice

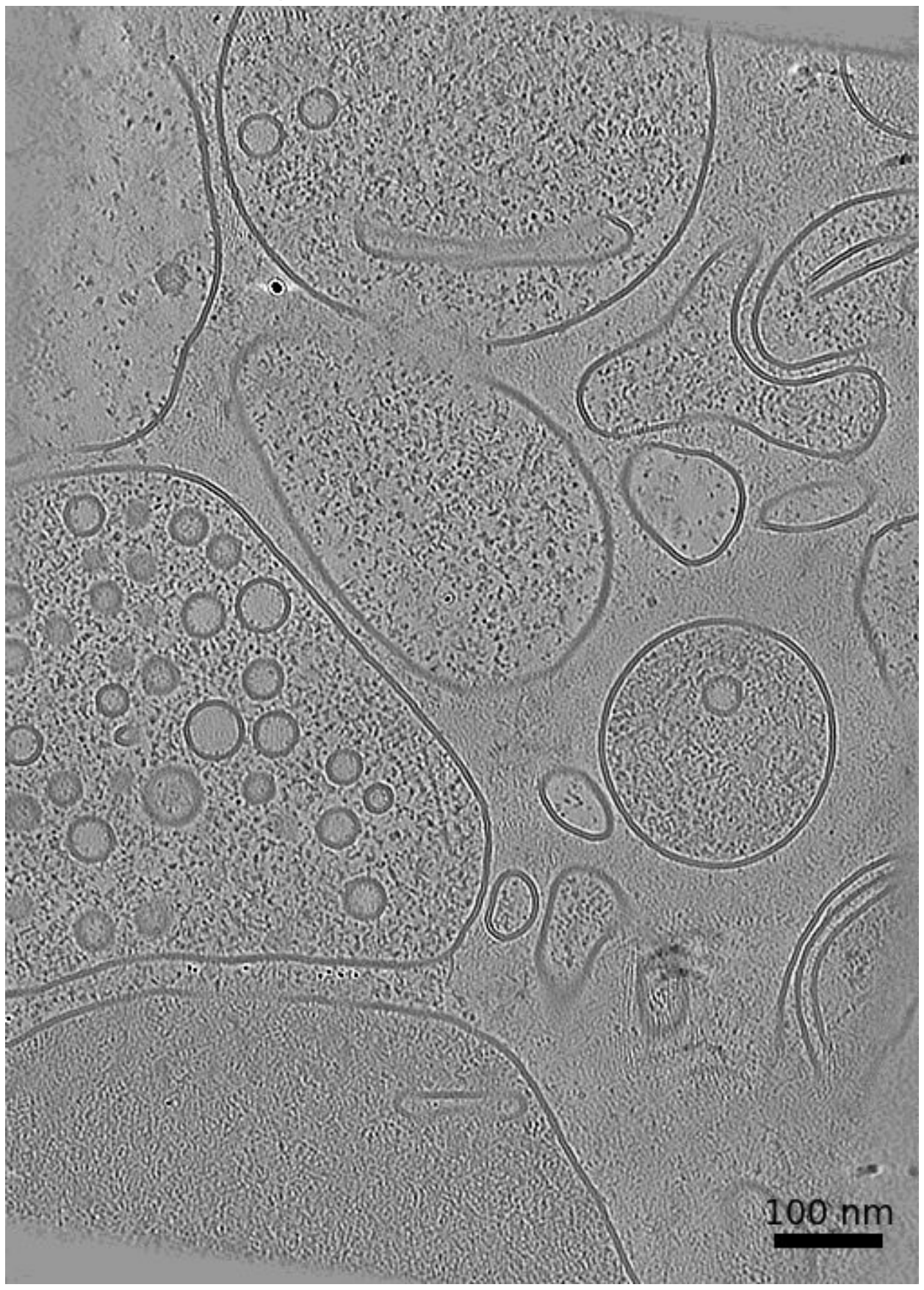

Membrane Segmentation & Cleft Position

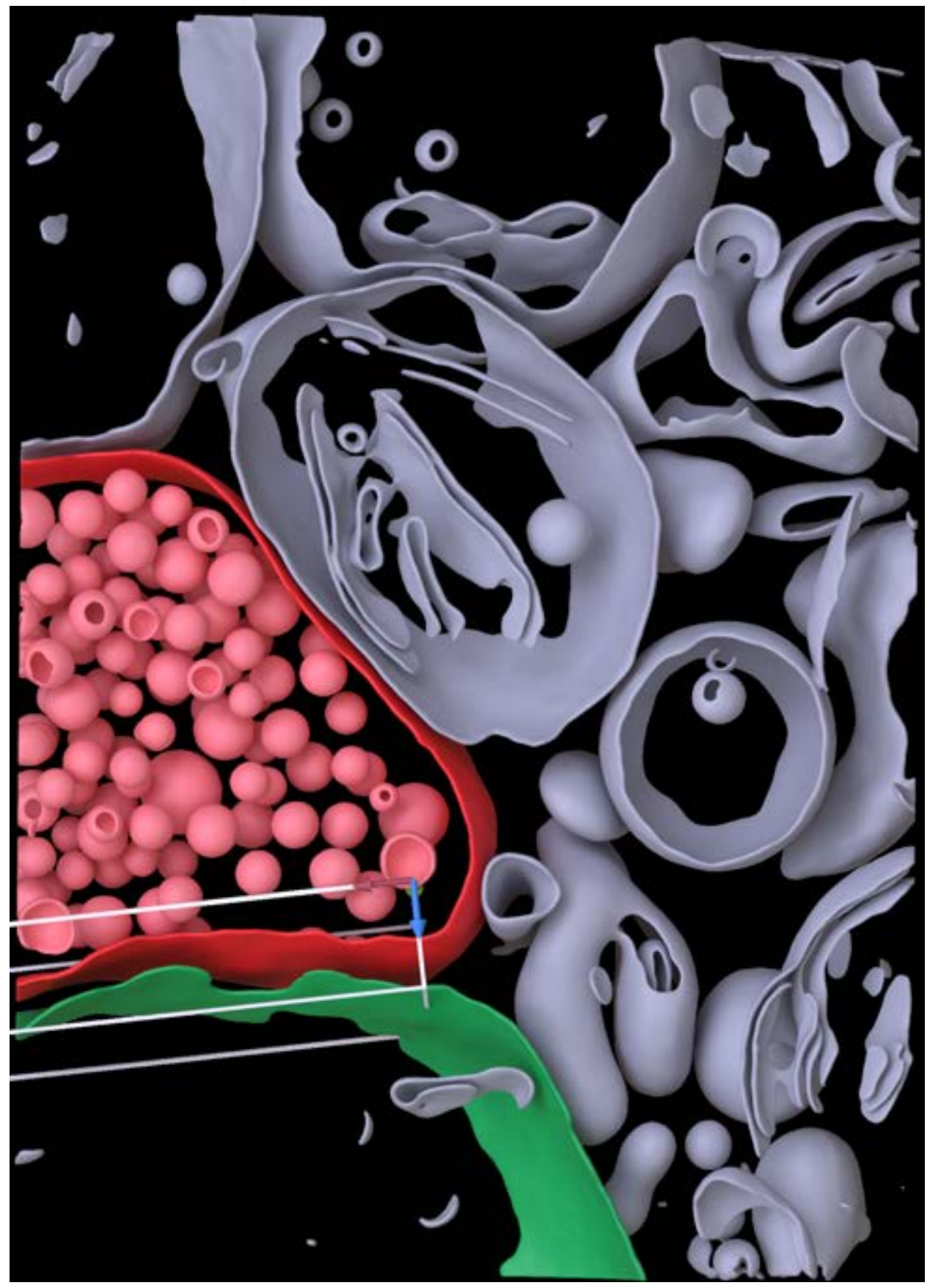

Cleft MIP

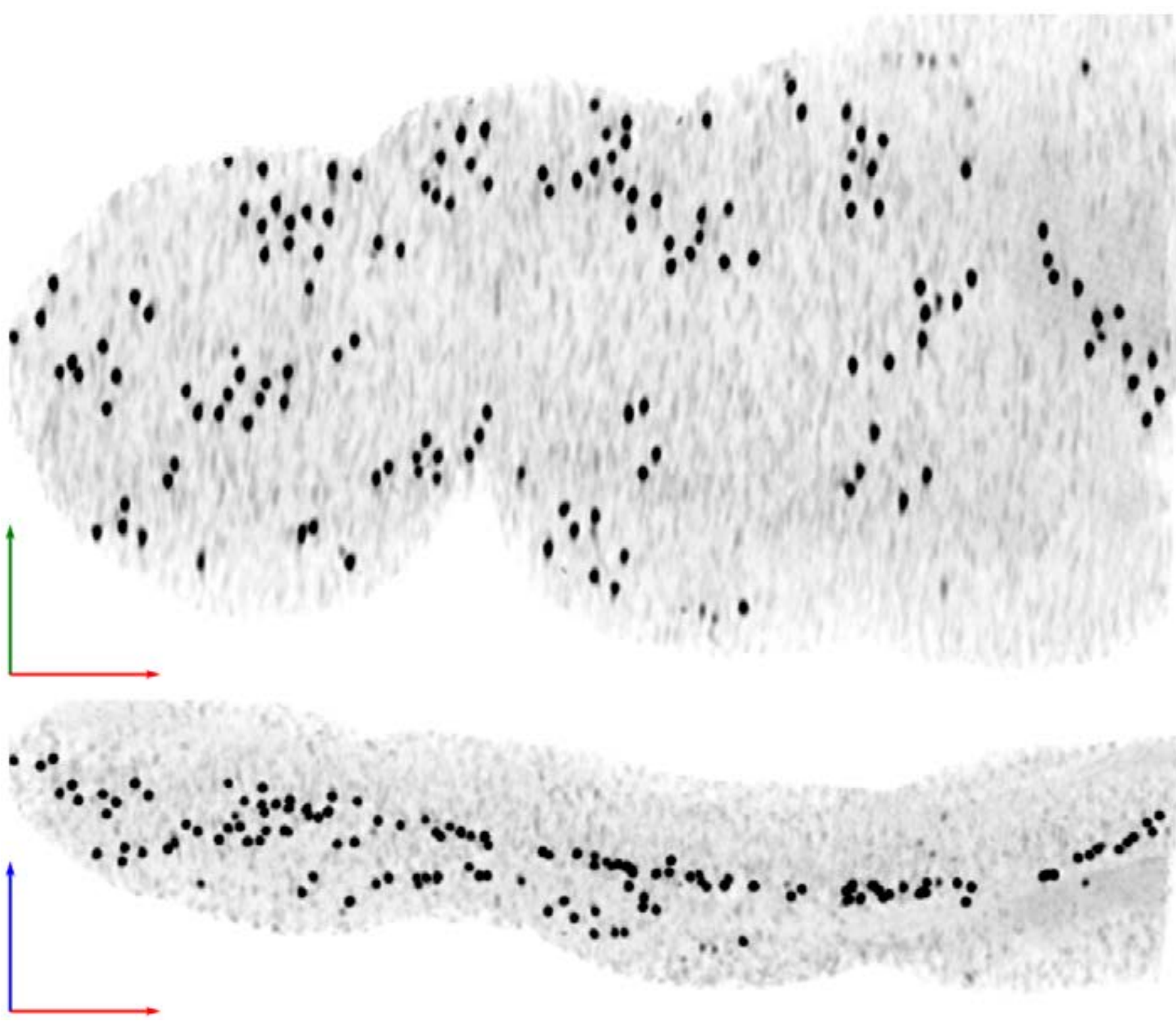

Tomogram ID: 20231026_HippAu_26 | **SET: anti-GluA2 15F1 "C-term"**

Cleft ID: 0 | Type: tissue

Denoised Tomogram Slice

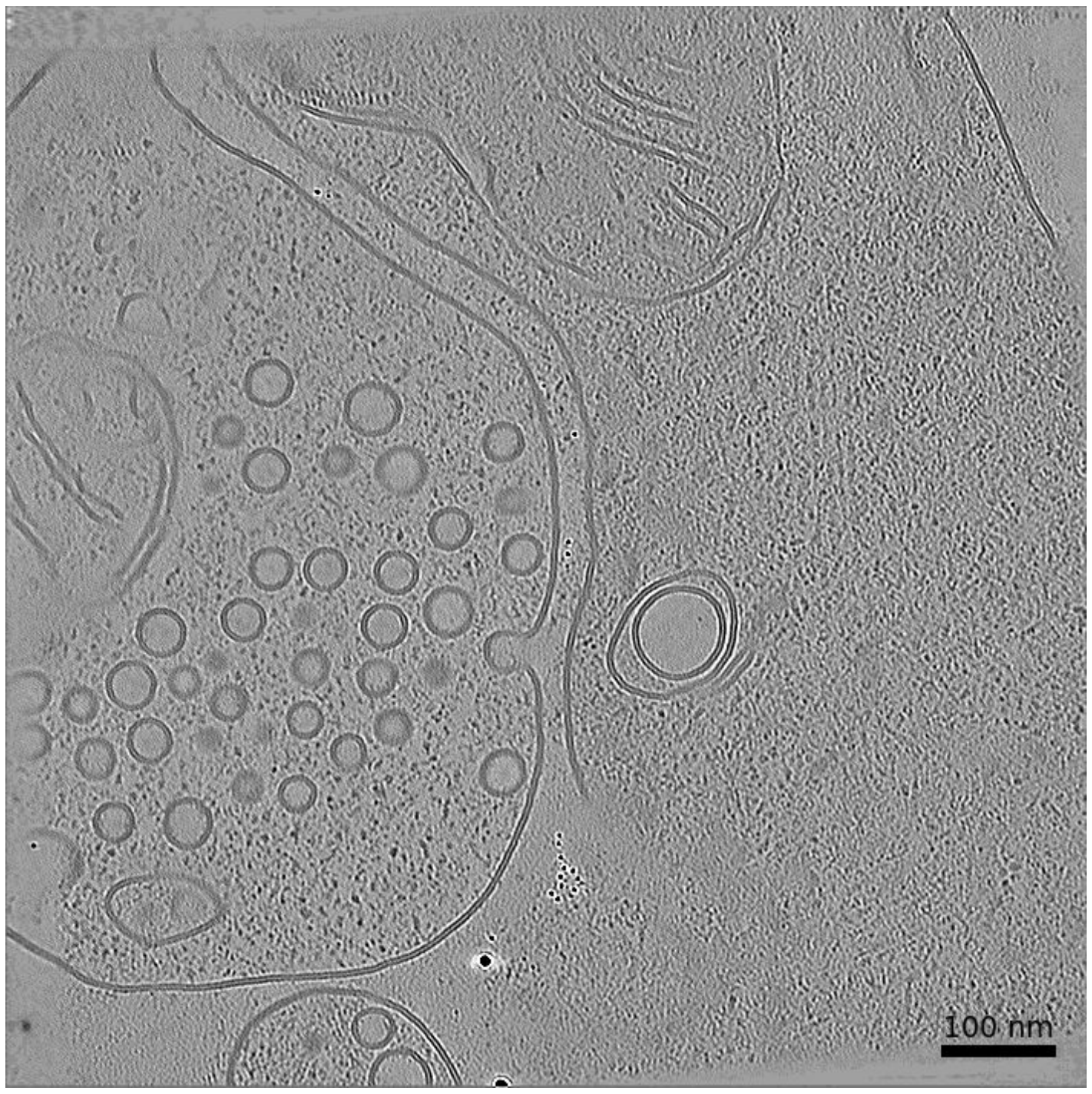

Membrane Segmentation & Cleft Position

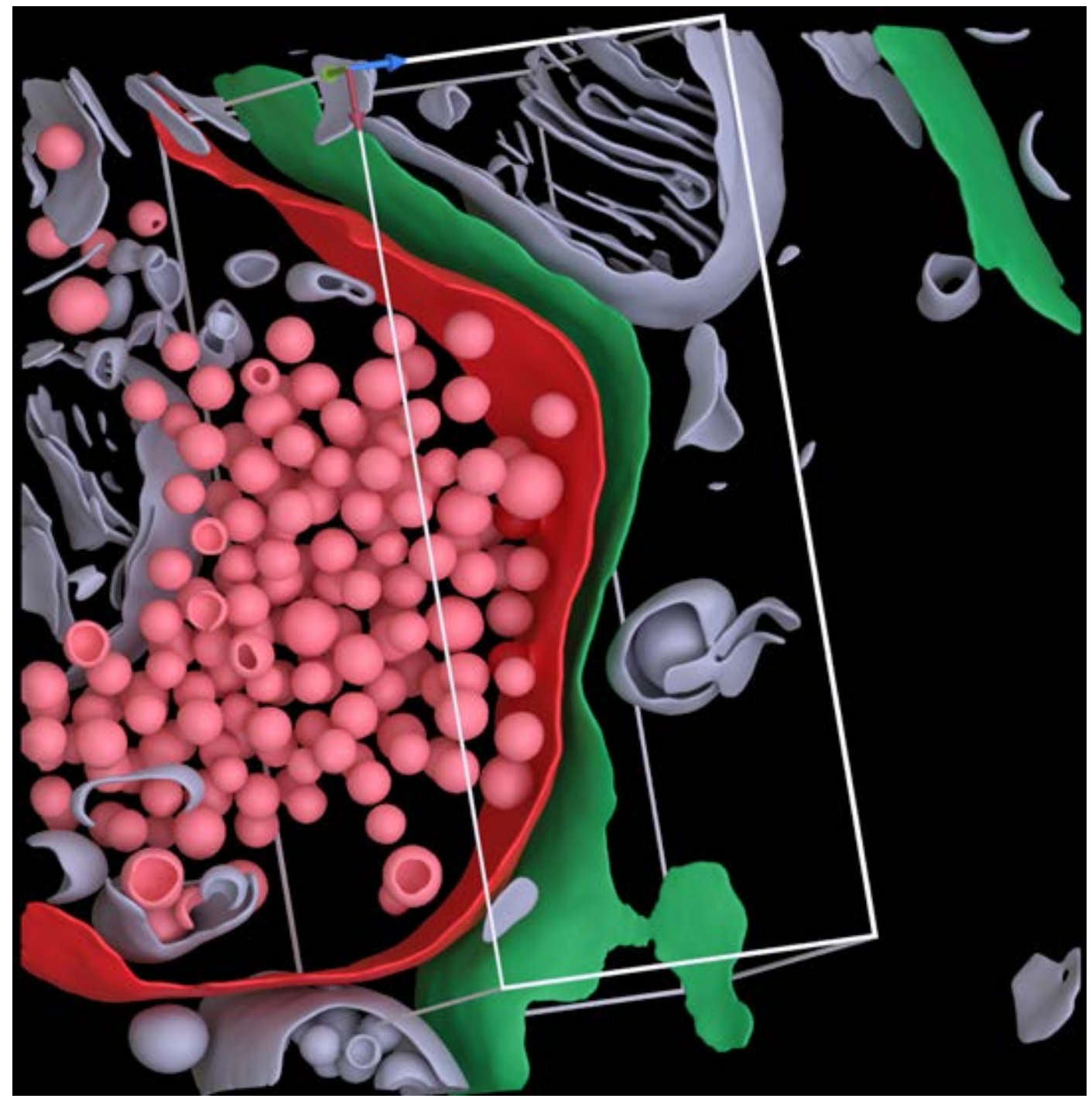

Cleft MIP

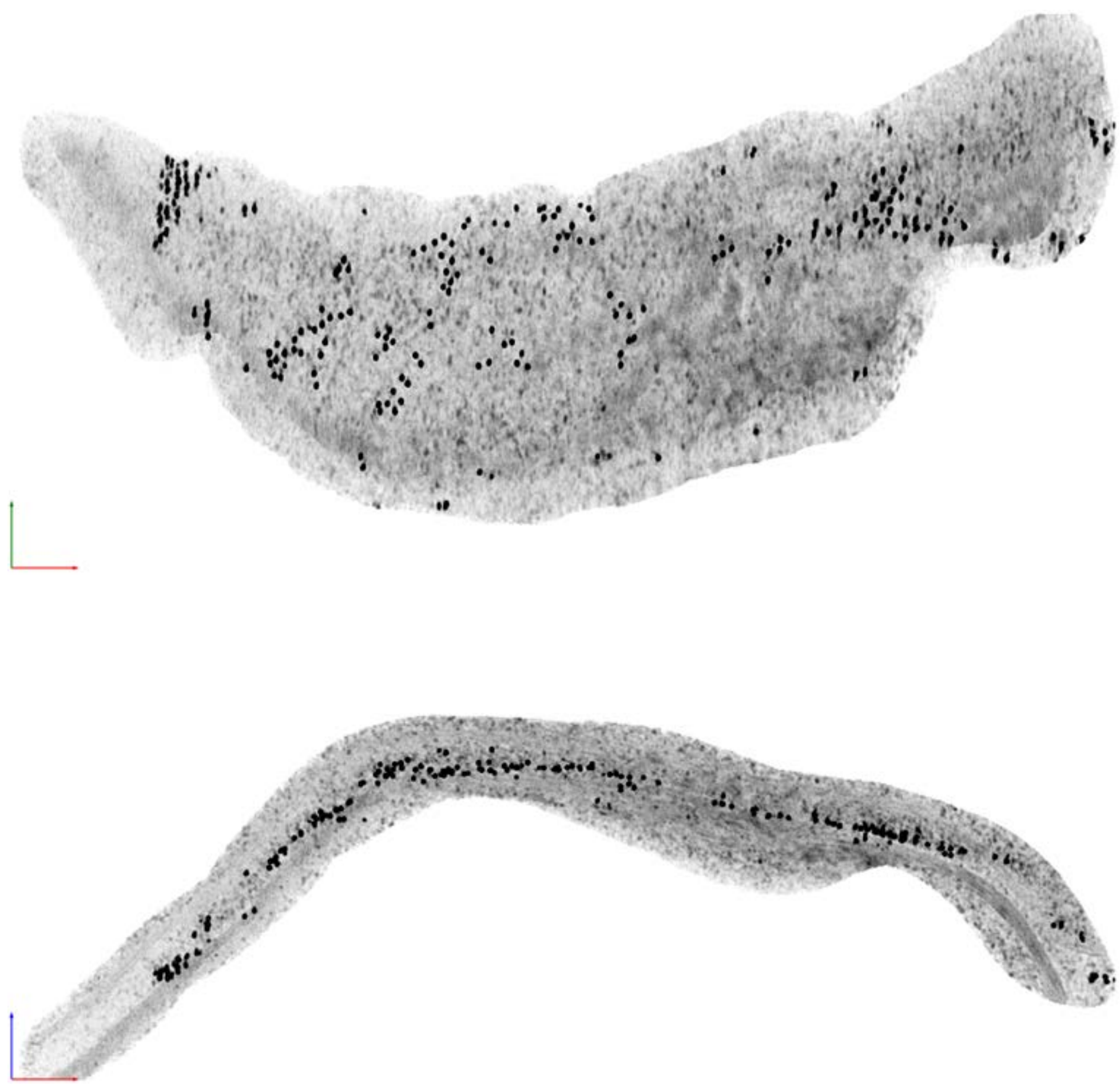

Tomogram ID: 20240111_WaffleHipp_116 | **SET: anti-GluA2 15F1 "C-term"**

Cleft ID: 0 | Type: tissue

Denoised Tomogram Slice

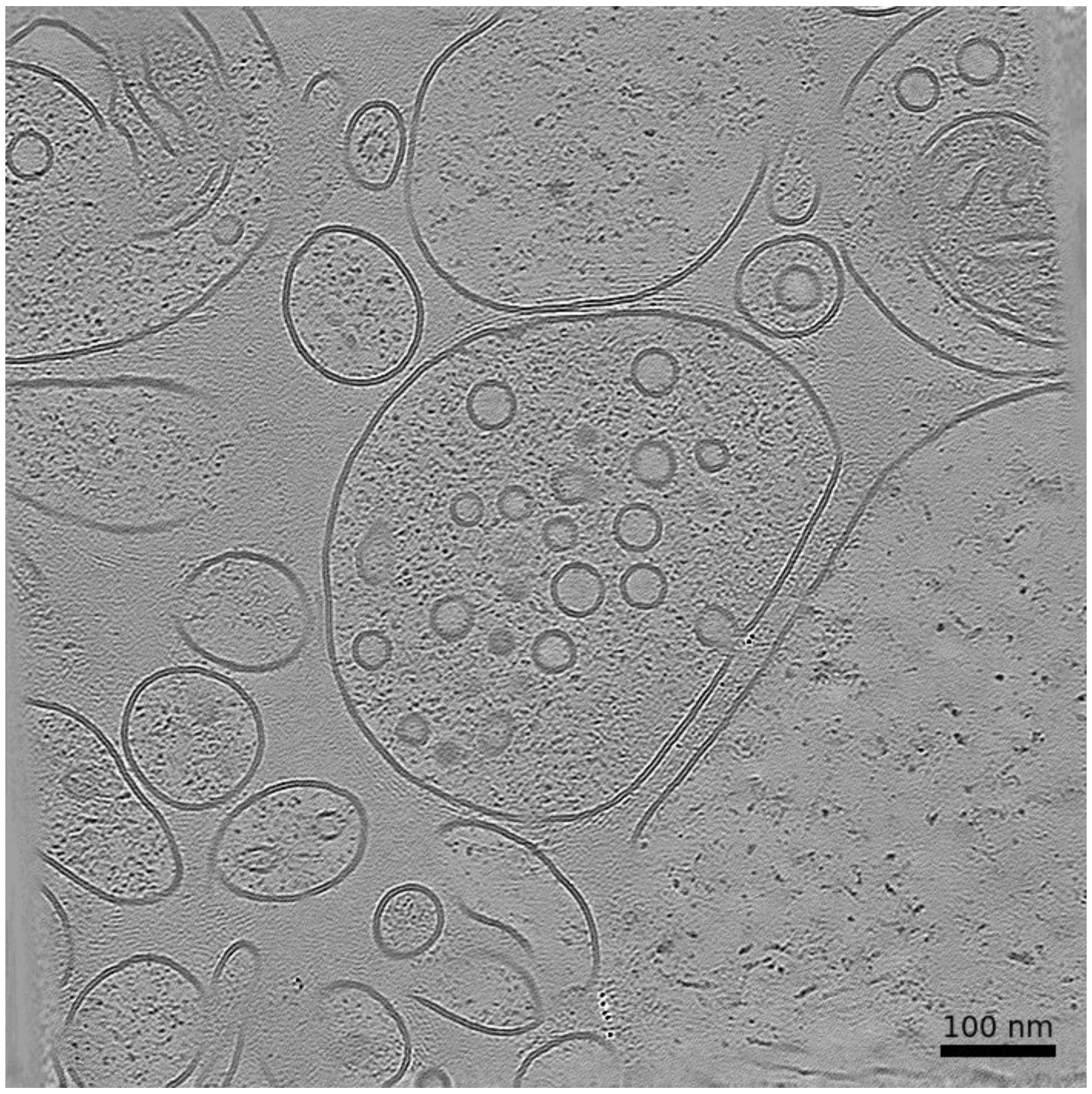

Membrane Segmentation & Cleft Position

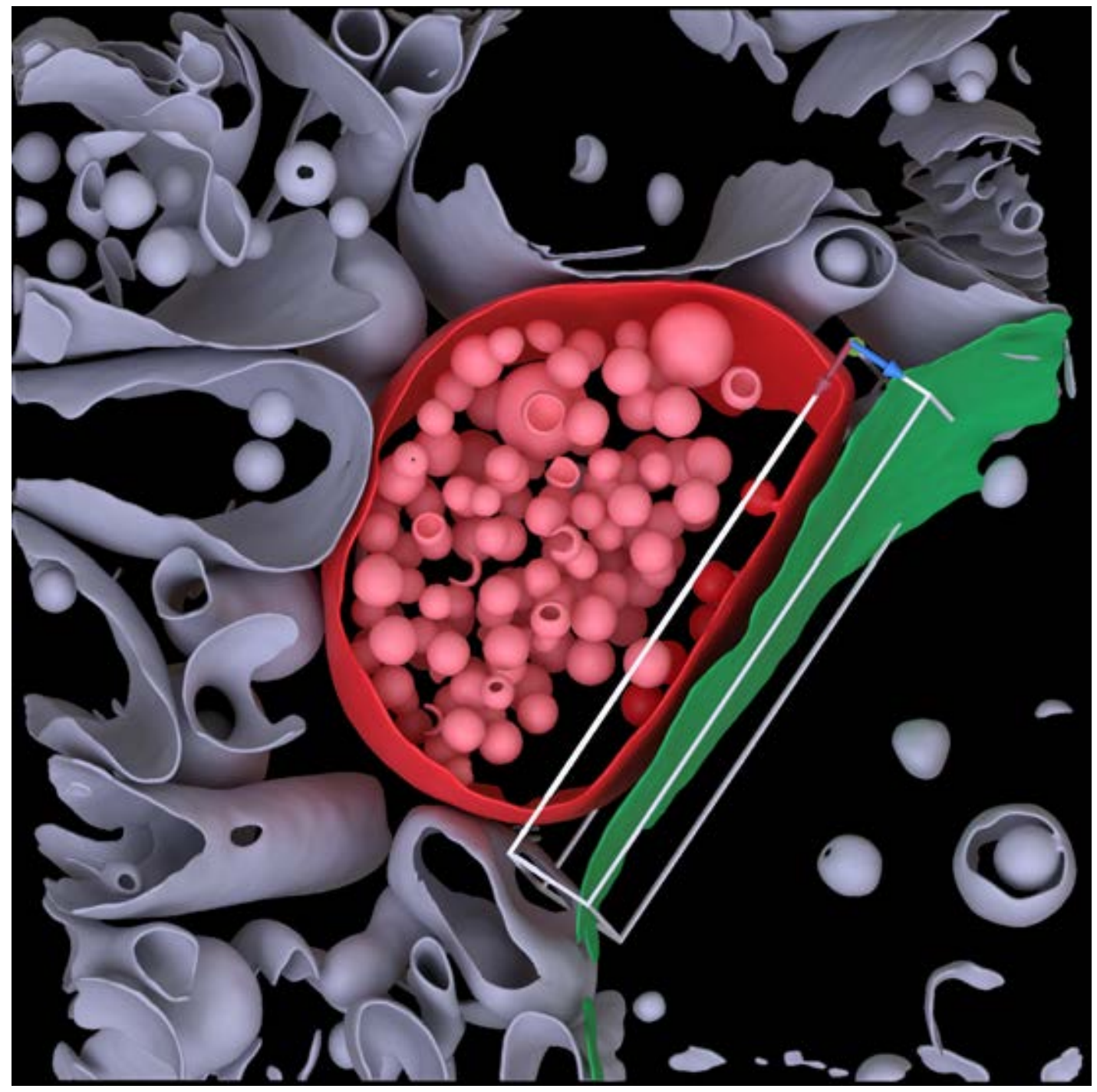

Cleft MIP

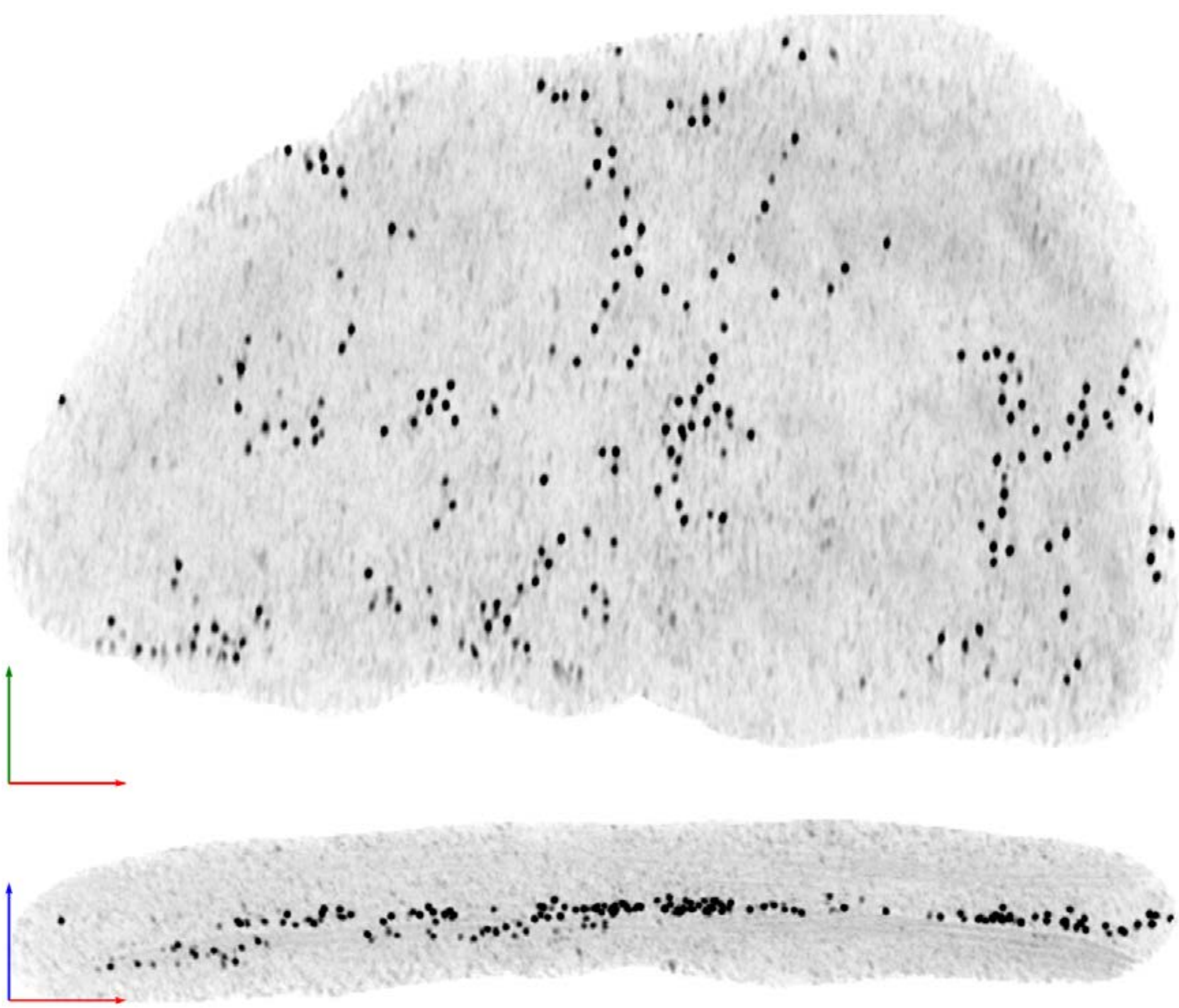

Tomogram ID: 20240111_WaffleHipp_183 | **SET: anti-GluA2 15F1 "C-term"**

Cleft ID: 0 | Type: tissue

Denoised Tomogram Slice

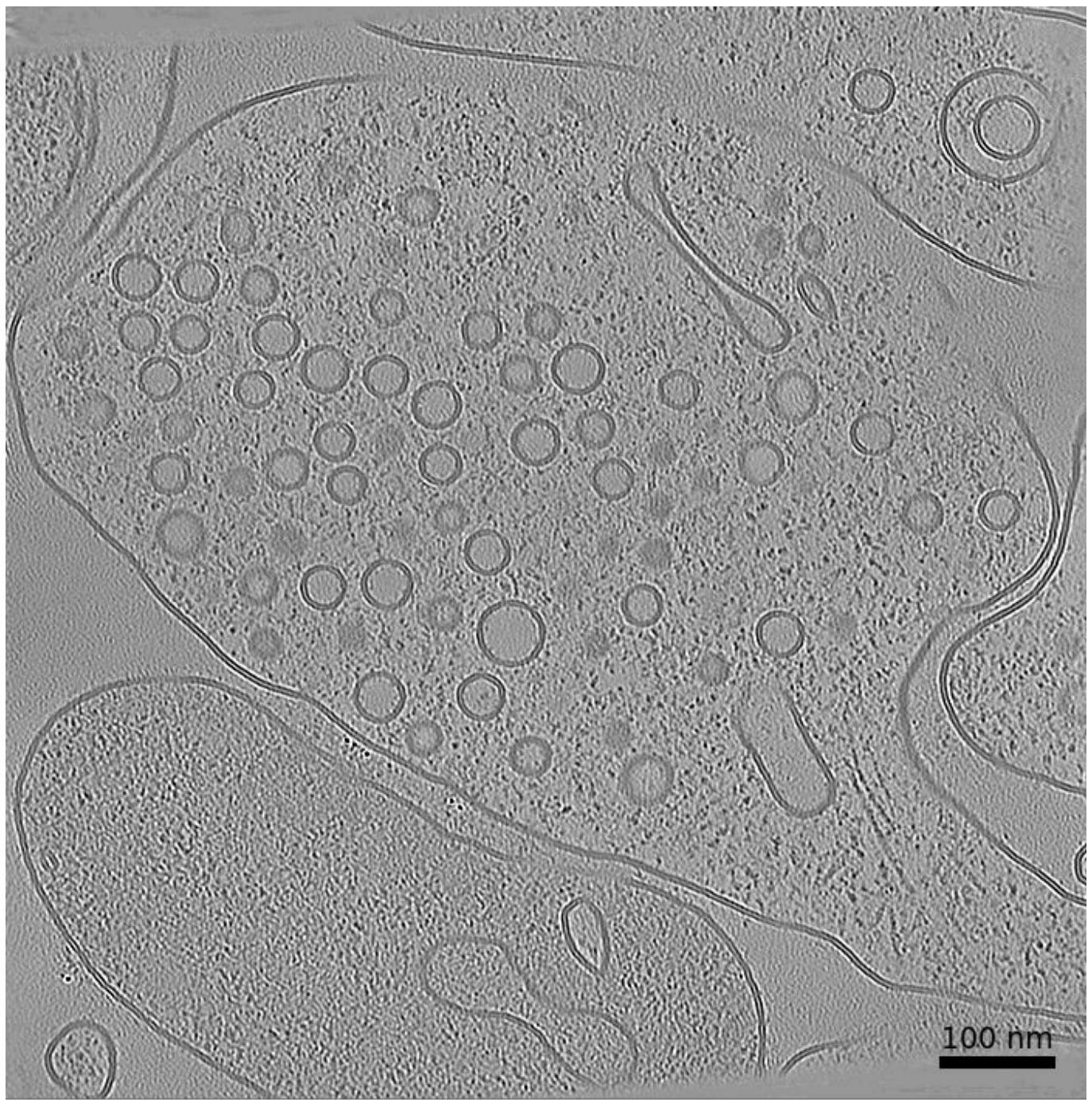

Membrane Segmentation & Cleft Position

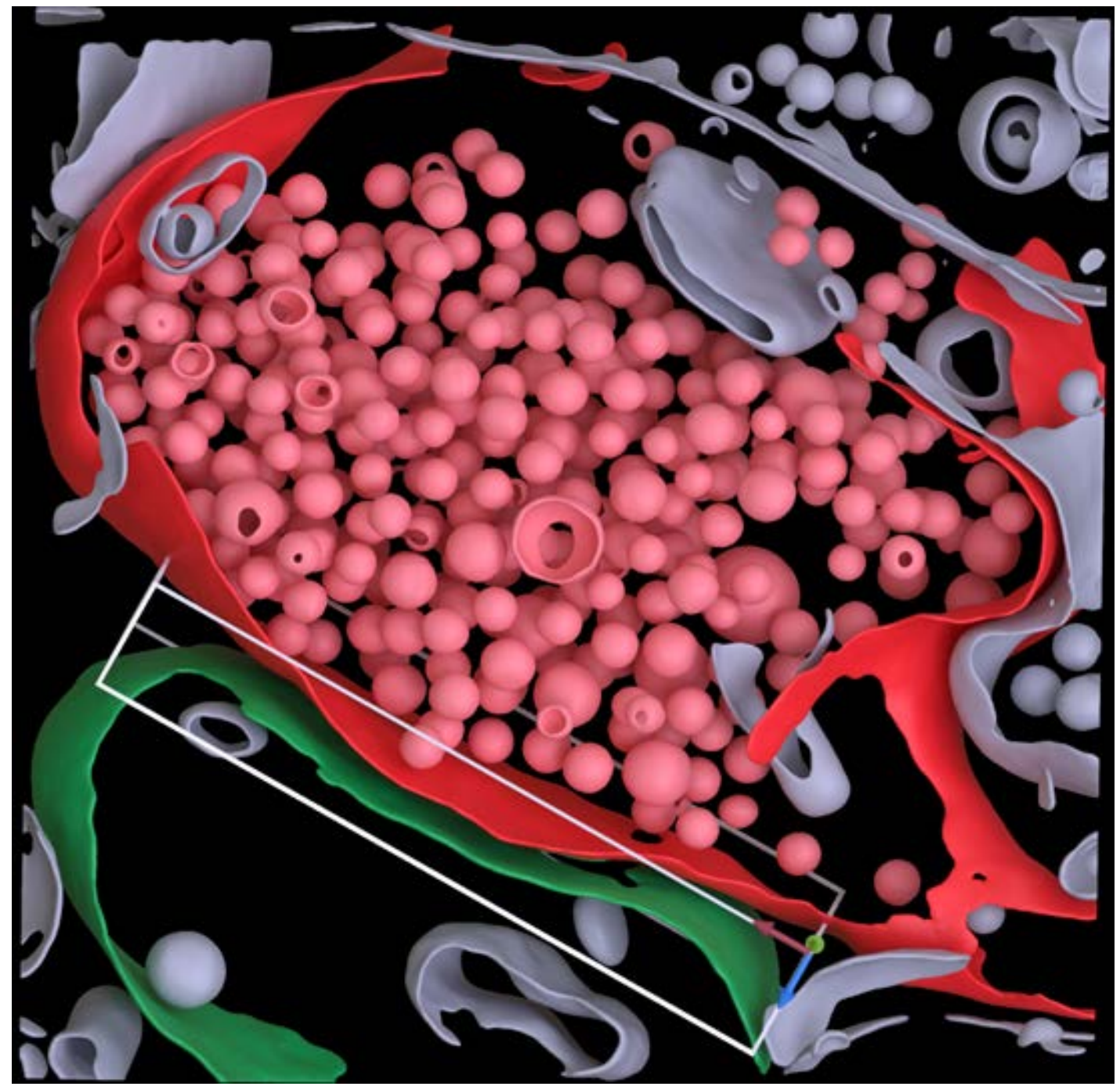

Cleft MIP

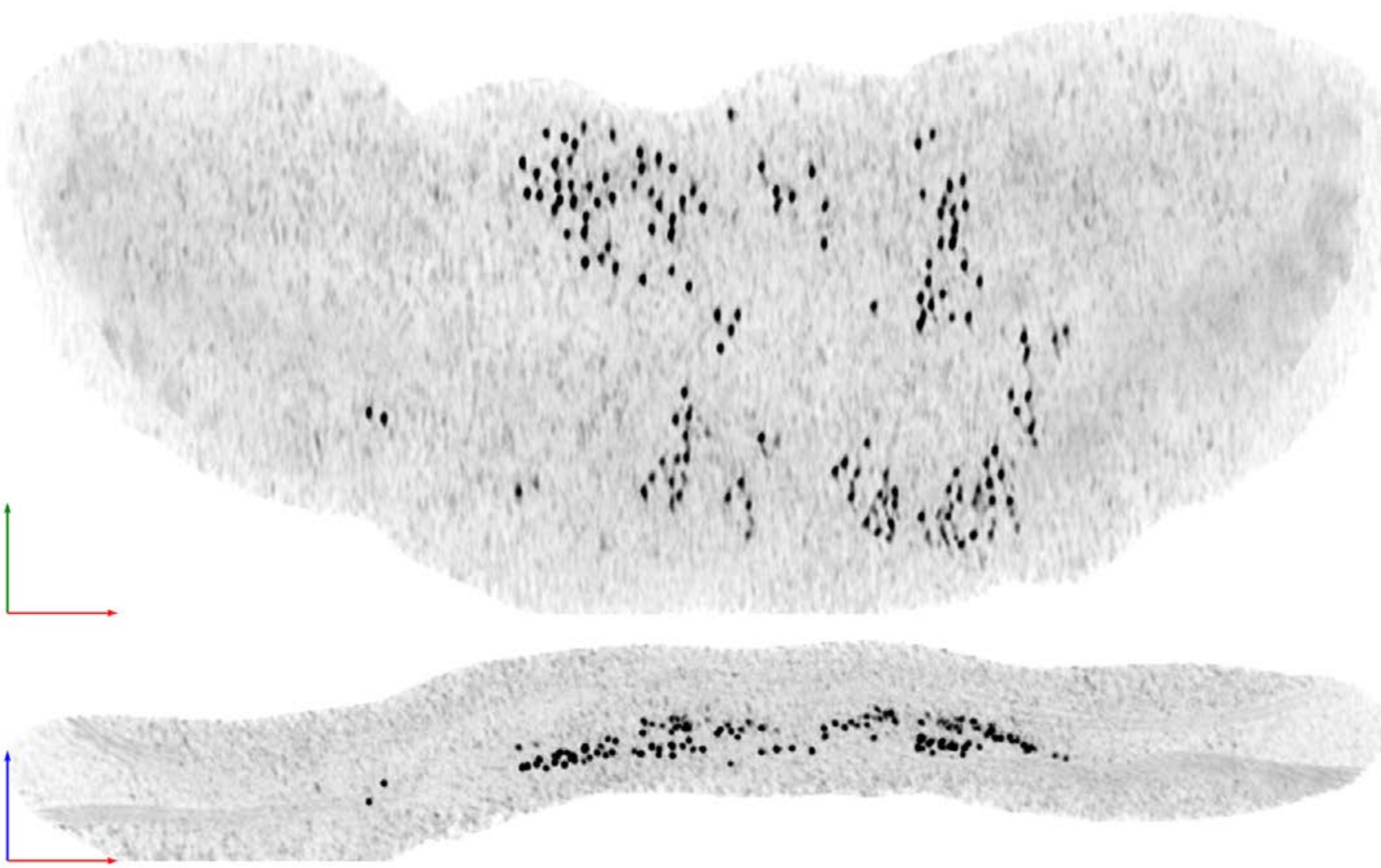

Tomogram ID: 20240111_WaffleHipp_195	SET: anti-GluA2 15F1 "C-term"
Cleft ID: 0	Type: synaptosome

Denoised Tomogram Slice

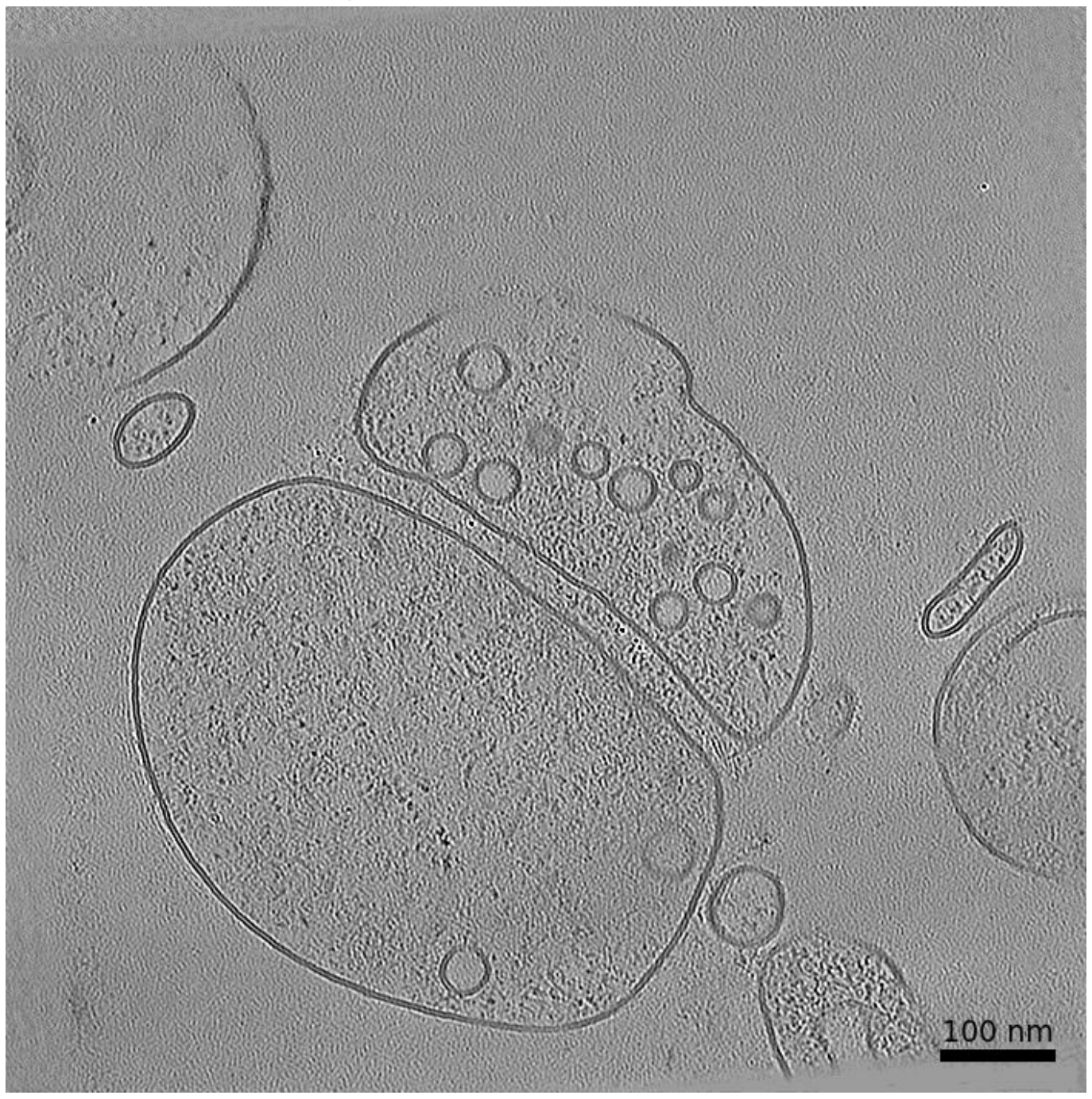

Membrane Segmentation & Cleft Position

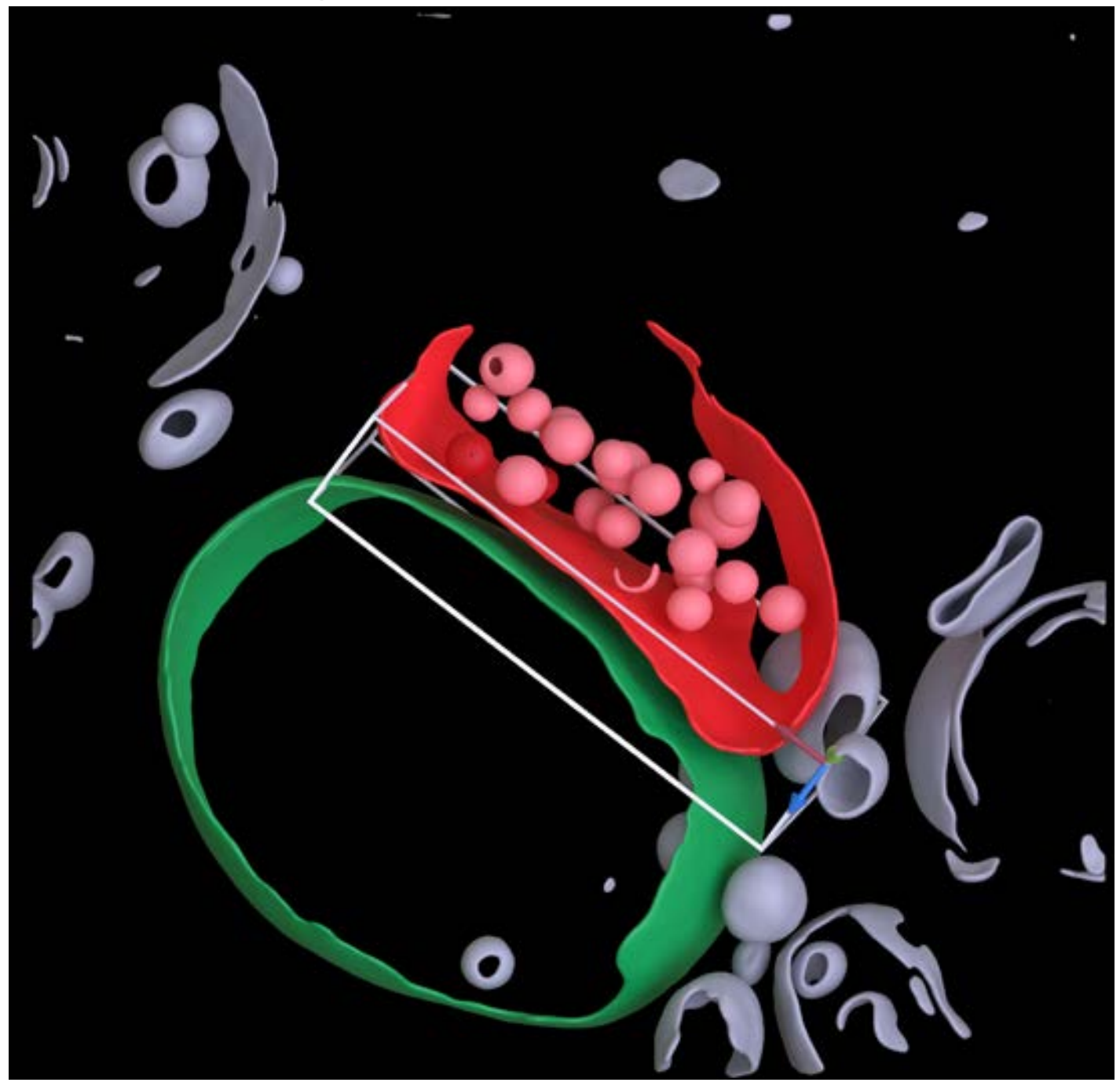

Cleft MIP

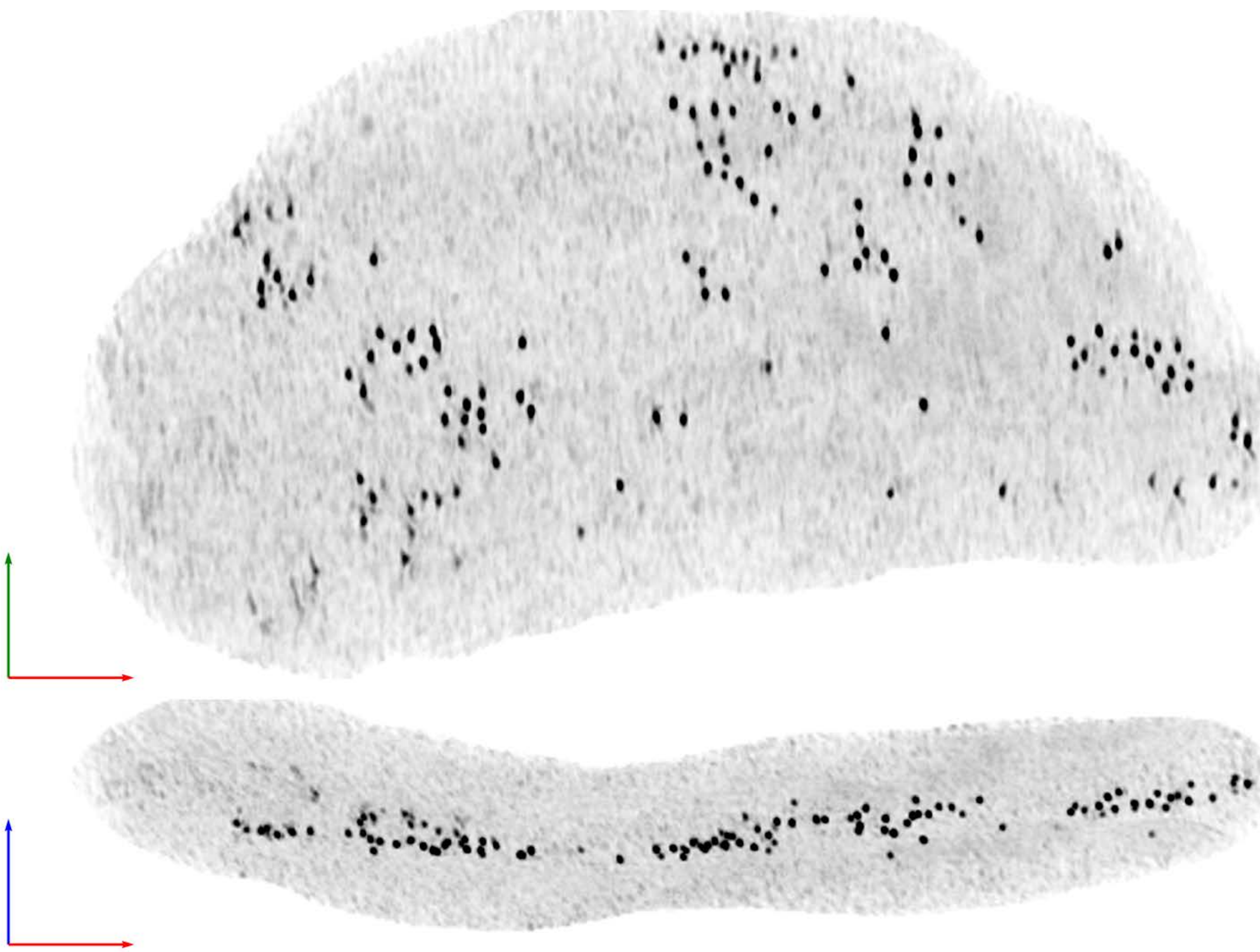

Tomogram ID: 20240111_WaffleHipp_198 **SET: anti-GluA2 15F1 "C-term"**

Cleft ID: 0 Type: tissue

Denoised Tomogram Slice

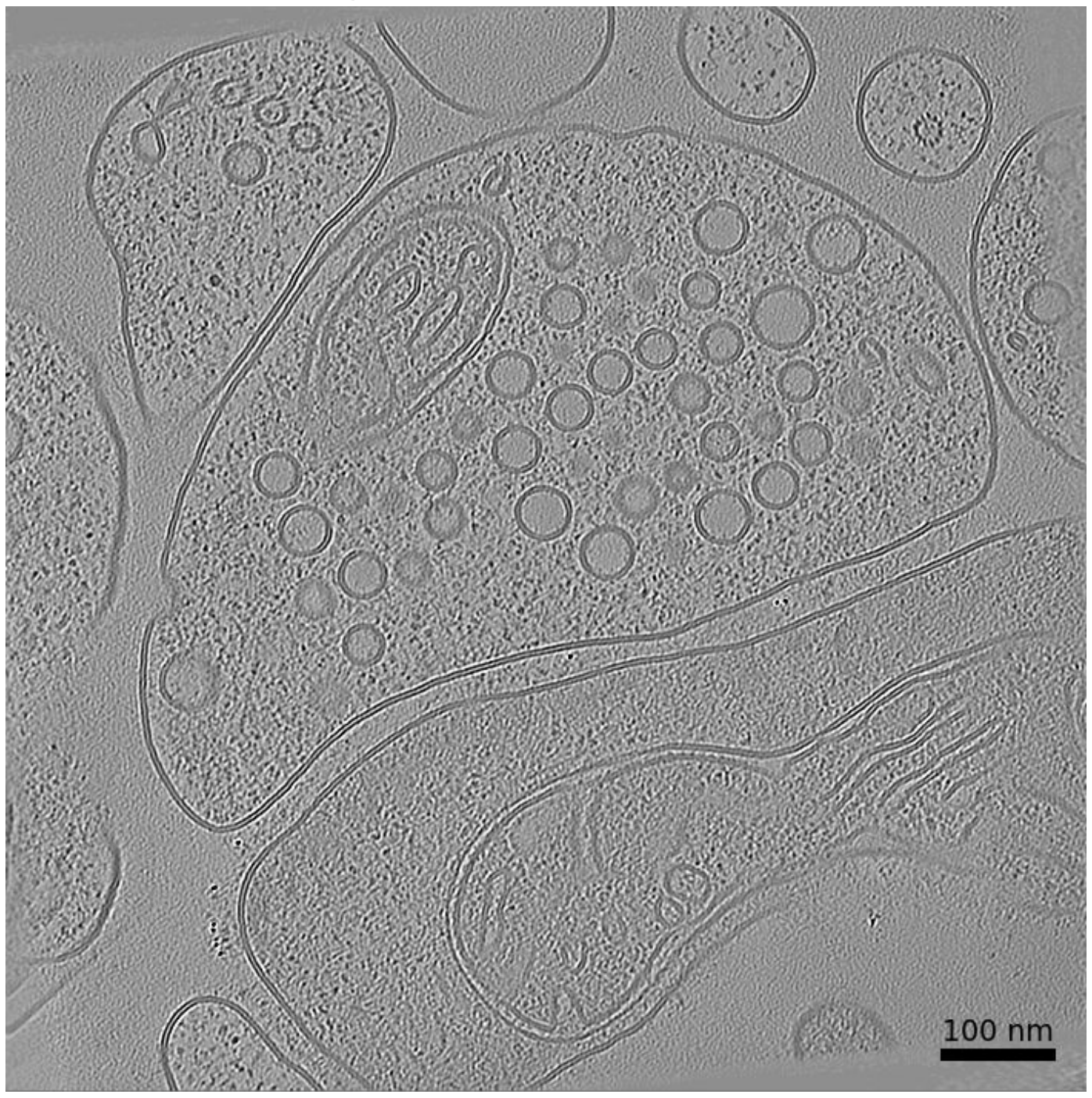

Membrane Segmentation & Cleft Position

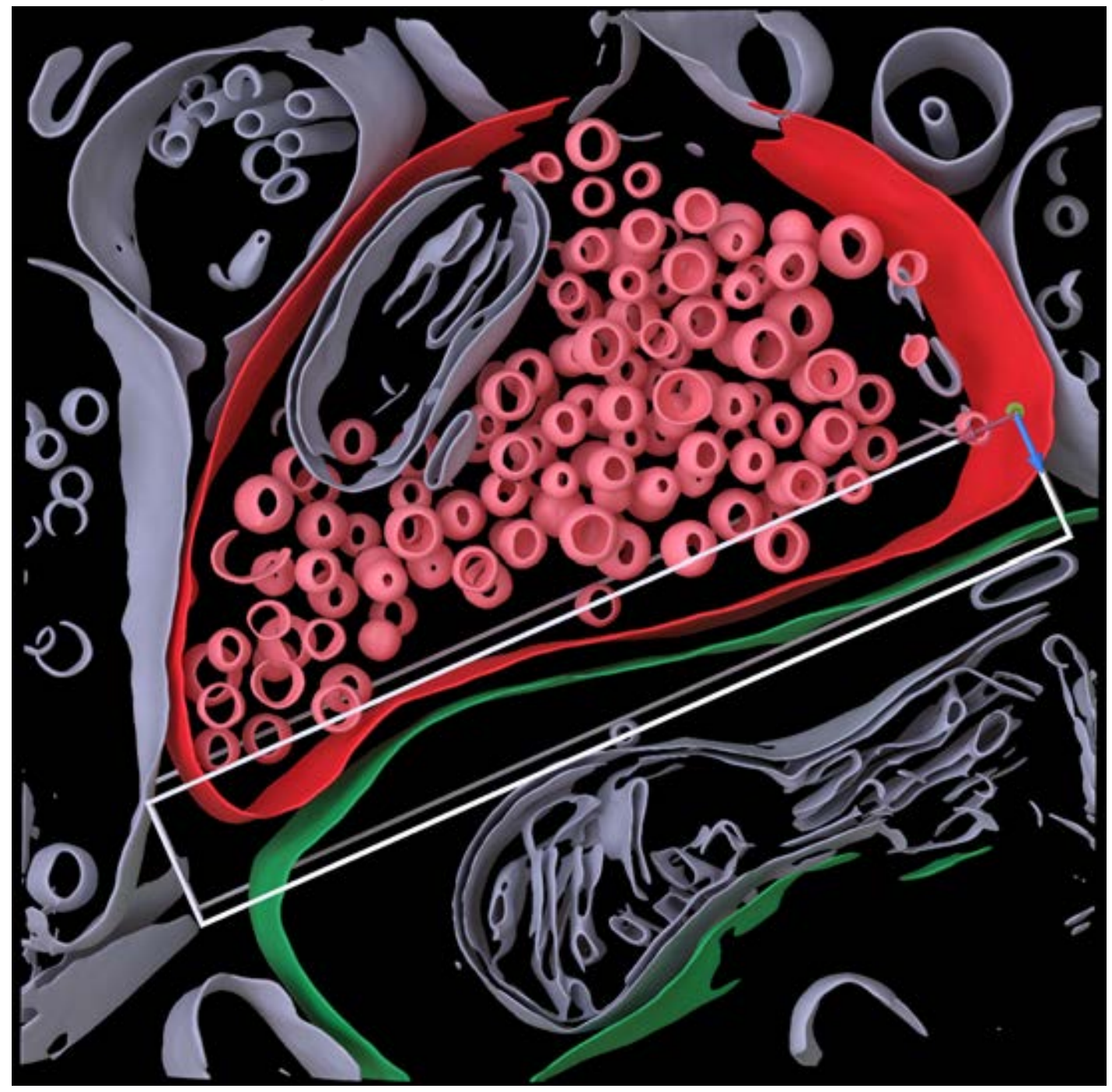

Cleft MIP

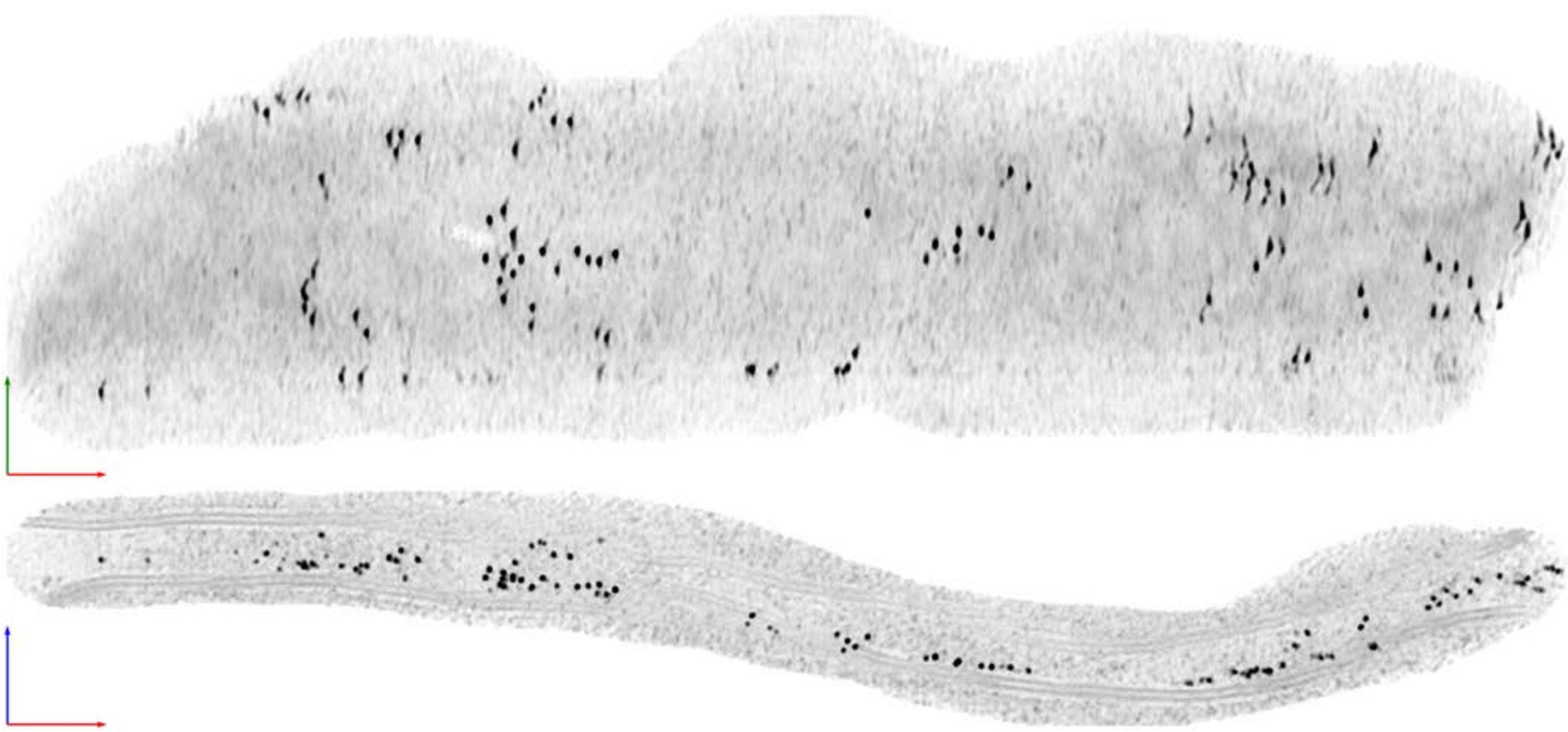

Tomogram ID: 20240111_WaffleHipp_227	SET: anti-GluA2 15F1 "C-term"
Cleft ID: 0	Type: semi-tissue

Denoised Tomogram Slice

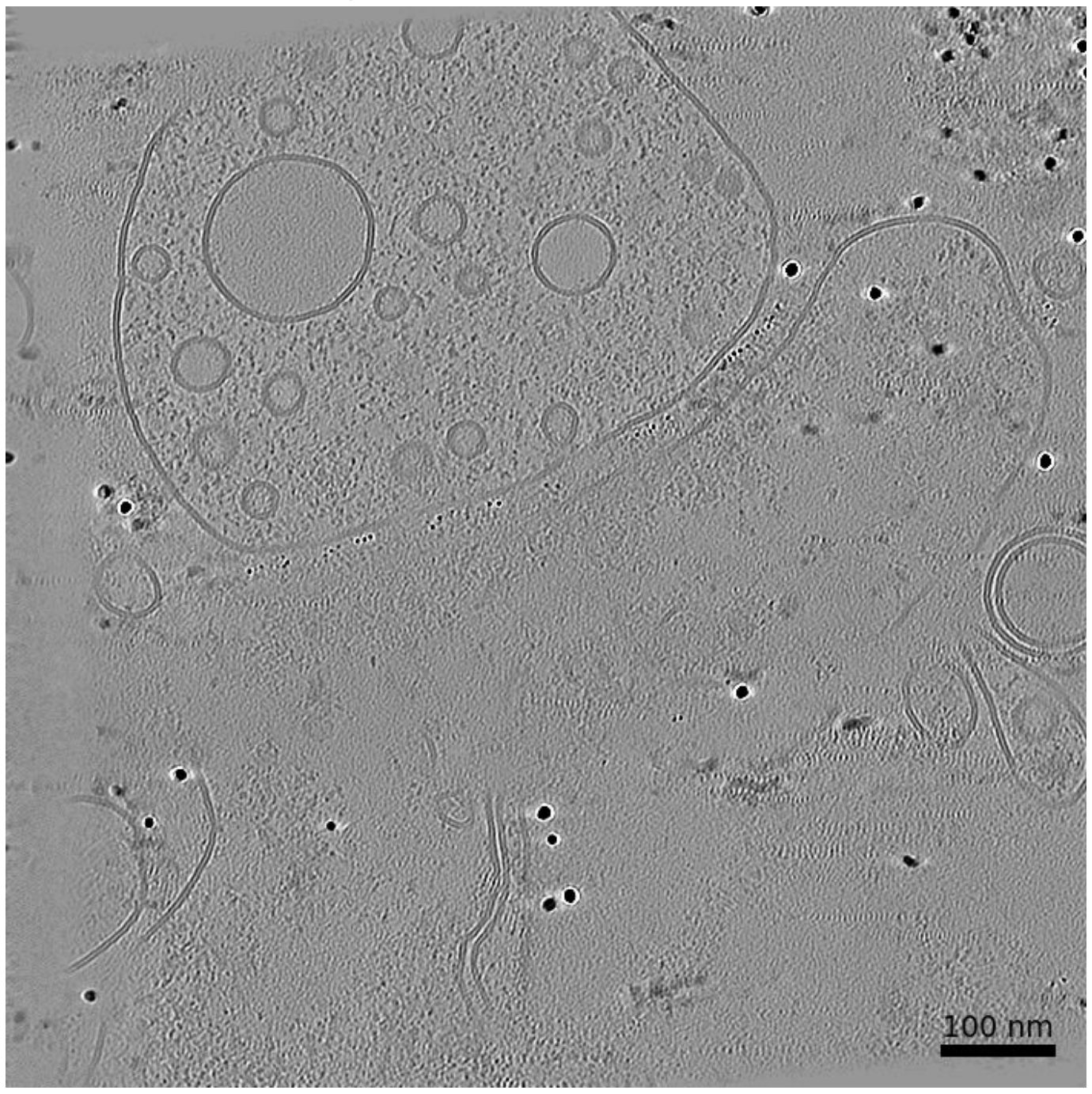

Membrane Segmentation & Cleft Position

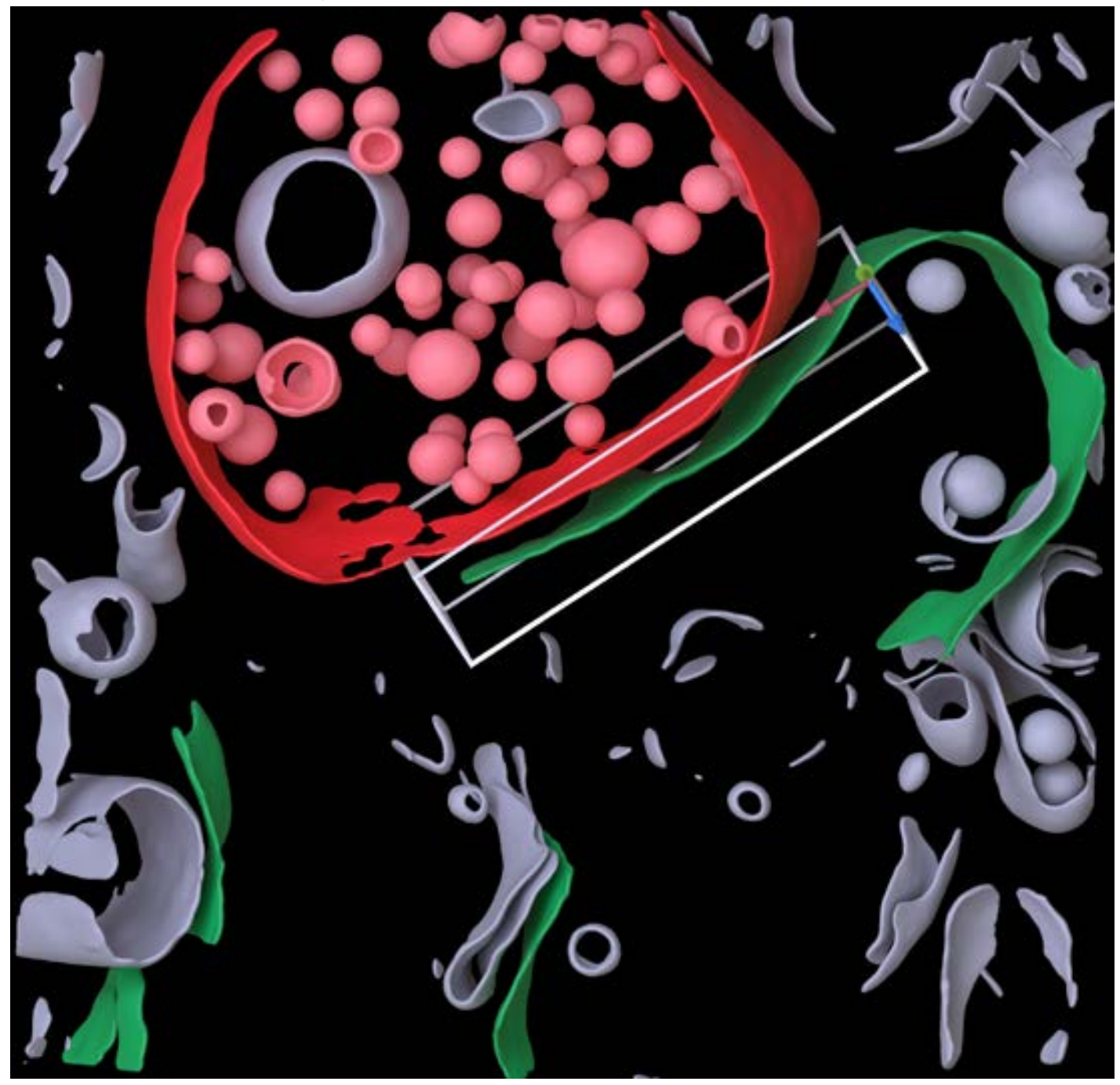

Cleft MIP

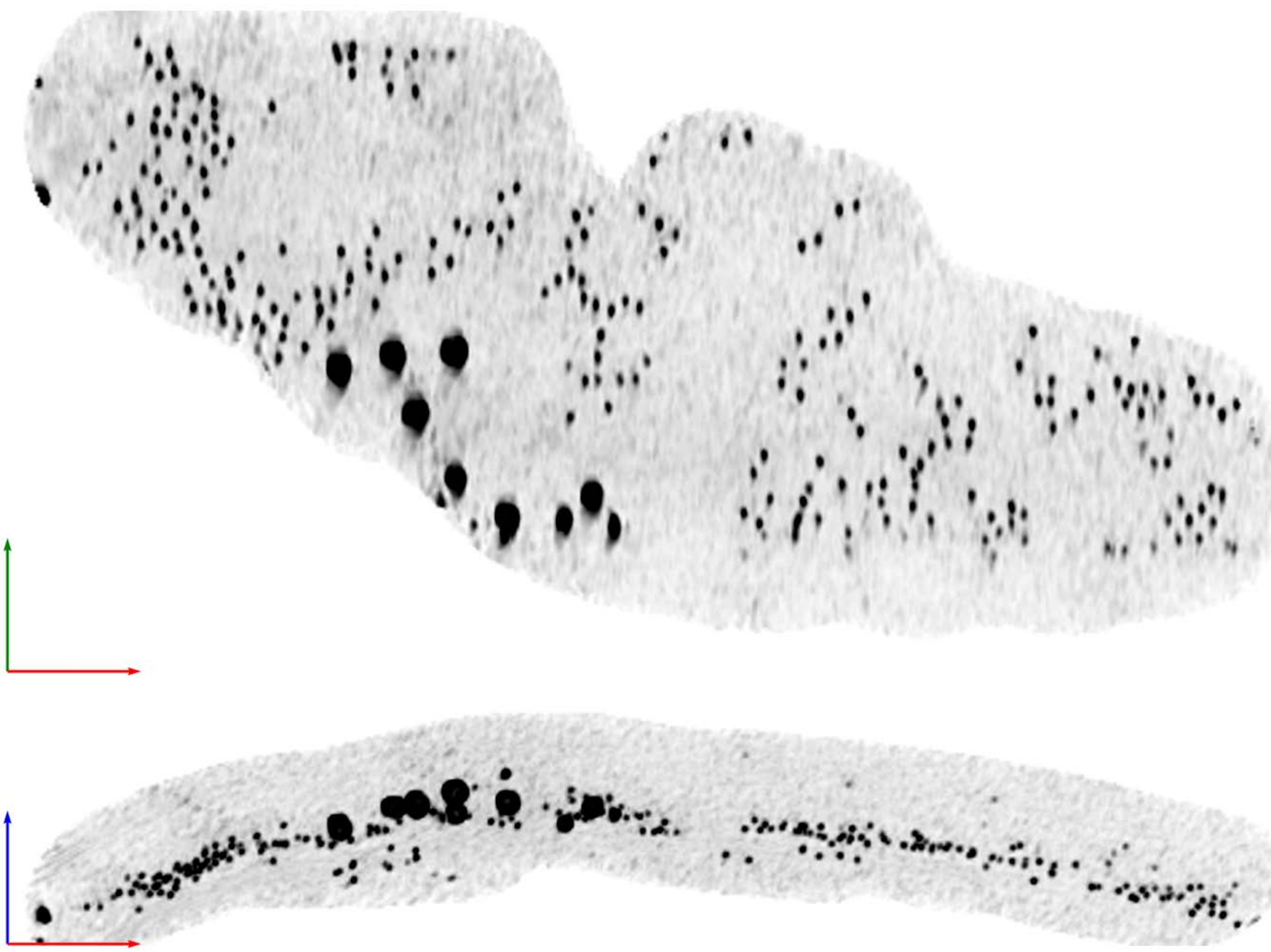

Tomogram ID: 20240209_waffleHip_78	SET: anti-GluA2 15F1 "C-term"
Cleft ID: 0	Type: semi-tissue

Denoised Tomogram Slice

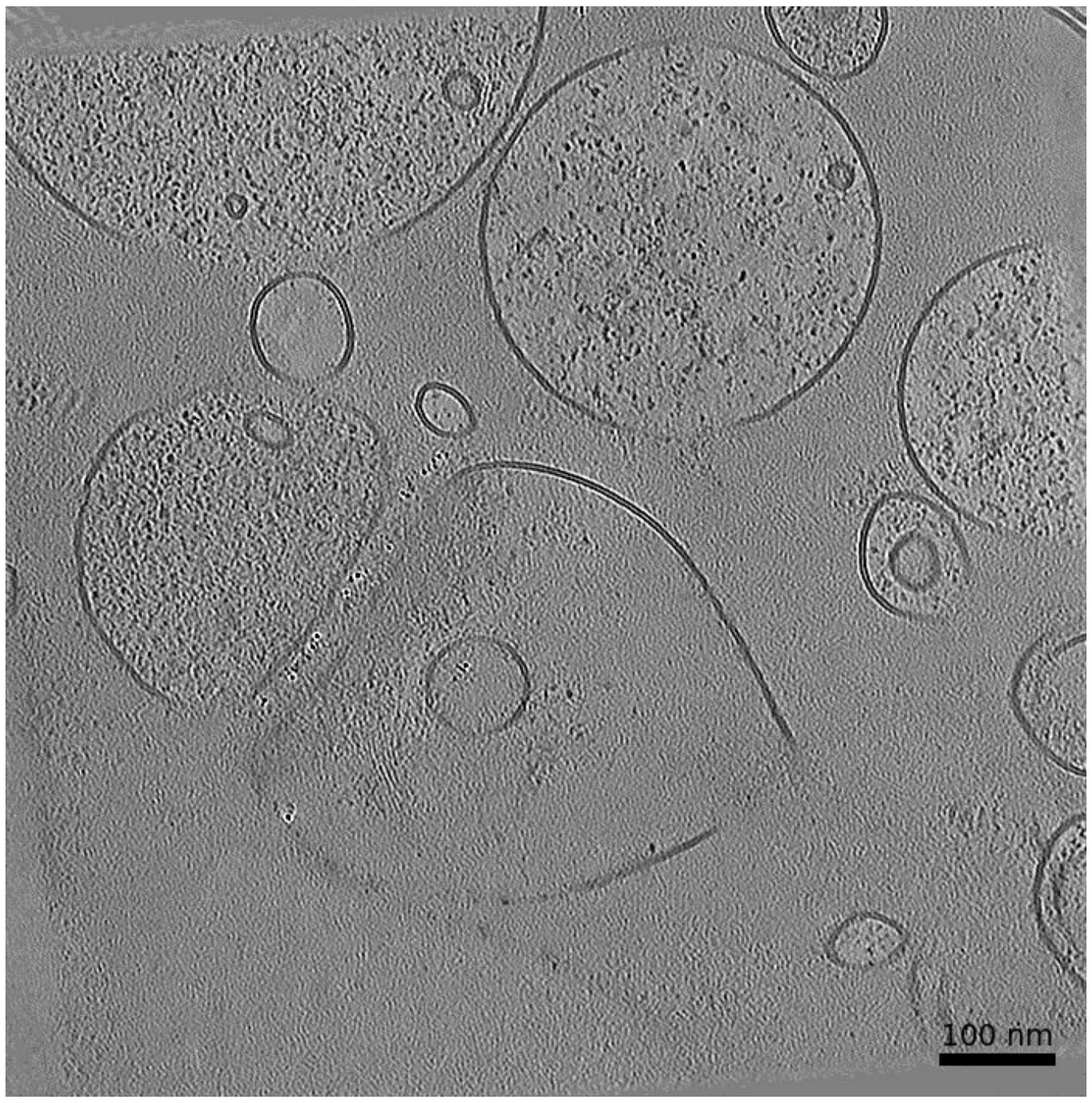

Membrane Segmentation & Cleft Position

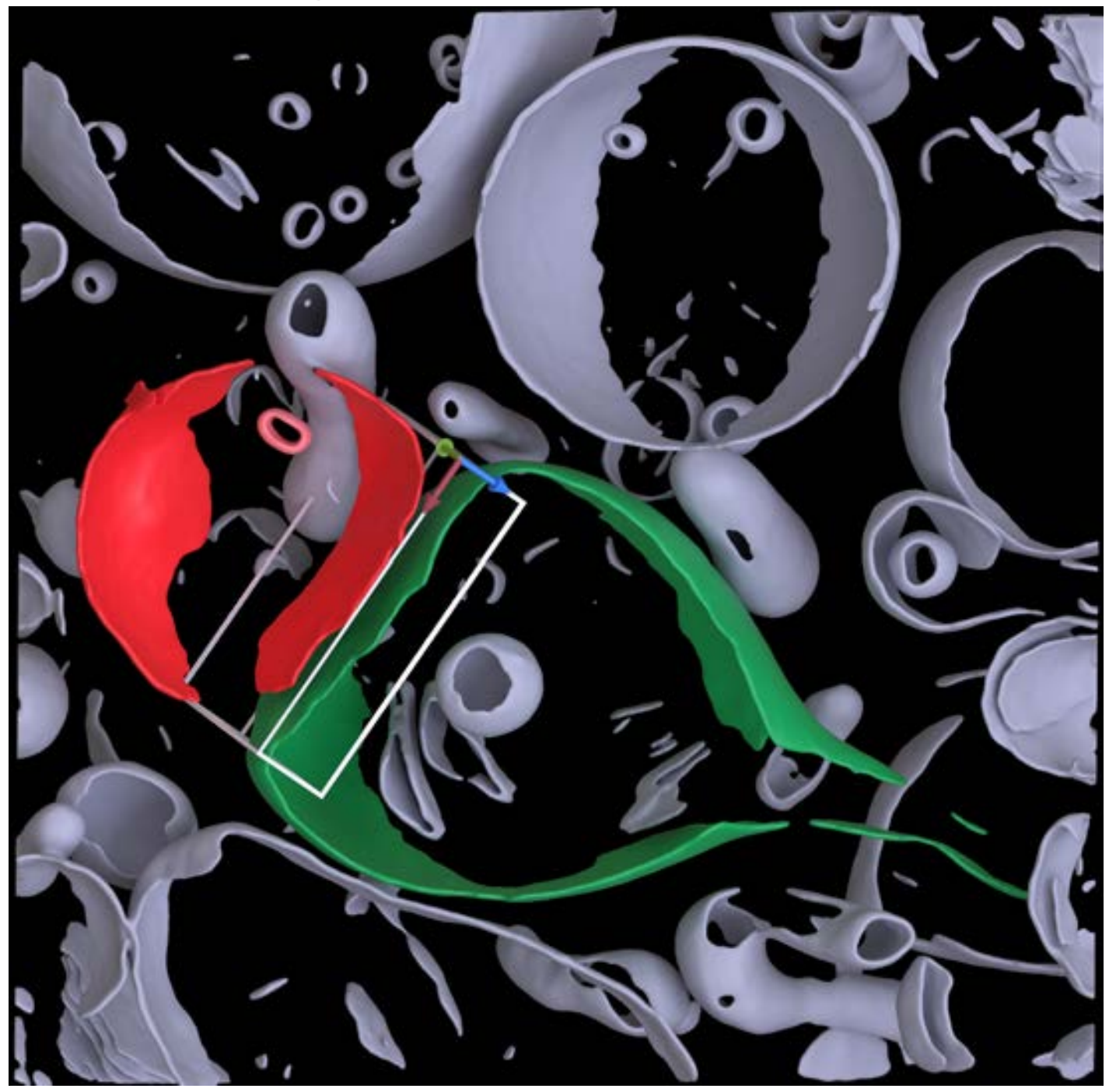

Cleft MIP

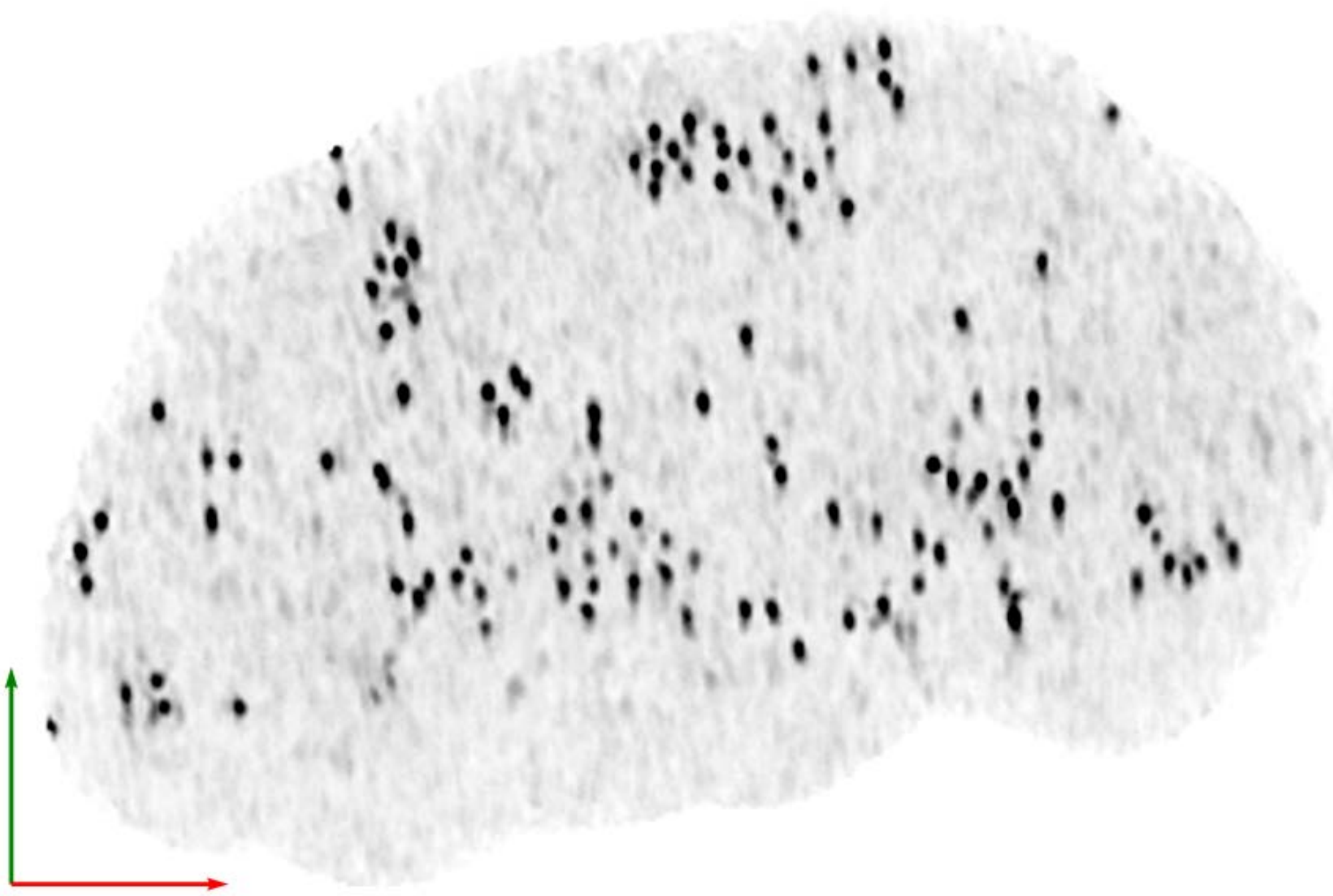

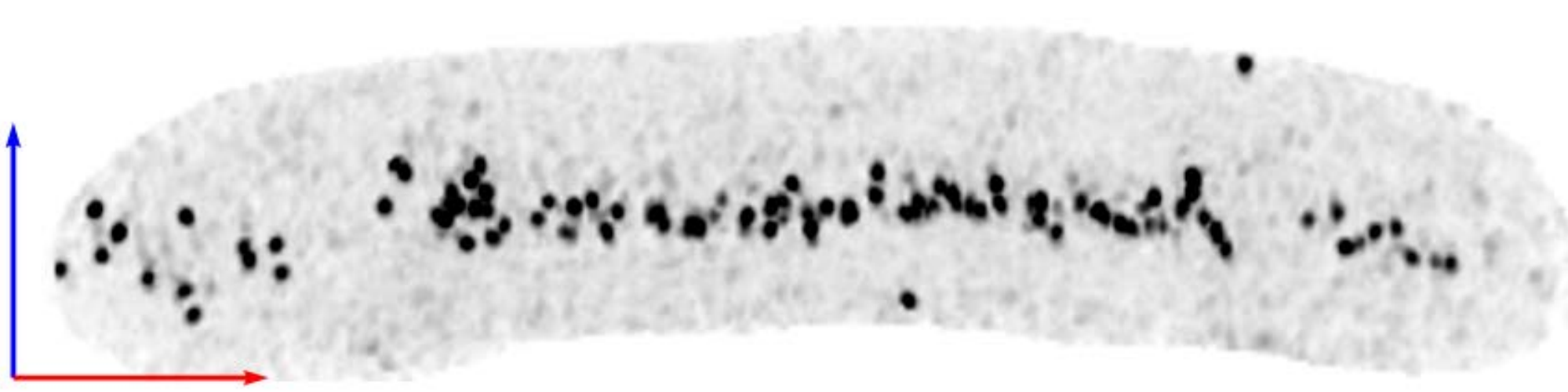

Tomogram ID: 20240523_HippWaffle_71 | **SET: anti-GluA2 15F1 "C-term"**

Cleft ID: 0 | Type: tissue

Denoised Tomogram Slice

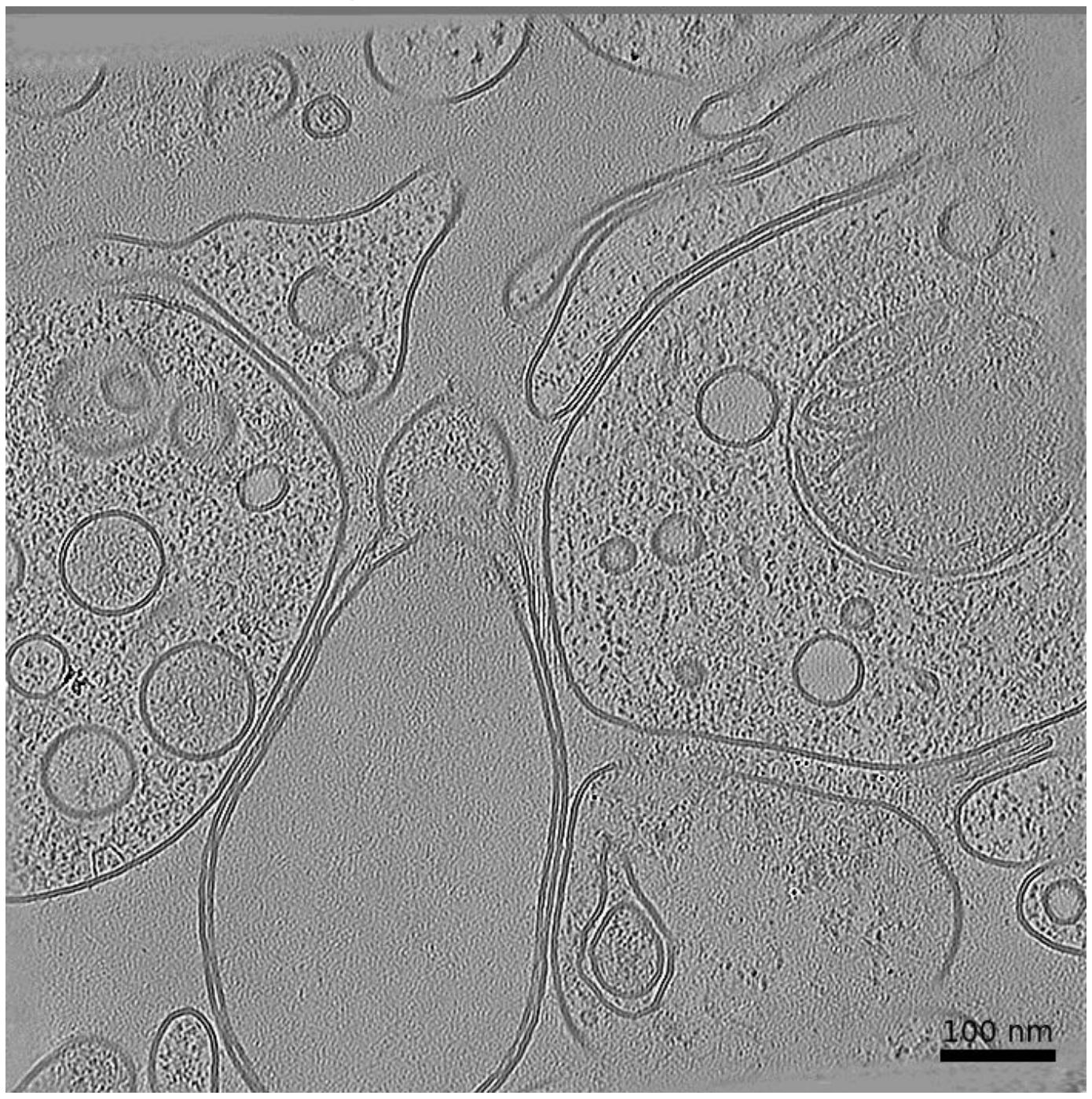

Membrane Segmentation & Cleft Position

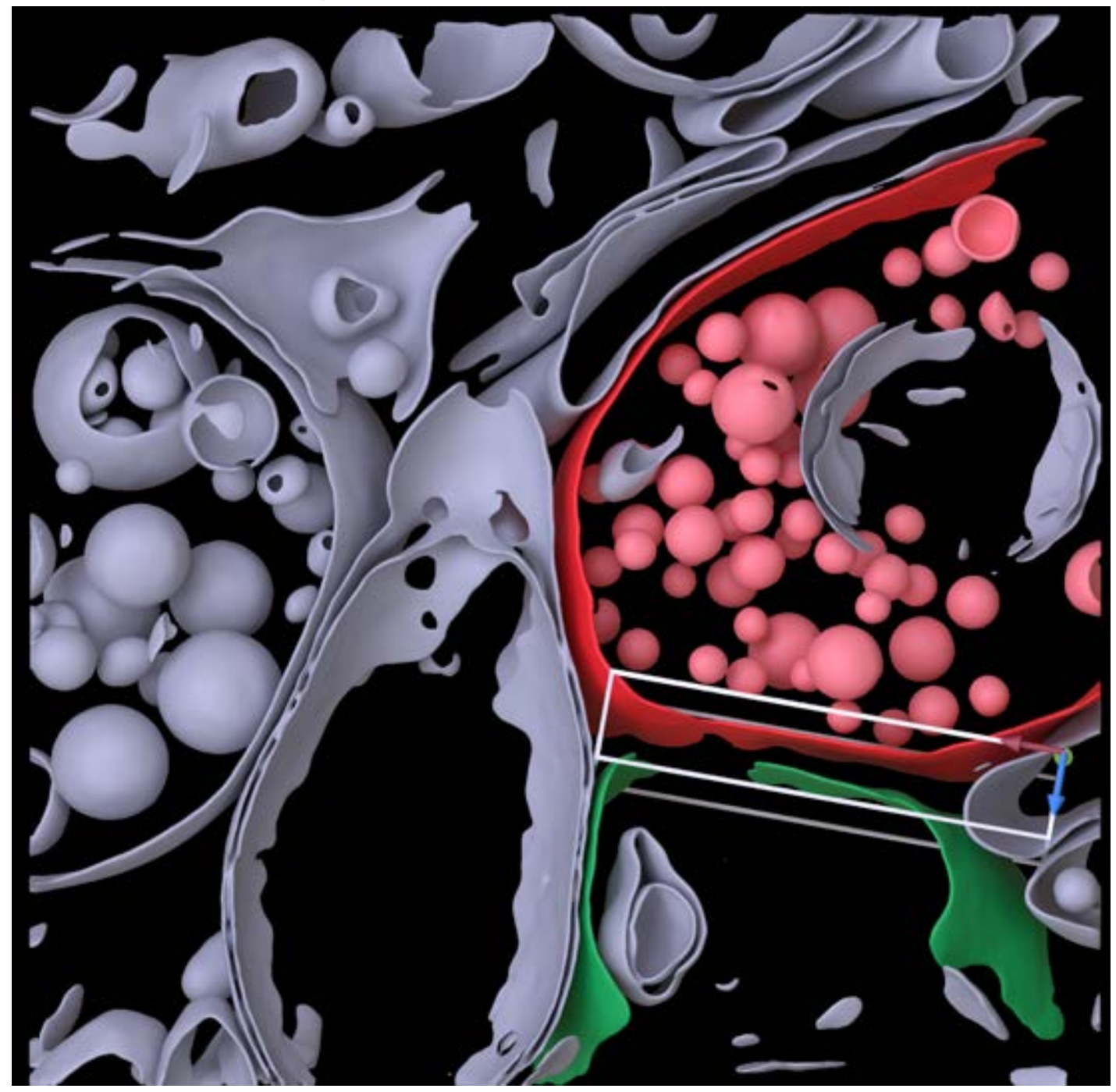

Cleft MIP

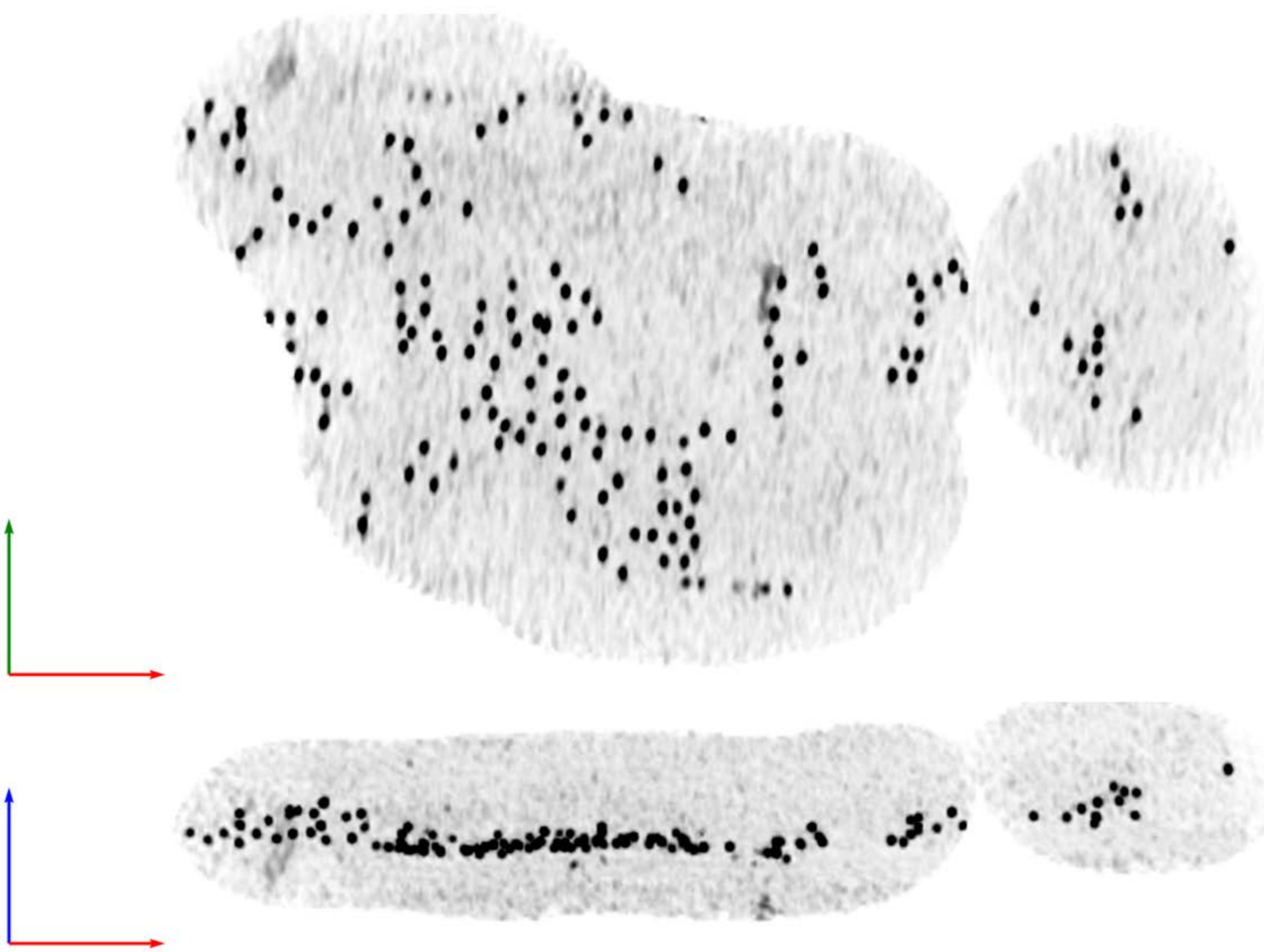

Tomogram ID: 20240624_HippWaffle_21 | **SET: anti-GluA2 15F1 "C-term"**

Cleft ID: 0 | Type: tissue

Denoised Tomogram Slice

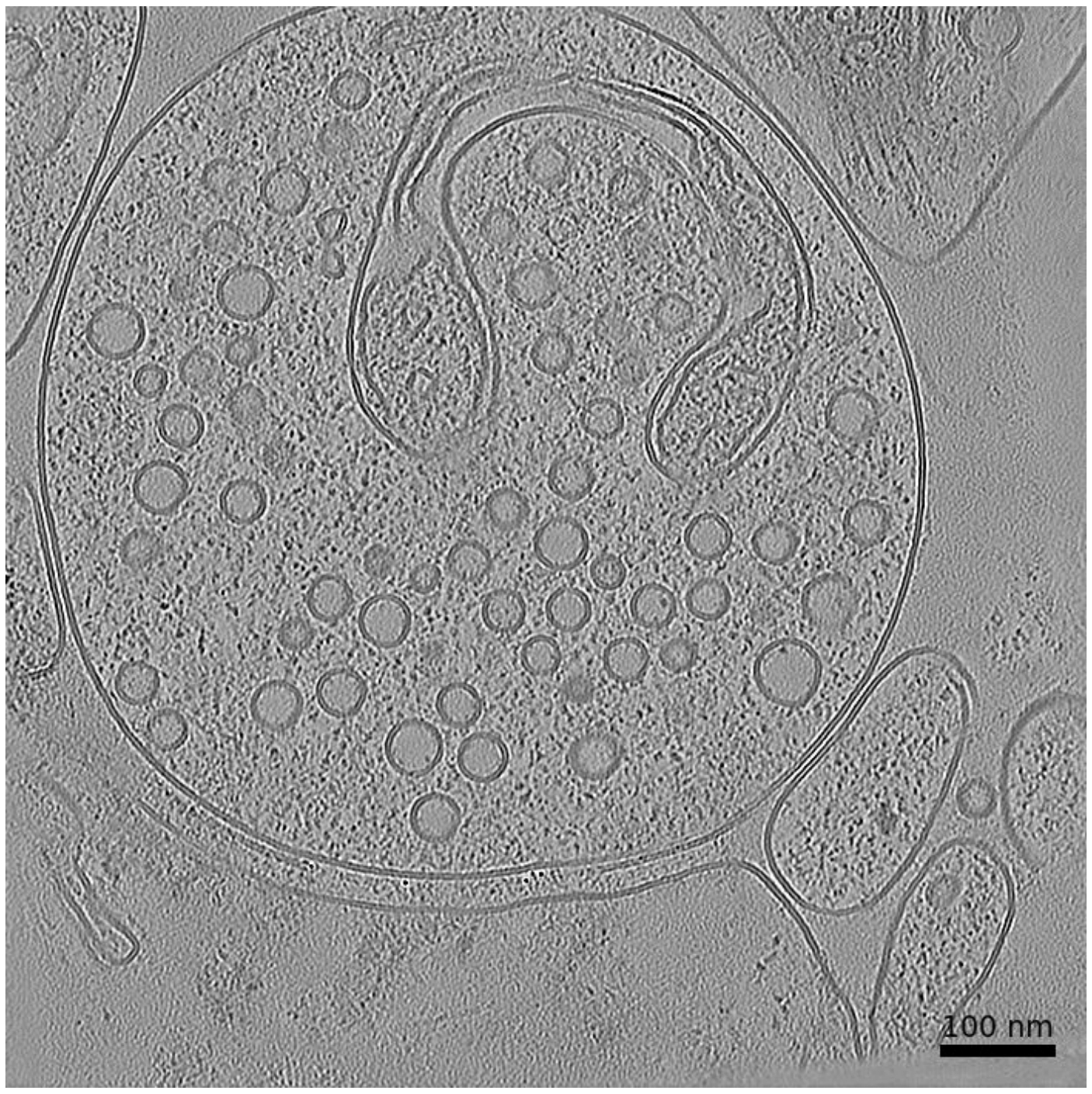

Membrane Segmentation & Cleft Position

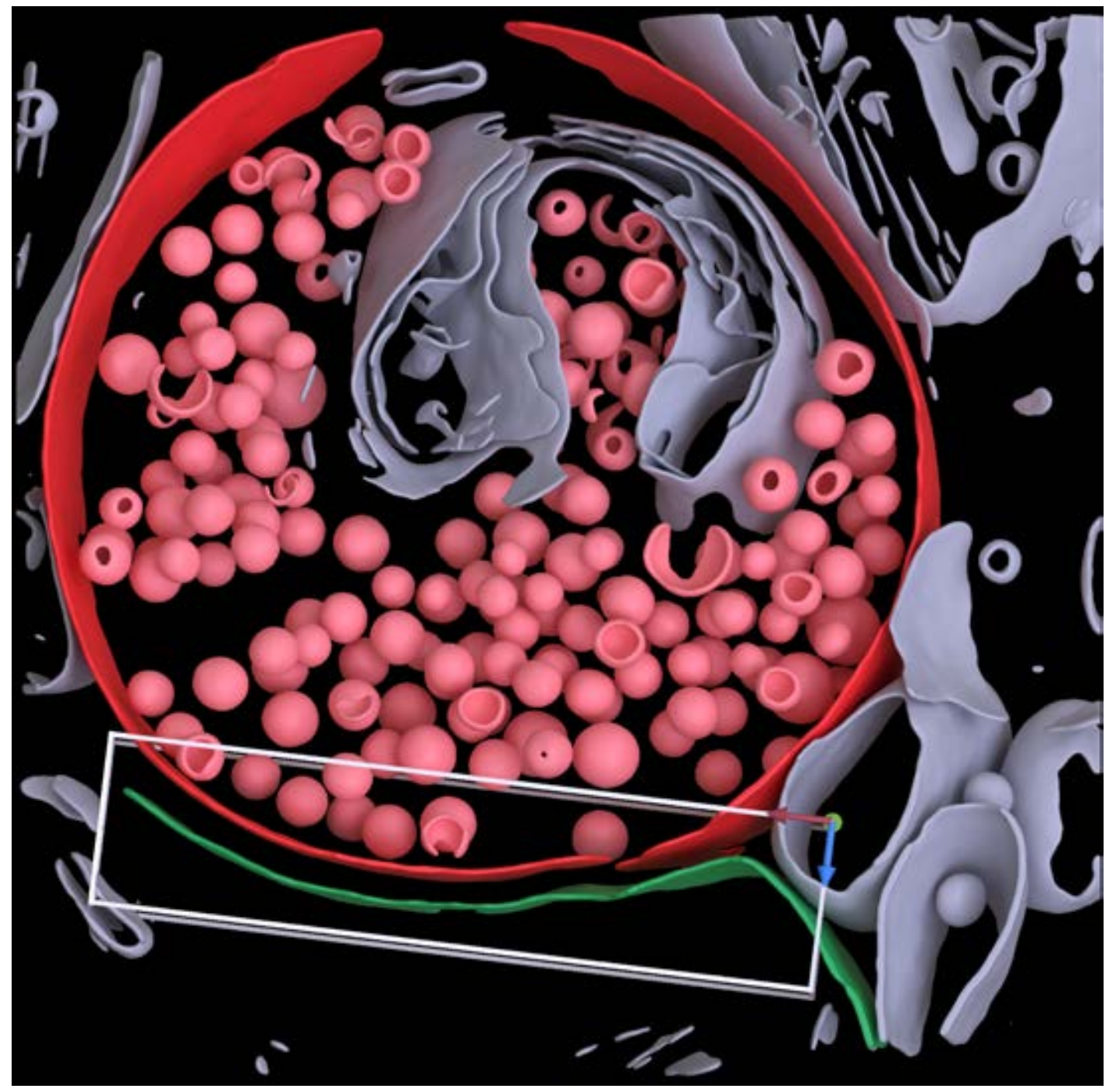

Cleft MIP

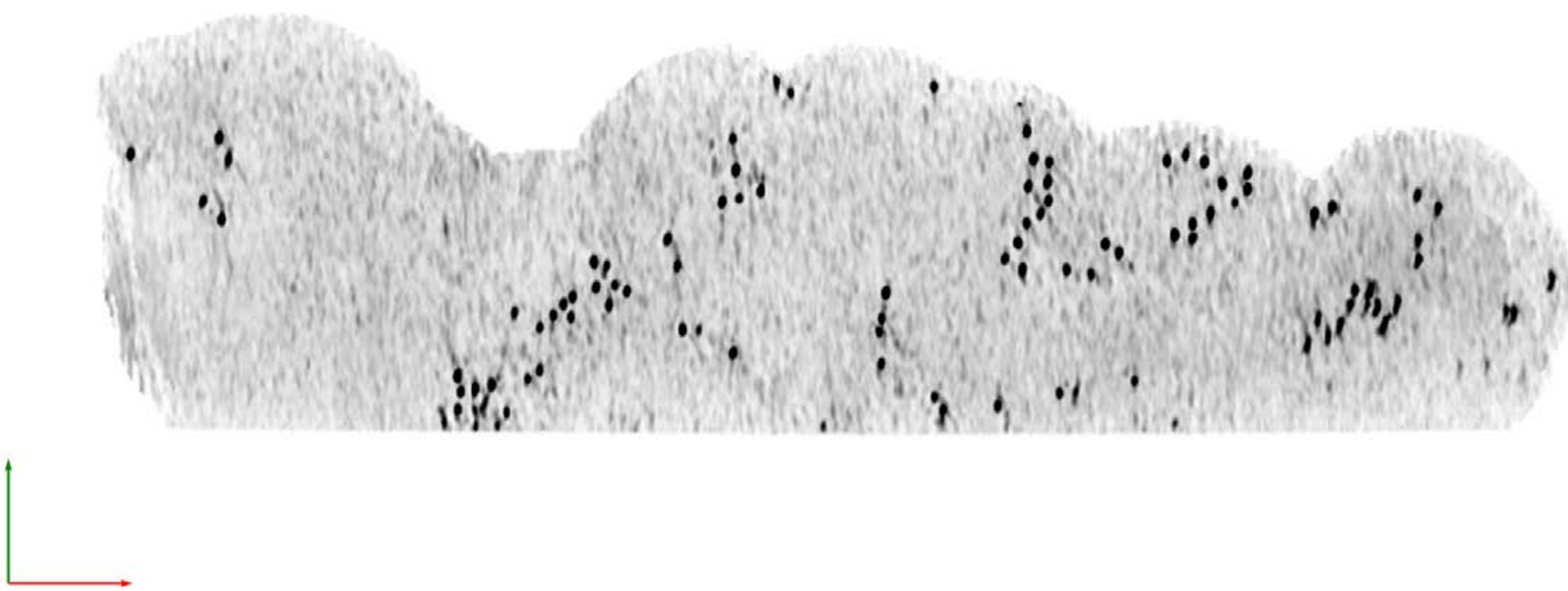

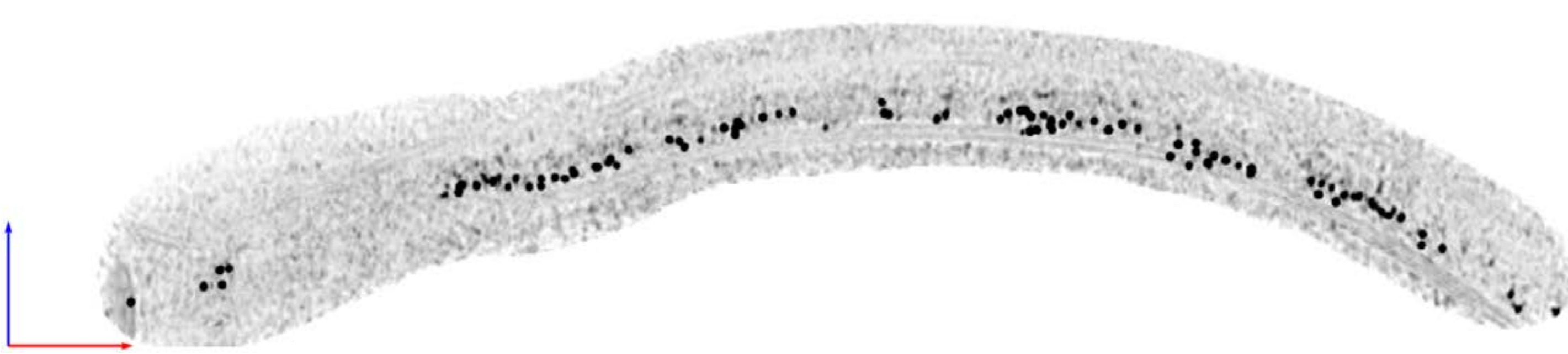

Tomogram ID: 20240624_HippWaffle_76	SET: anti-GluA2 15F1 "C-term"
Cleft ID: 0	Type: tissue

Denoised Tomogram Slice

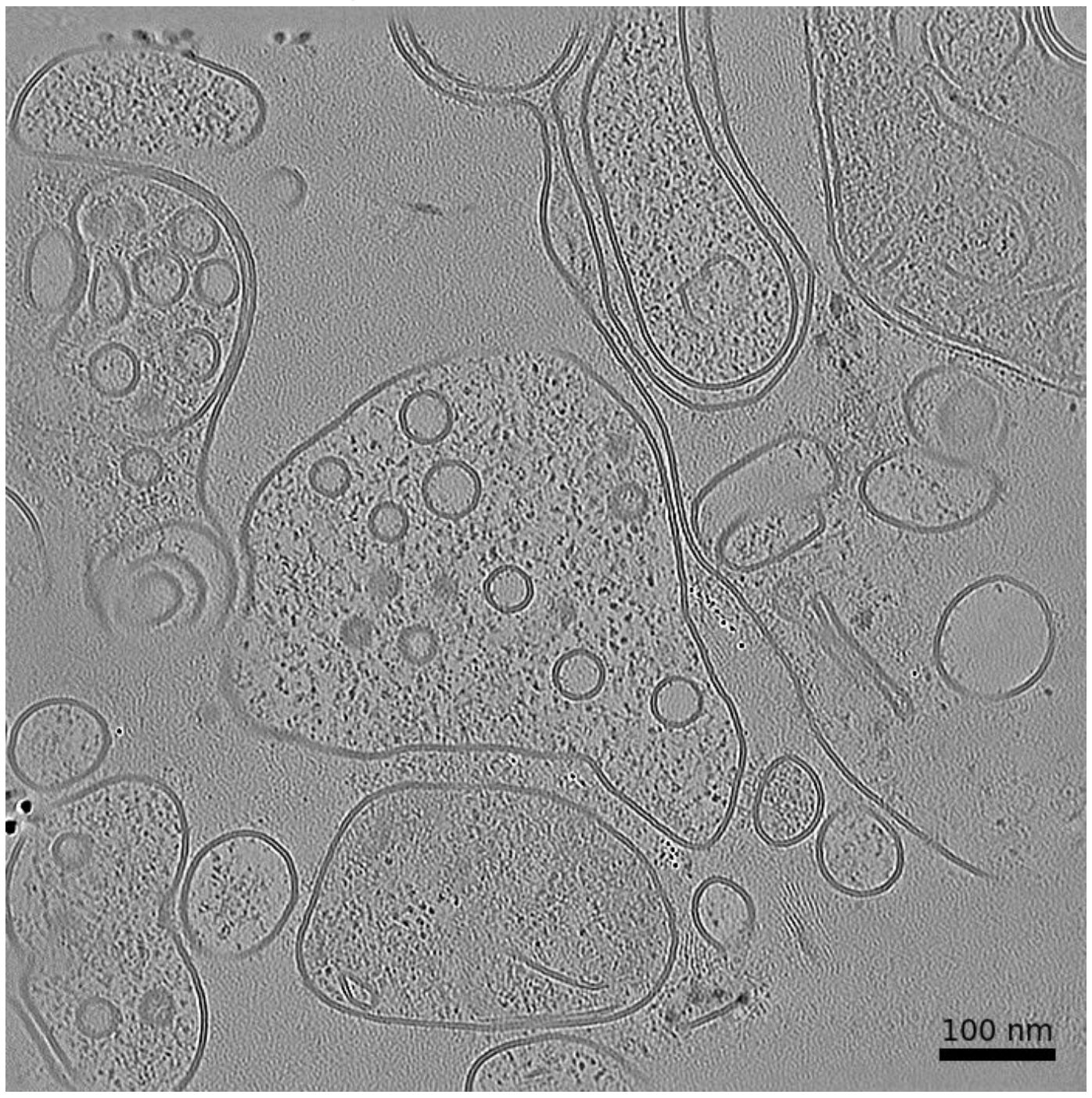

Membrane Segmentation & Cleft Position

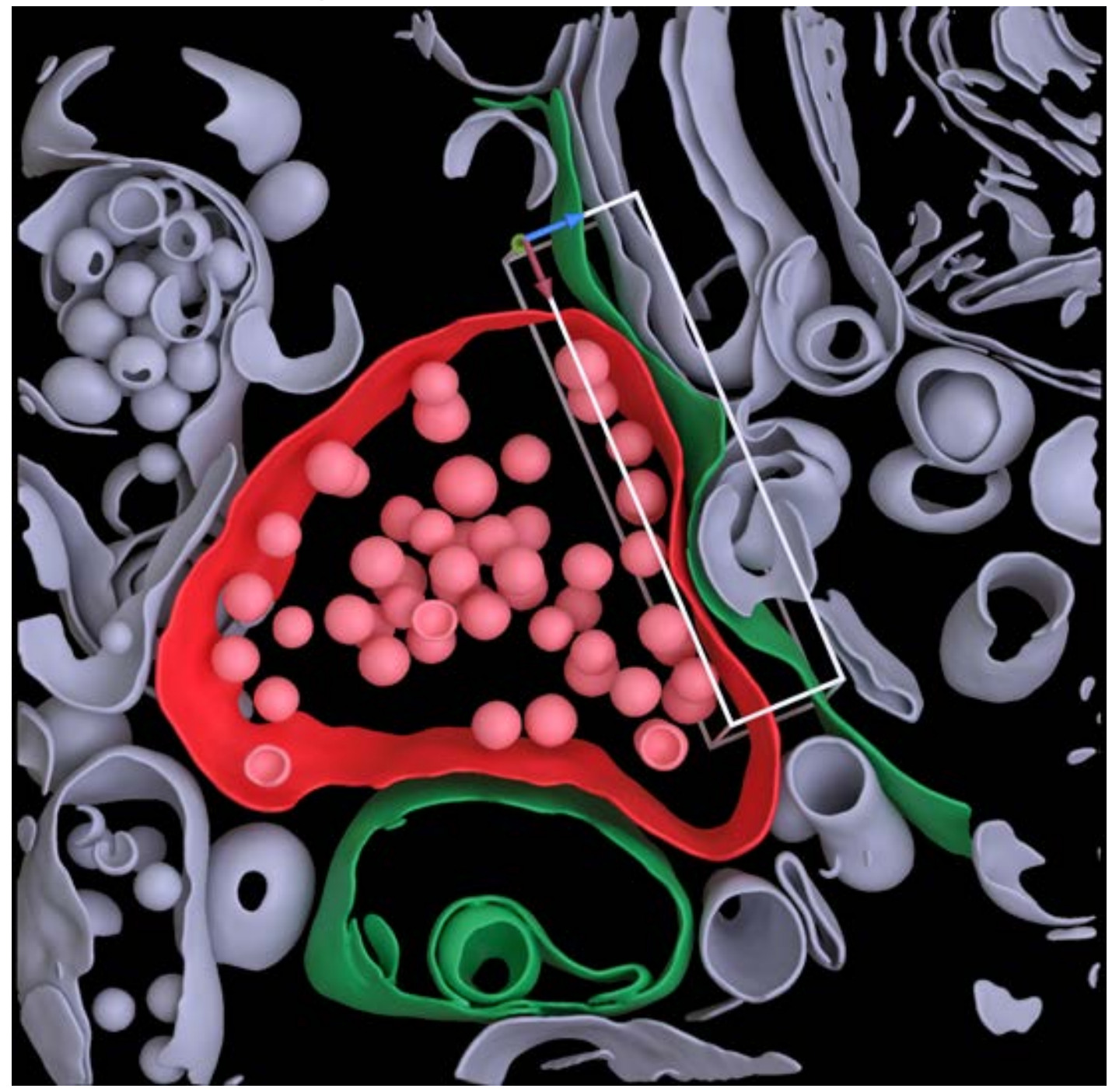

Cleft MIP

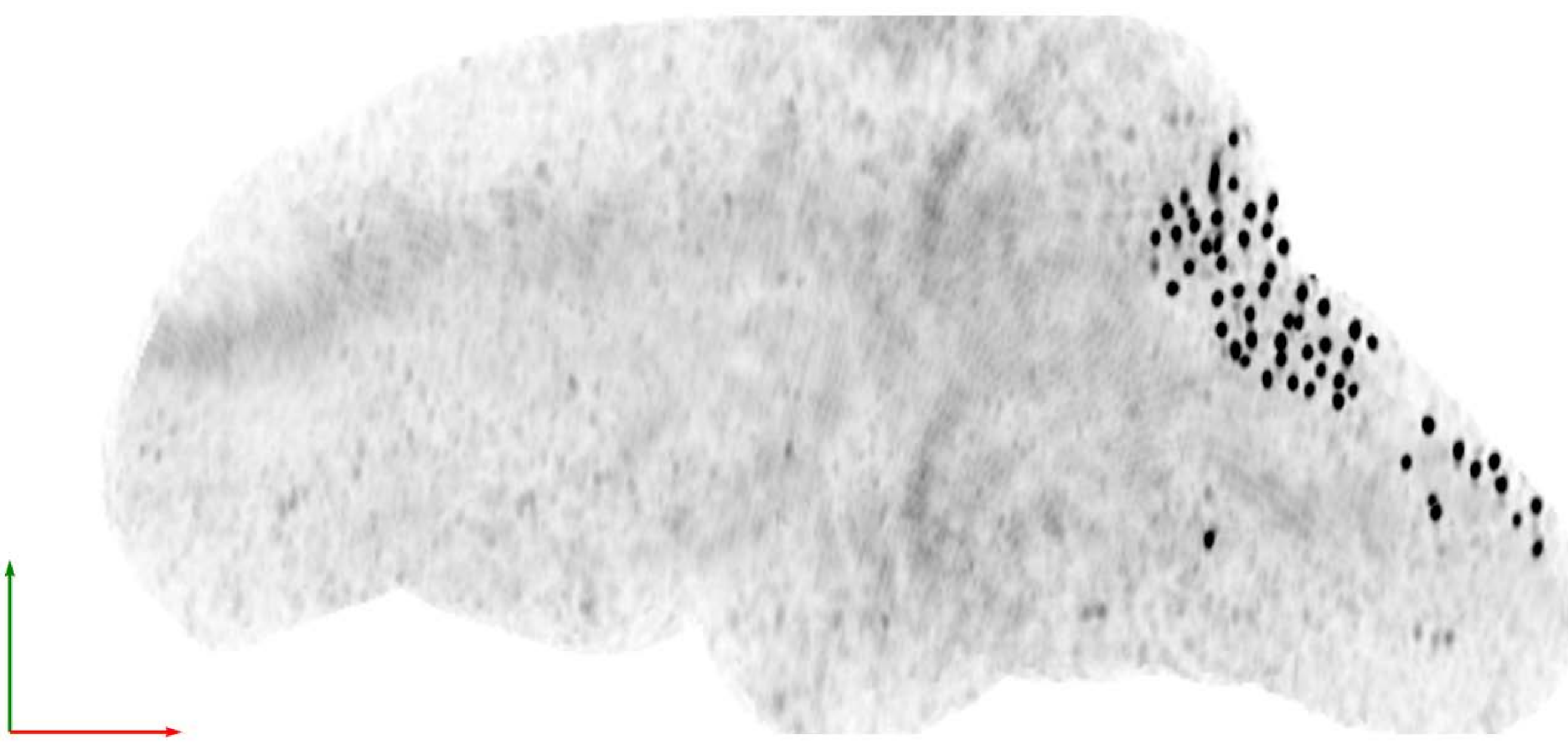

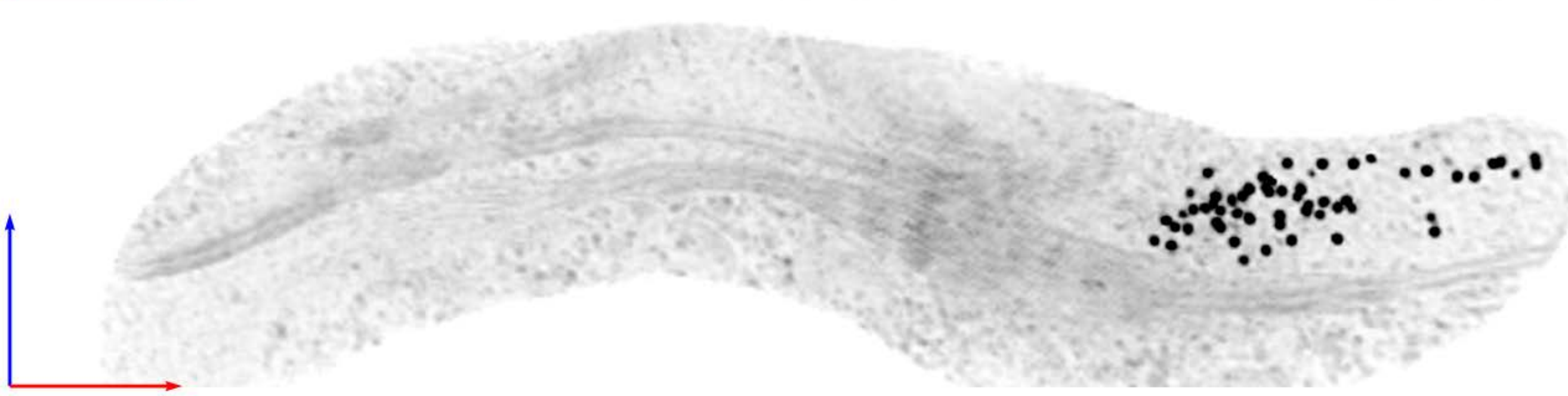

Tomogram ID: 20240624_HippWaffle_76 **SET: anti-GluA2 15F1 "C-term"**

Cleft ID: 1 Type: tissue

Denoised Tomogram Slice

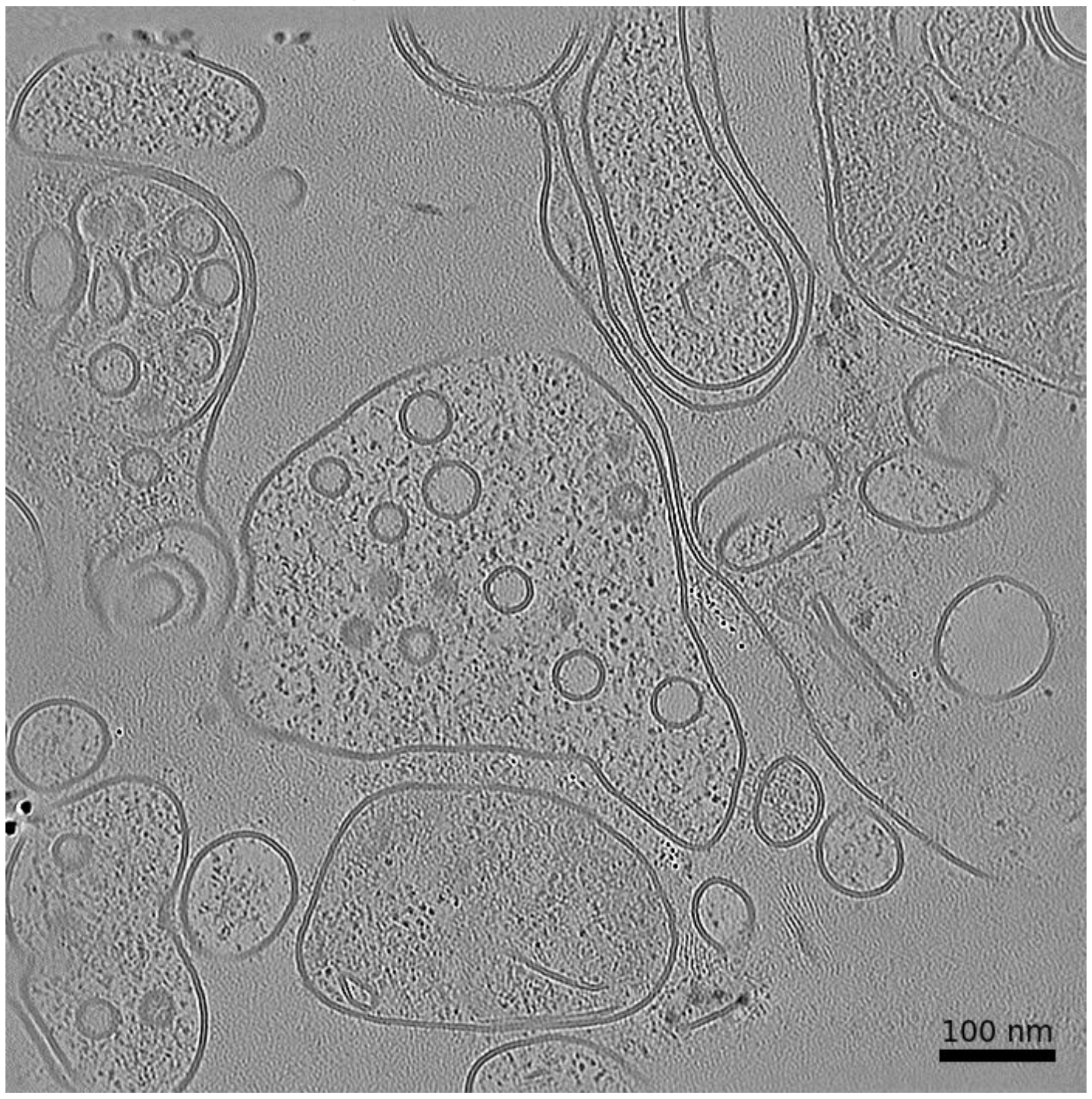

Membrane Segmentation & Cleft Position

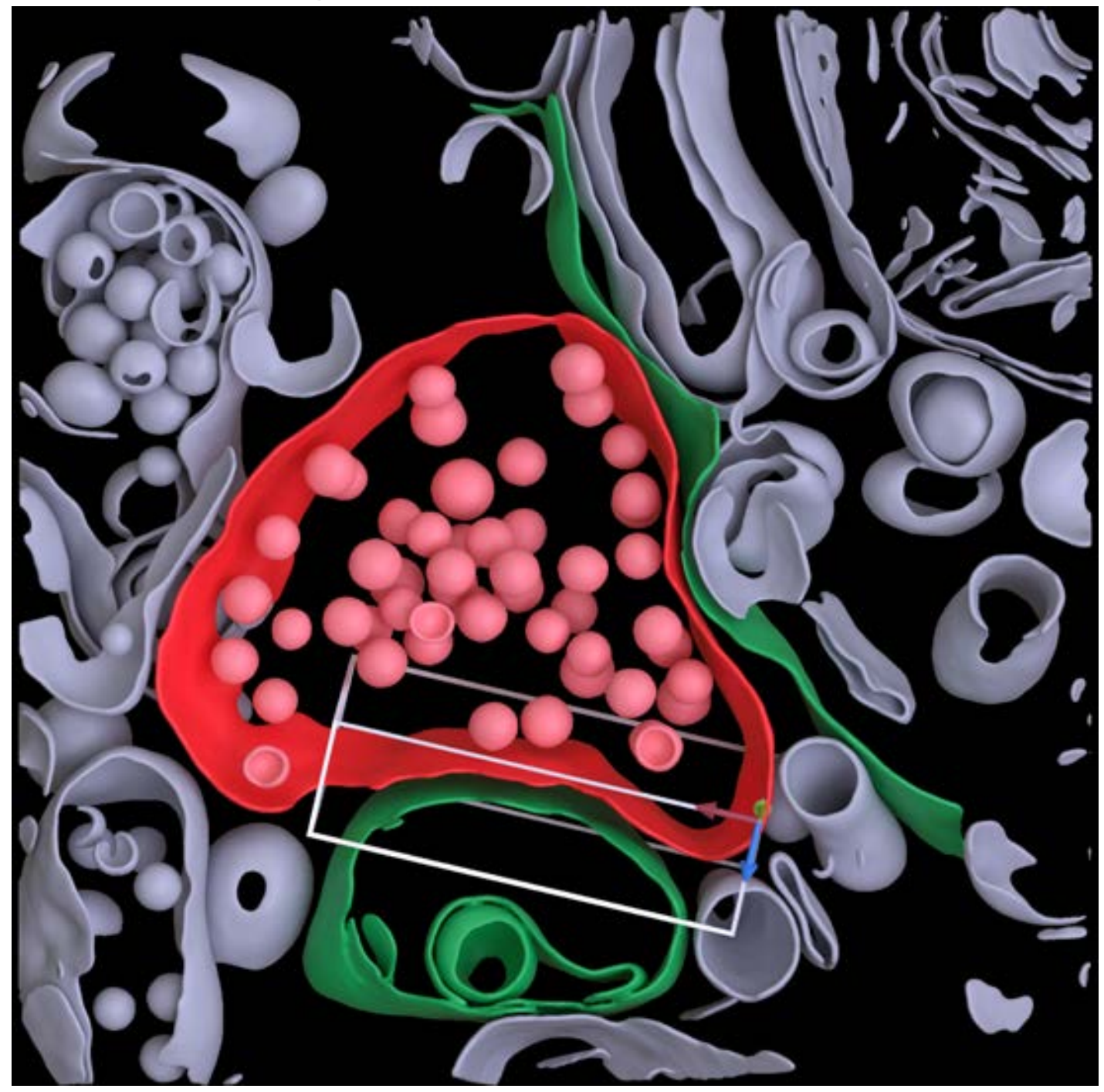

Cleft MIP

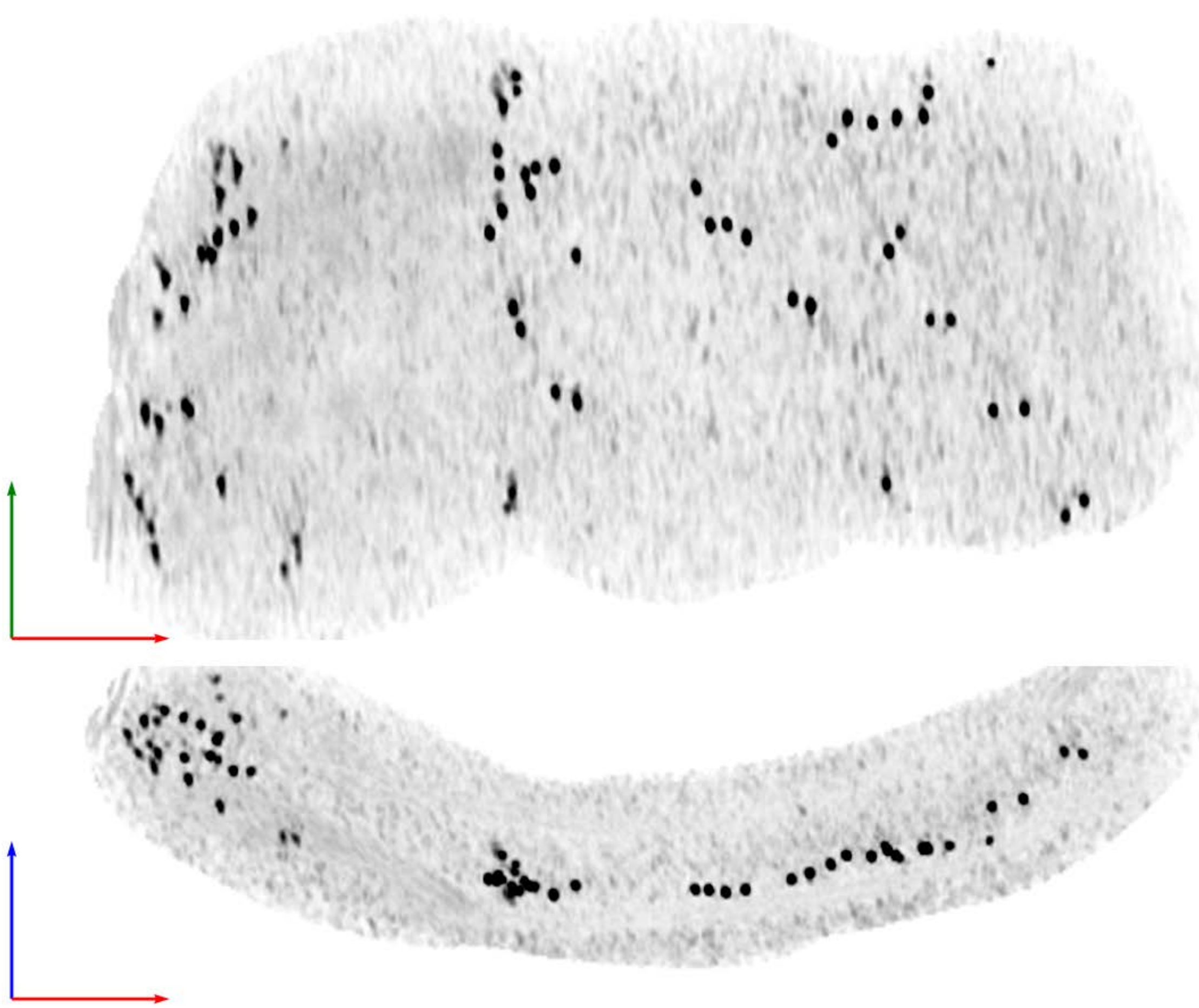

Tomogram ID: 20240624_HippWaffle_86	SET: anti-GluA2 15F1 "C-term"
Cleft ID: 0	Type: tissue

Denoised Tomogram Slice

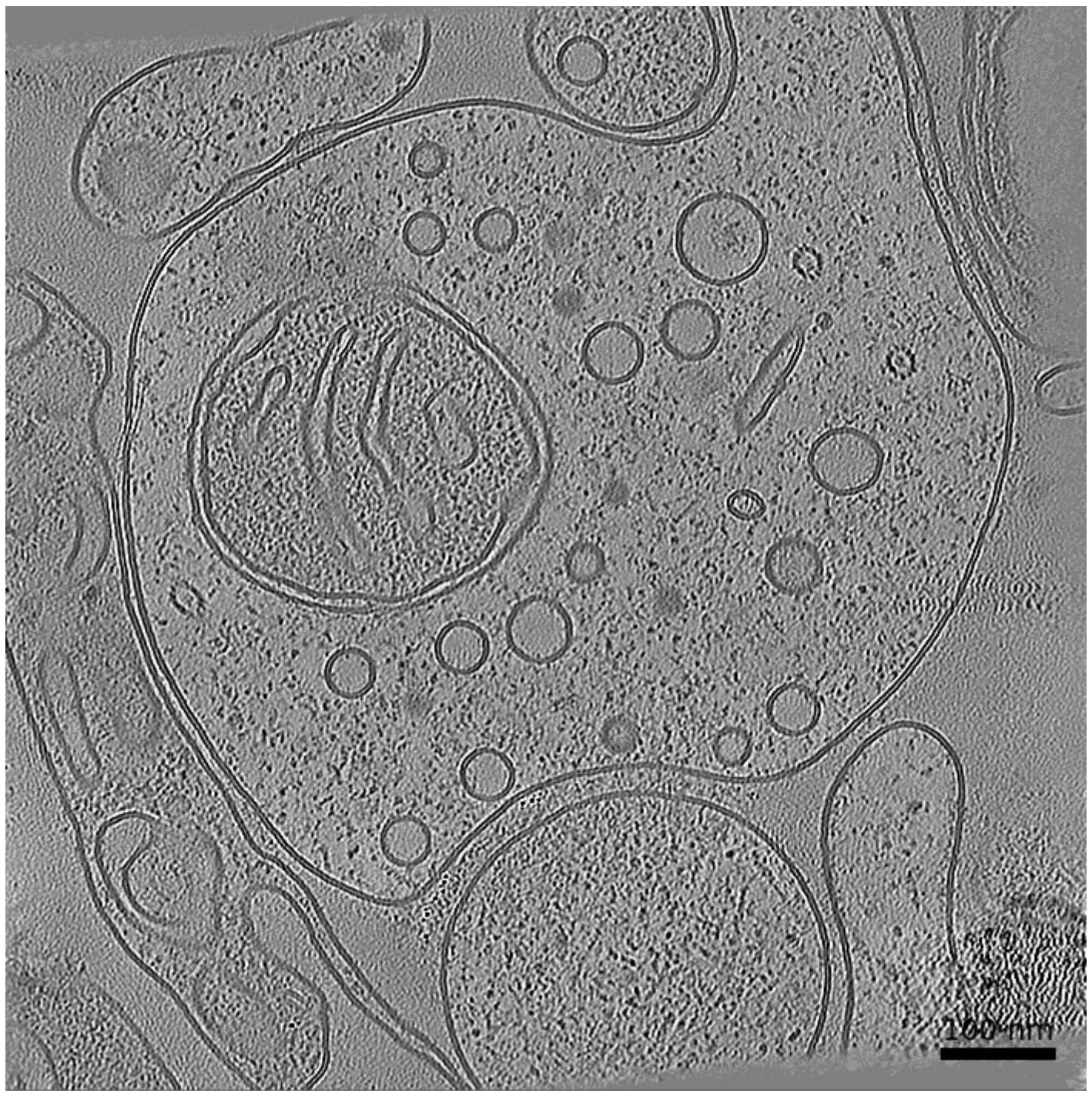

Membrane Segmentation & Cleft Position

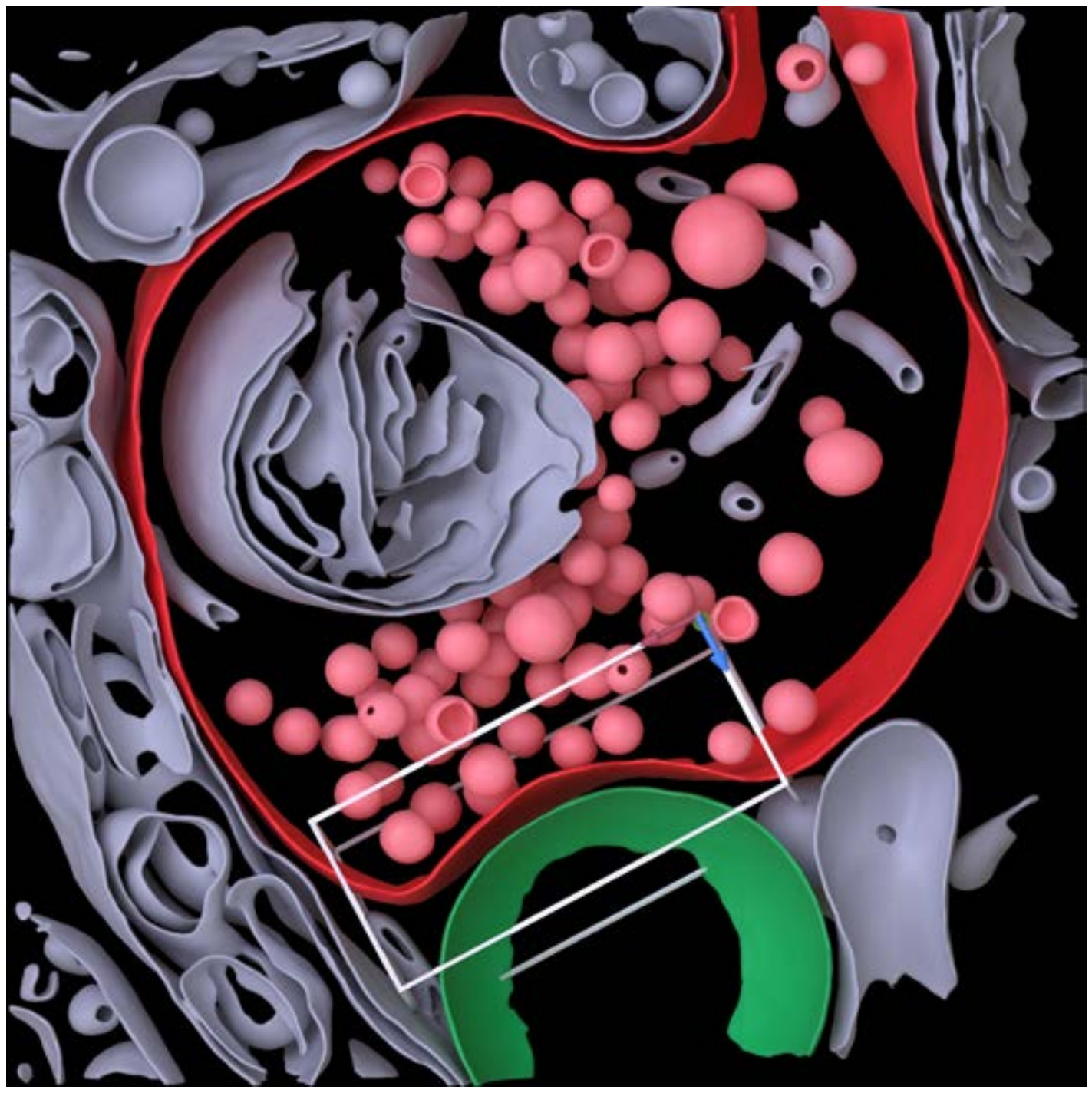

Cleft MIP

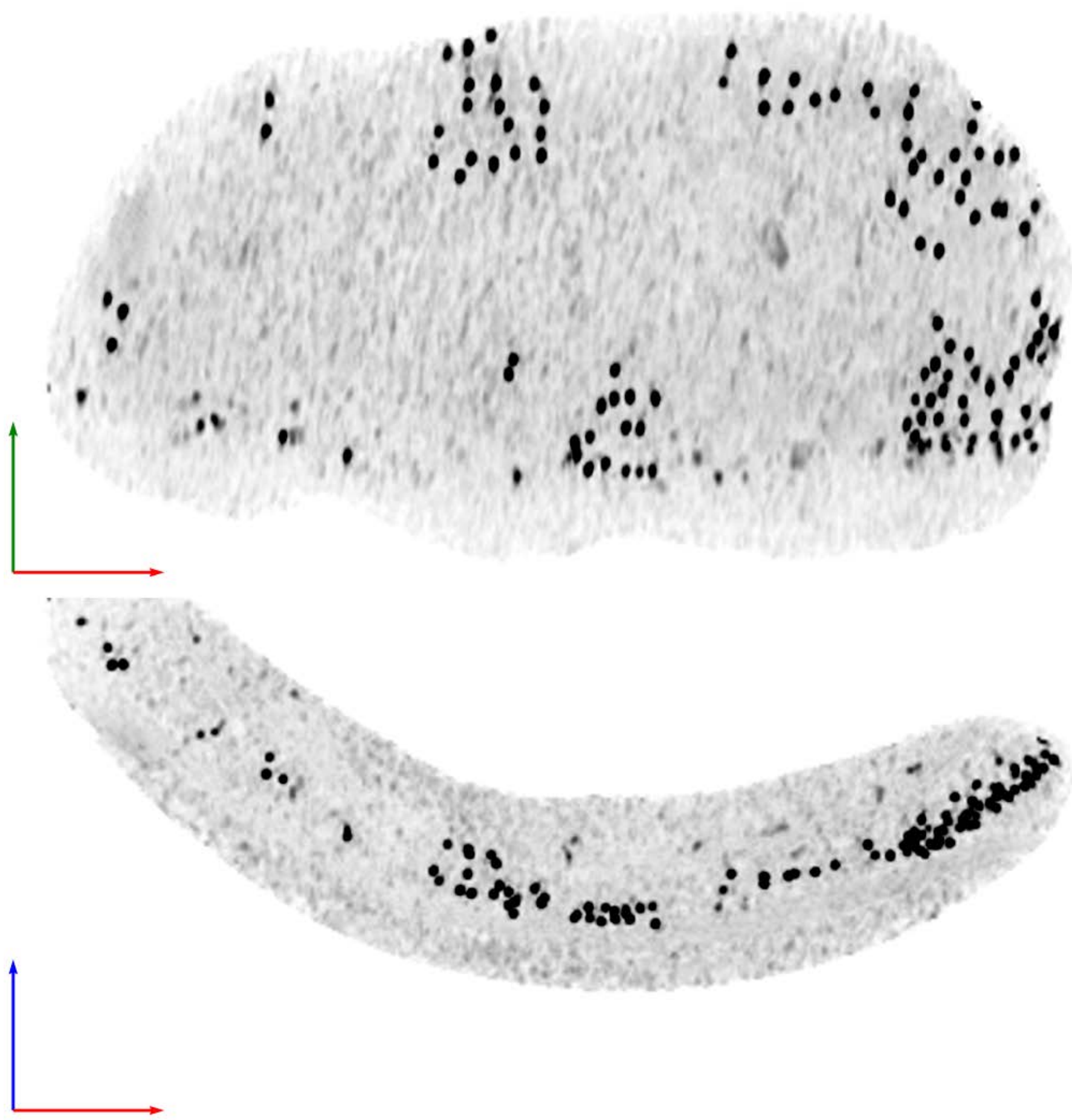

Tomogram ID: 20241030_AMmilled12-1_12	SET: anti-GluA2 15F1 "C-term"
Cleft ID: 0	Type: tissue

Denoised Tomogram Slice

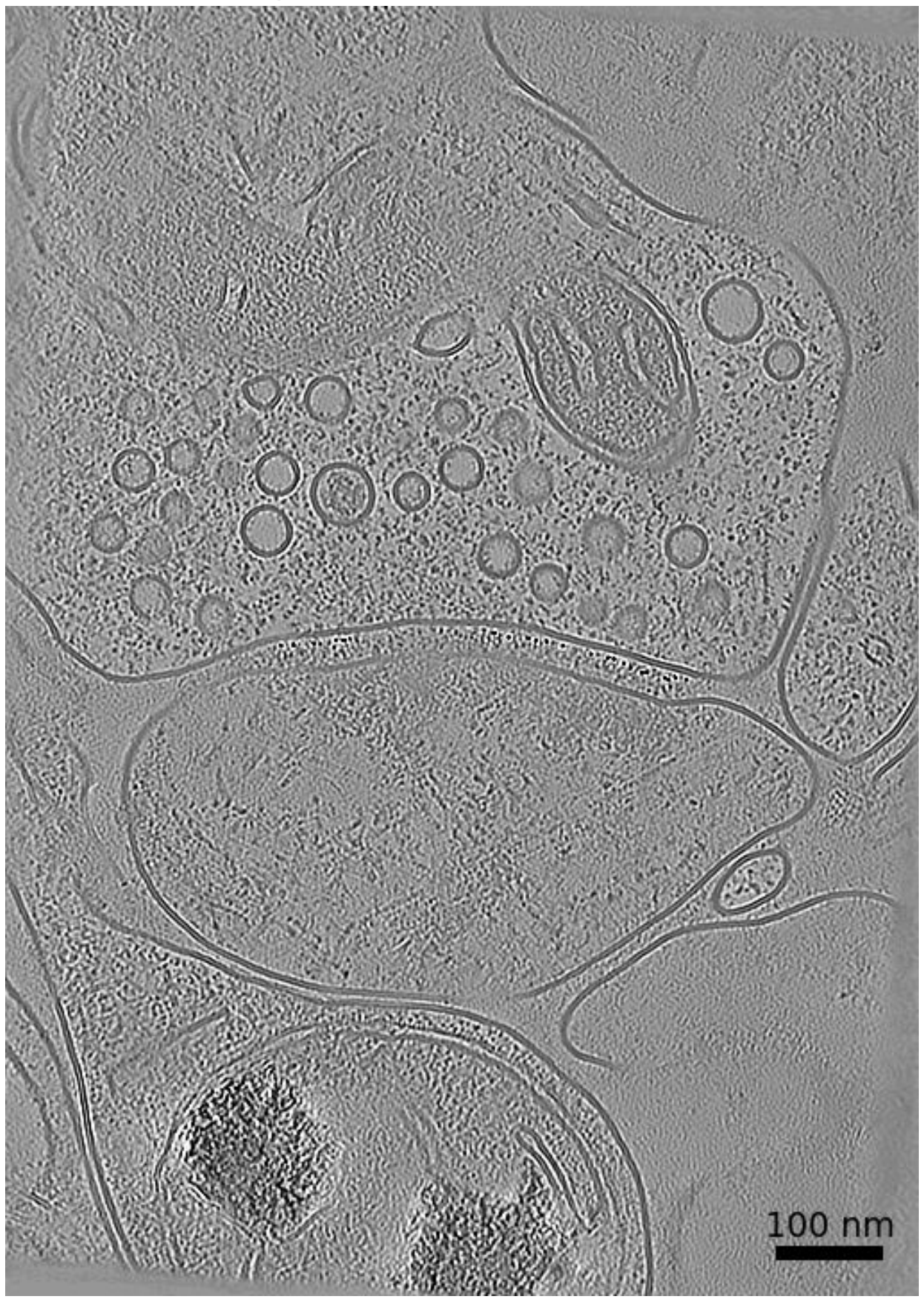

Membrane Segmentation & Cleft Position

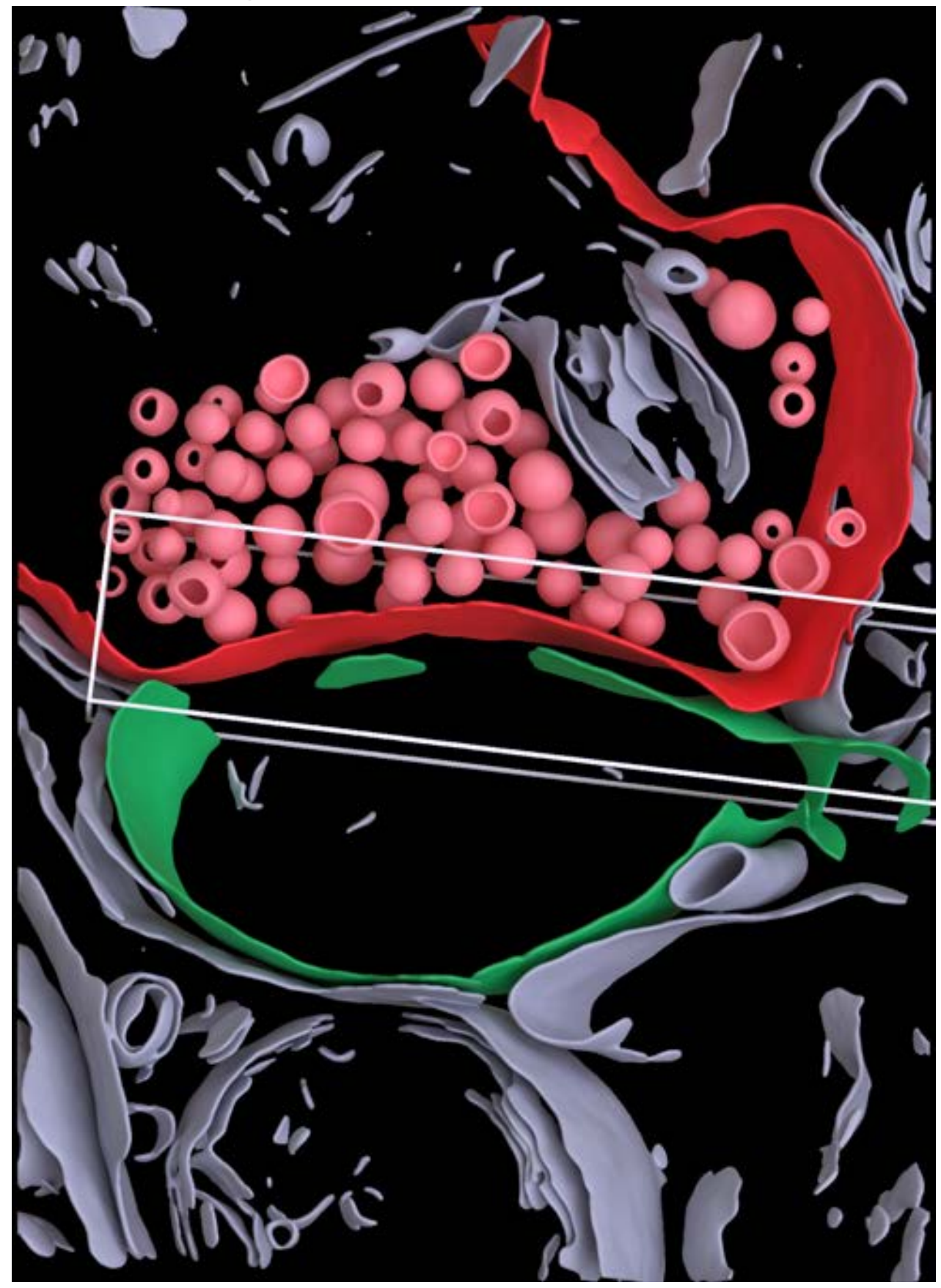

Cleft MIP

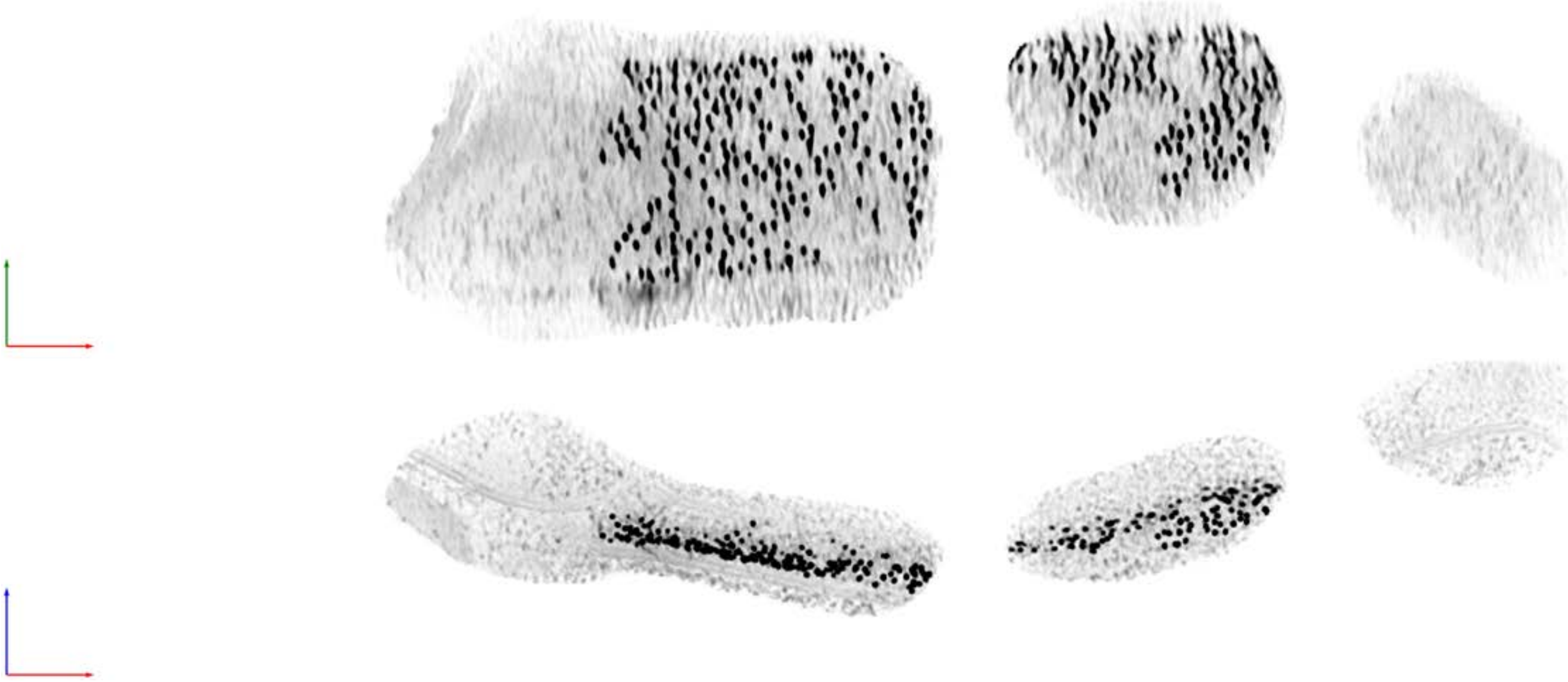

Tomogram ID: 20241030_AMmilled12-1_15	SET: anti-GluA2 15F1 "C-term"
Cleft ID: 0	Type: tissue

Denoised Tomogram Slice

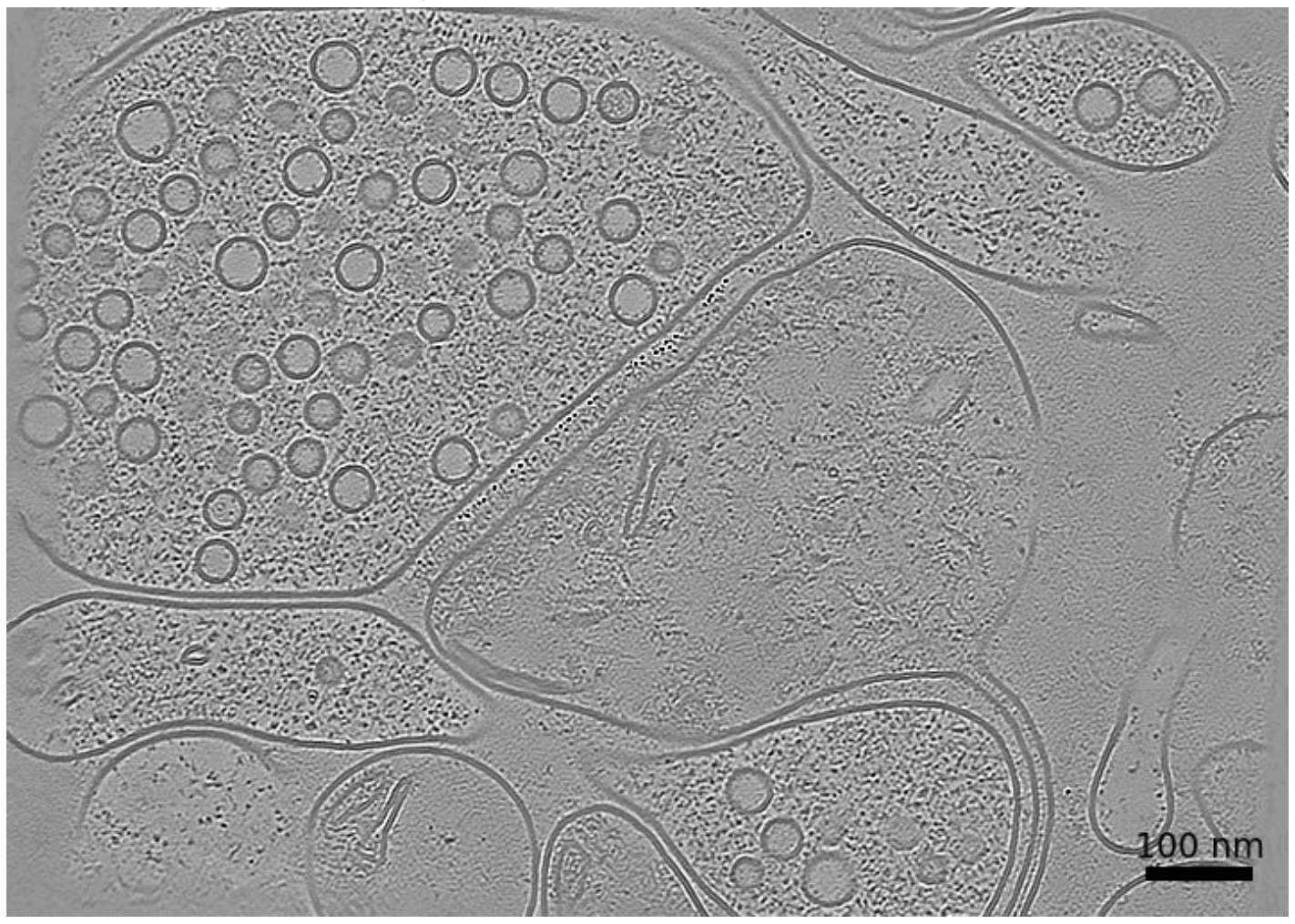

Membrane Segmentation & Cleft Position

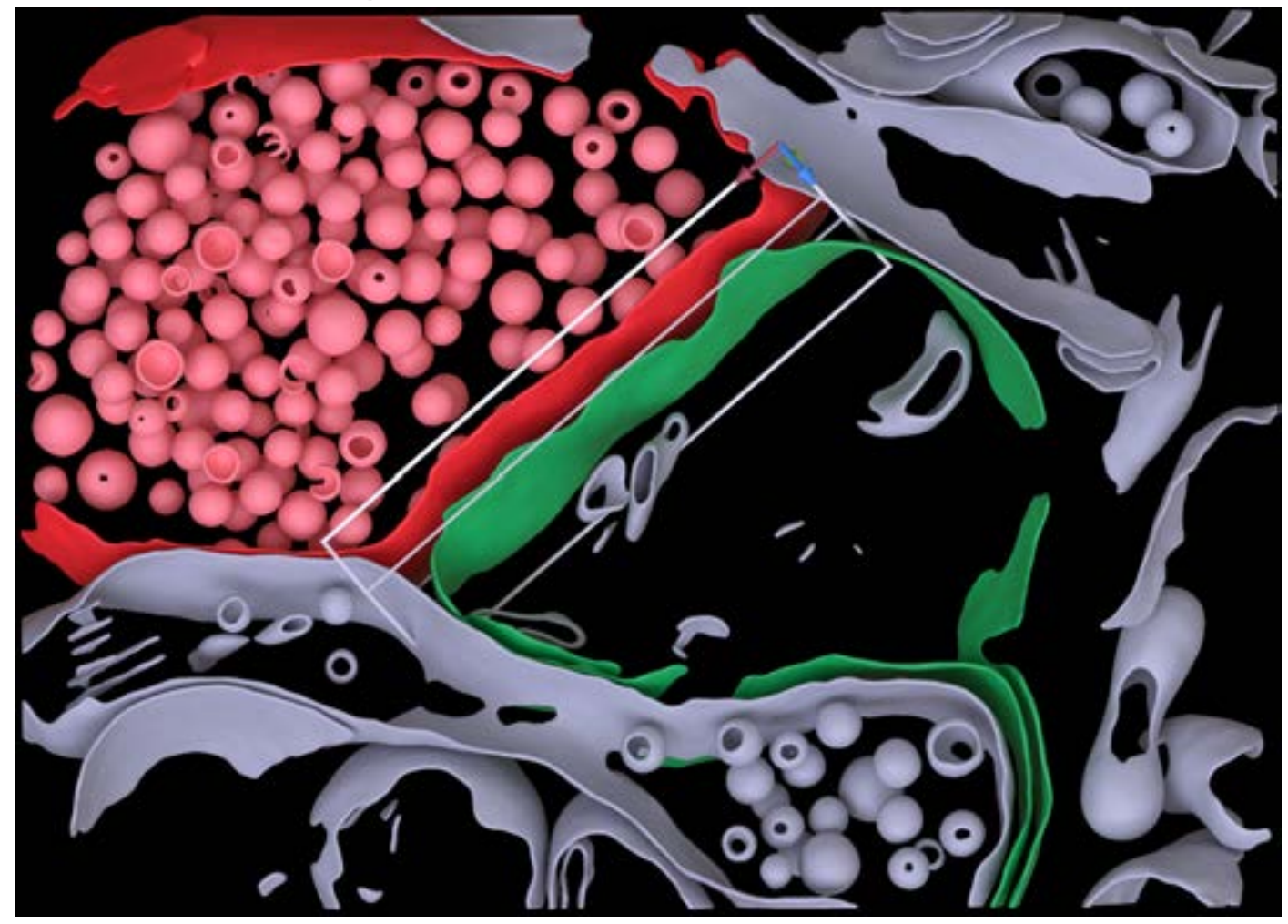

Cleft MIP

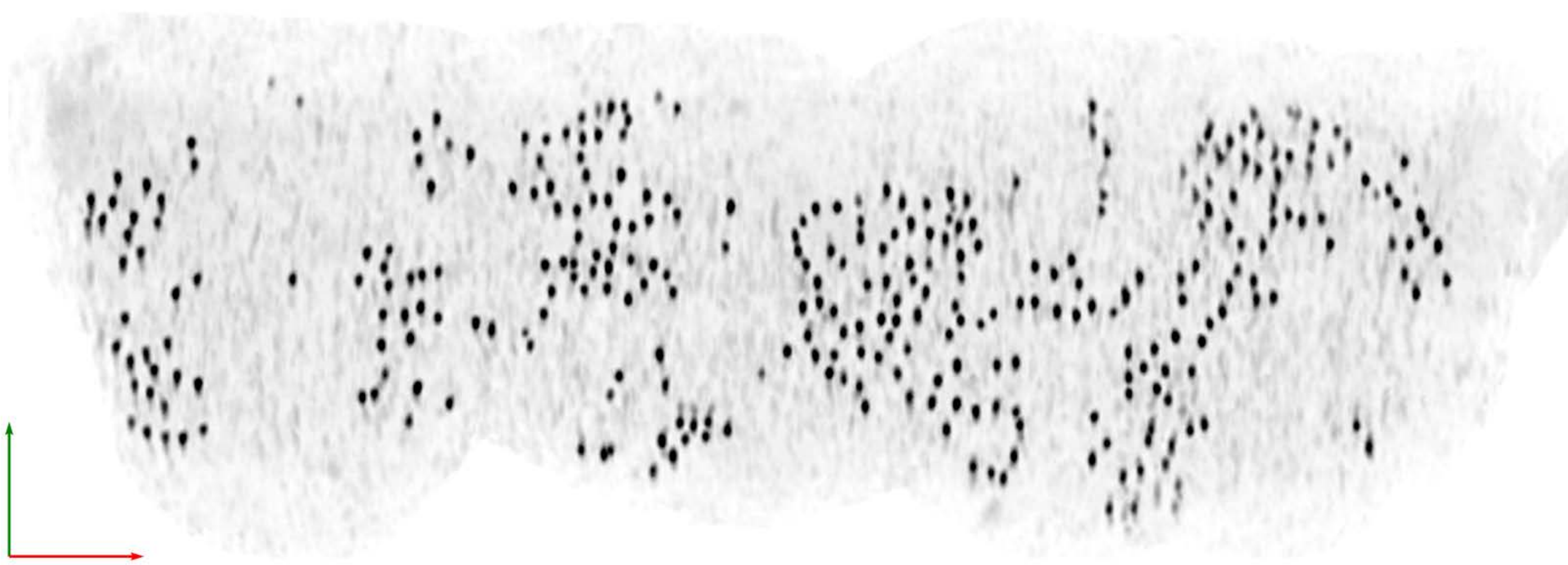

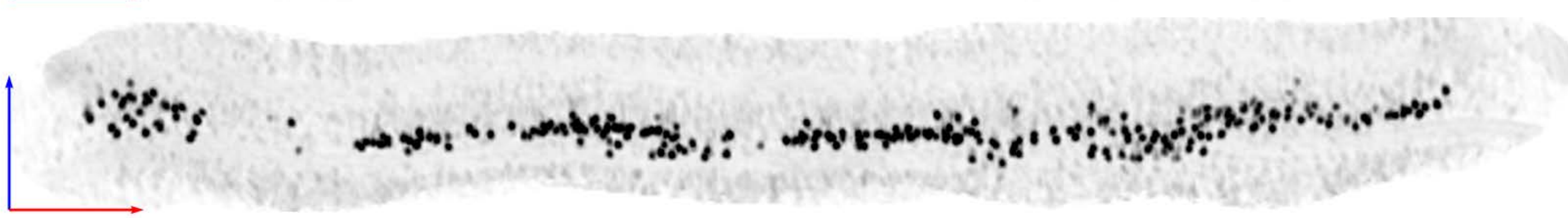

Tomogram ID: 20241030_AMmilled12-1_15 **SET: anti-GluA2 15F1 "C-term"**

Cleft ID: 2 Type: tissue

Denoised Tomogram Slice

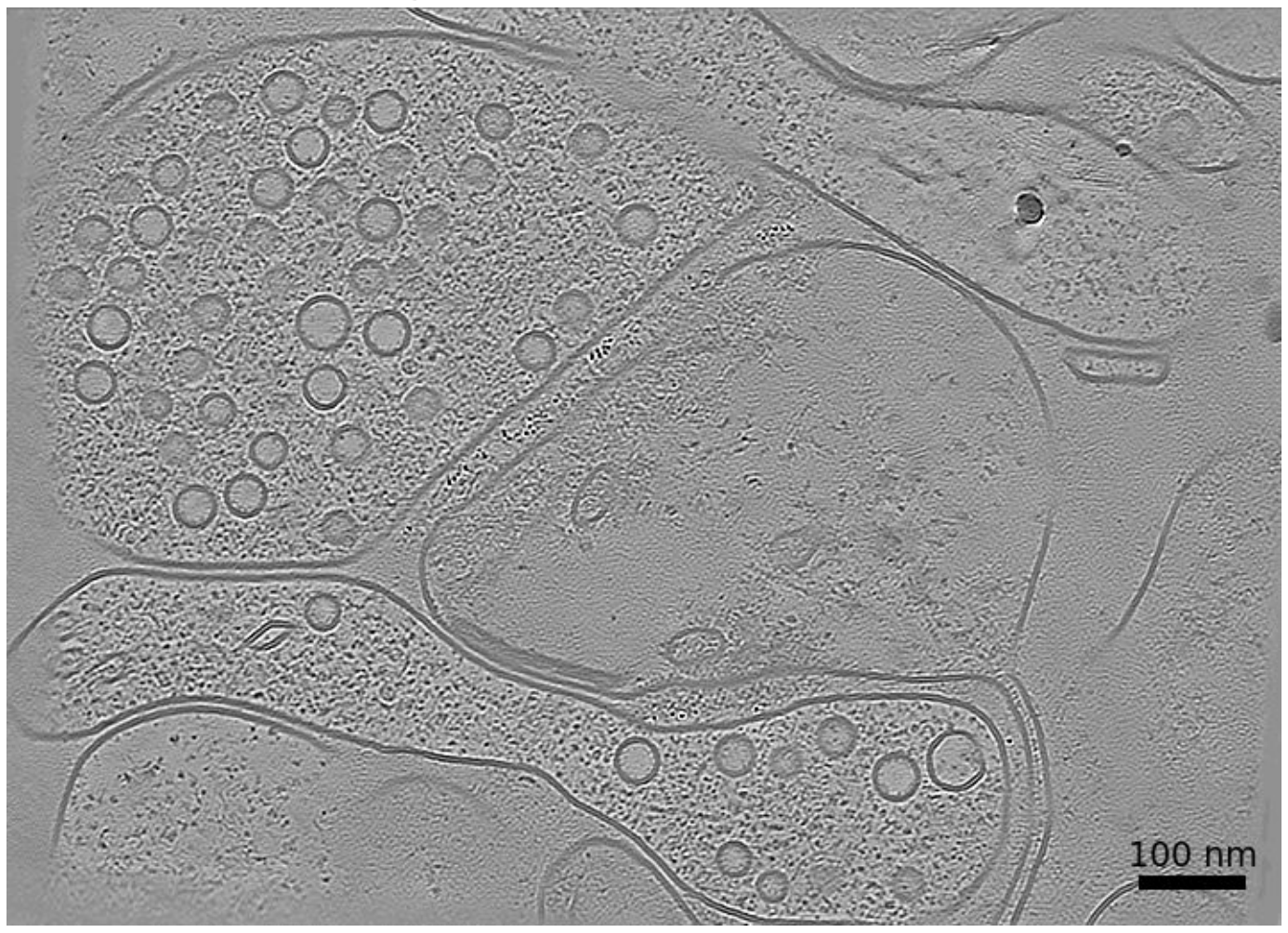

Membrane Segmentation & Cleft Position

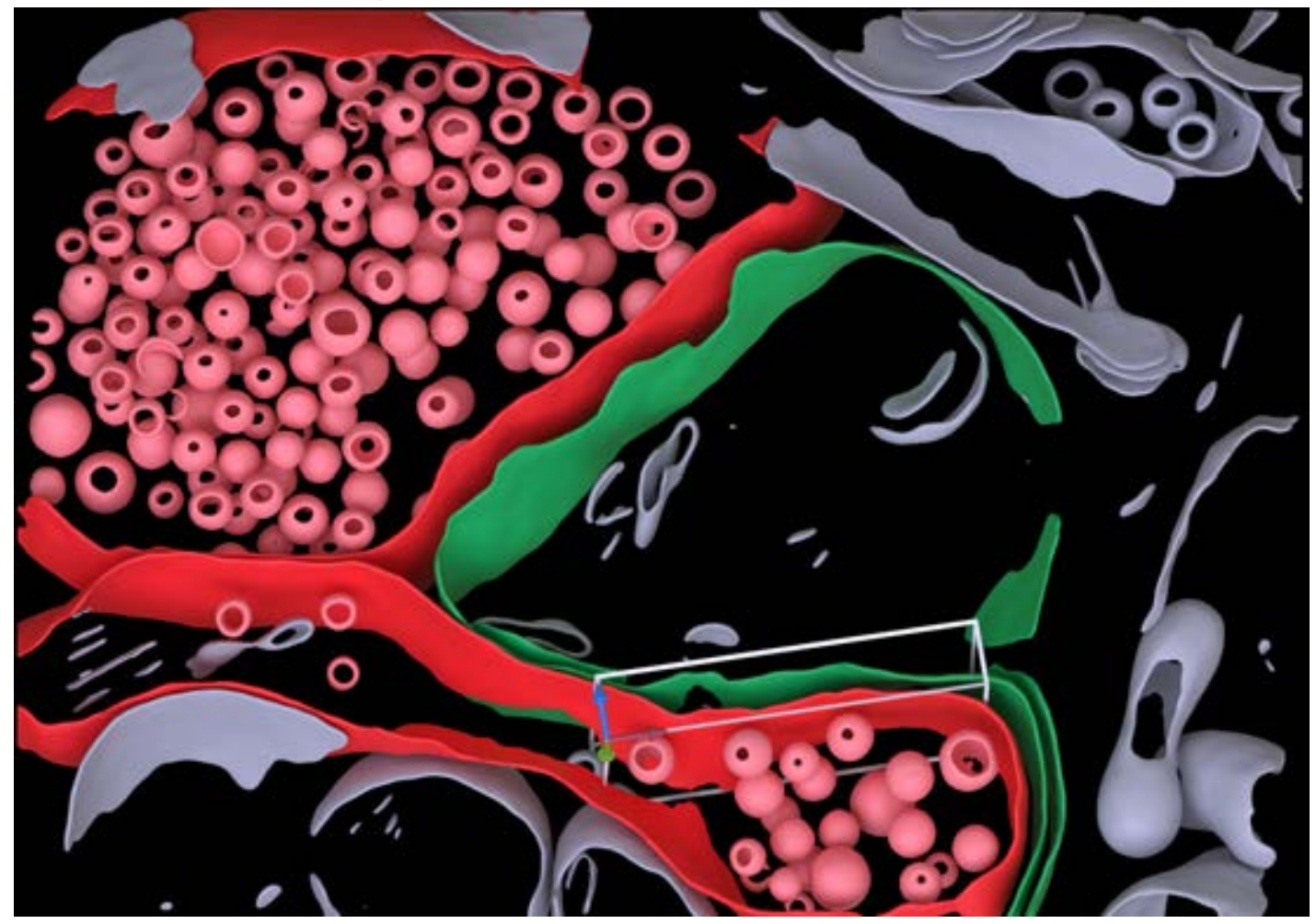

Cleft MIP

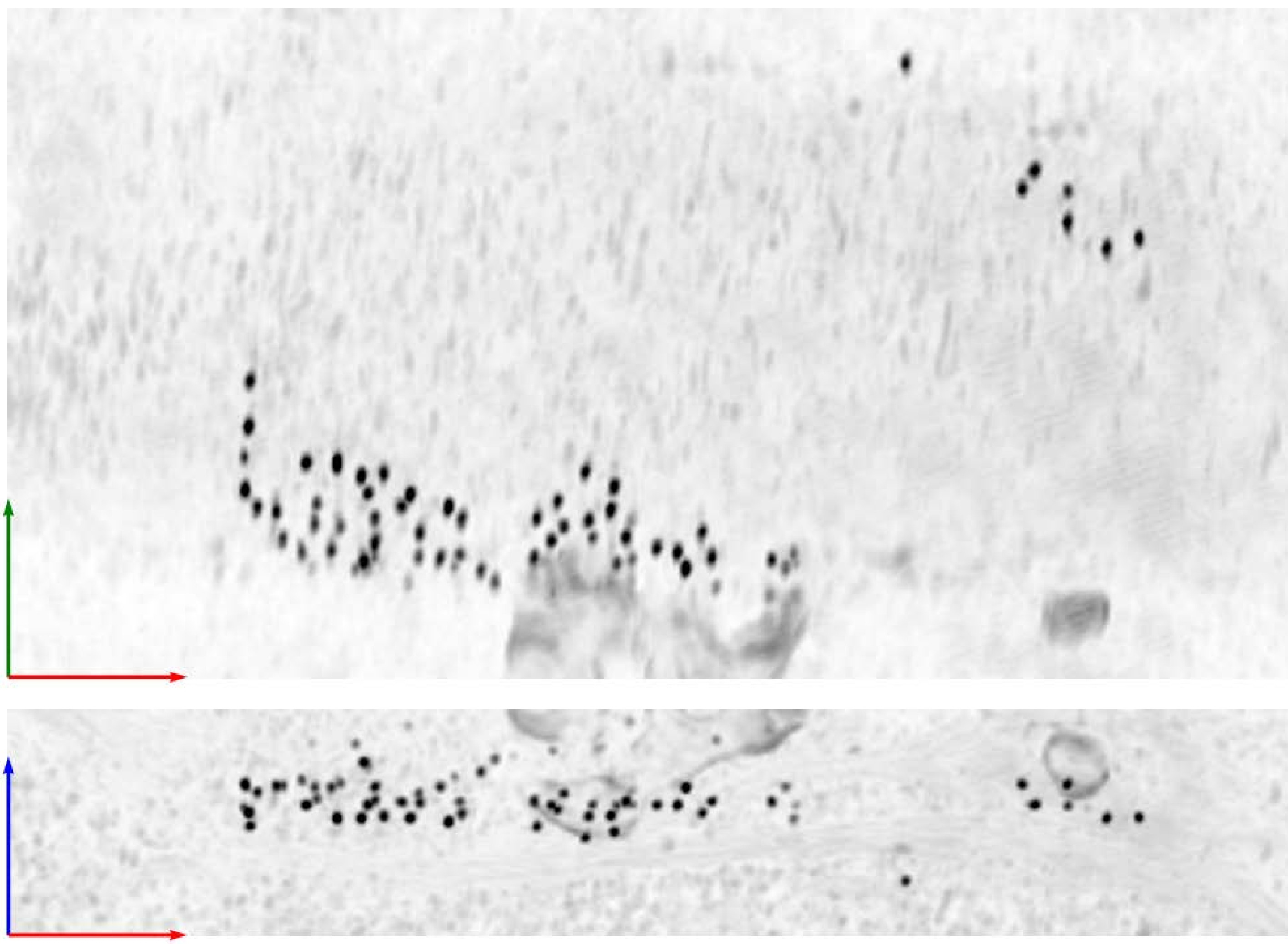

Tomogram ID: 20241030_AMmilled12-2_78 | **SET: anti-GluA2 15F1 "C-term"**

Cleft ID: 0 | Type: tissue

Denoised Tomogram Slice

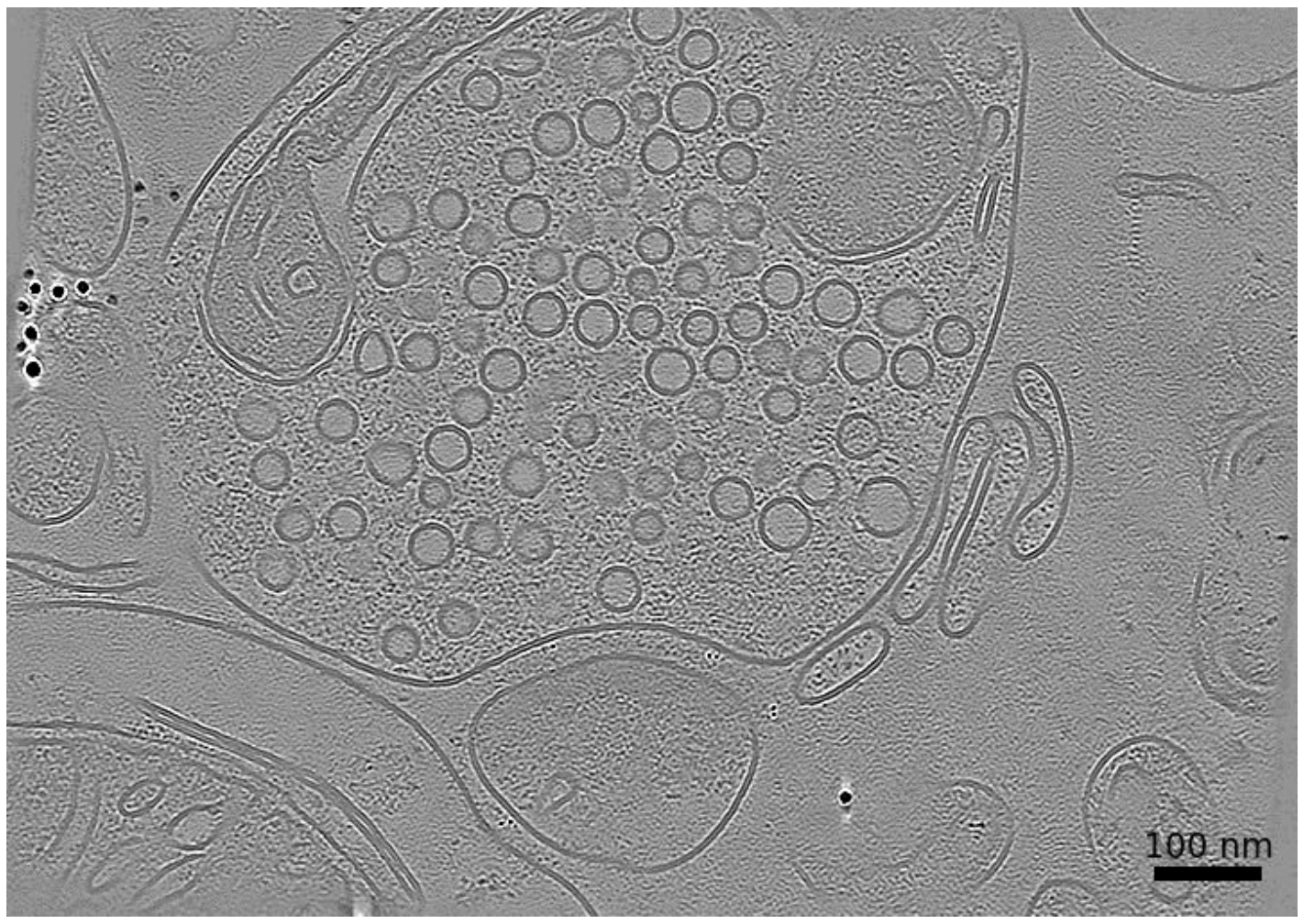

Membrane Segmentation & Cleft Position

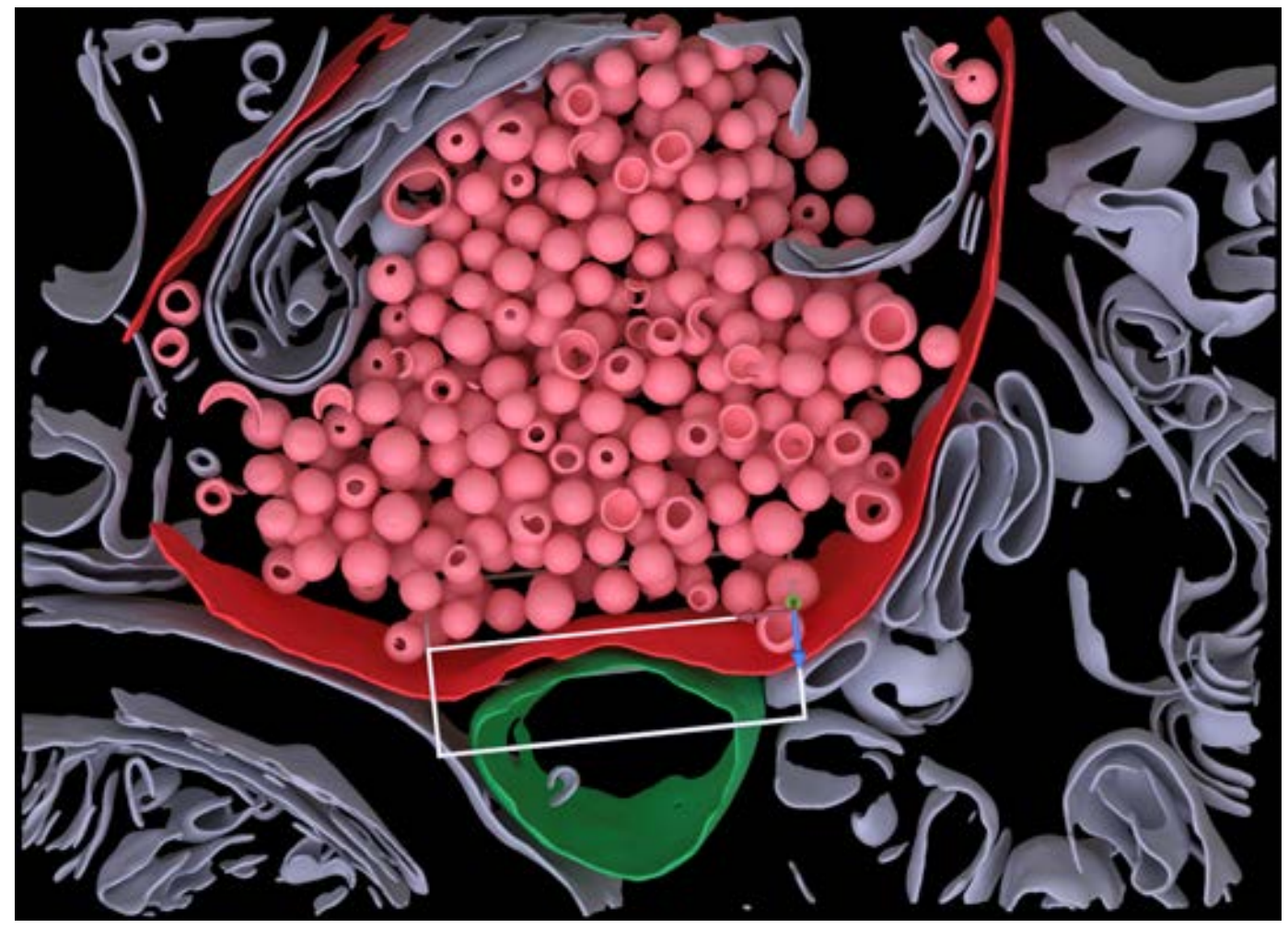

Cleft MIP

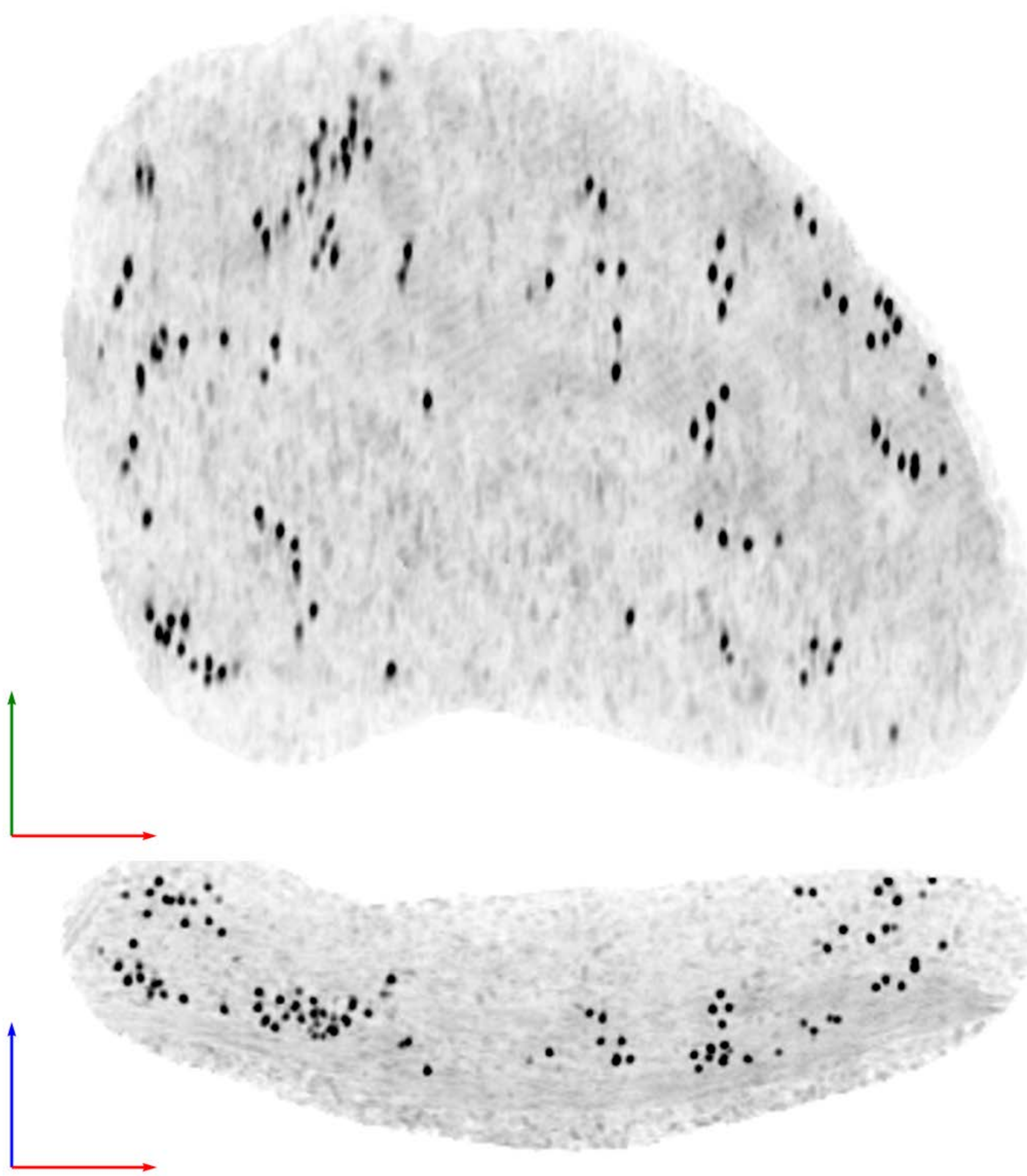

Tomogram ID: AMmilled54-1_Position_61	SET: anti-GluA2 15F1 "H4K-2Cys"
Cleft ID: 0	Type: tissue

Denoised Tomogram Slice

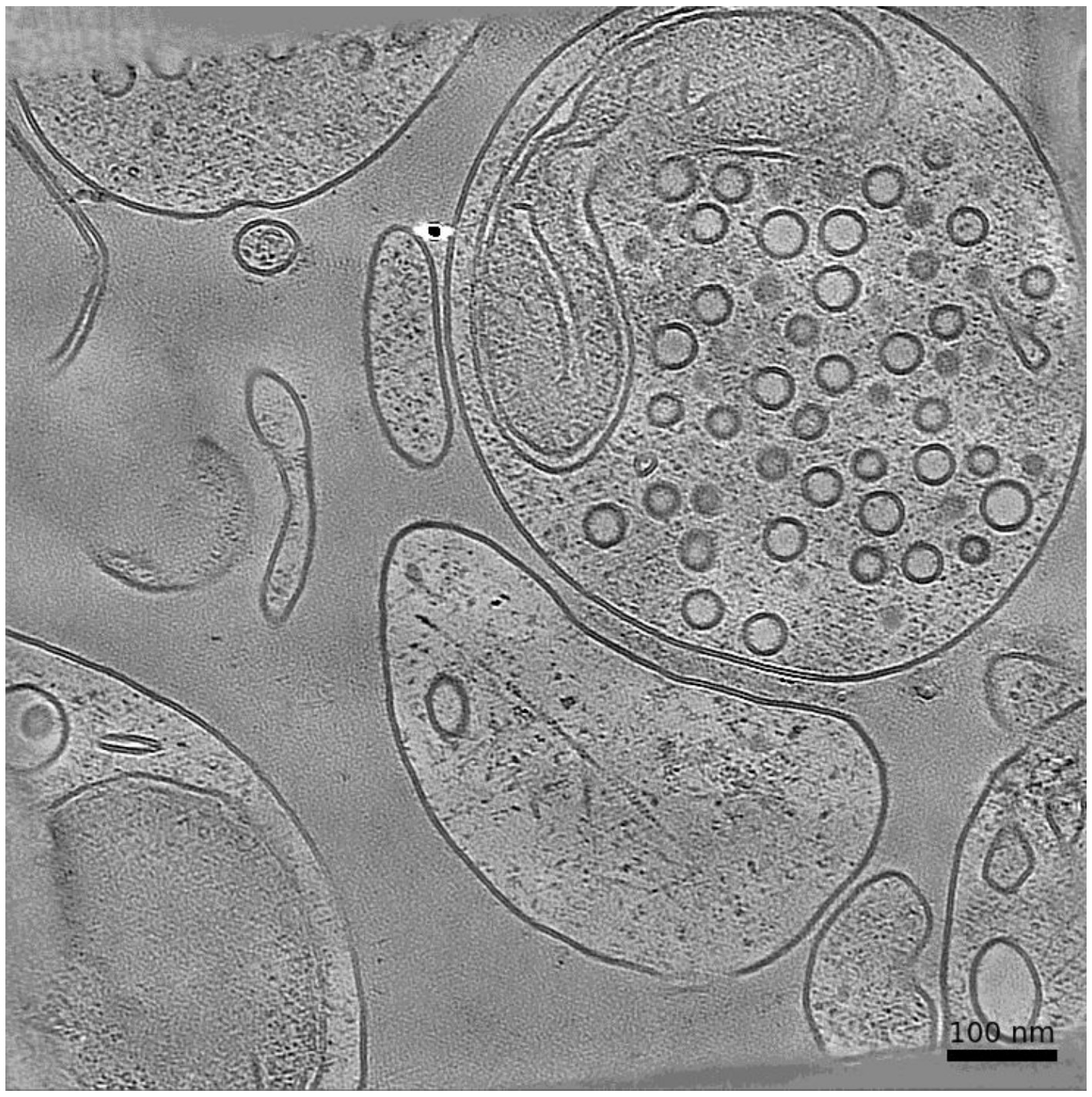

Membrane Segmentation & Cleft Position

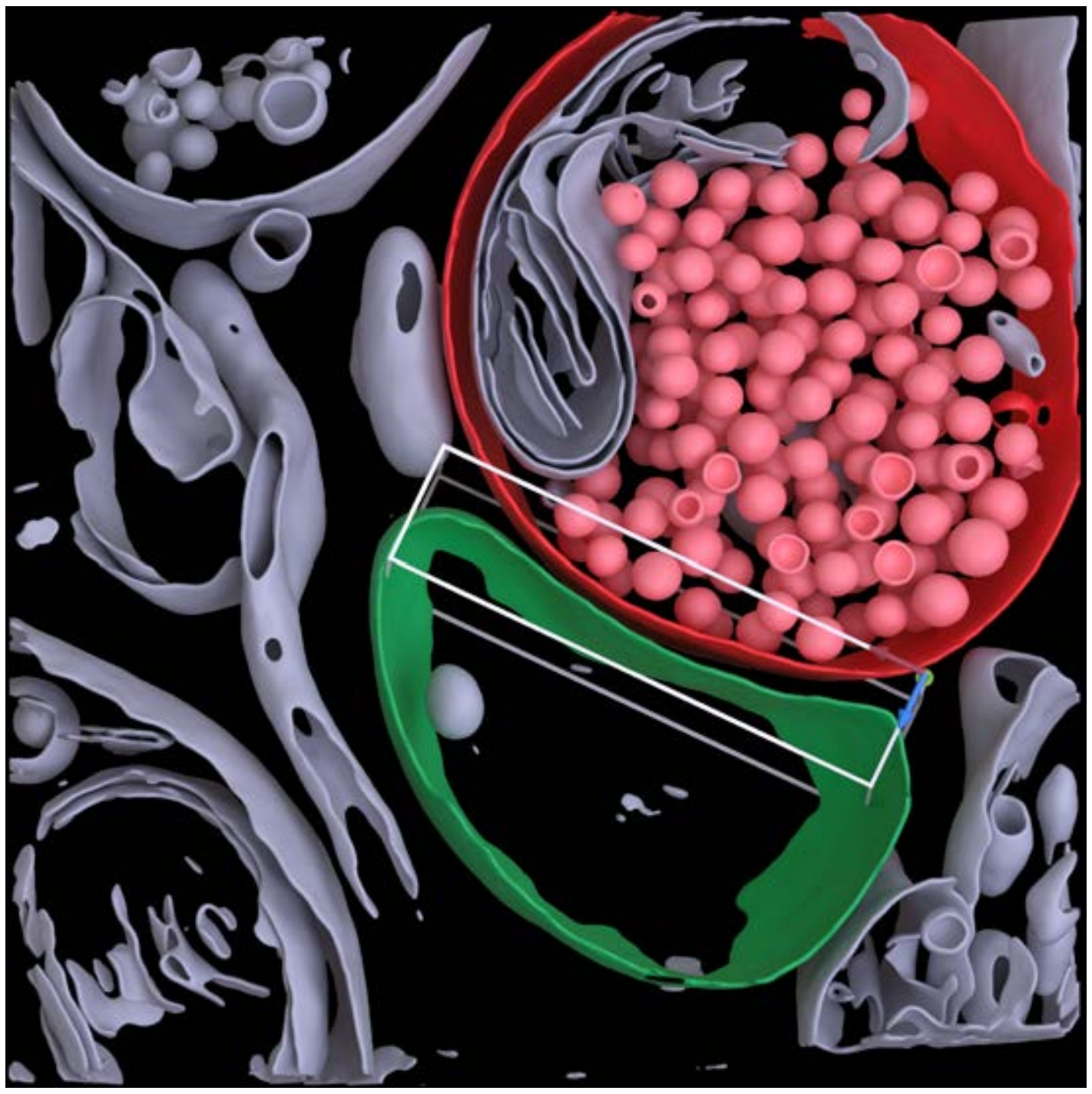

Cleft MIP

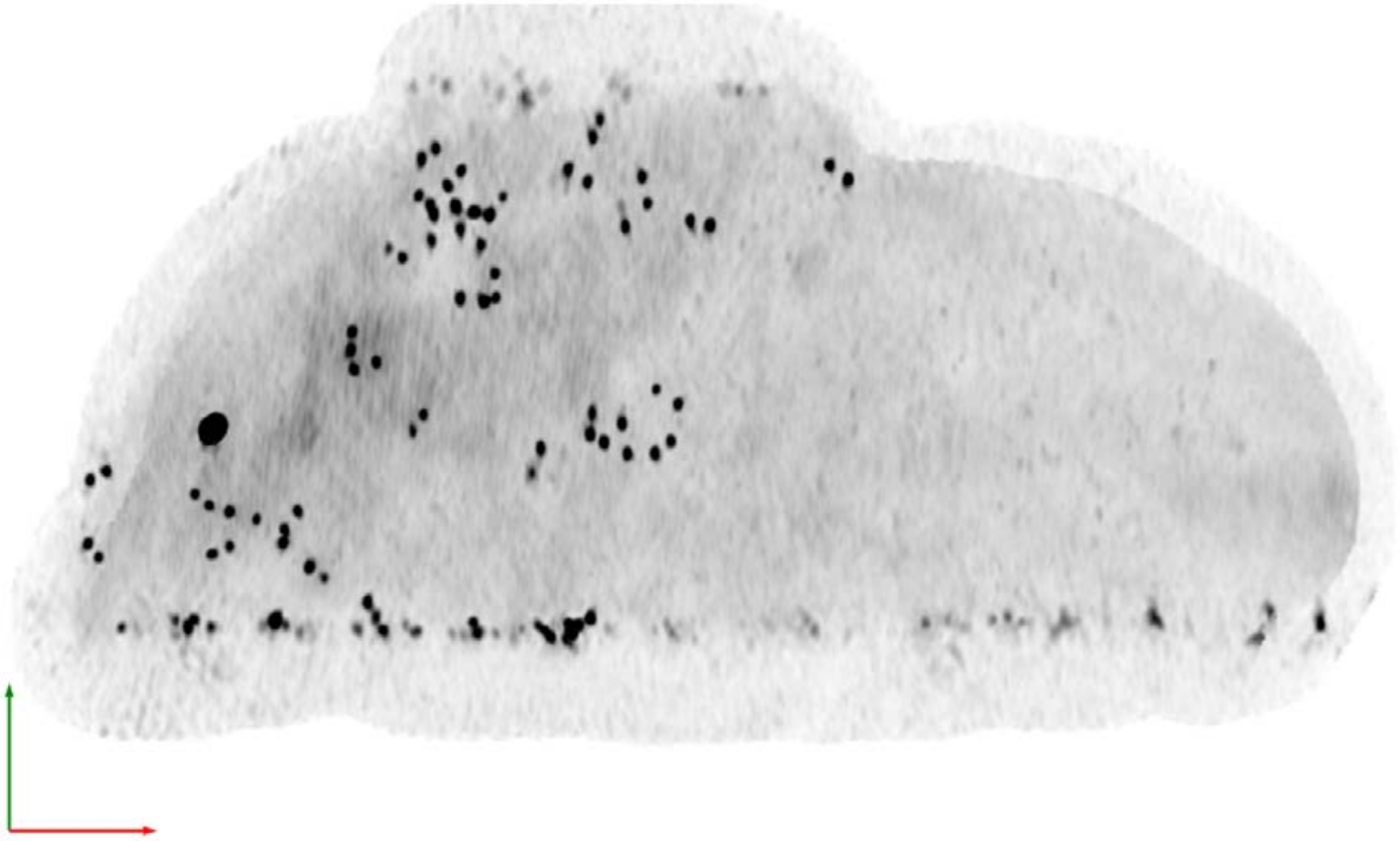

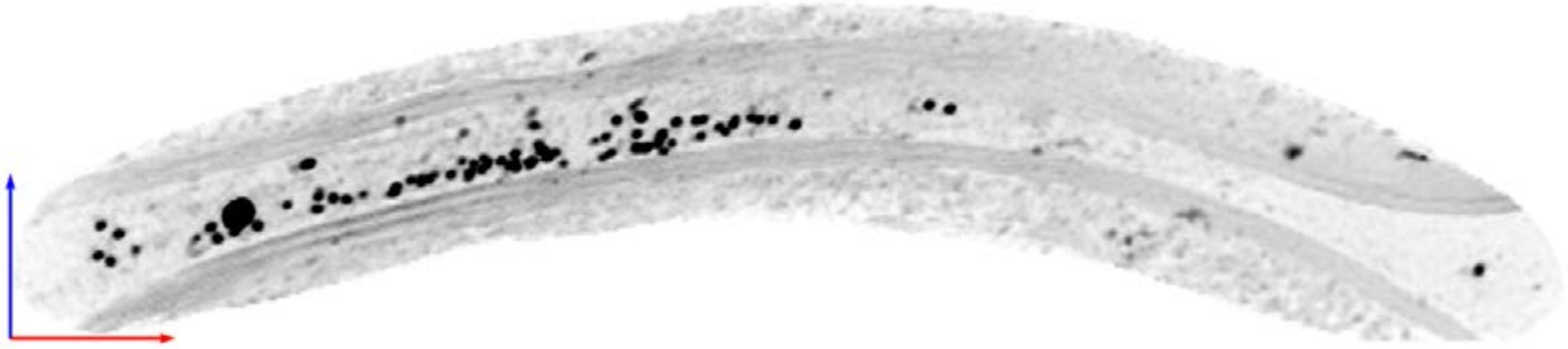

Tomogram ID: AMmilled55-1_Position_10_3	SET: anti-GluA2 15F1 "H4K-2Cys"
Cleft ID: 0	Type: tissue

Denoised Tomogram Slice

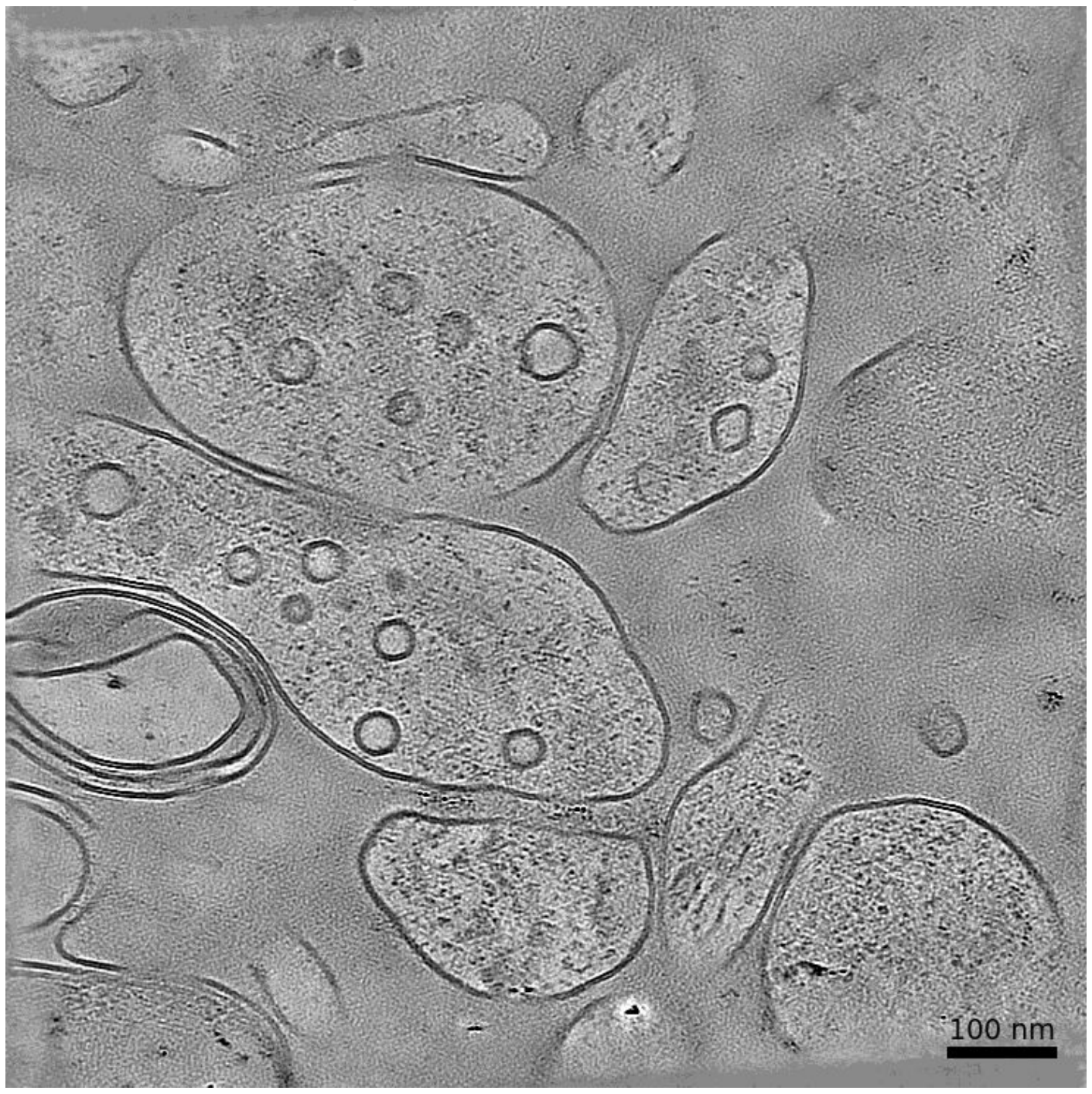

Membrane Segmentation & Cleft Position

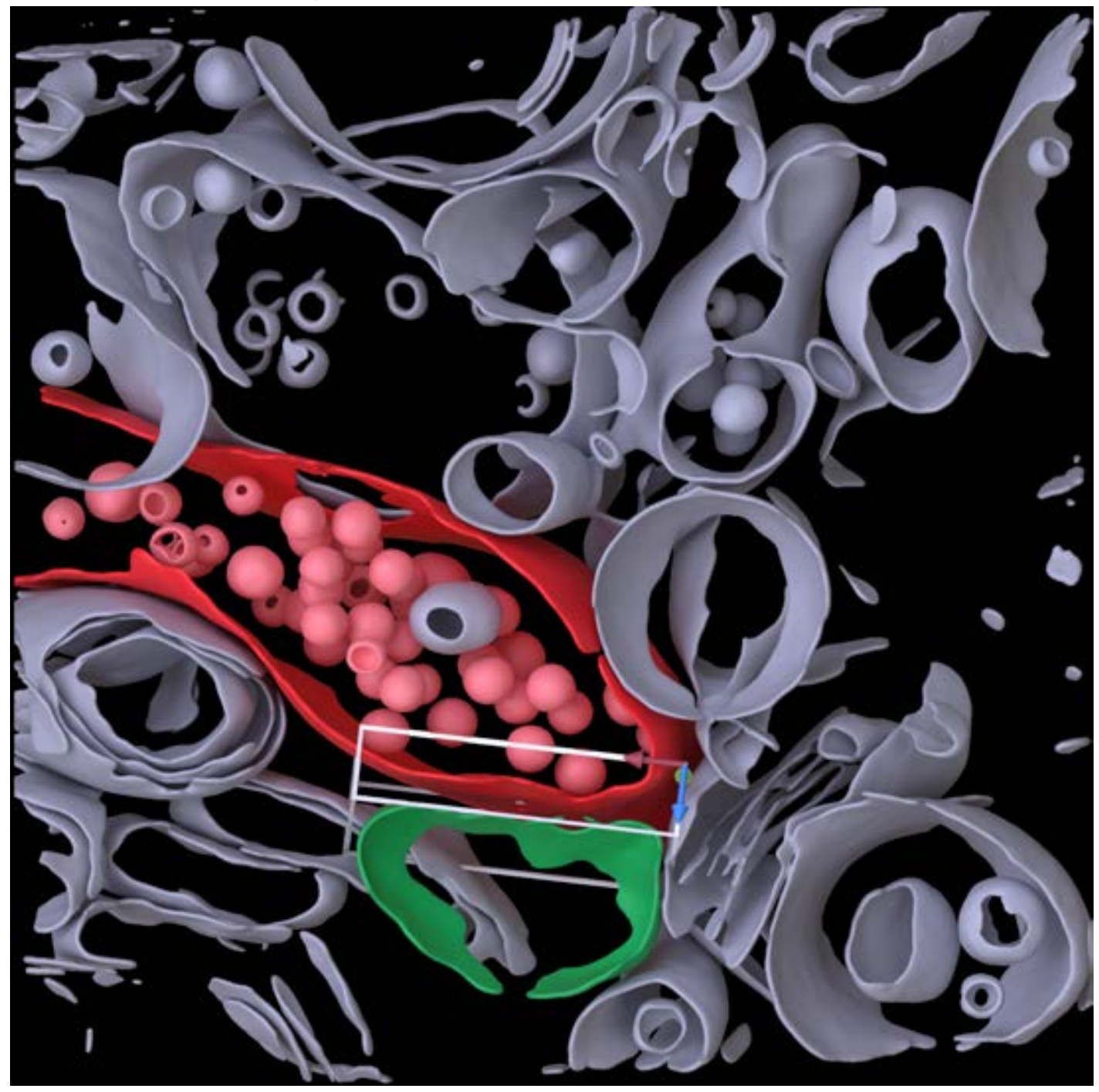

Cleft MIP

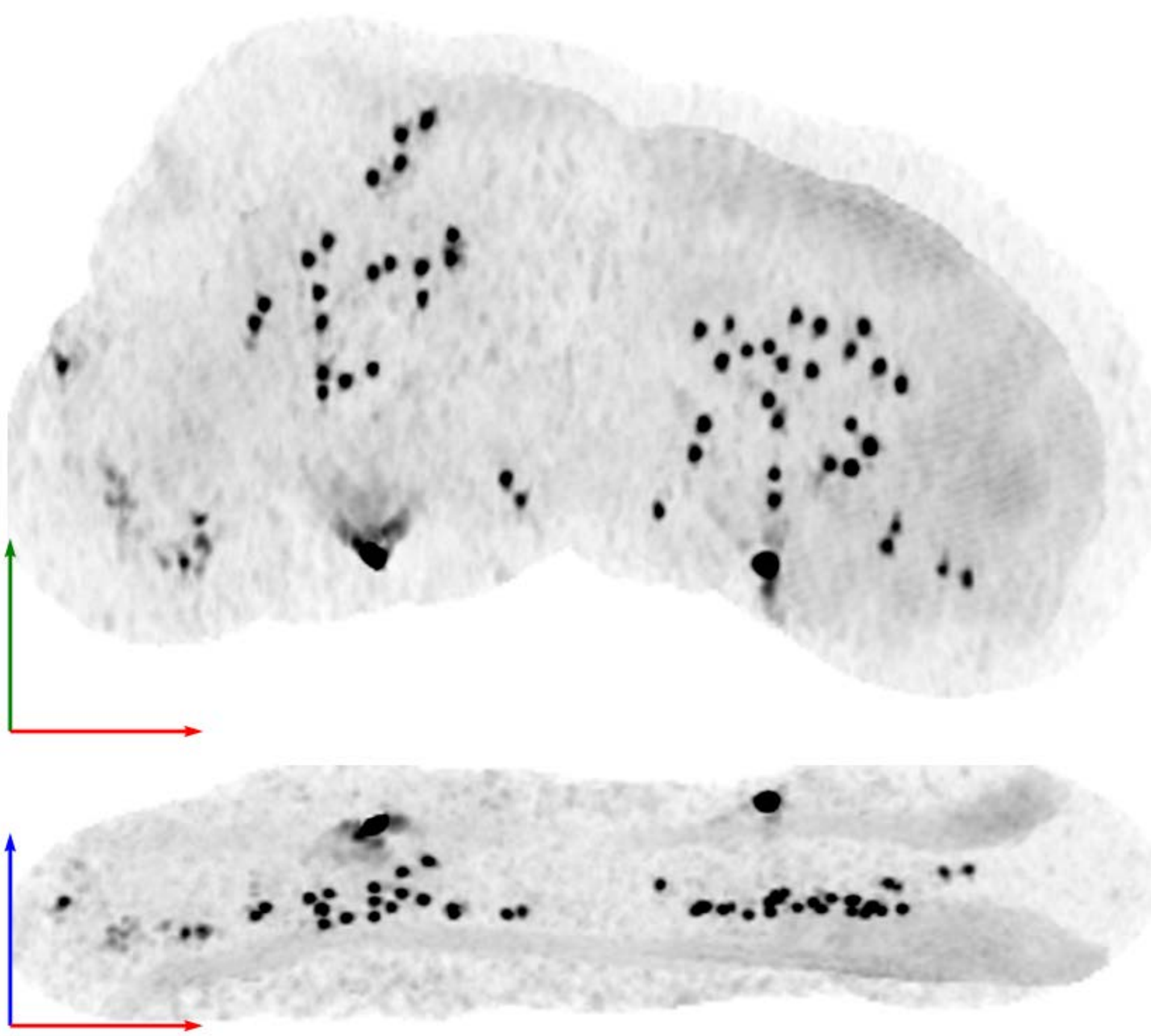

Tomogram ID: AMmilled55-1_Position_112_2	SET: anti-GluA2 15F1 "H4K-2Cys"
Cleft ID: 0	Type: tissue

Denoised Tomogram Slice

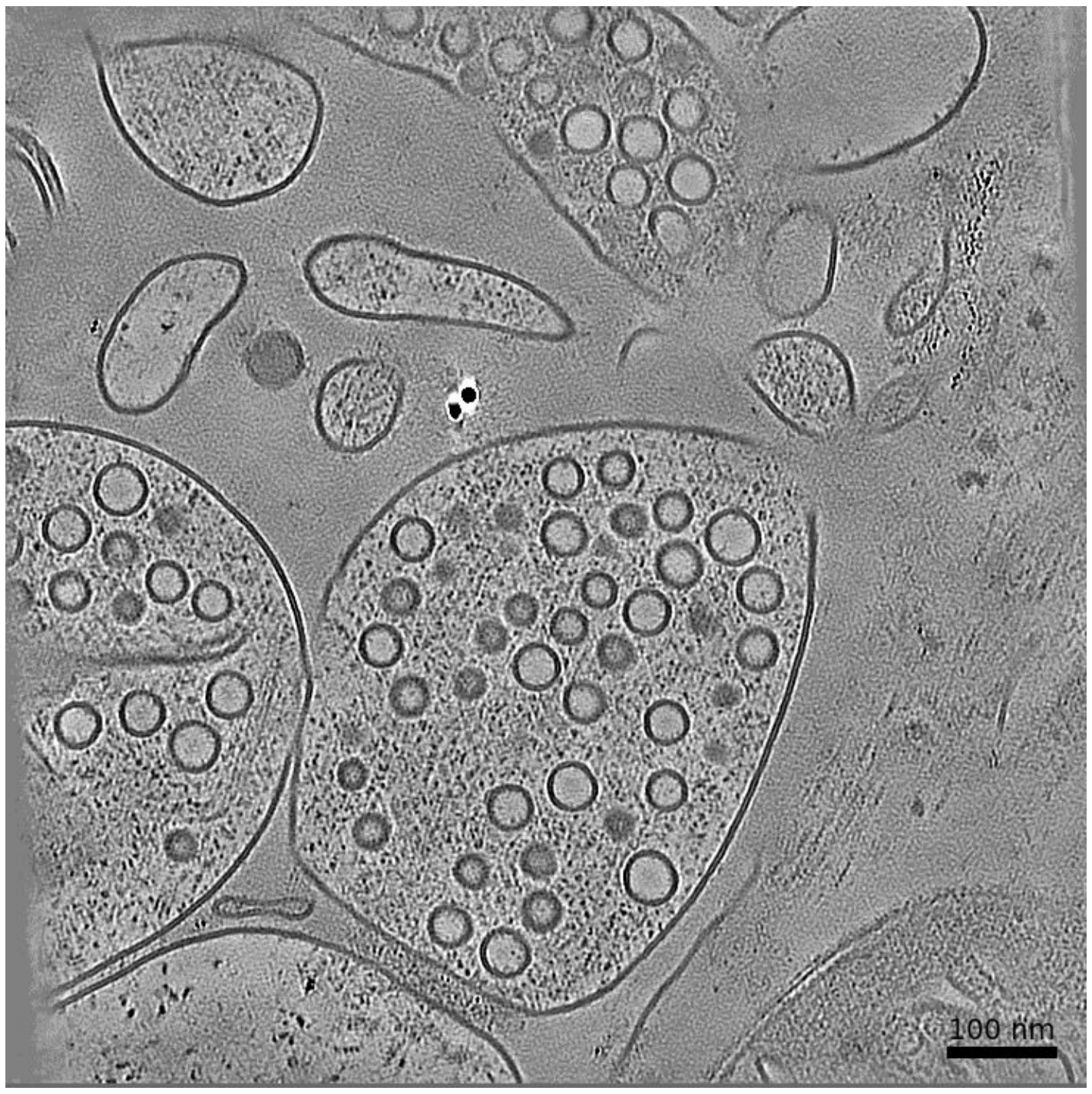

Membrane Segmentation & Cleft Position

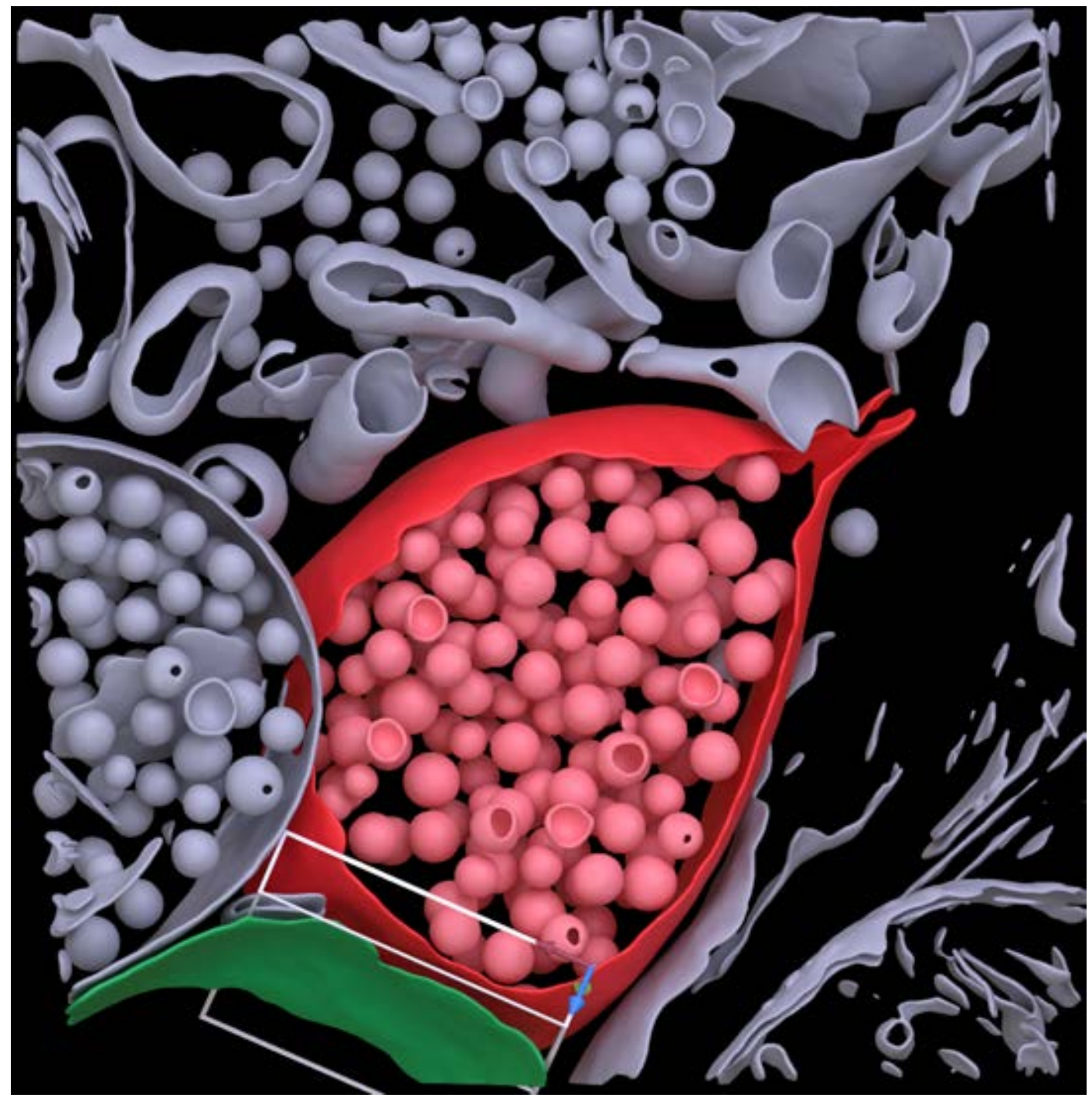

Cleft MIP

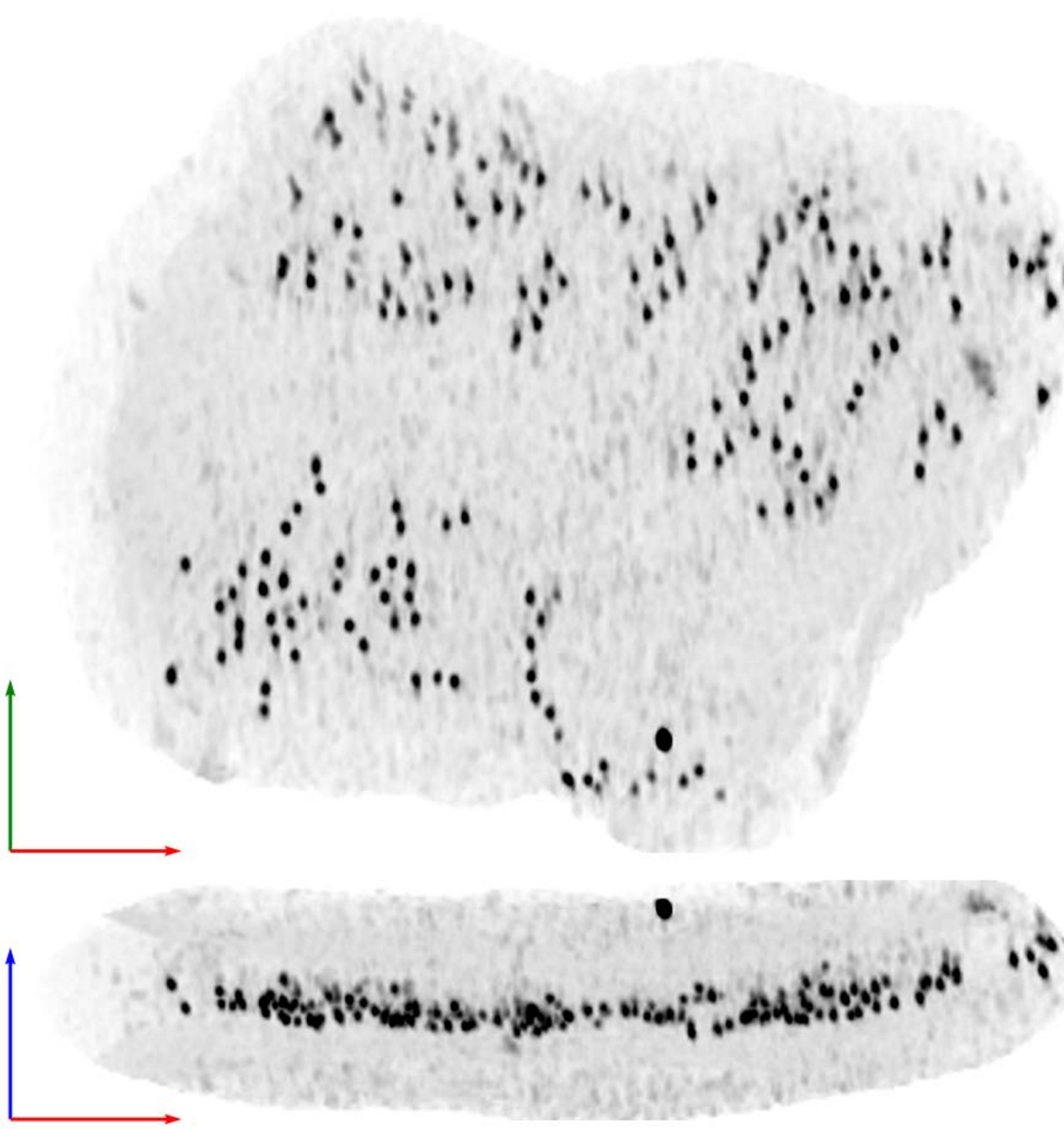

Tomogram ID: AMmilled55-1_Position_34 **SET: anti-GluA2 15F1 "H4K-2Cys"**

Cleft ID: 0 Type: tissue

Denoised Tomogram Slice

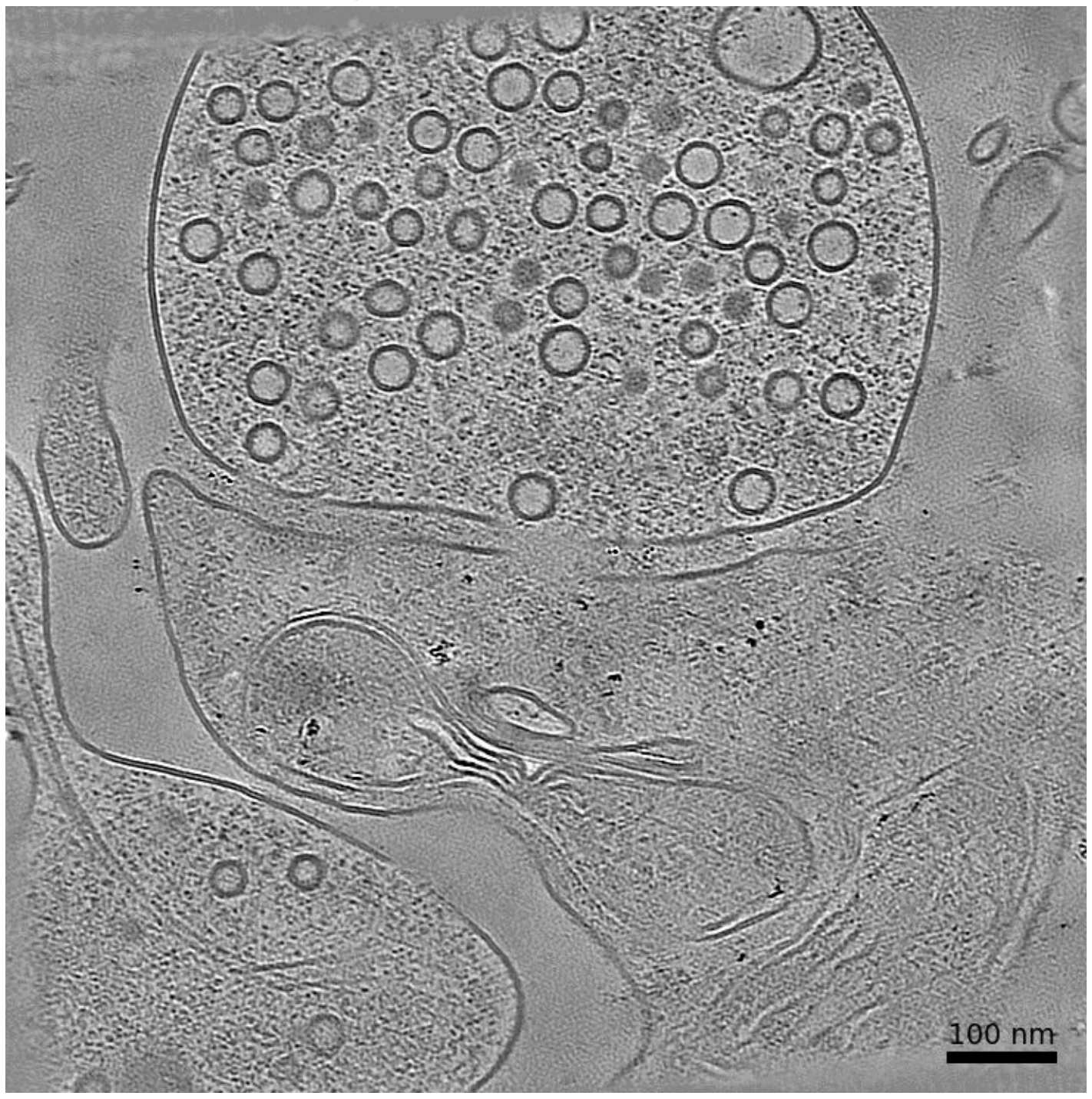

Membrane Segmentation & Cleft Position

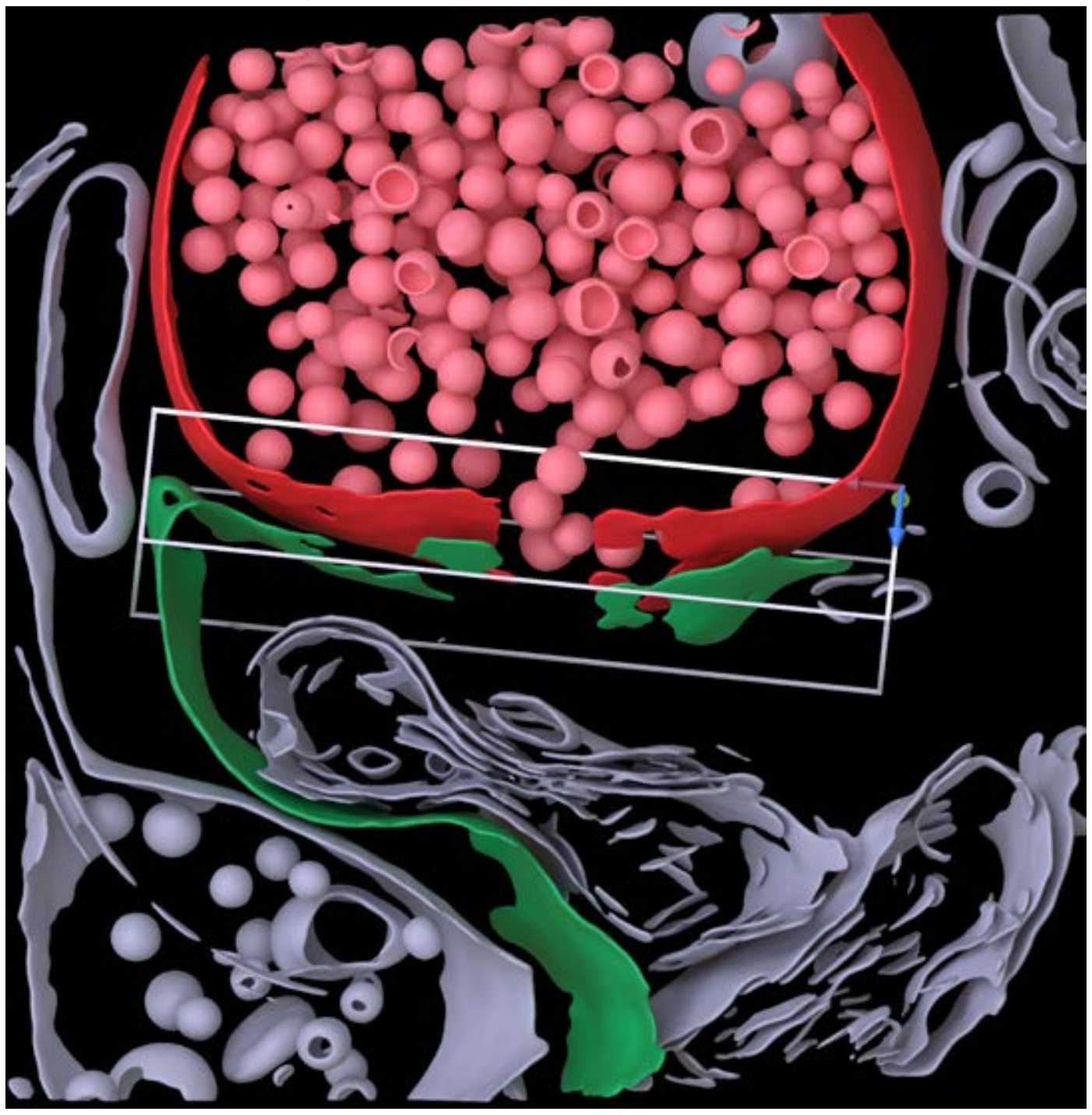

Cleft MIP

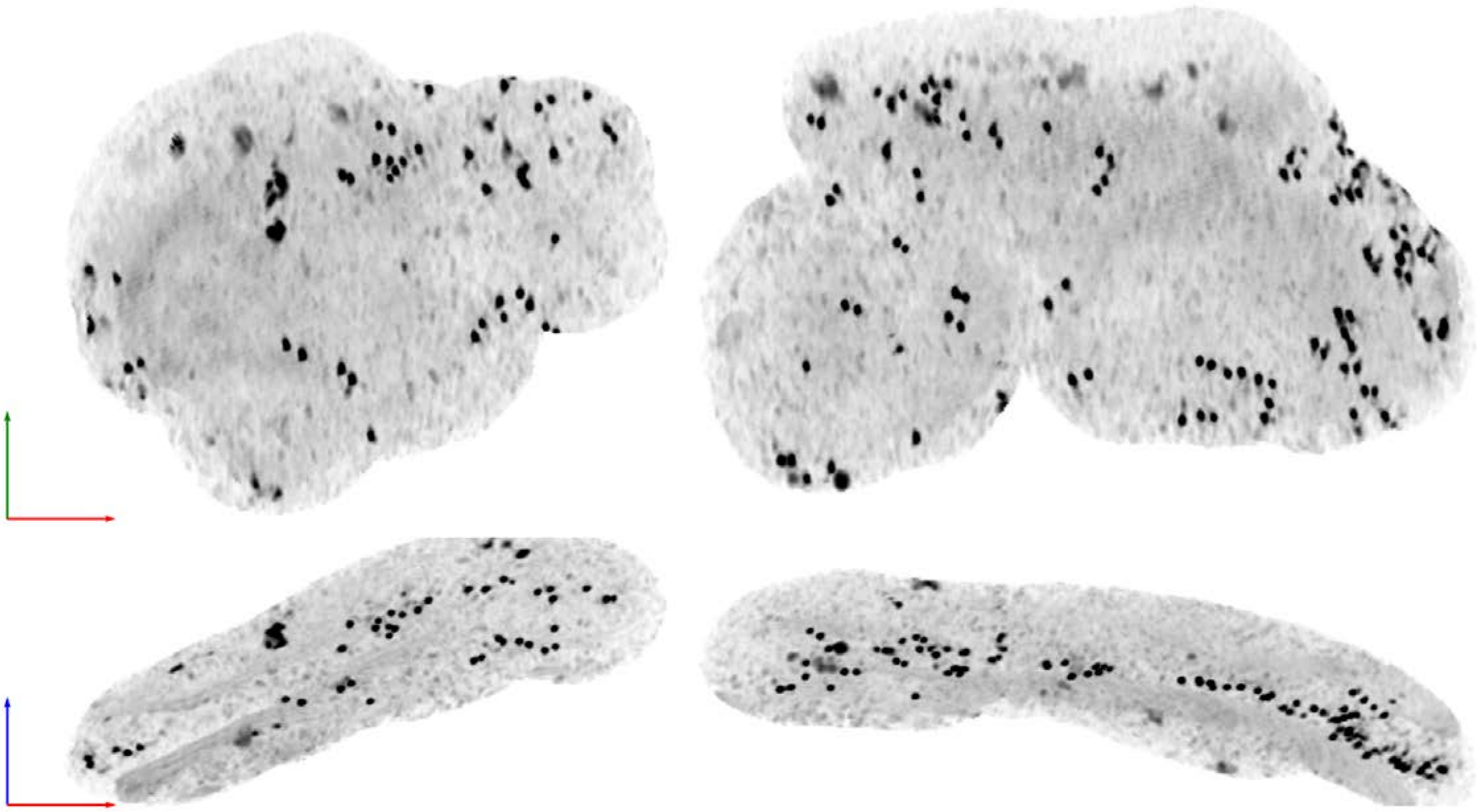

Tomogram ID: AMmilled55-1_Position_35_2	SET: anti-GluA2 15F1 "H4K-2Cys"
Cleft ID: 0	Type: tissue

Denoised Tomogram Slice

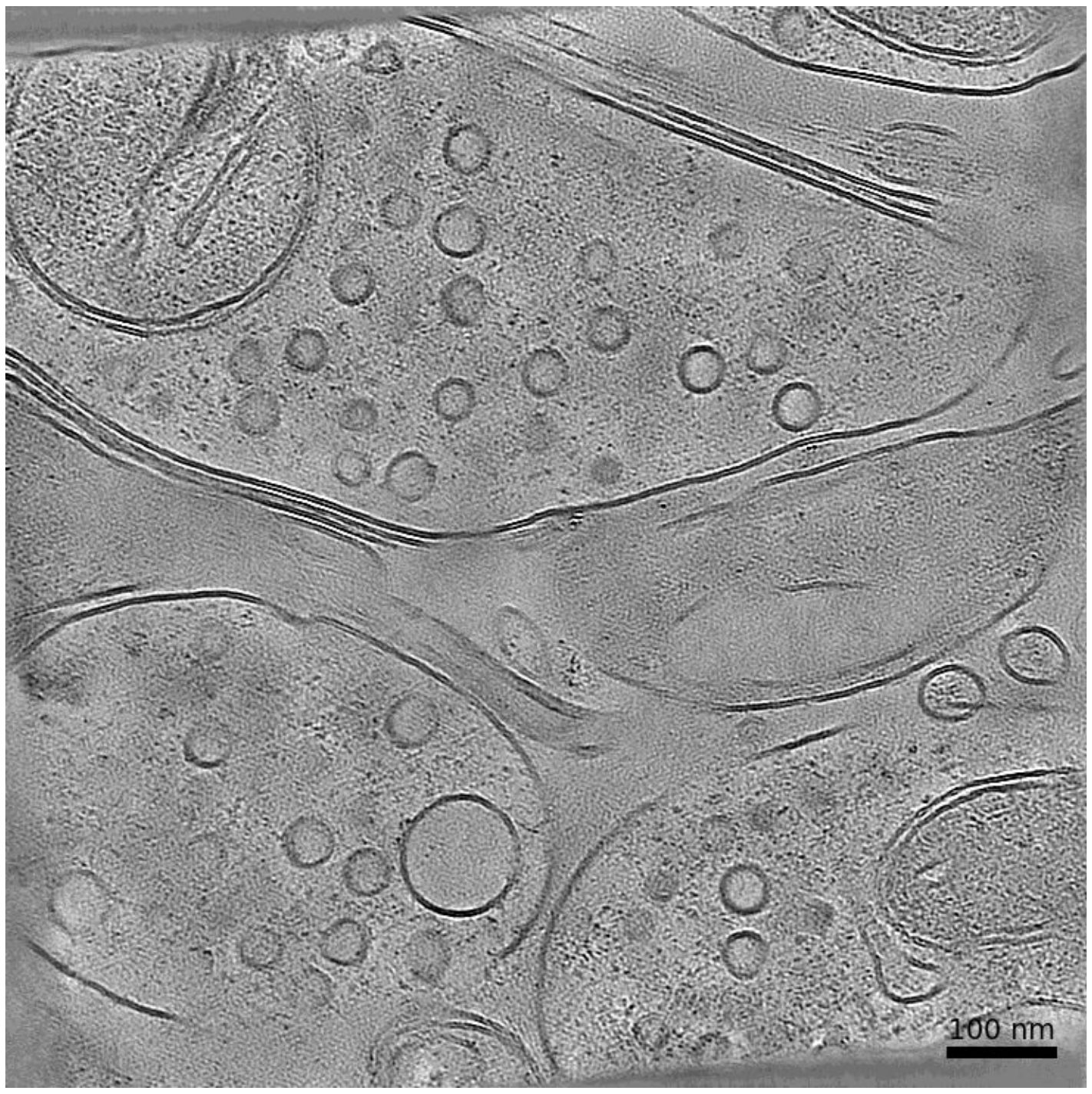

Membrane Segmentation & Cleft Position

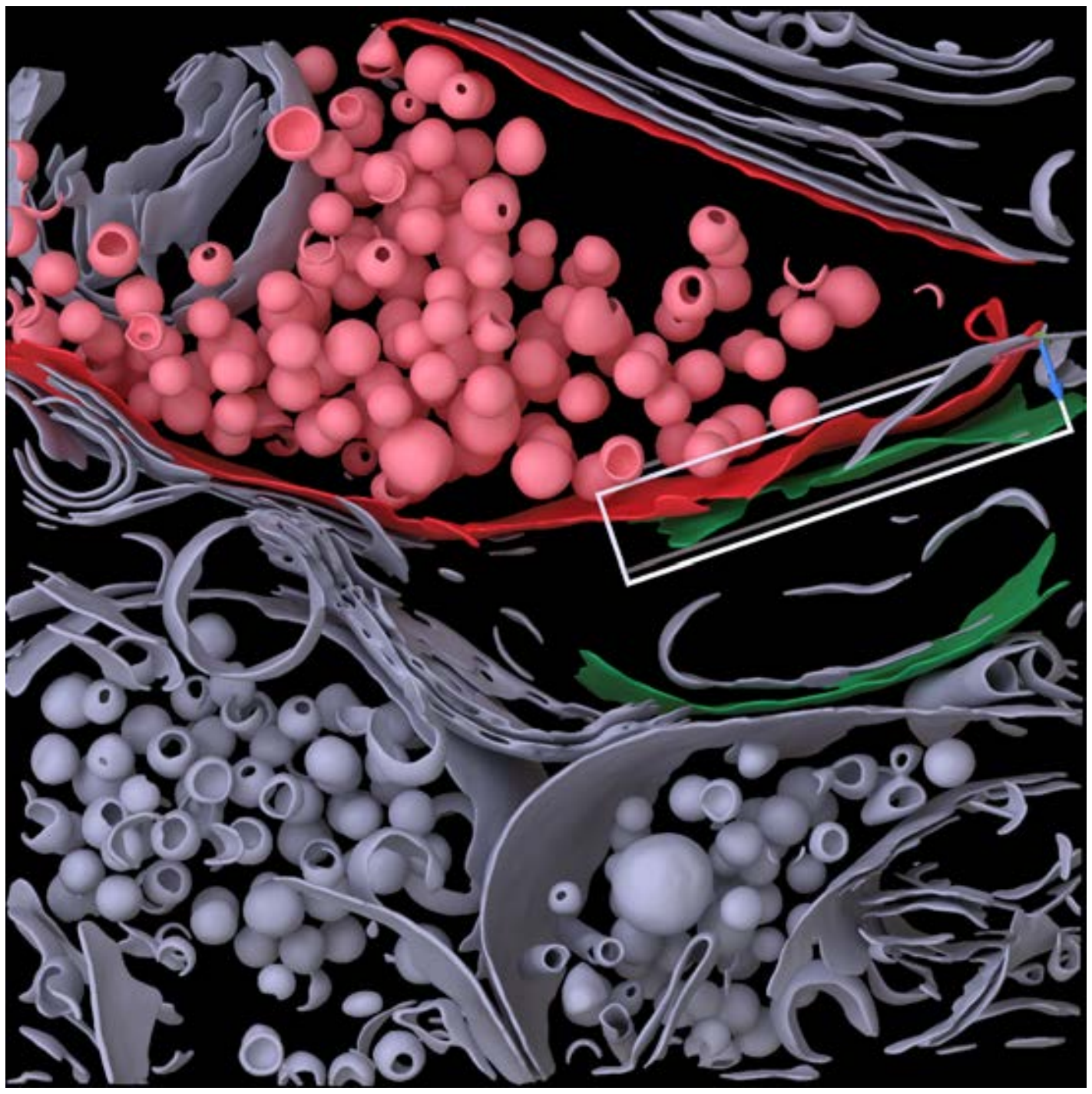

Cleft MIP

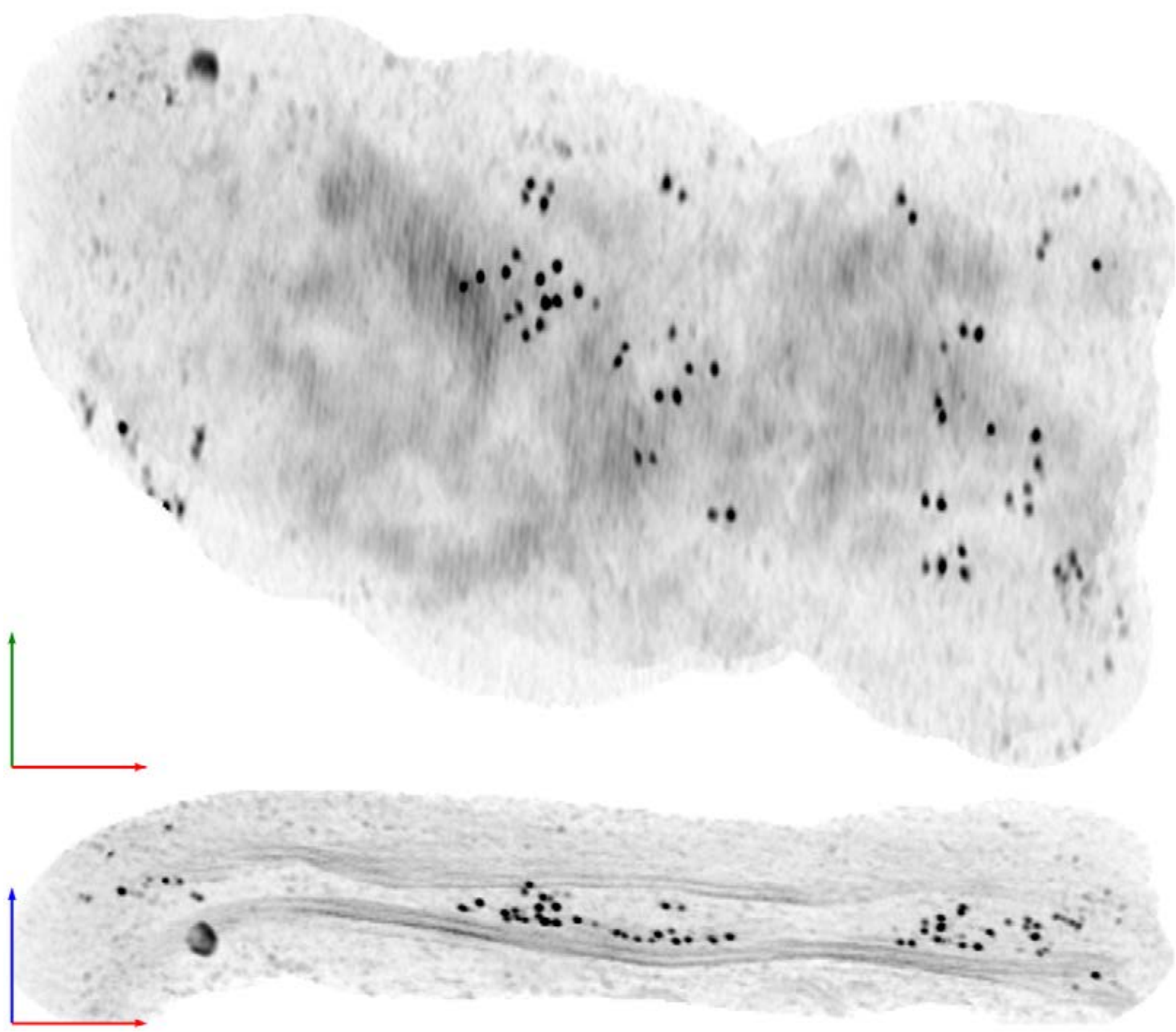

Tomogram ID: AMmilled55-1_Position_43 **SET: anti-GluA2 15F1 "H4K-2Cys"**

Cleft ID: 0 Type: tissue

Denoised Tomogram Slice

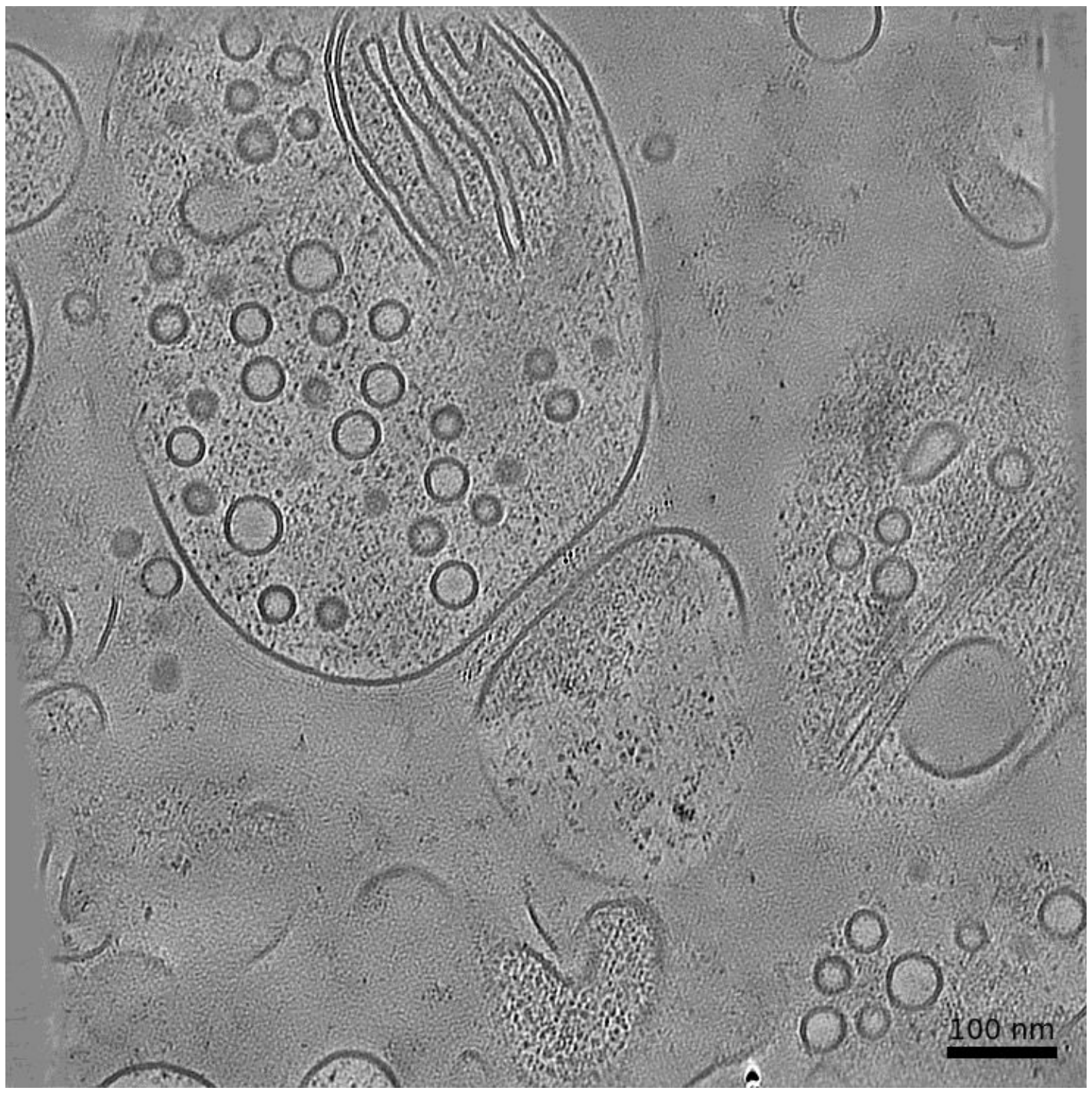

Membrane Segmentation & Cleft Position

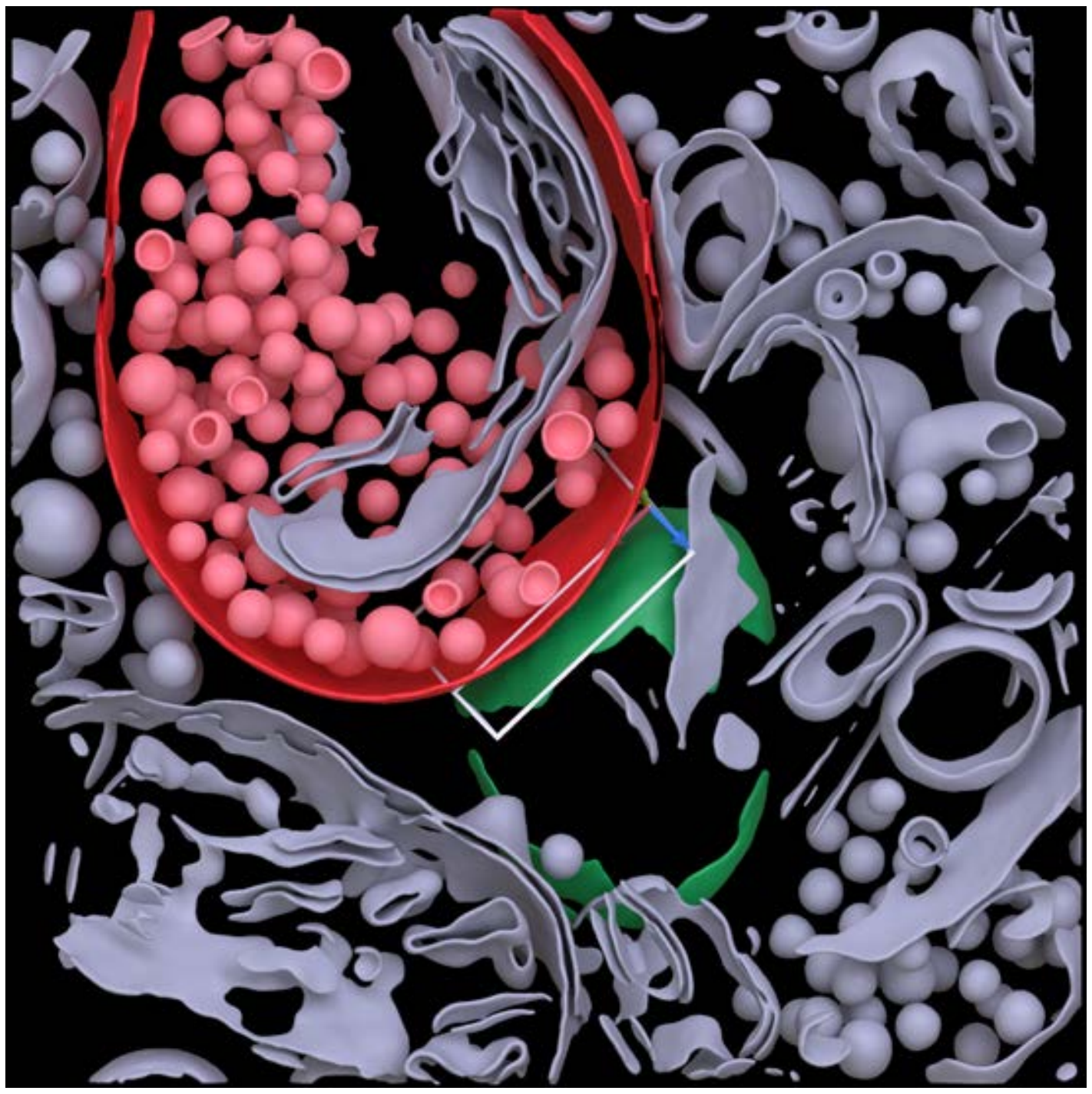

Cleft MIP

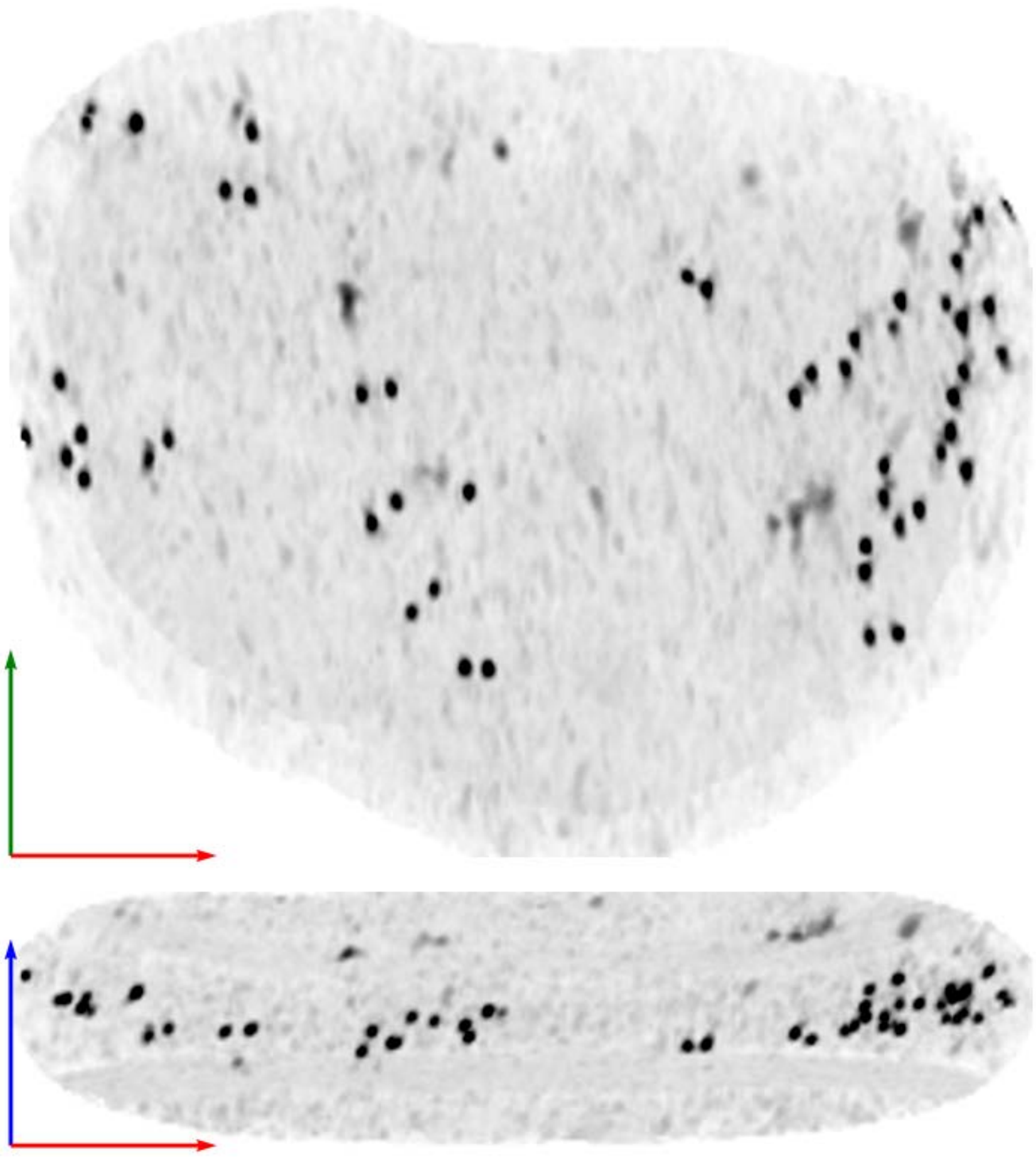

Tomogram ID: AMmilled55-1_Position_47 | **SET: anti-GluA2 15F1 "H4K-2Cys"**

Cleft ID: 0 | Type: tissue

Denoised Tomogram Slice

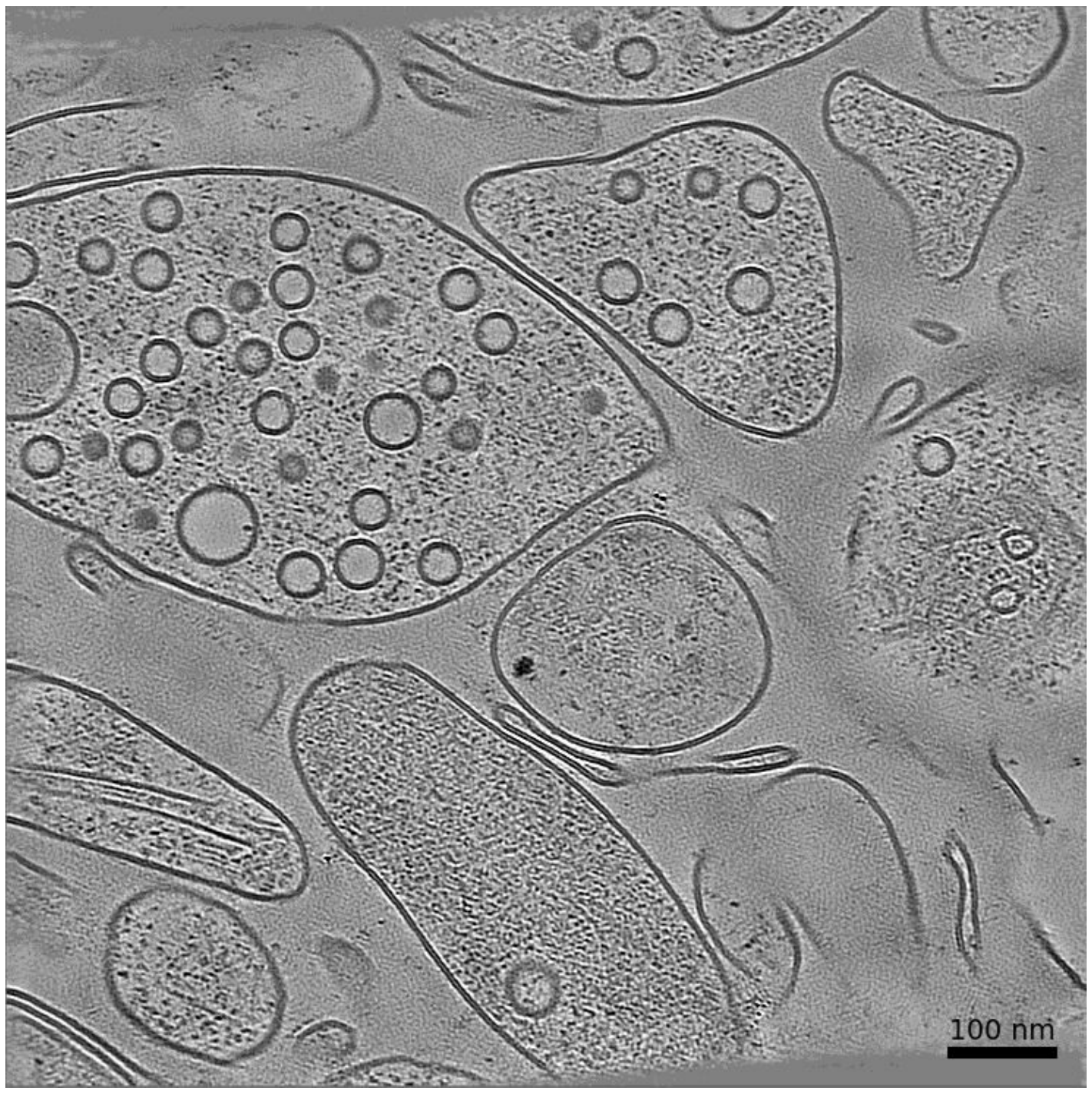

Membrane Segmentation & Cleft Position

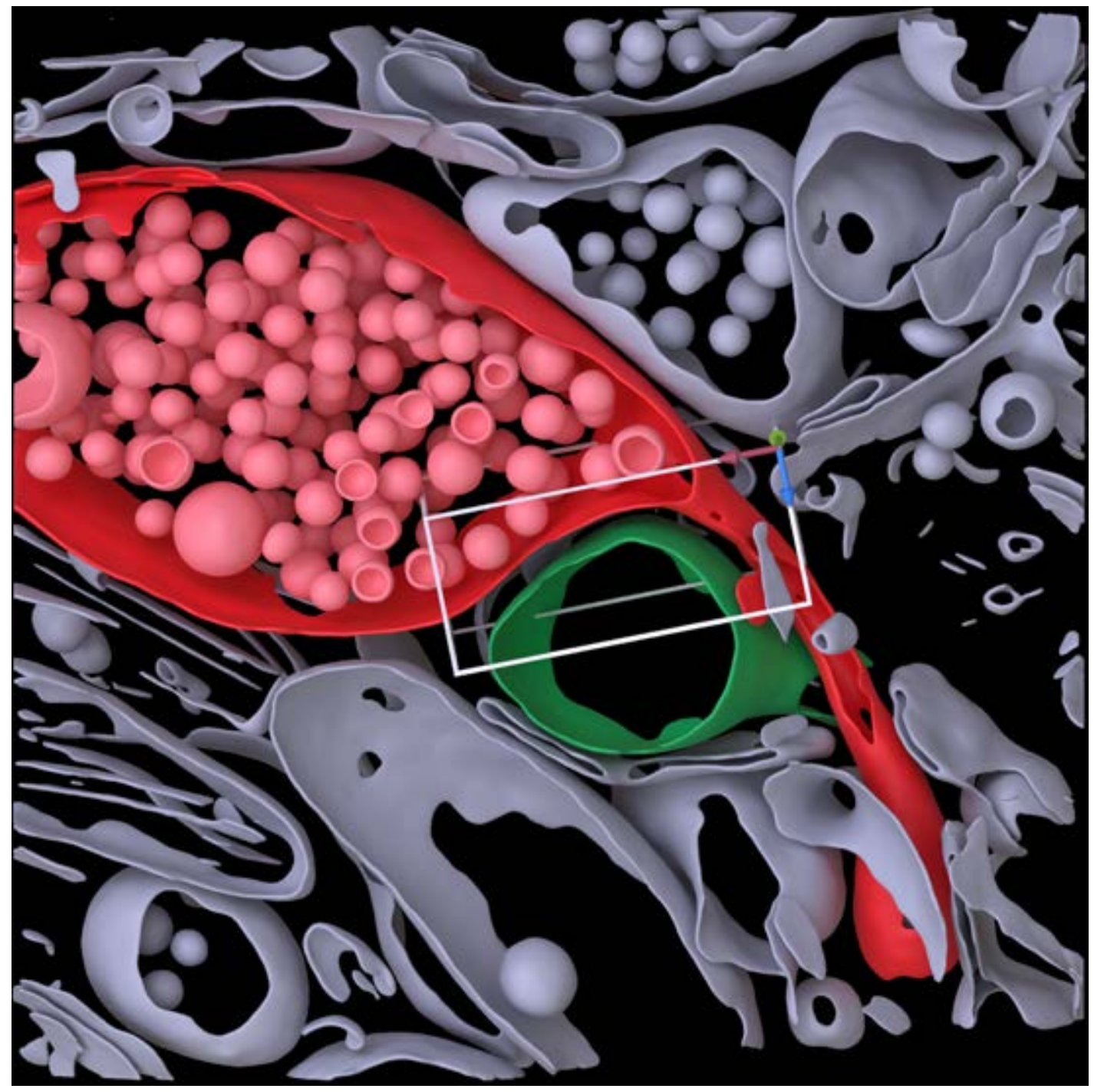

Cleft MIP

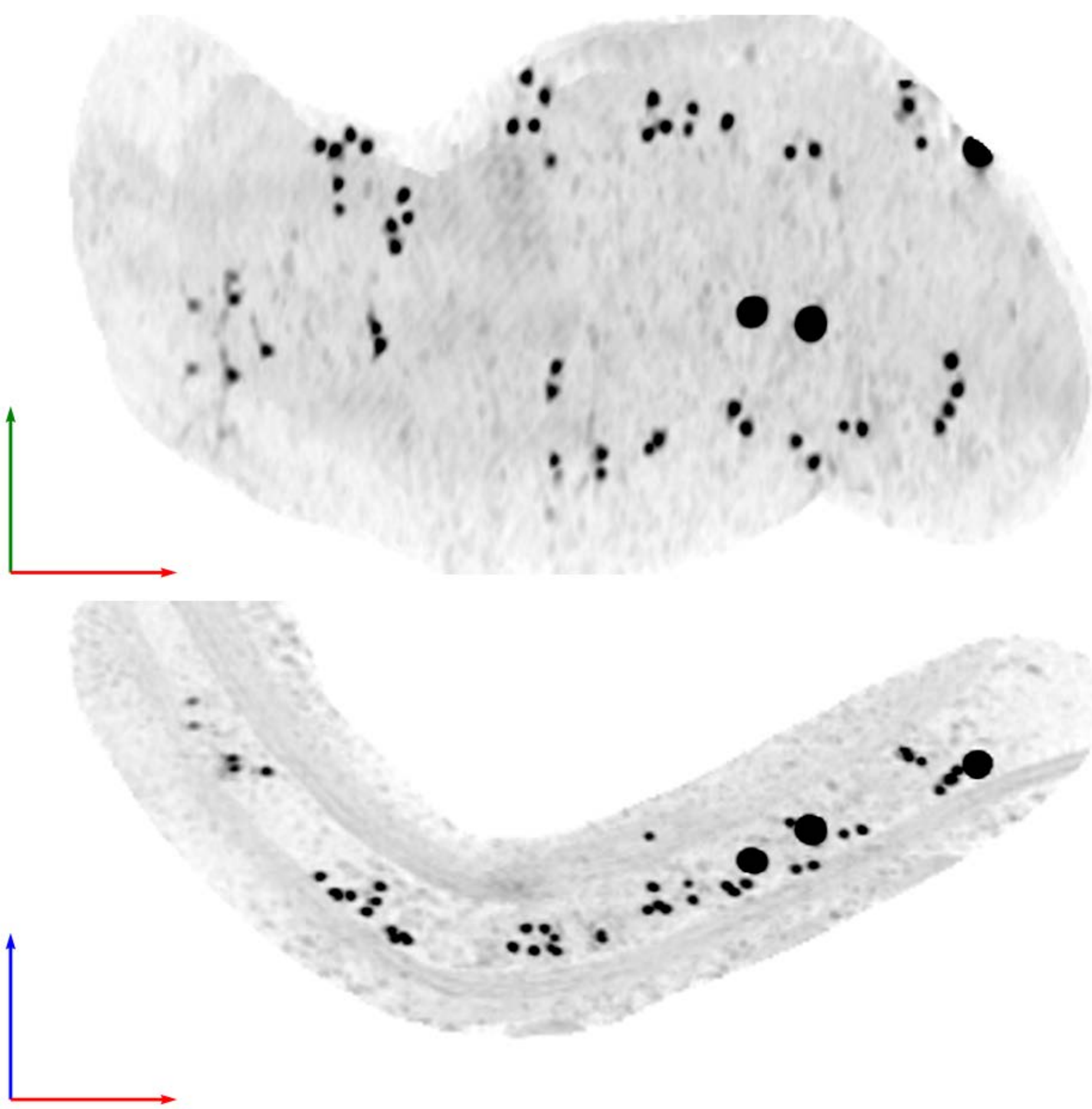

Tomogram ID: AMmilled55-1_Position_49	SET: anti-GluA2 15F1 "H4K-2Cys"
Cleft ID: 0	Type: tissue

Denoised Tomogram Slice

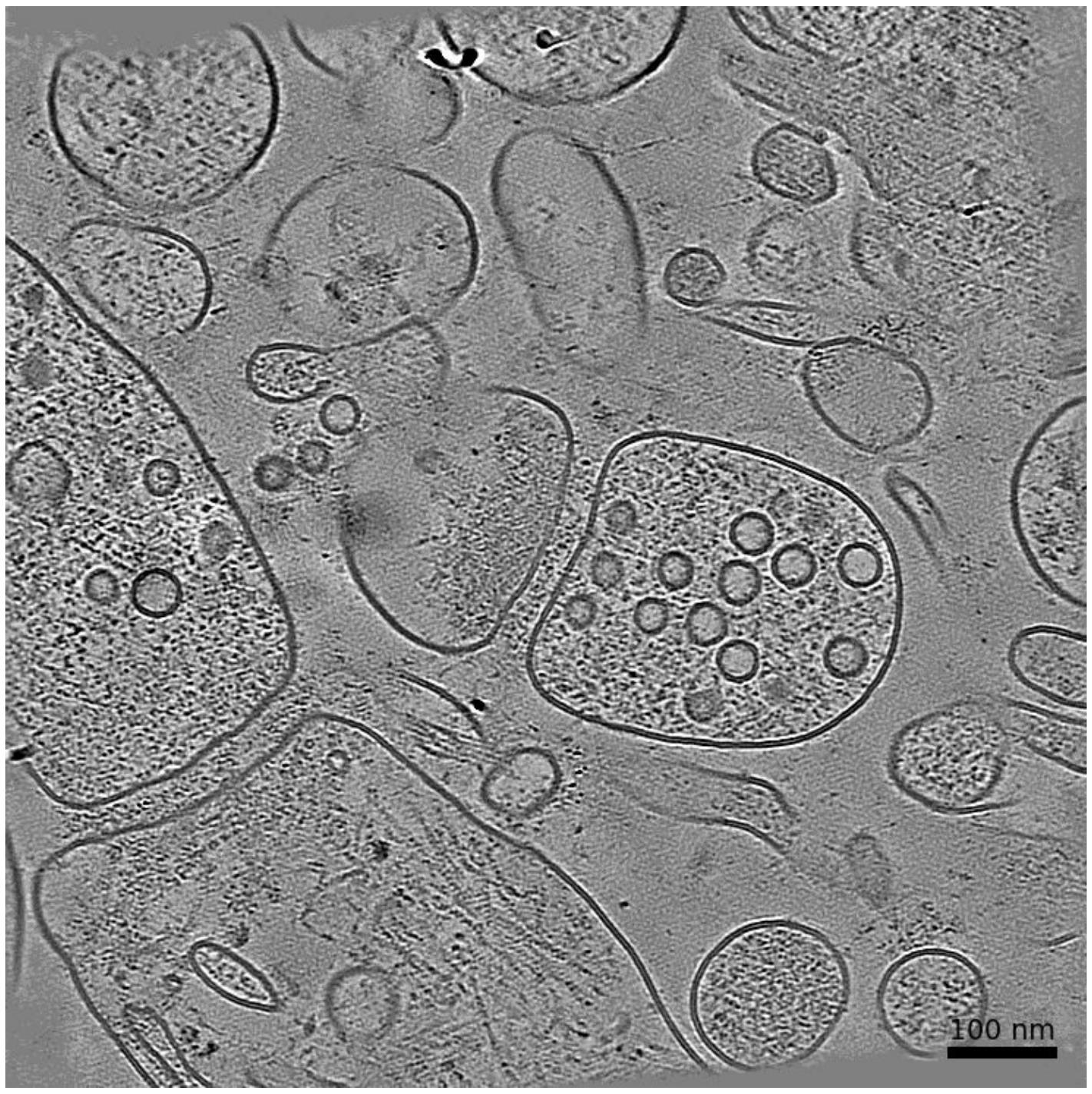

Membrane Segmentation & Cleft Position

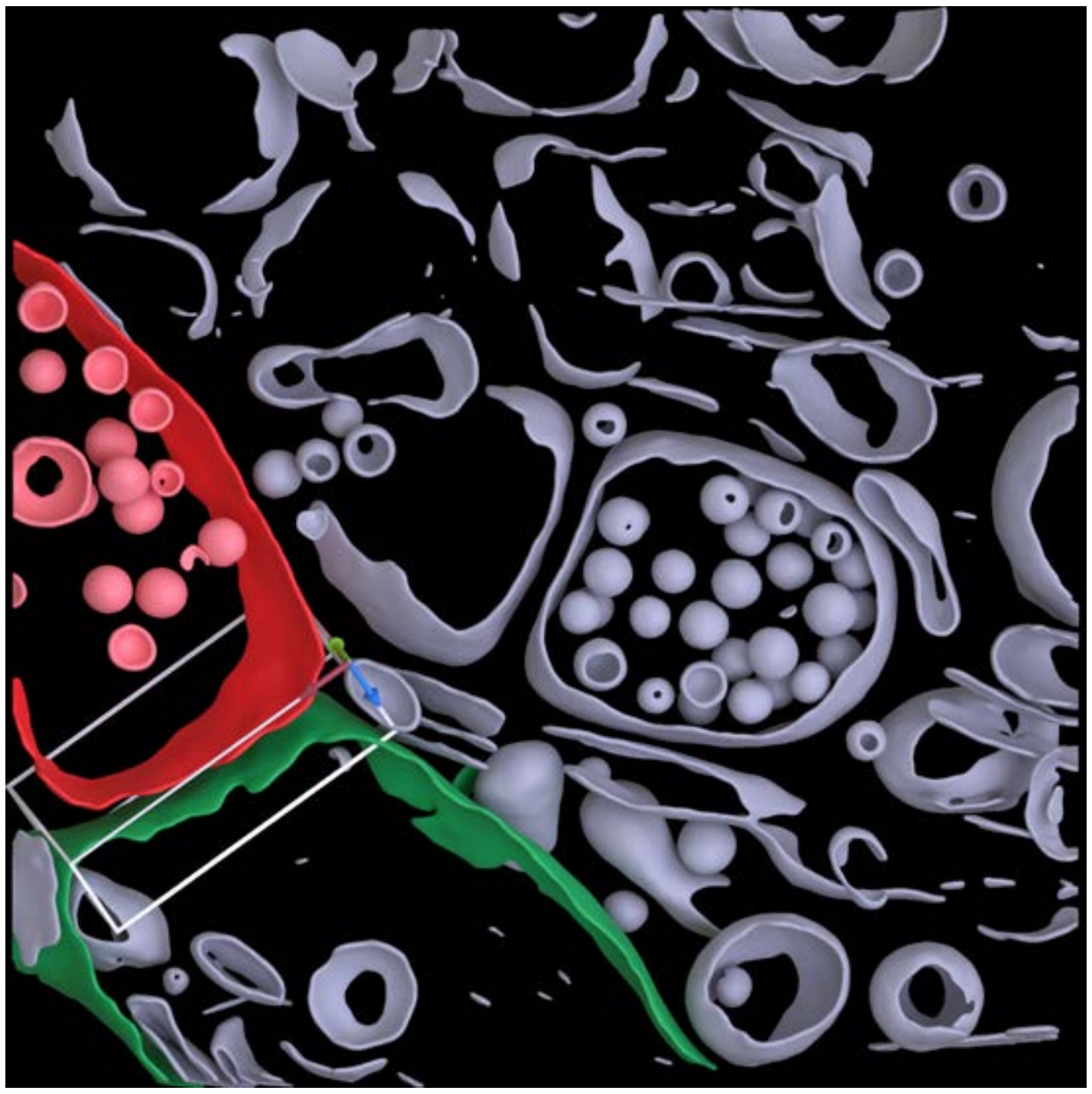

Cleft MIP

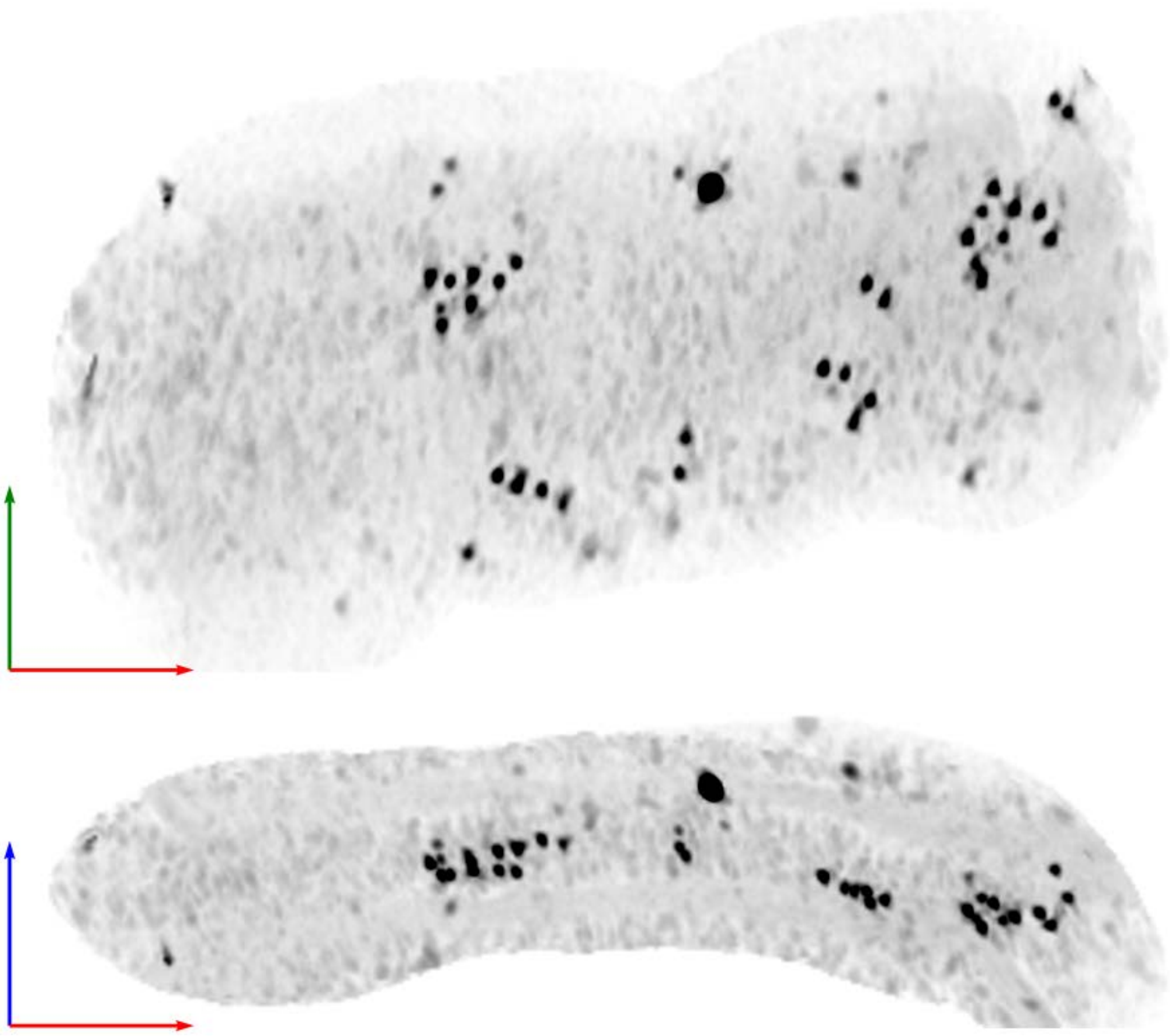

Tomogram ID: AMmilled55-1_Position_49 | **SET: anti-GluA2 15F1 "H4K-2Cys"**

Cleft ID: 0 | Type: tissue

Denoised Tomogram Slice

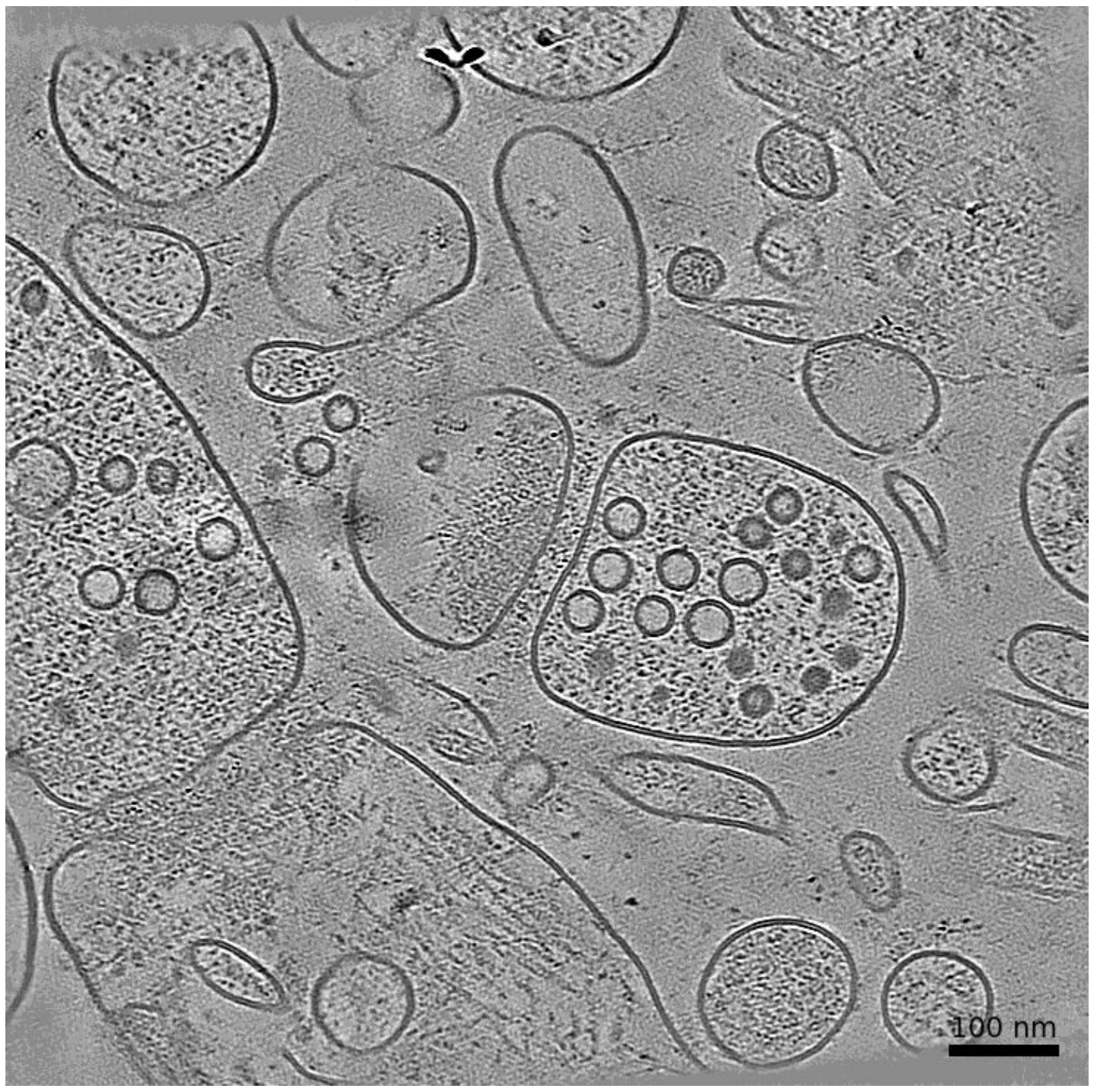

Membrane Segmentation & Cleft Position

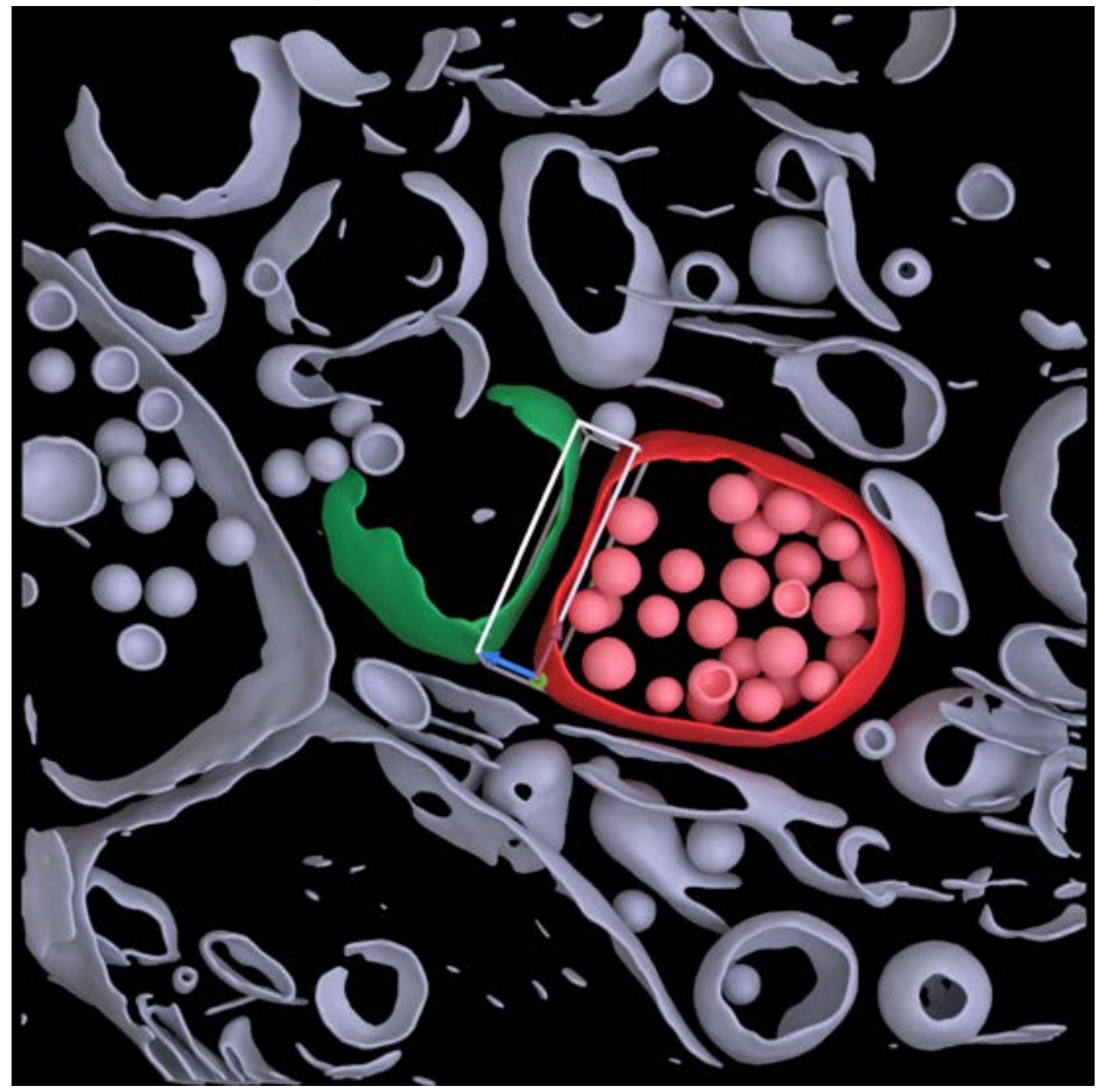

Cleft MIP

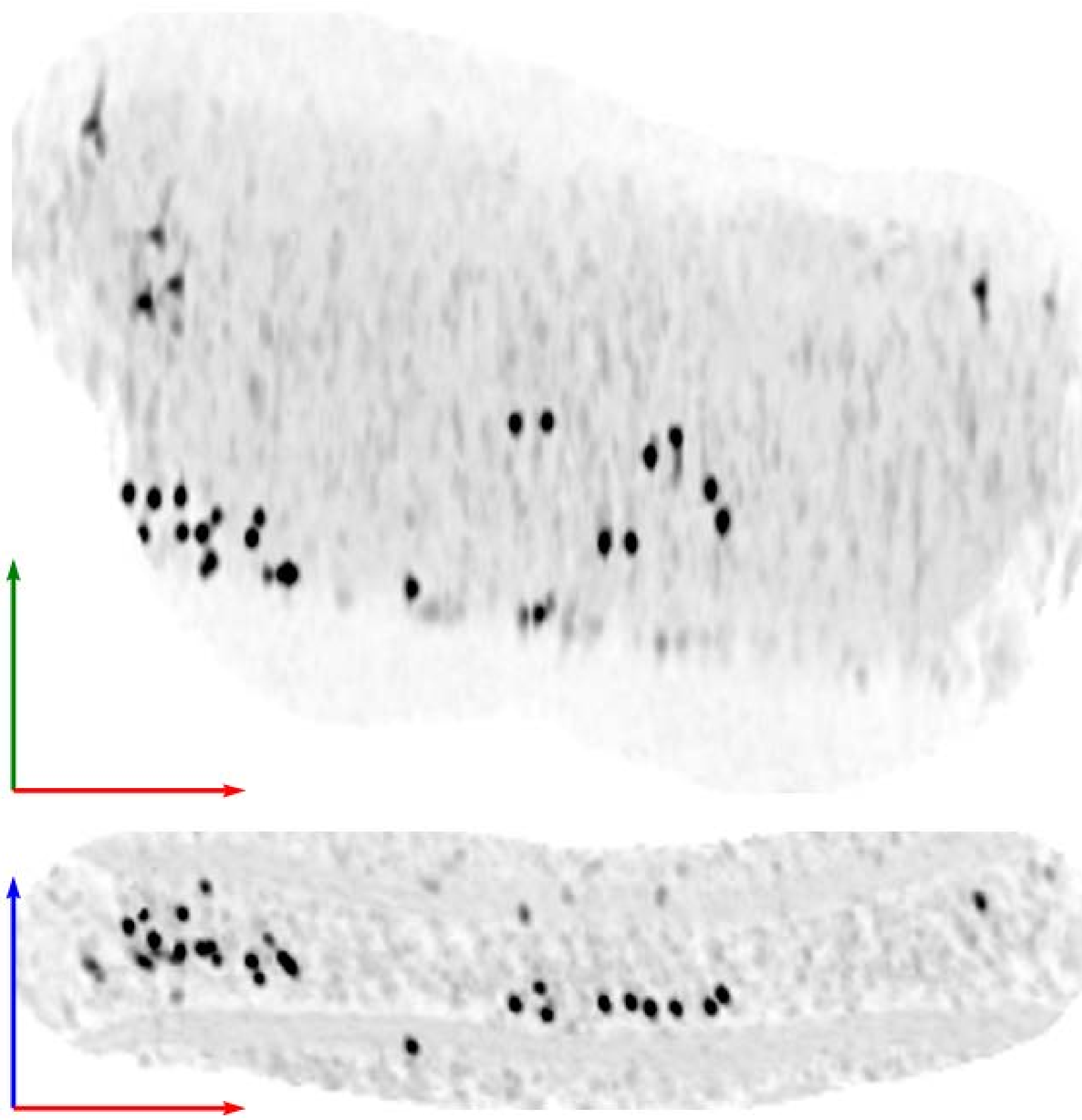

Tomogram ID: AMmilled55-1_Position_75	SET: anti-GluA2 15F1 "H4K-2Cys"
Cleft ID: 0	Type: tissue

Denoised Tomogram Slice

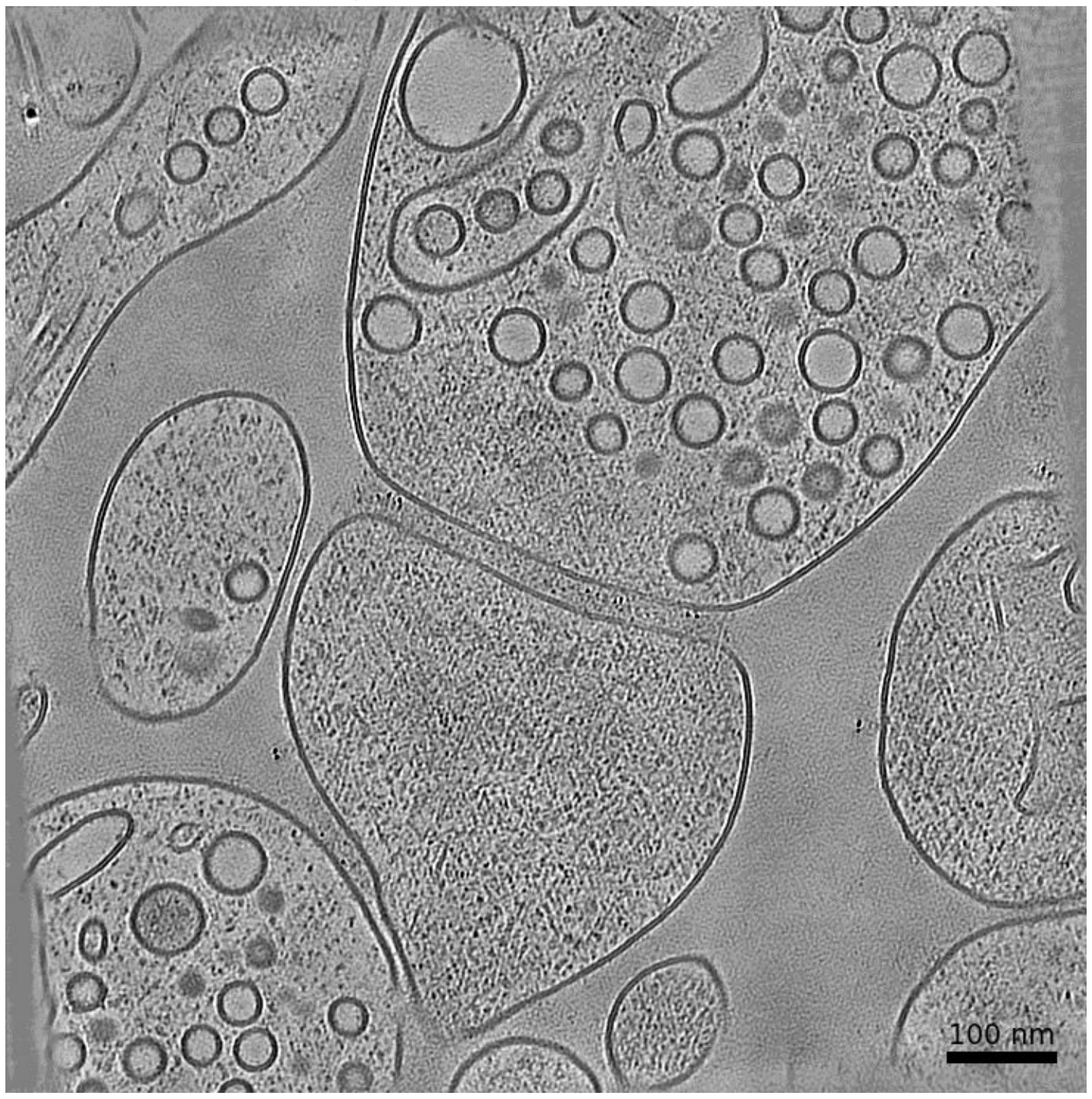

Membrane Segmentation & Cleft Position

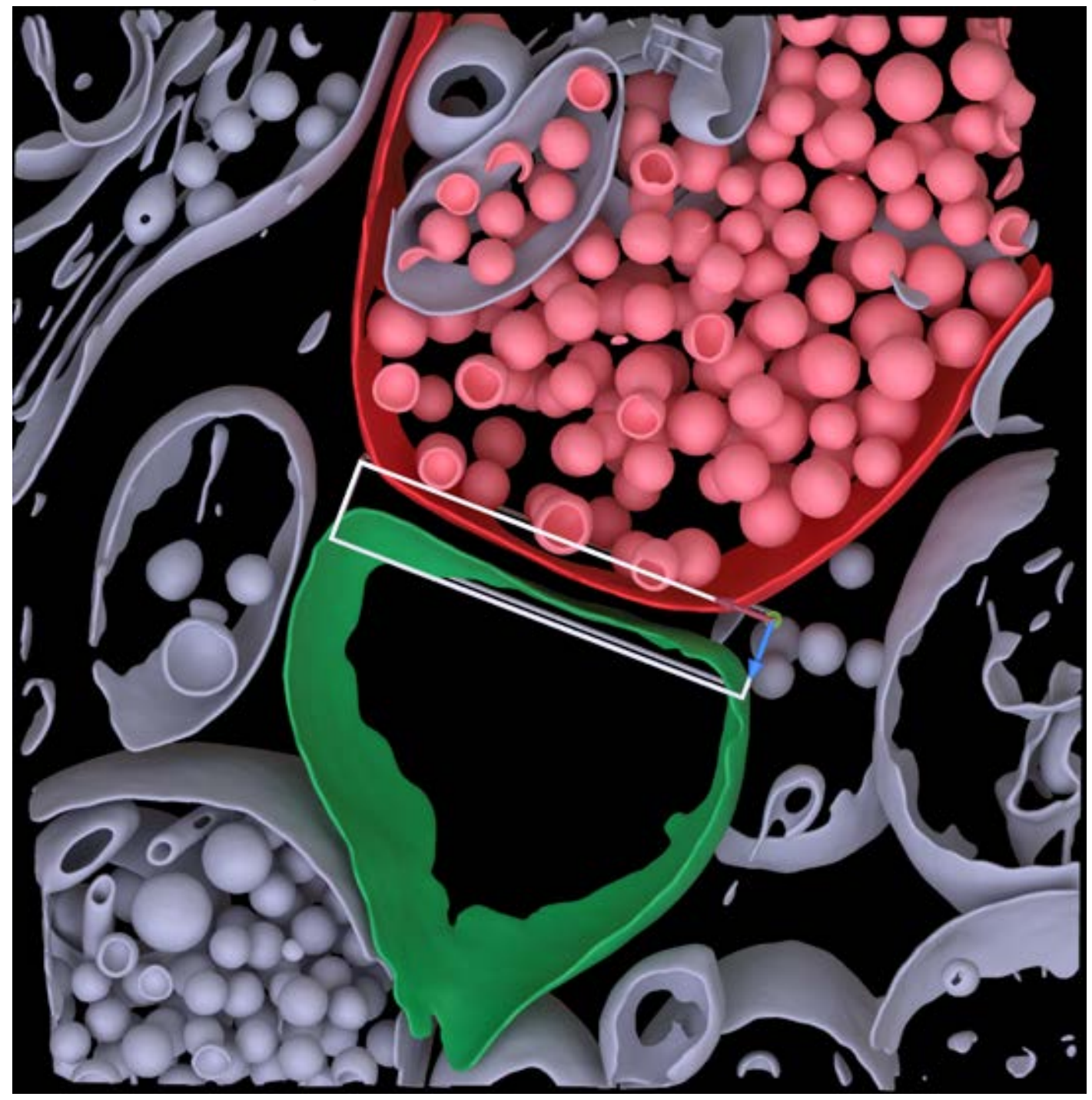

Cleft MIP

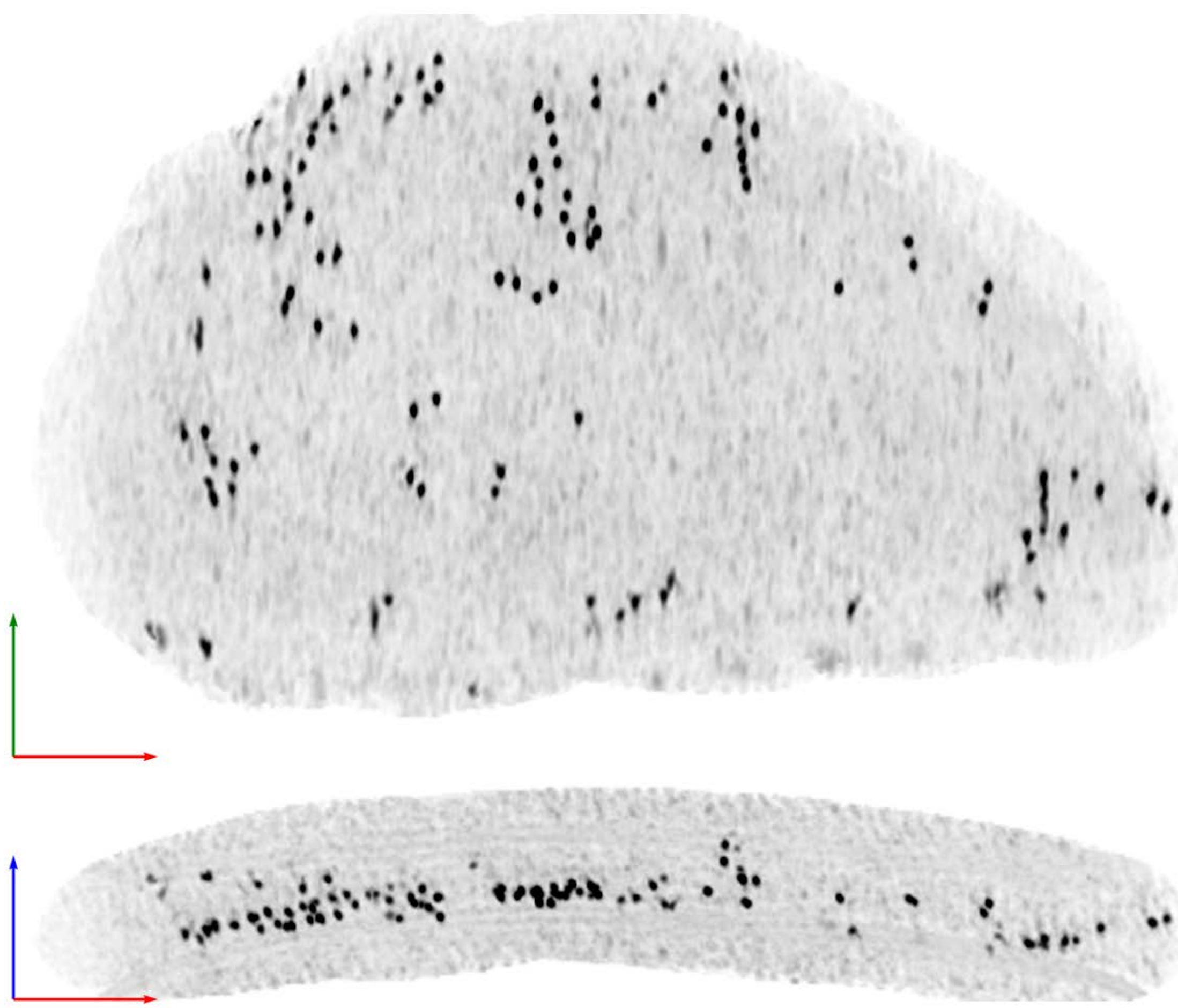

Tomogram ID: AMmilled55-1_Position_75	SET: anti-GluA2 15F1 "H4K-2Cys"
Cleft ID: 0	Type: tissue

Denoised Tomogram Slice

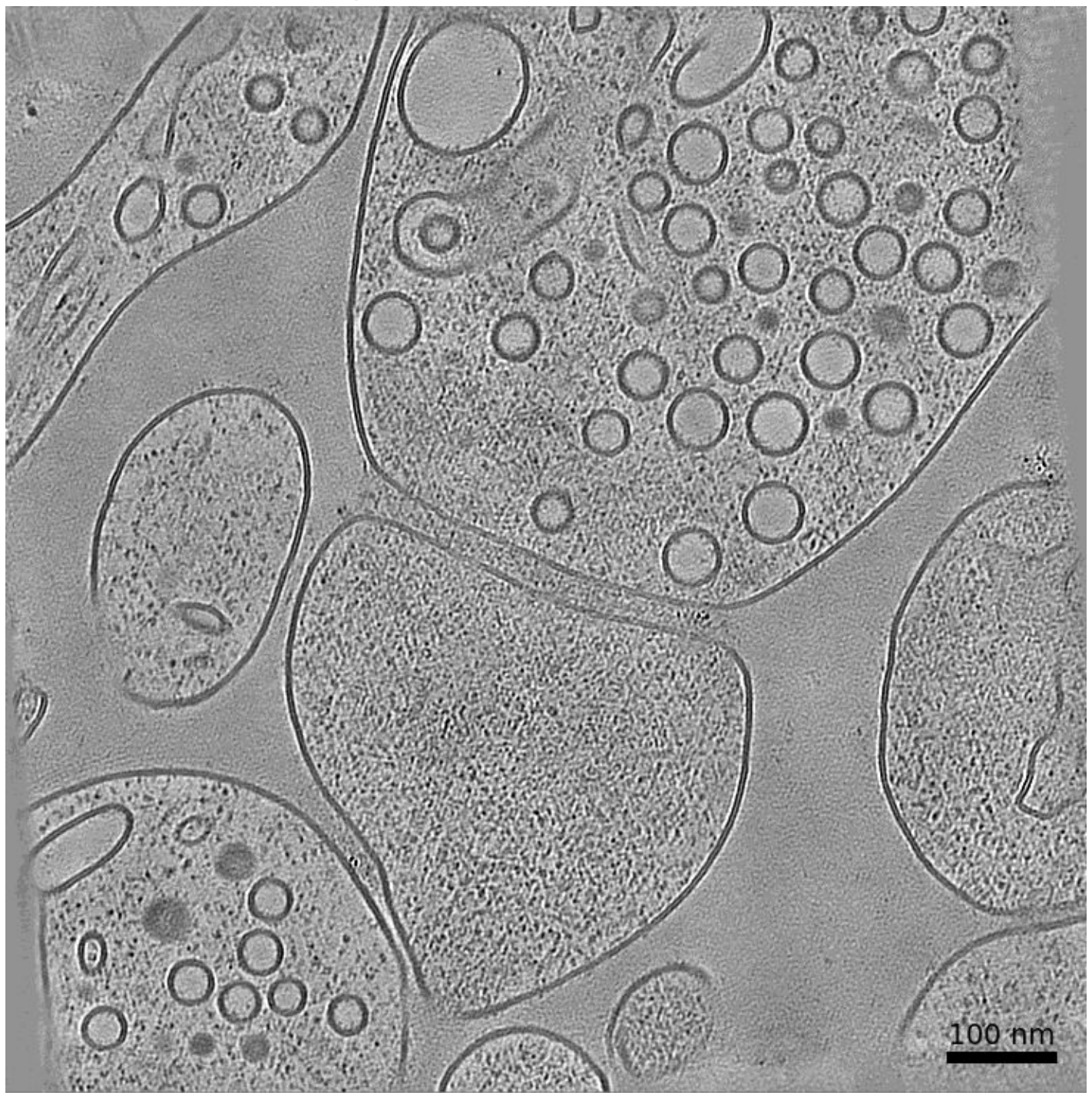

Membrane Segmentation & Cleft Position

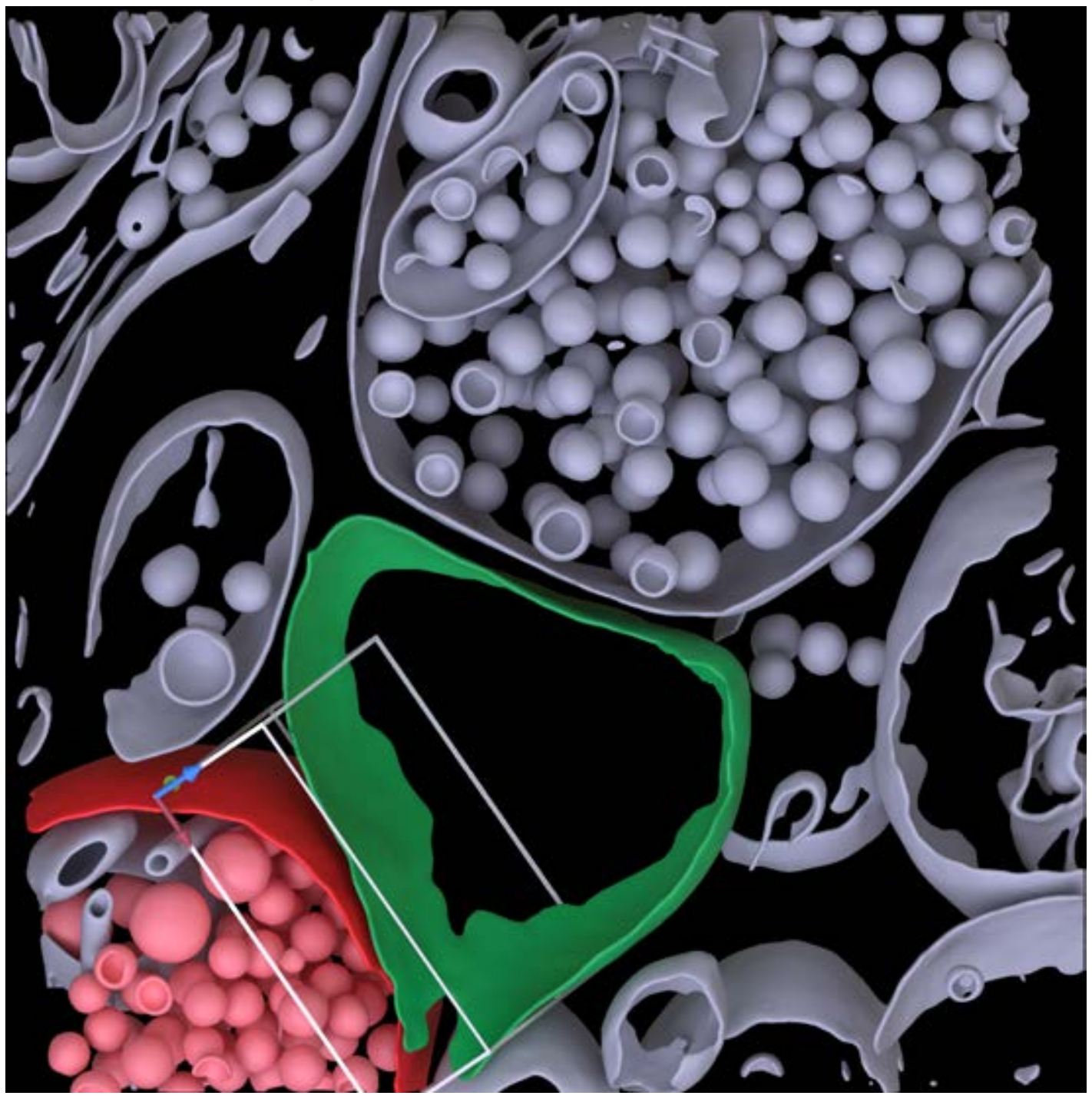

Cleft MIP

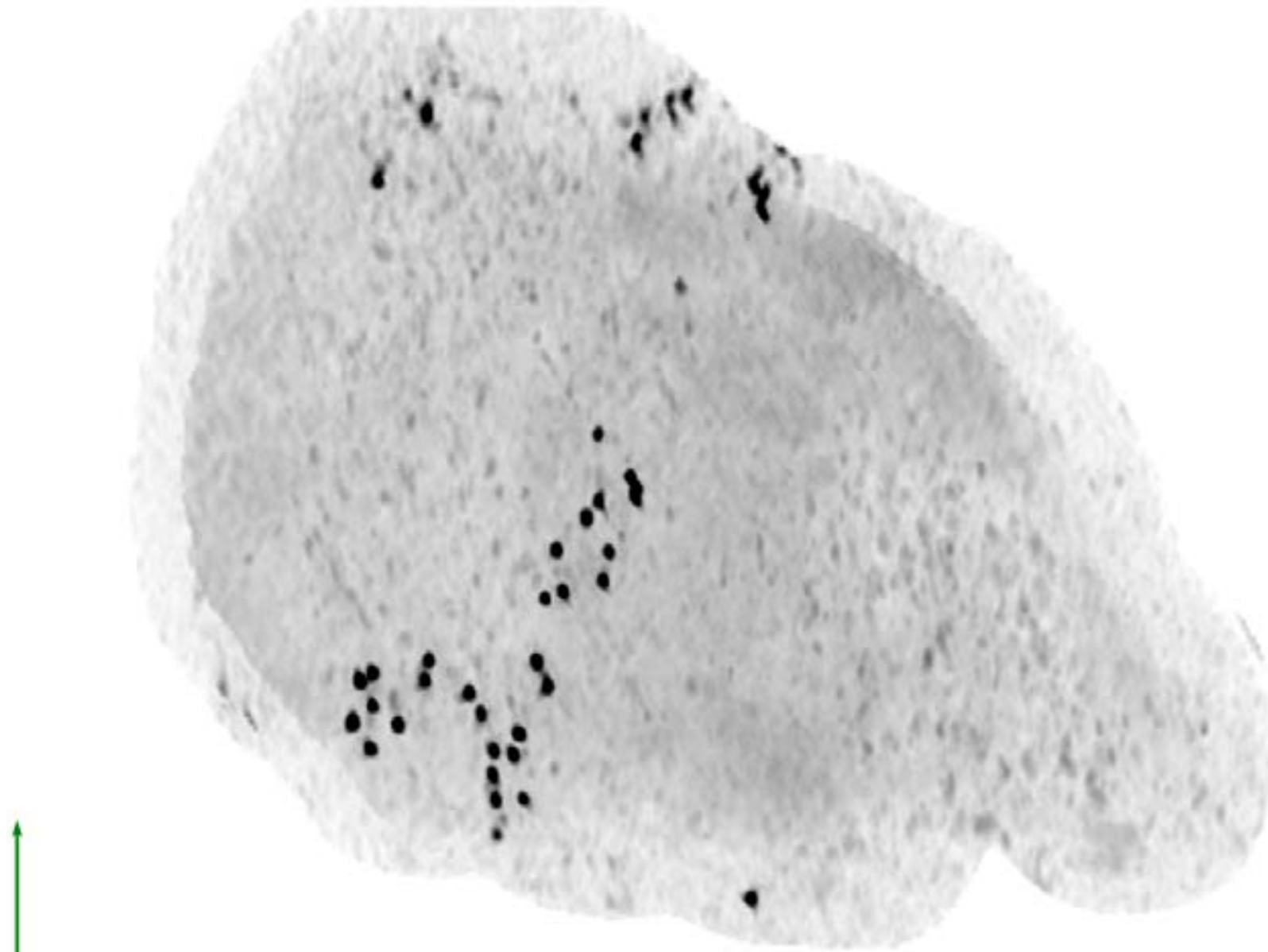

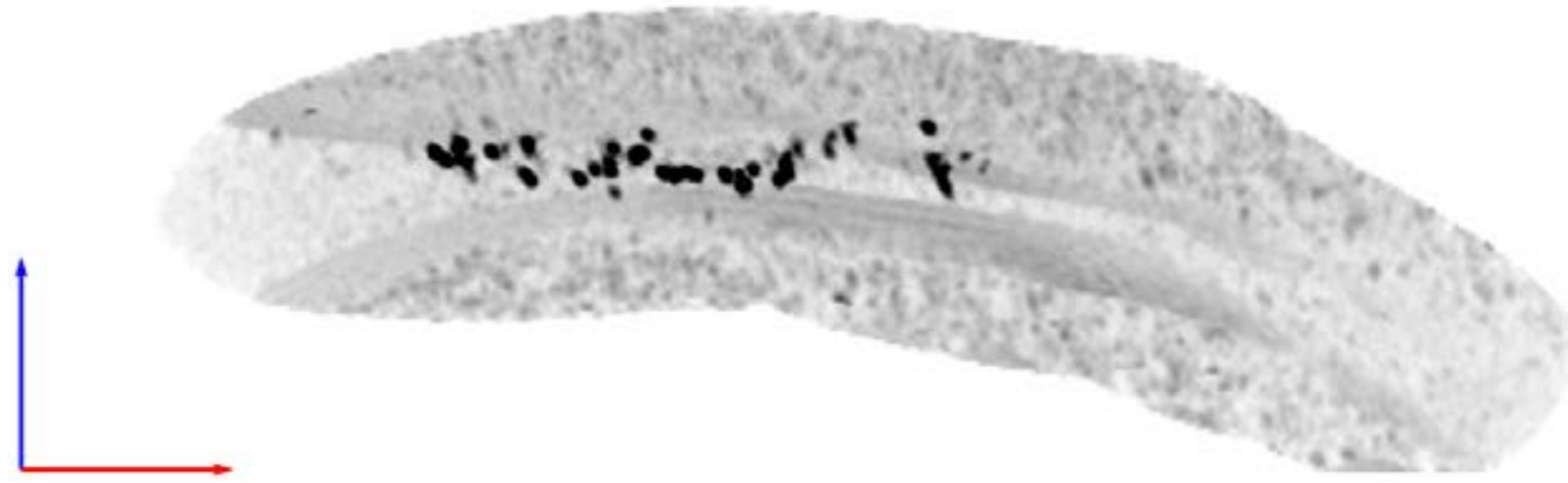

Tomogram ID: AMmilled55-1_Position_82 | **SET: anti-GluA2 15F1 "H4K-2Cys"**

Cleft ID: 0 | Type: tissue

Denoised Tomogram Slice

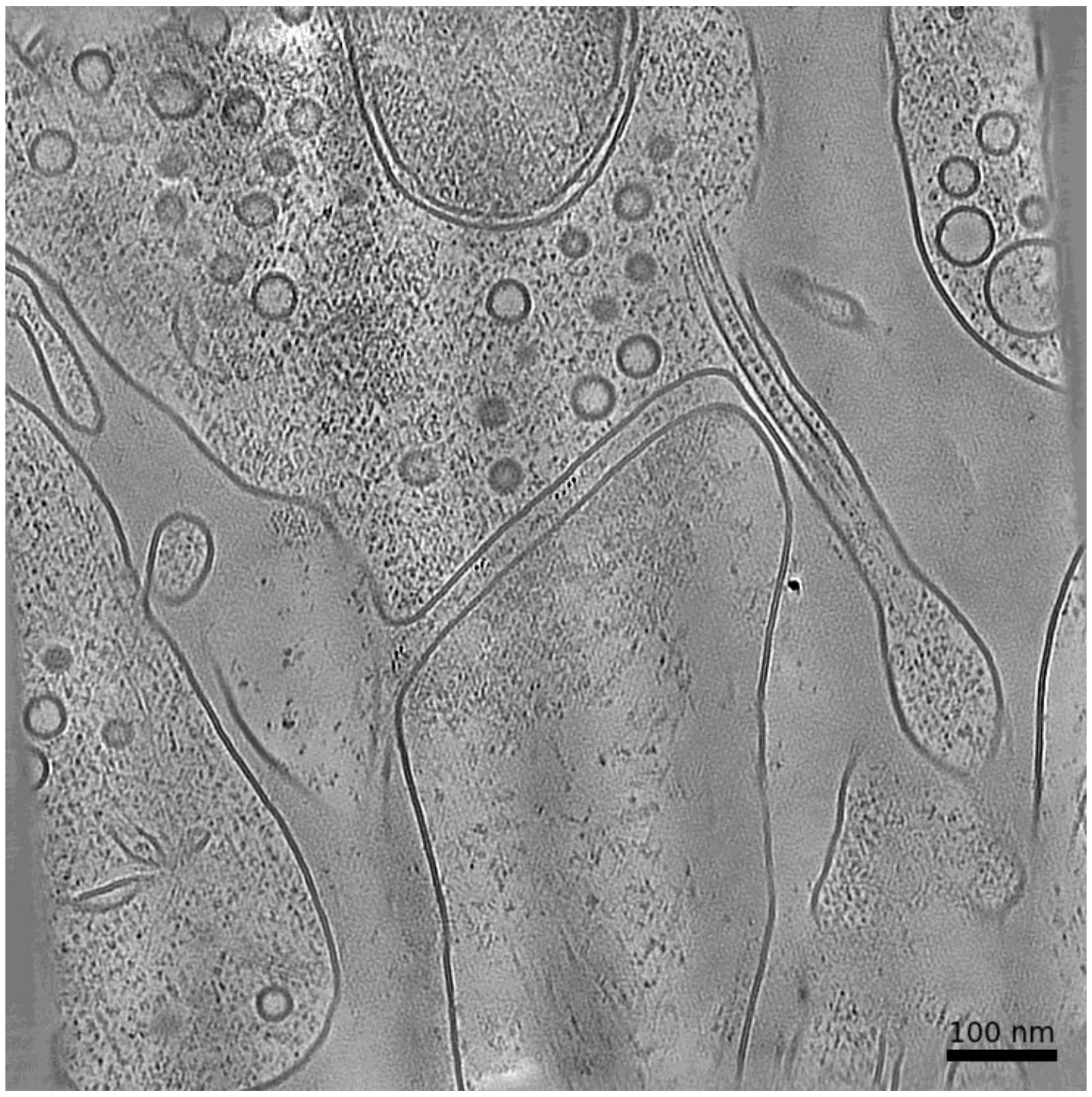

Membrane Segmentation & Cleft Position

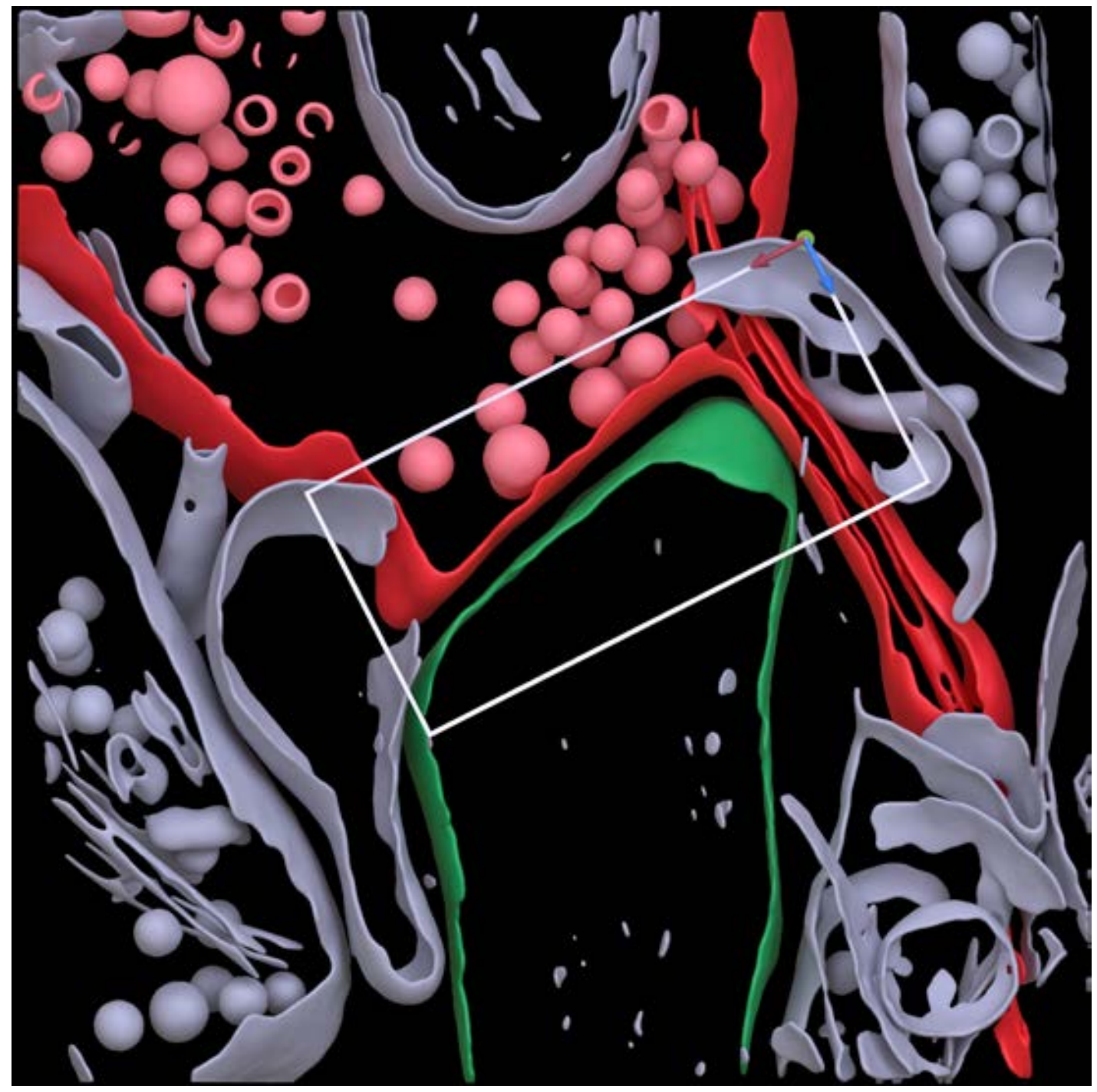

Cleft MIP

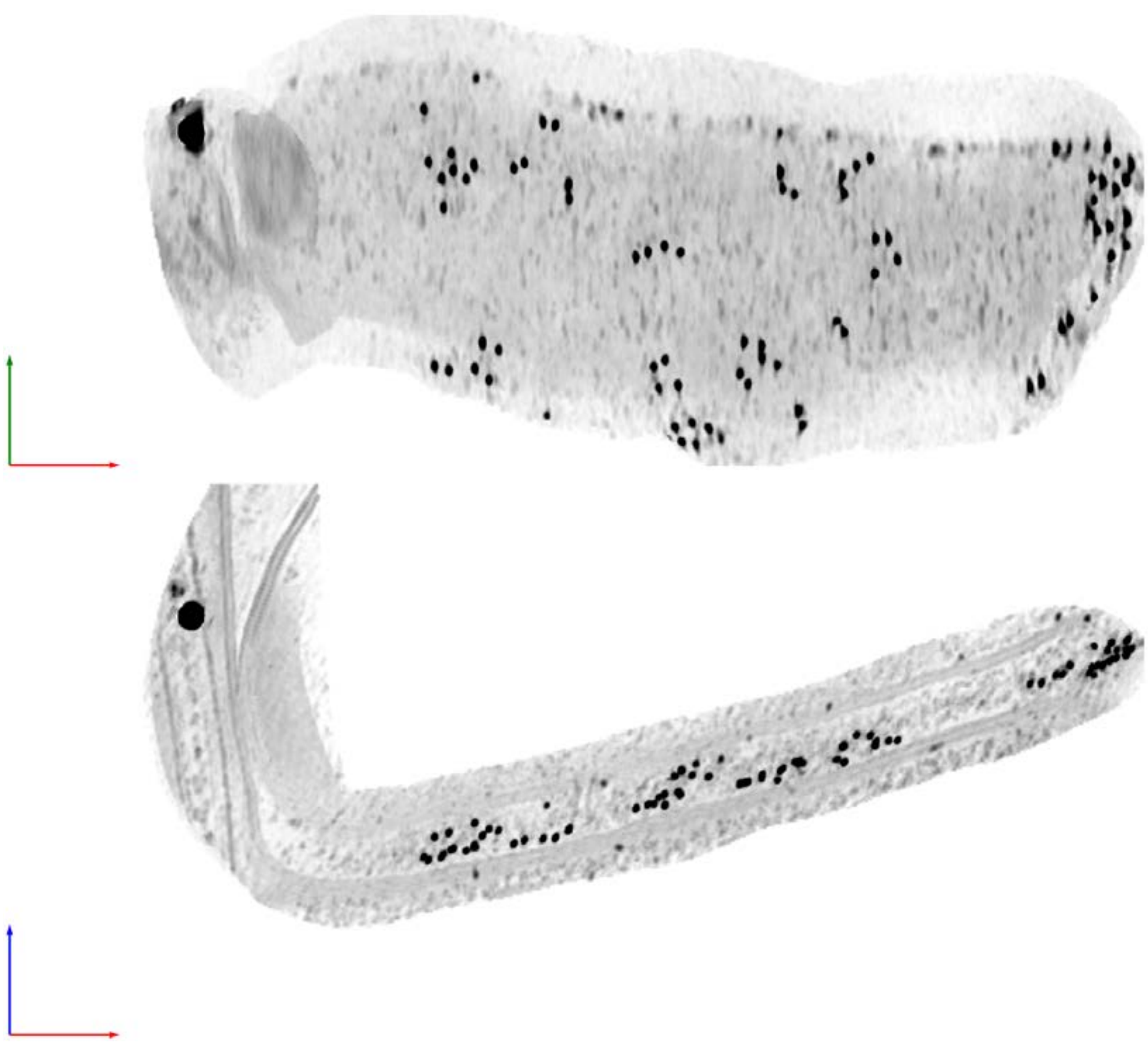

Tomogram ID: AMmilled55-1_Position_82	SET: anti-GluA2 15F1 "H4K-2Cys"
Cleft ID: 0	Type: tissue

Denoised Tomogram Slice

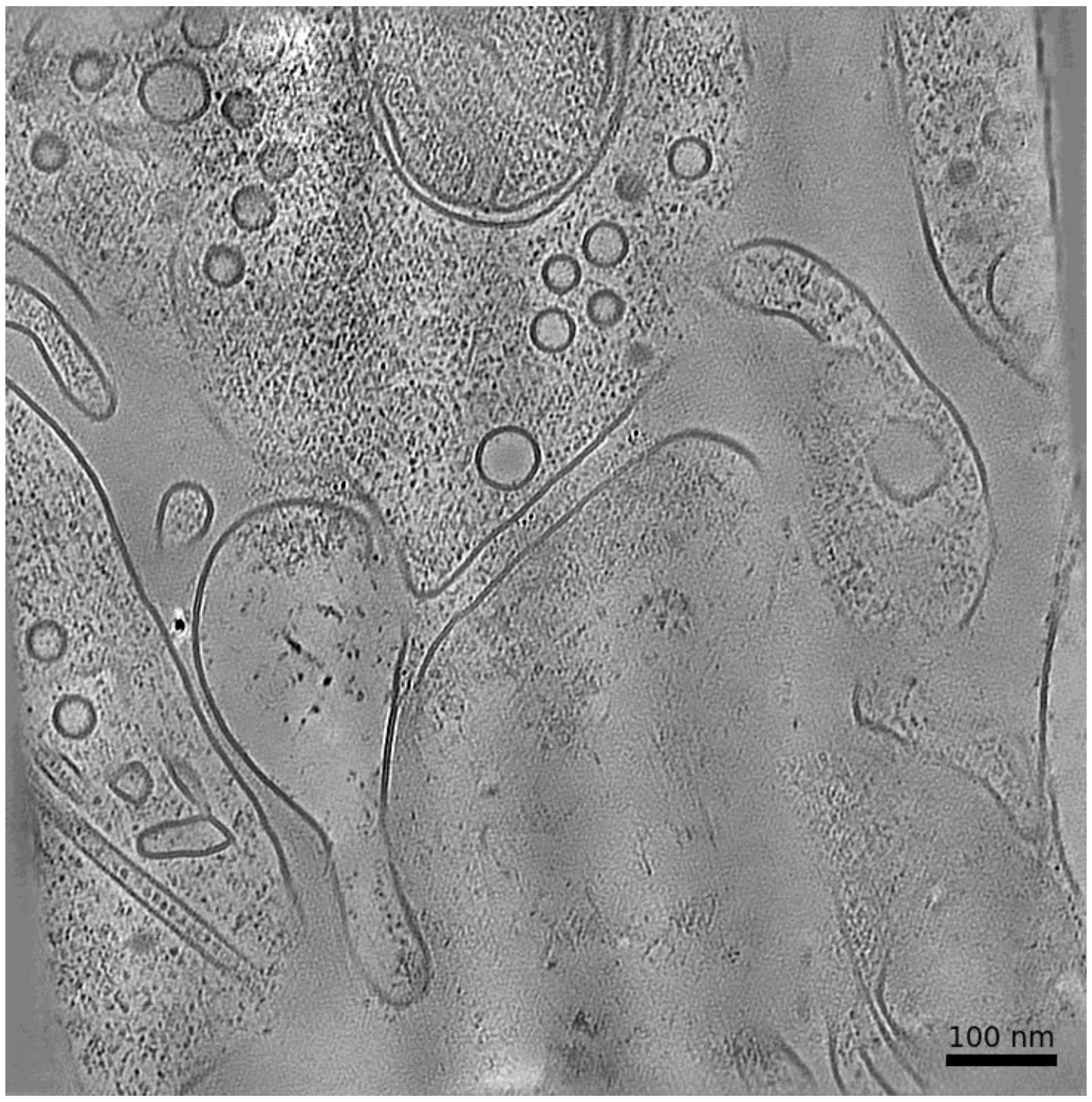

Membrane Segmentation & Cleft Position

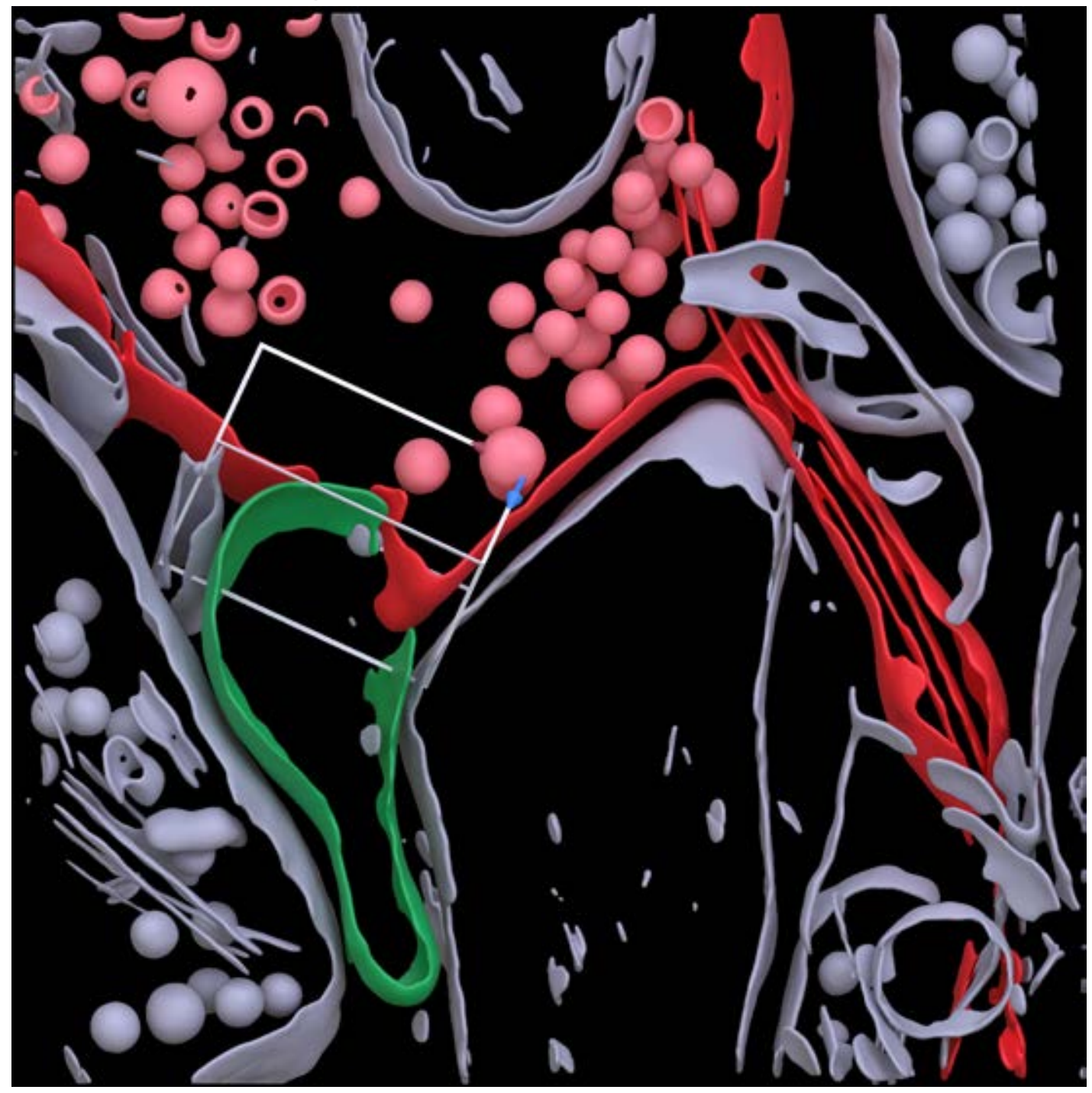

Cleft MIP

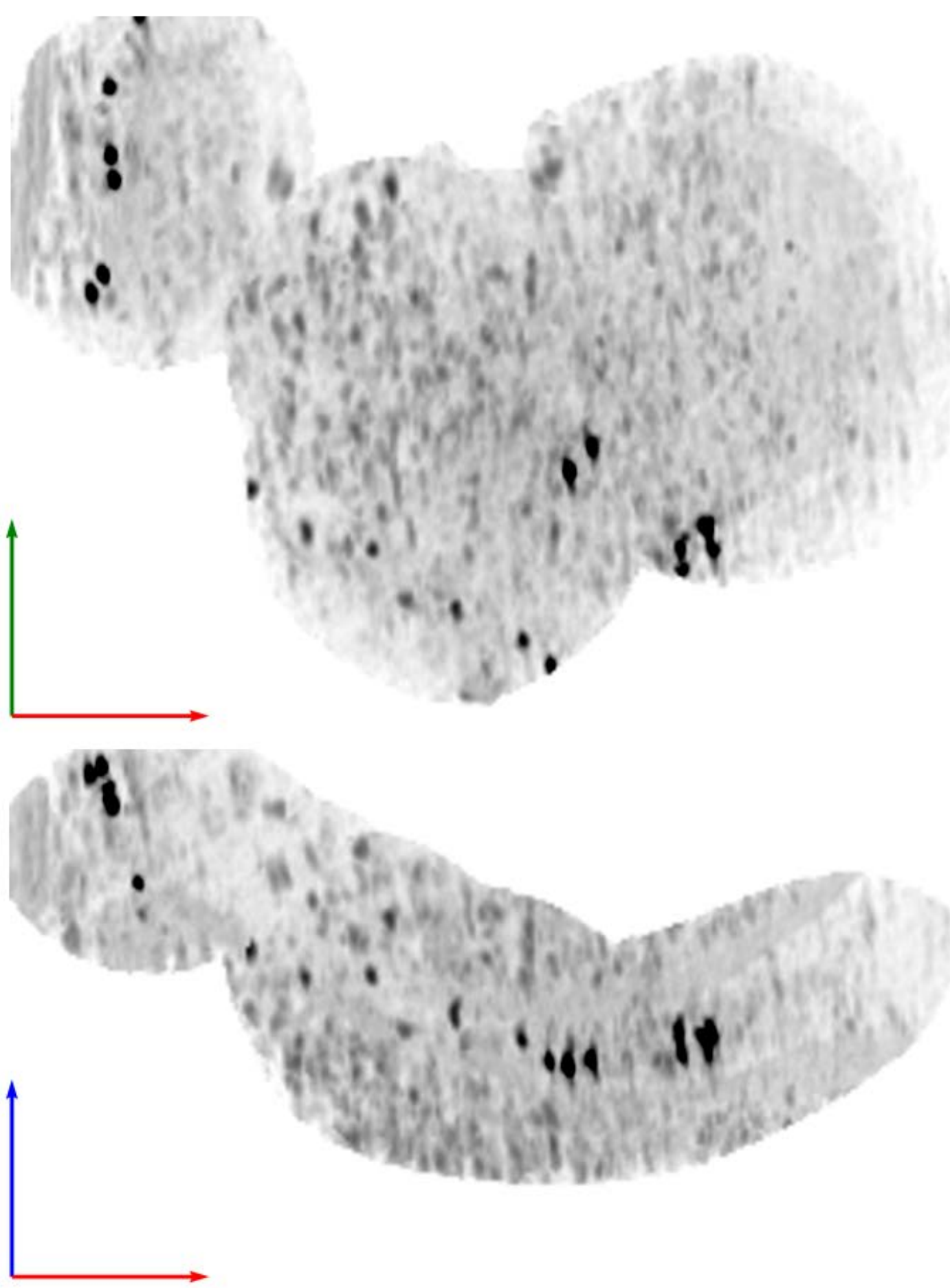

Tomogram ID: 20250206_AMmilled27-2_100	SET: anti-GluA1 11B8 "C-term"
Cleft ID: 0	Type: semi-tissue

Denoised Tomogram Slice

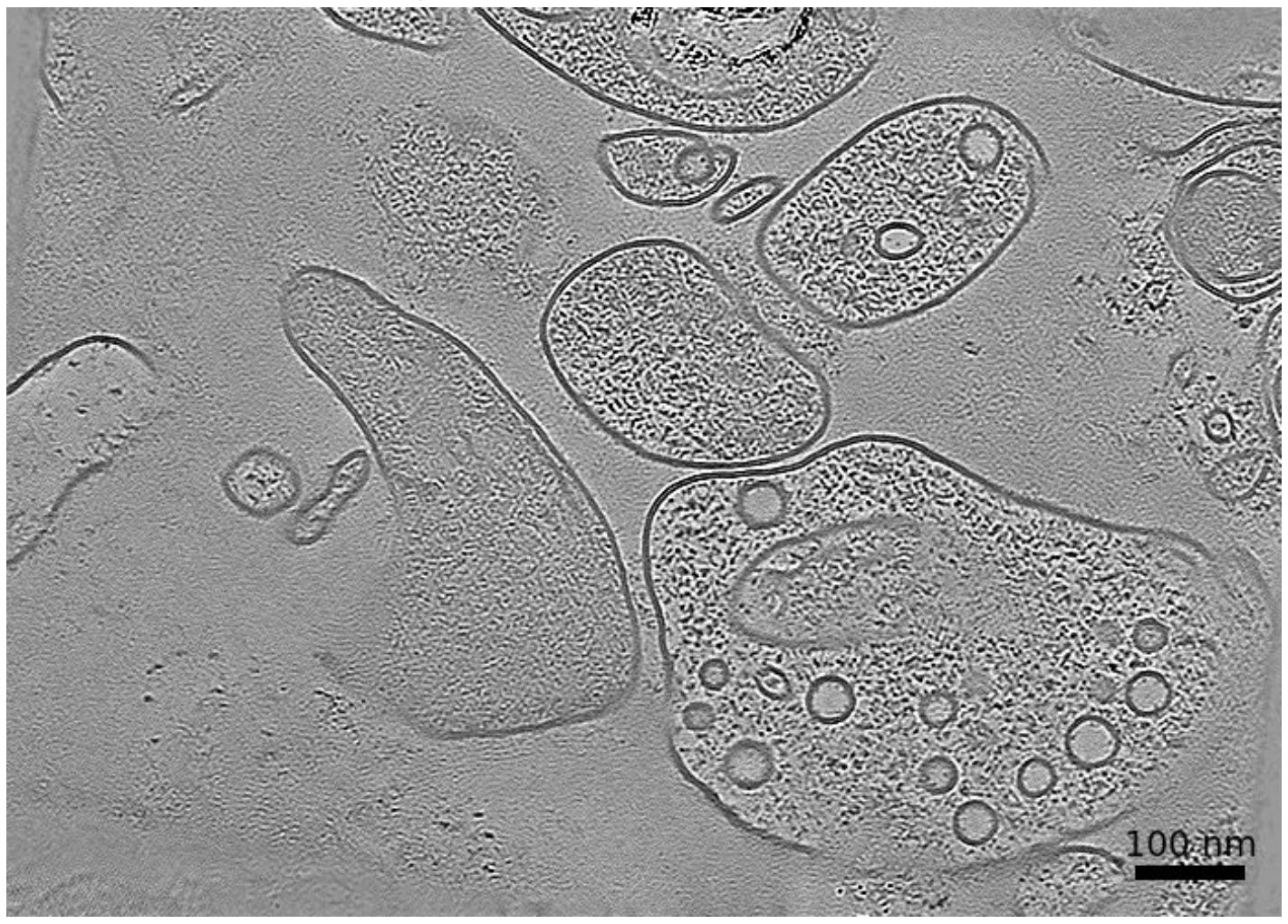

Membrane Segmentation & Cleft Position

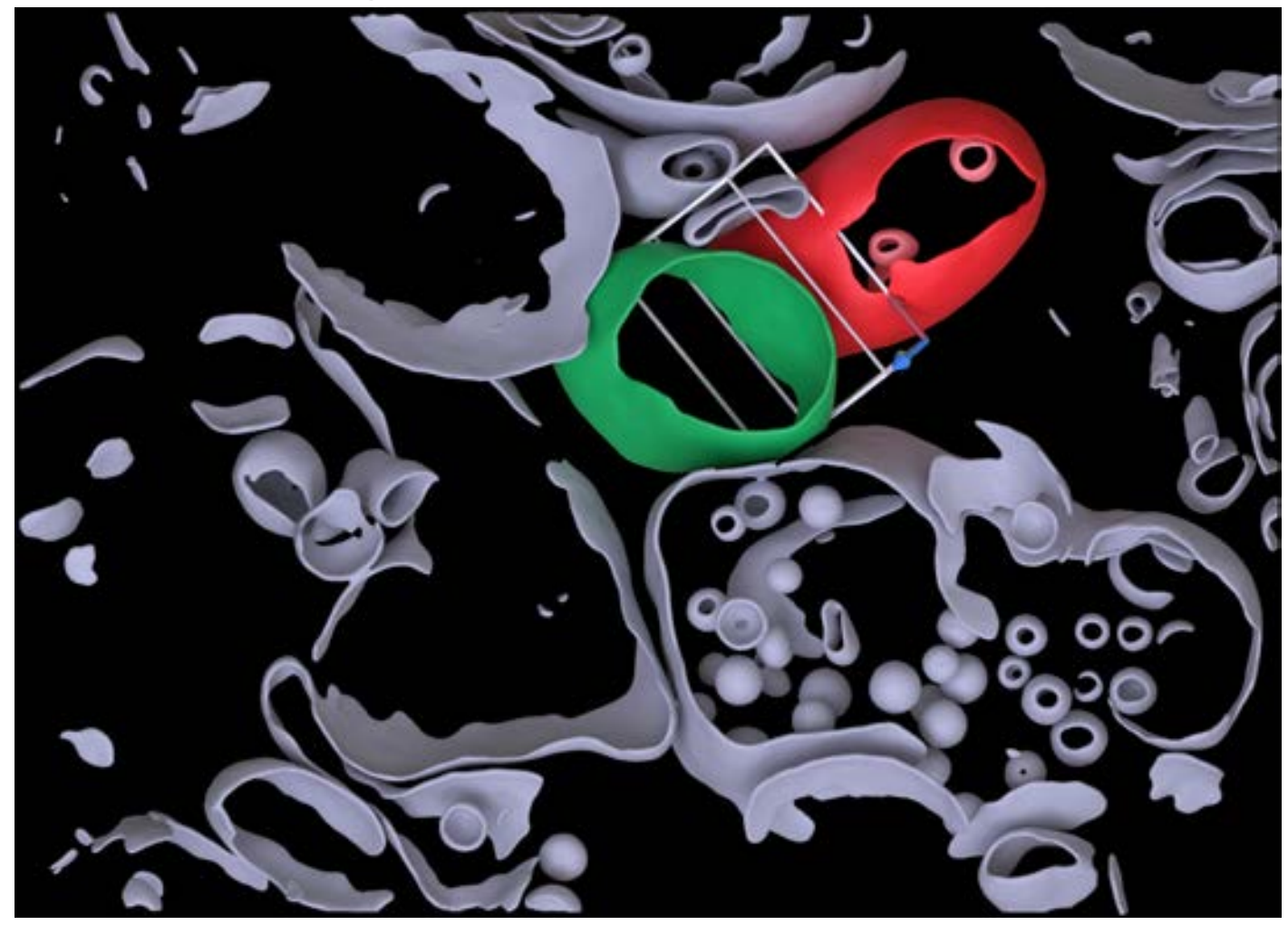

Cleft MIP

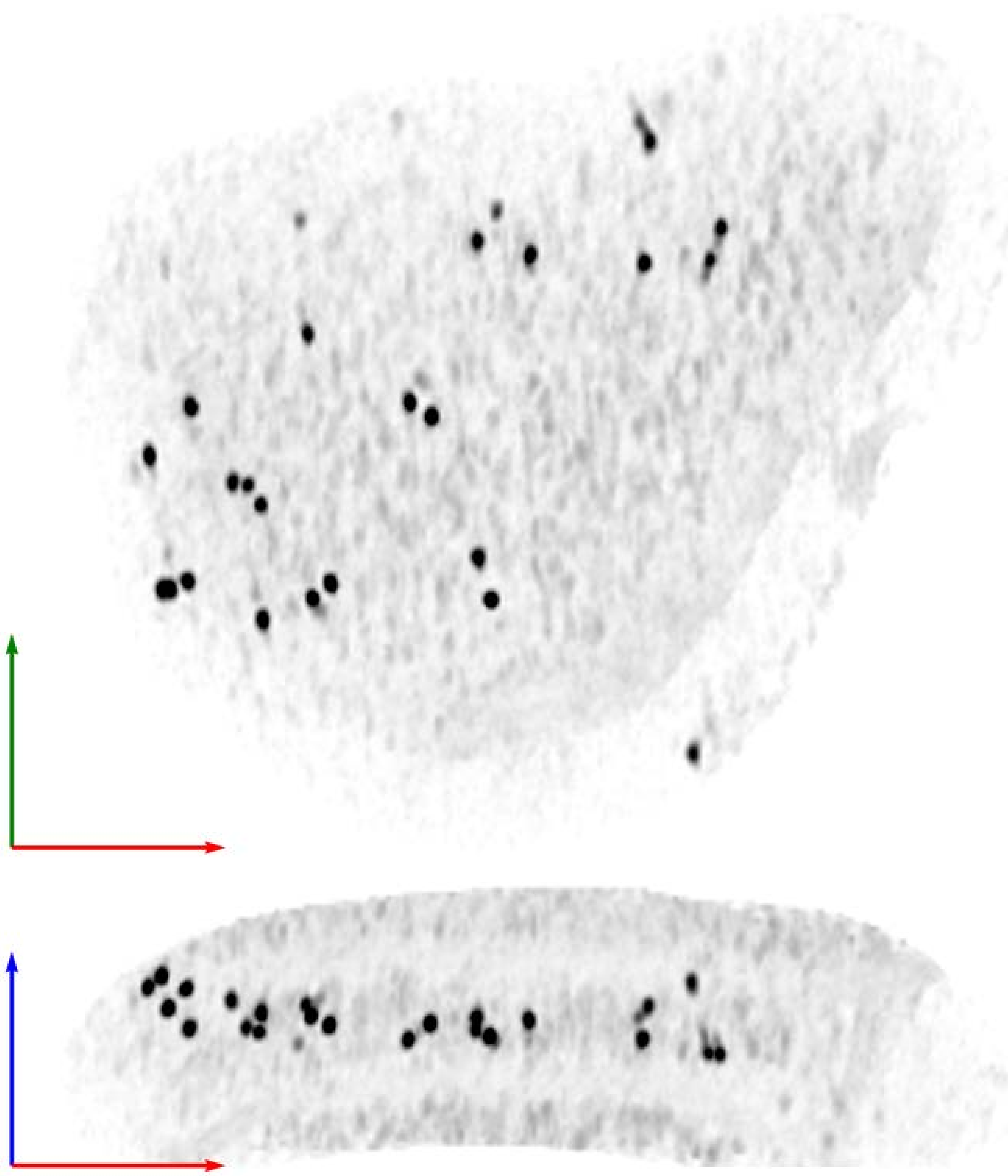

Tomogram ID: 20250206_AMmilled27-2_100	SET: anti-GluA1 11B8 "C-term"
Cleft ID: 0	Type: semi-tissue

Denoised Tomogram Slice

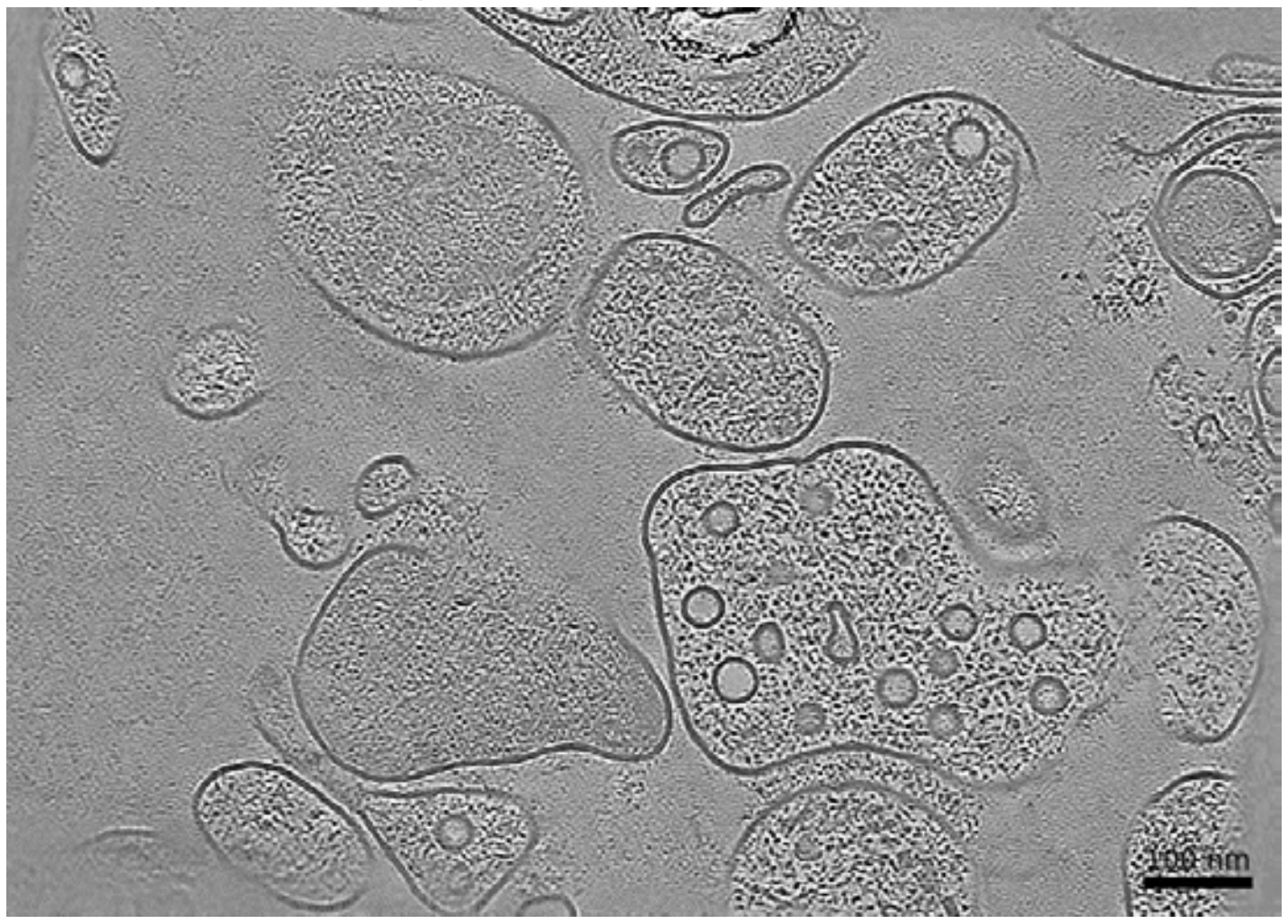

Membrane Segmentation & Cleft Position

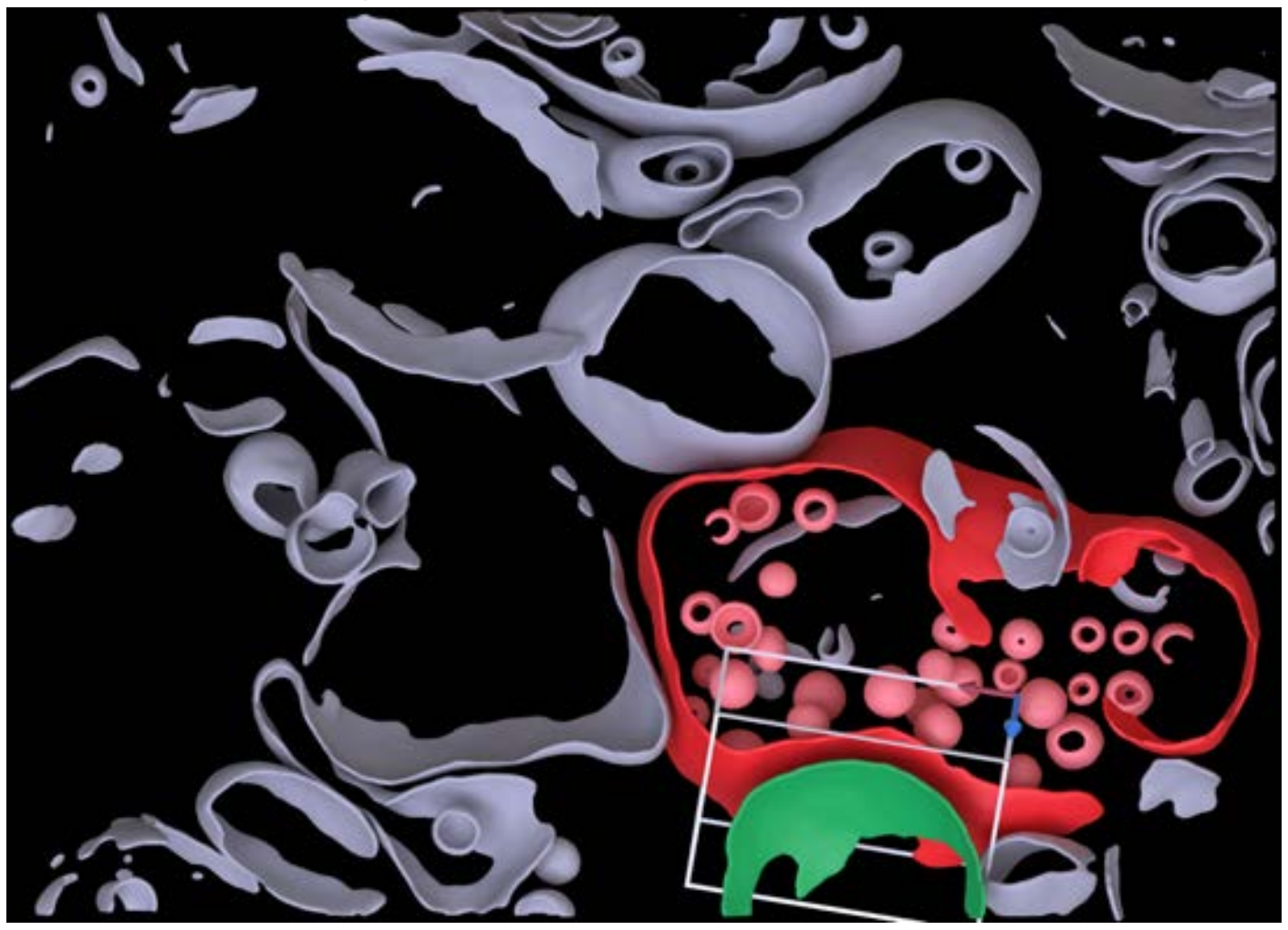

Cleft MIP

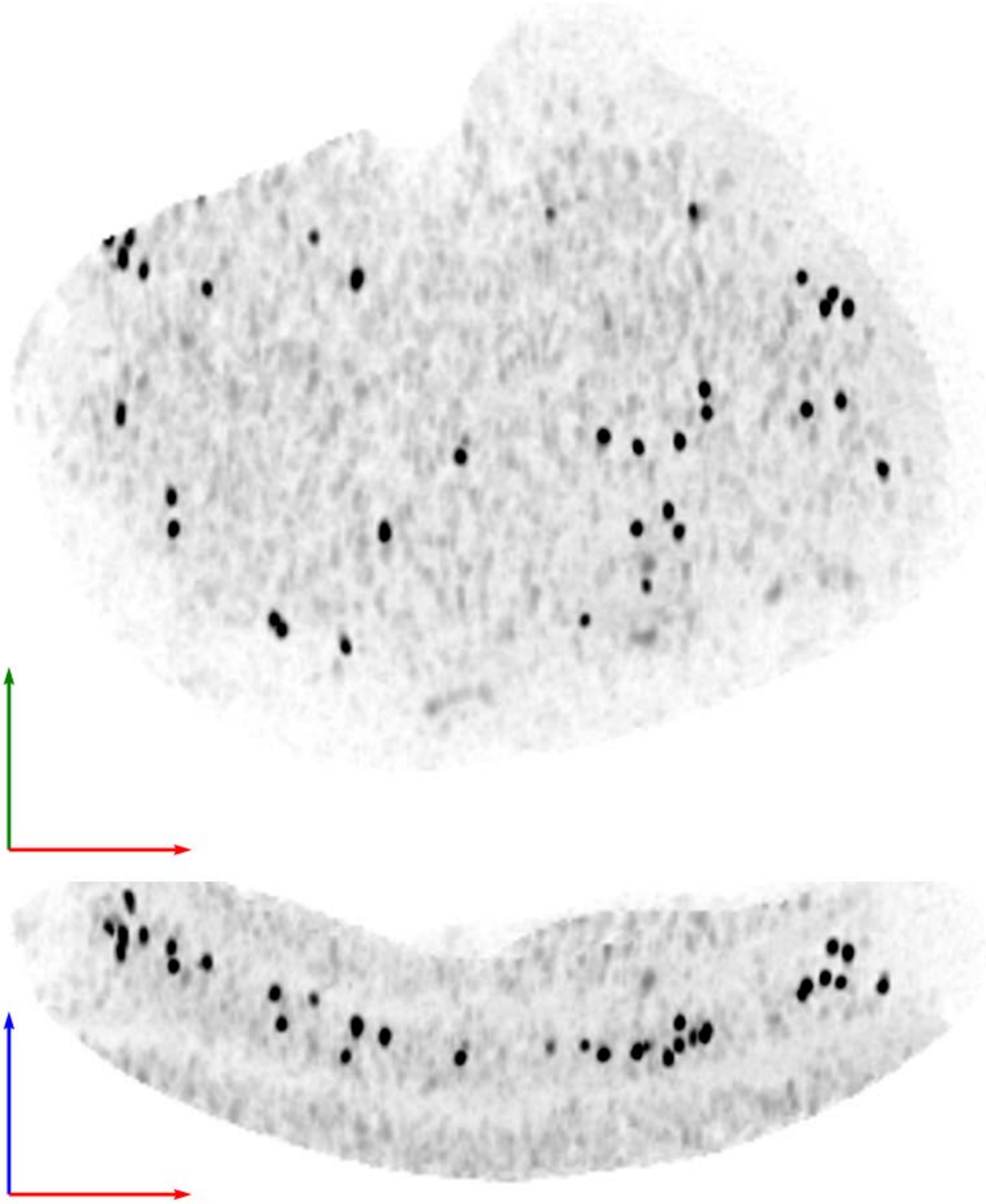

Tomogram ID: 20250206_AMmilled27-2_101	SET: anti-GluA1 11B8 "C-term"
Cleft ID: 0	Type: synaptosome

Denoised Tomogram Slice

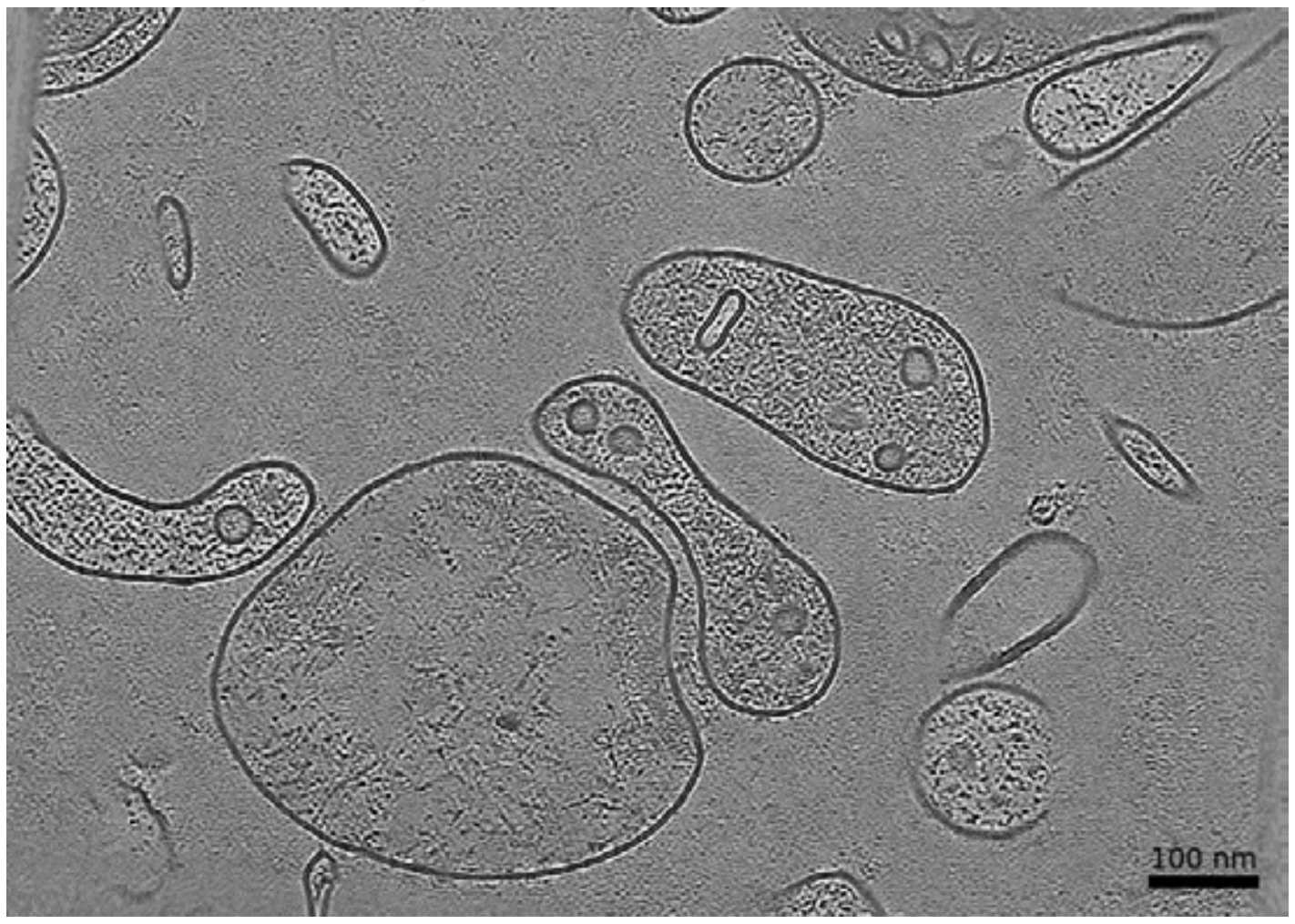

Membrane Segmentation & Cleft Position

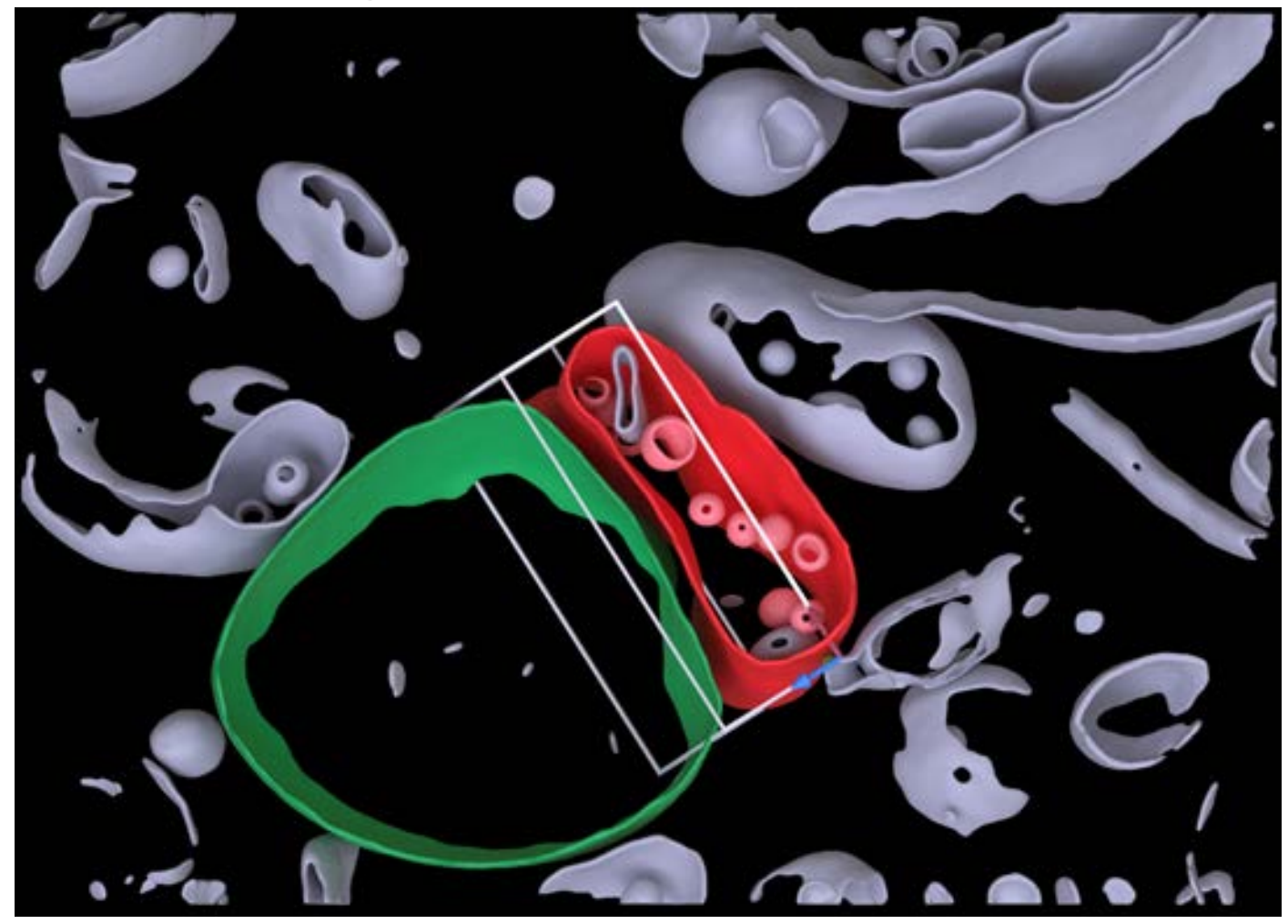

Cleft MIP

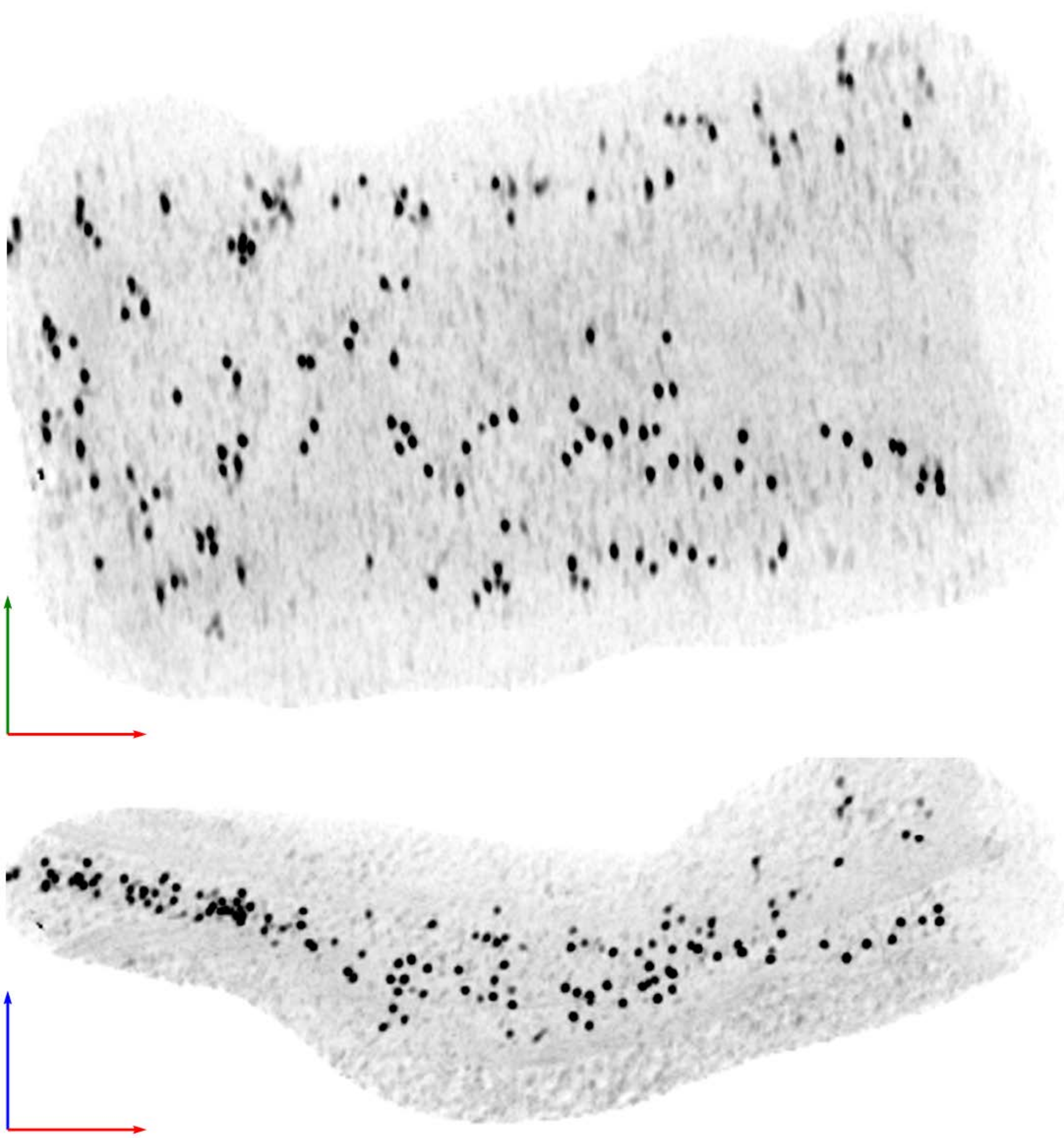

Tomogram ID: 20250206_AMmilled27-2_289	SET: anti-GluA1 11B8 "C-term"
Cleft ID: 0	Type: synaptosome

Denoised Tomogram Slice

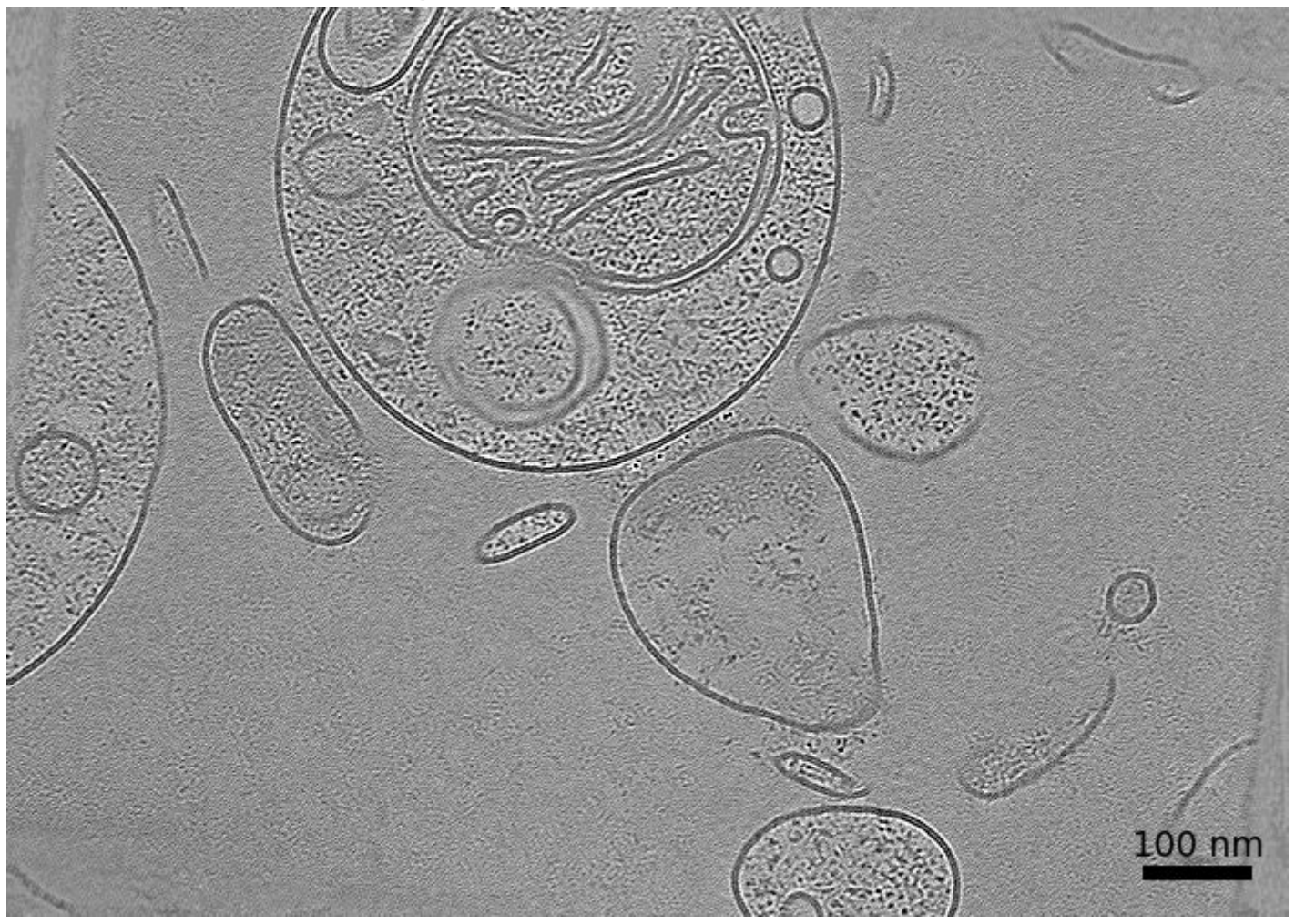

Membrane Segmentation & Cleft Position

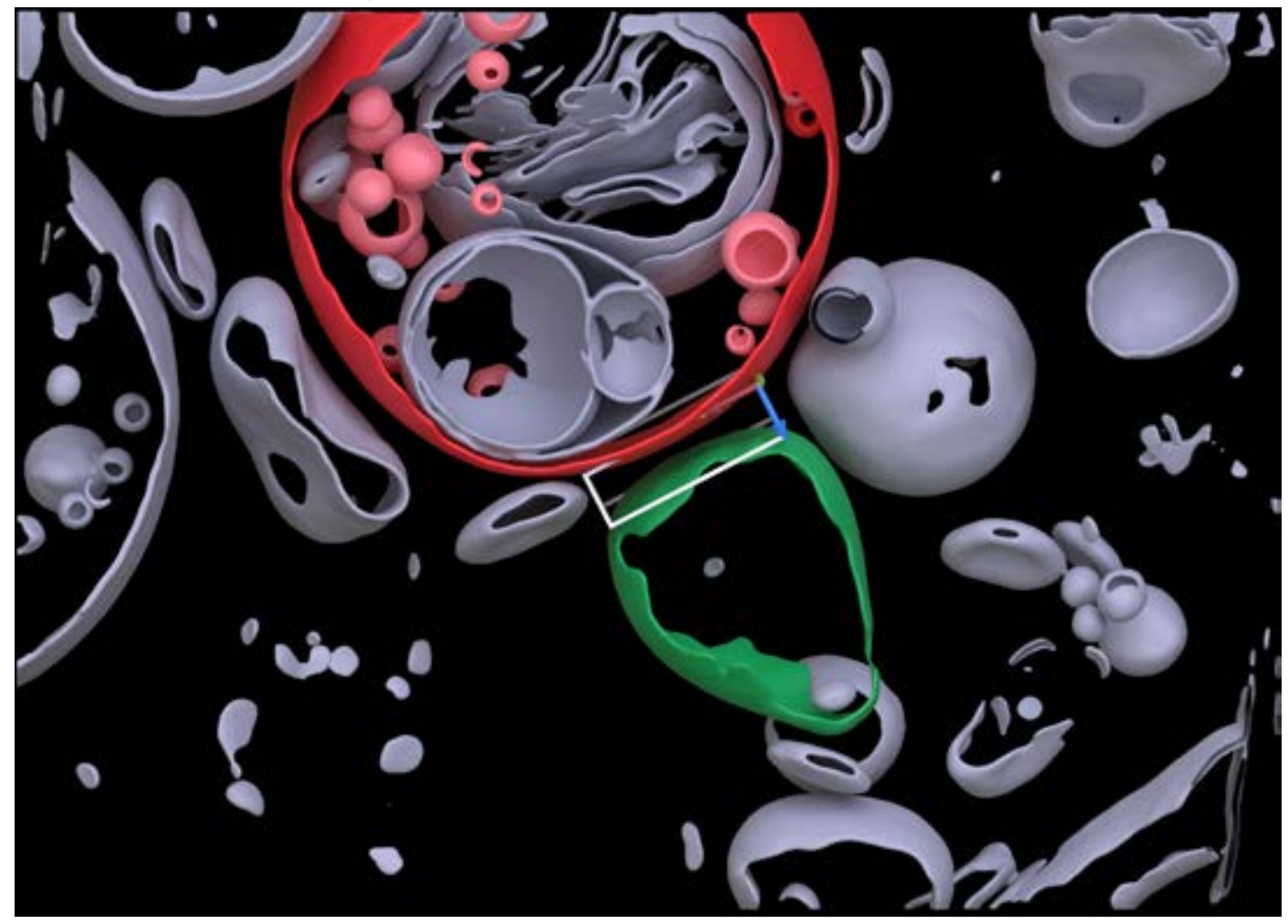

Cleft MIP

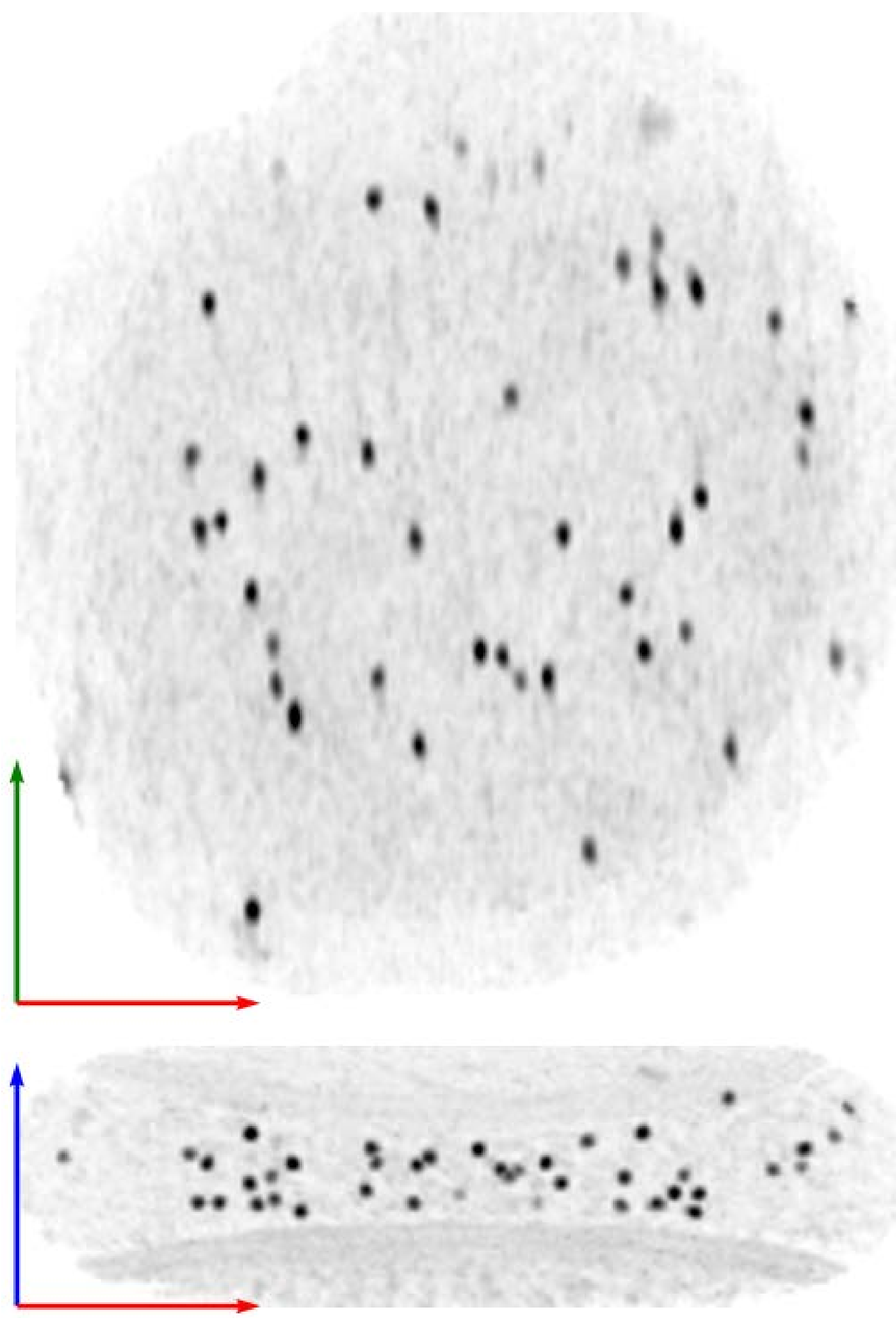

**Tomogram ID: 20240822_HippWaffle_125**	**SET: anti-GluN1 5F11 "C-term"**
Cleft ID: 0	Type: synaptosome

Denoised Tomogram Slice

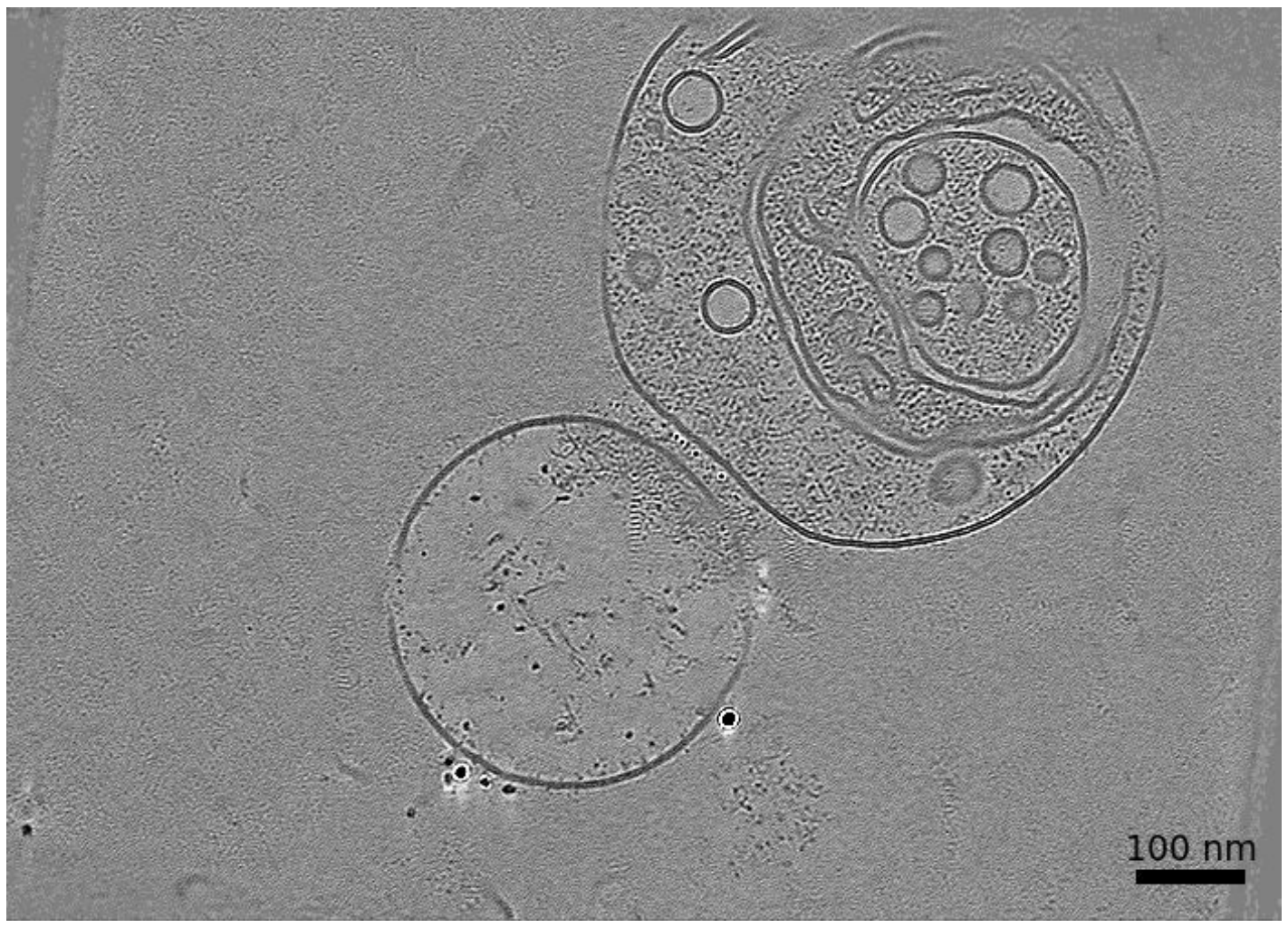

Membrane Segmentation & Cleft Position

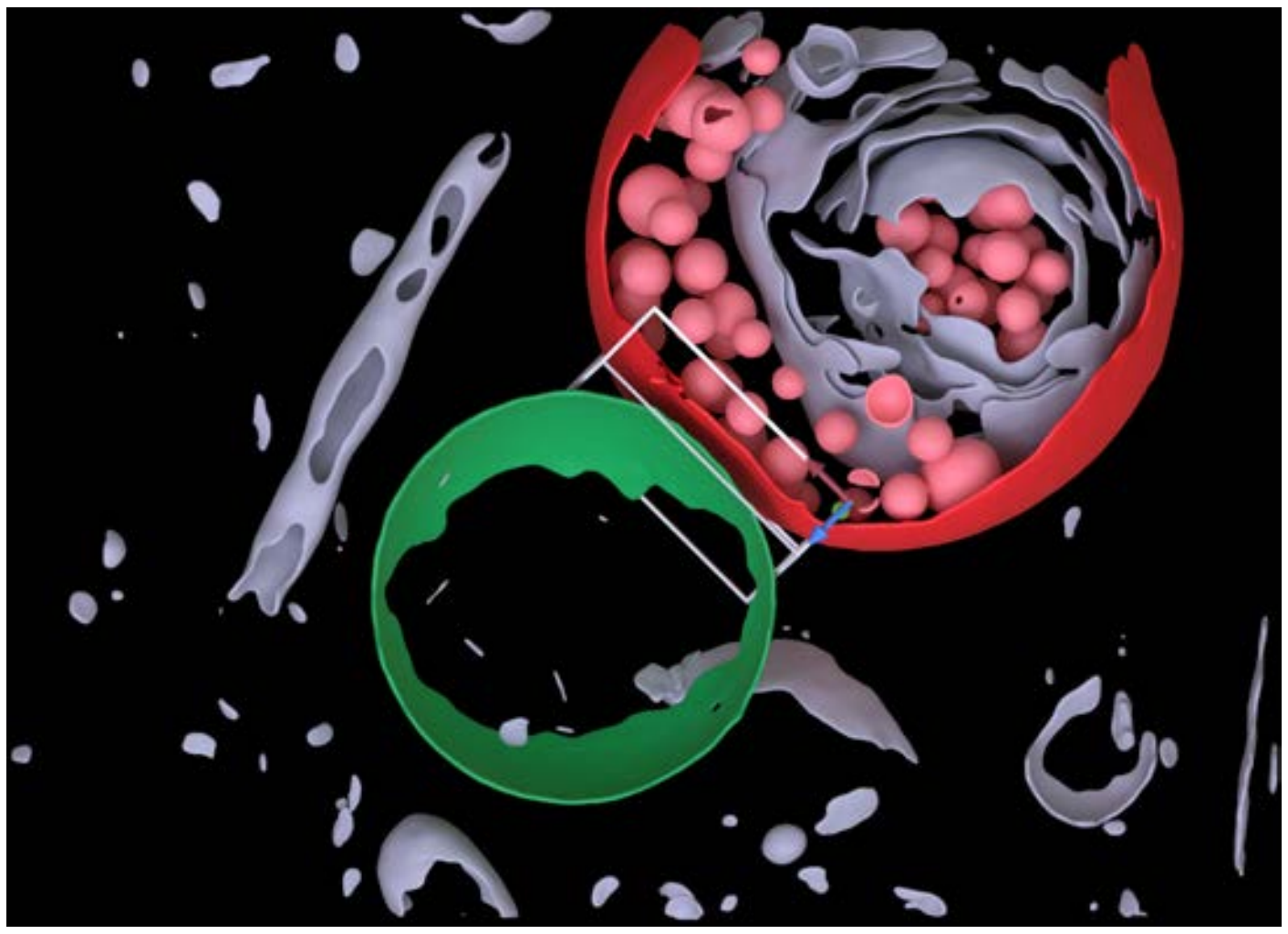

Cleft MIP

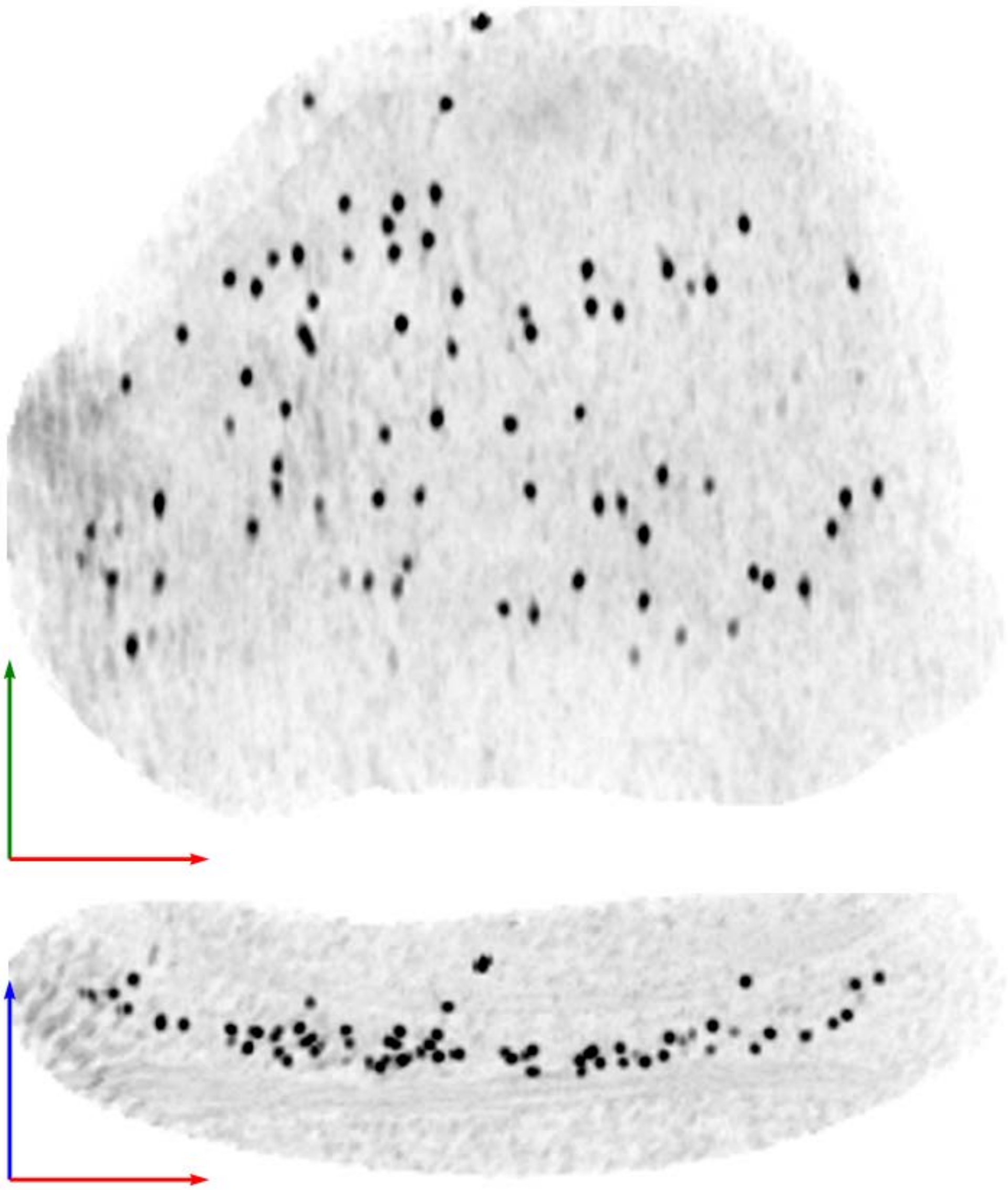

Tomogram ID: 20240822_HippWaffle_129	SET: anti-GluN1 5F11 "C-term"
Cleft ID: 0	Type: semi-tissue

Denoised Tomogram Slice

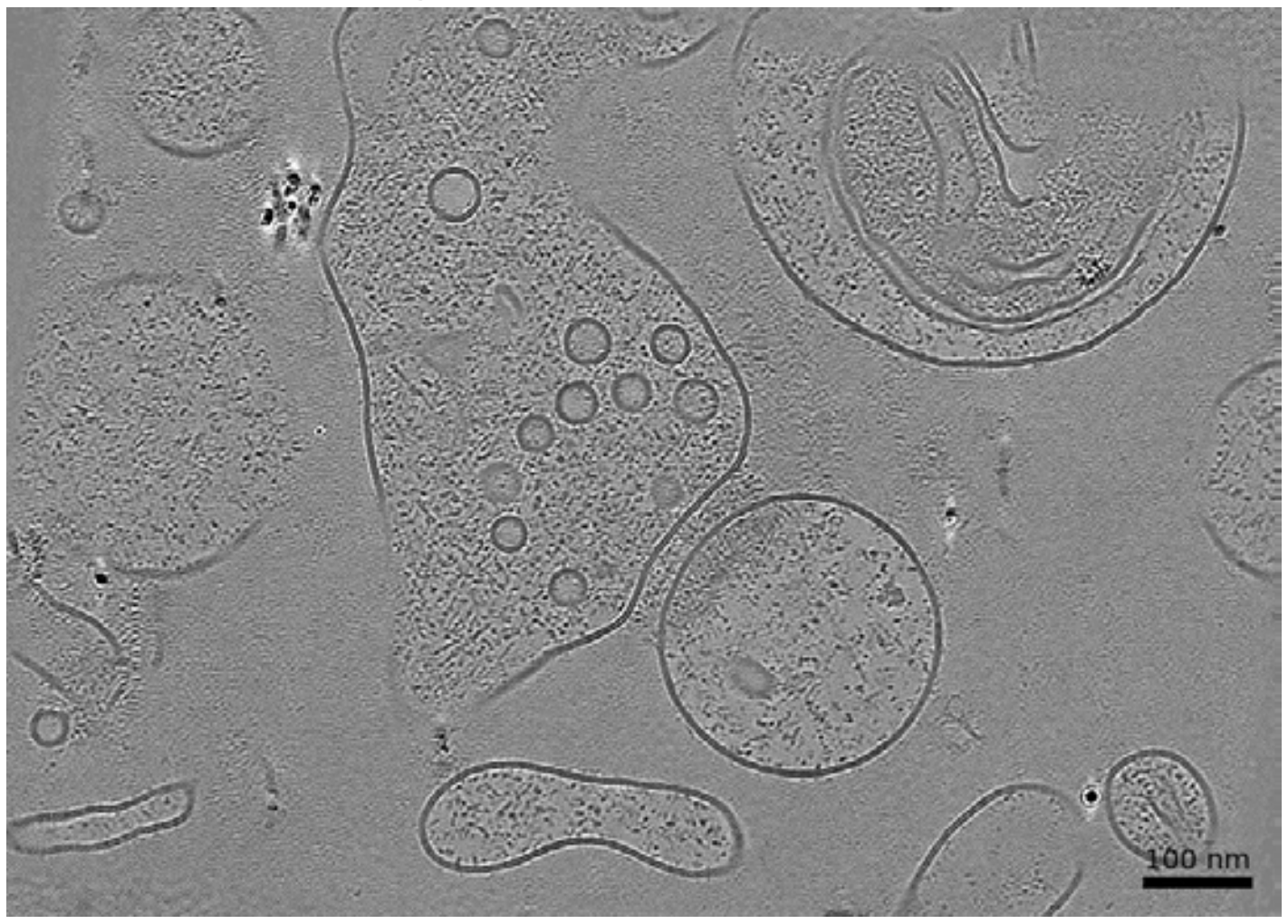

Membrane Segmentation & Cleft Position

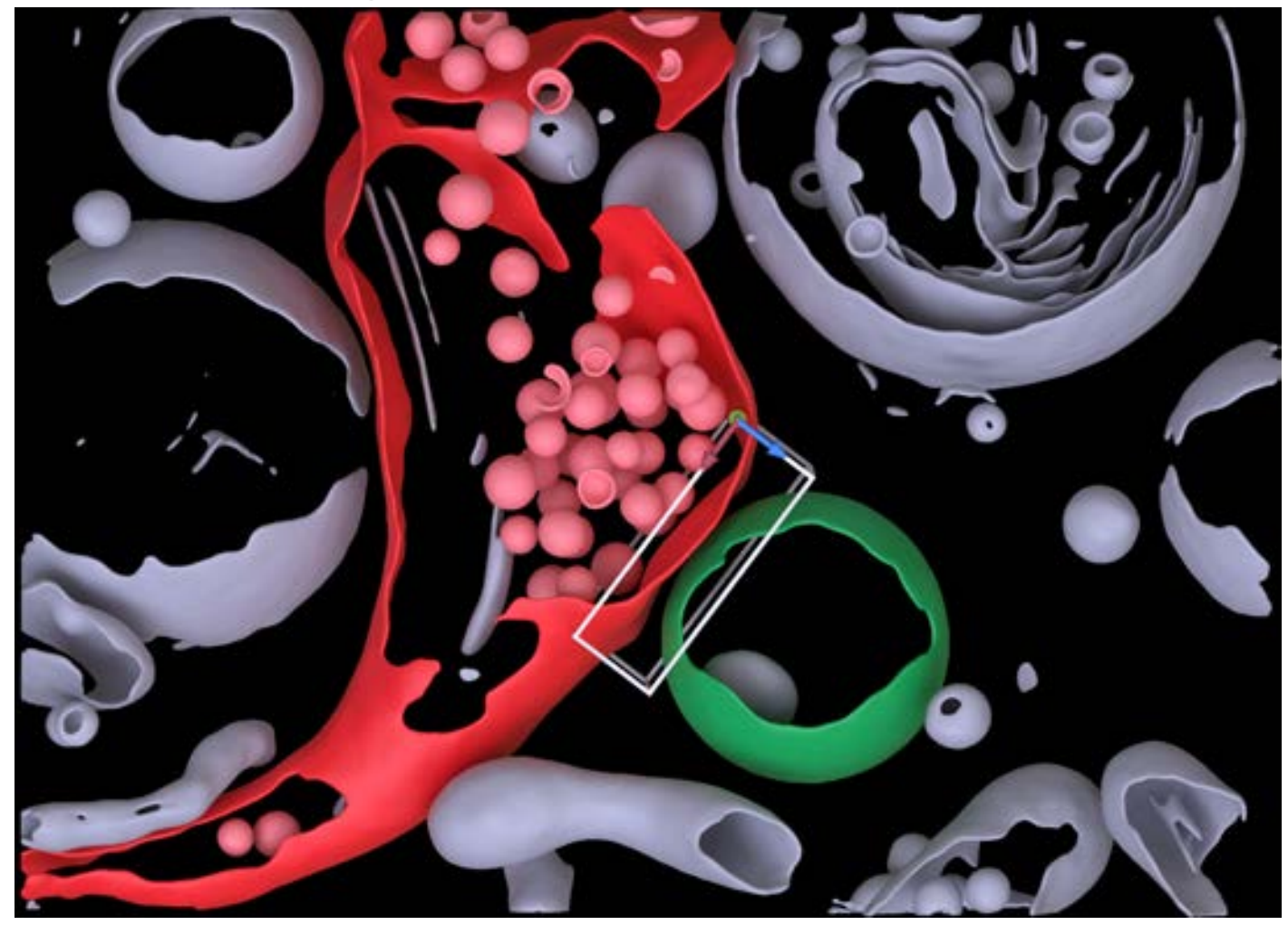

Cleft MIP

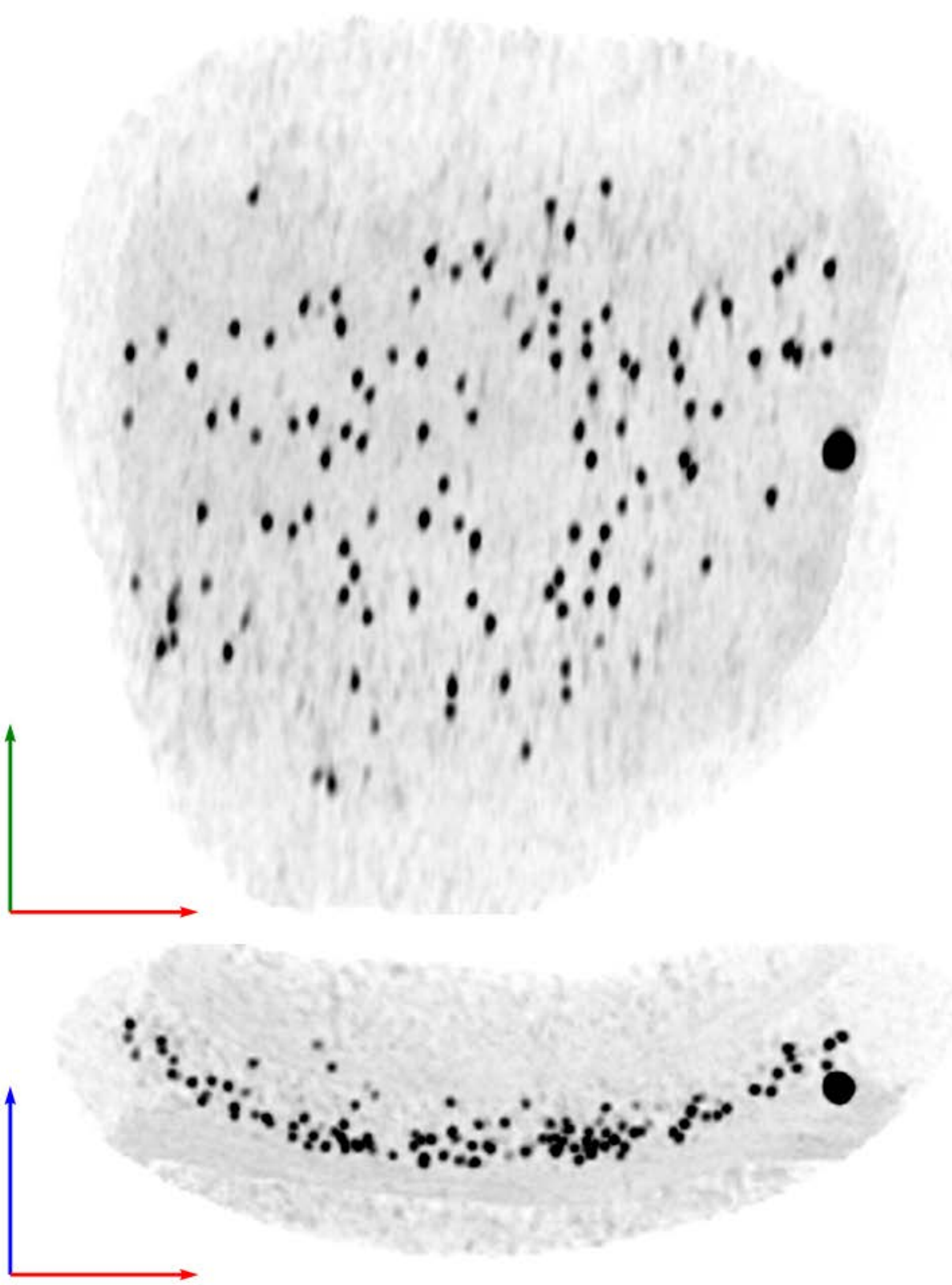

Tomogram ID: 20240822_HippWaffle_51 **SET: anti-GluN1 5F11 "C-term"**

Cleft ID: 0 Type: synaptosome

Denoised Tomogram Slice

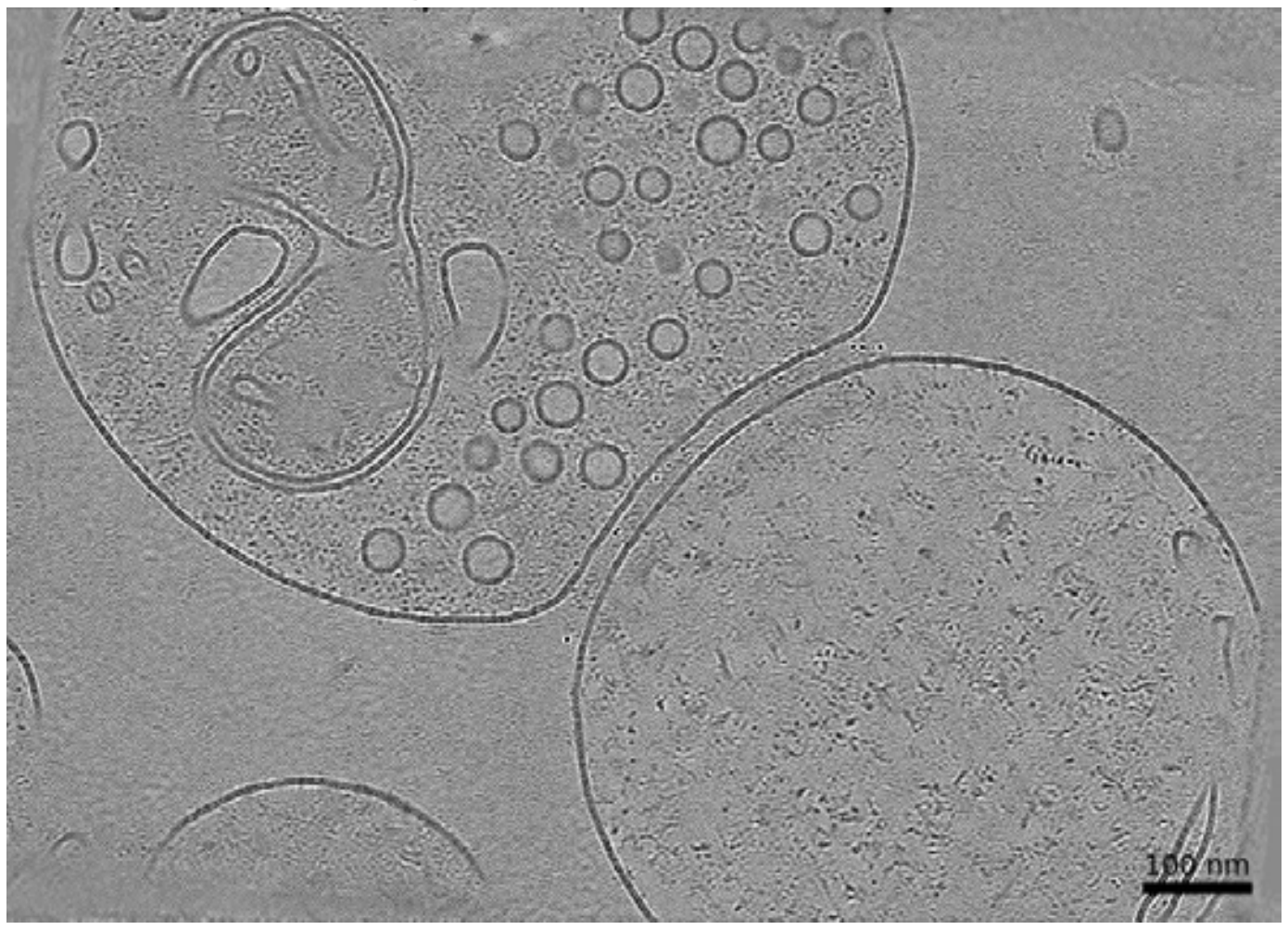

Membrane Segmentation & Cleft Position

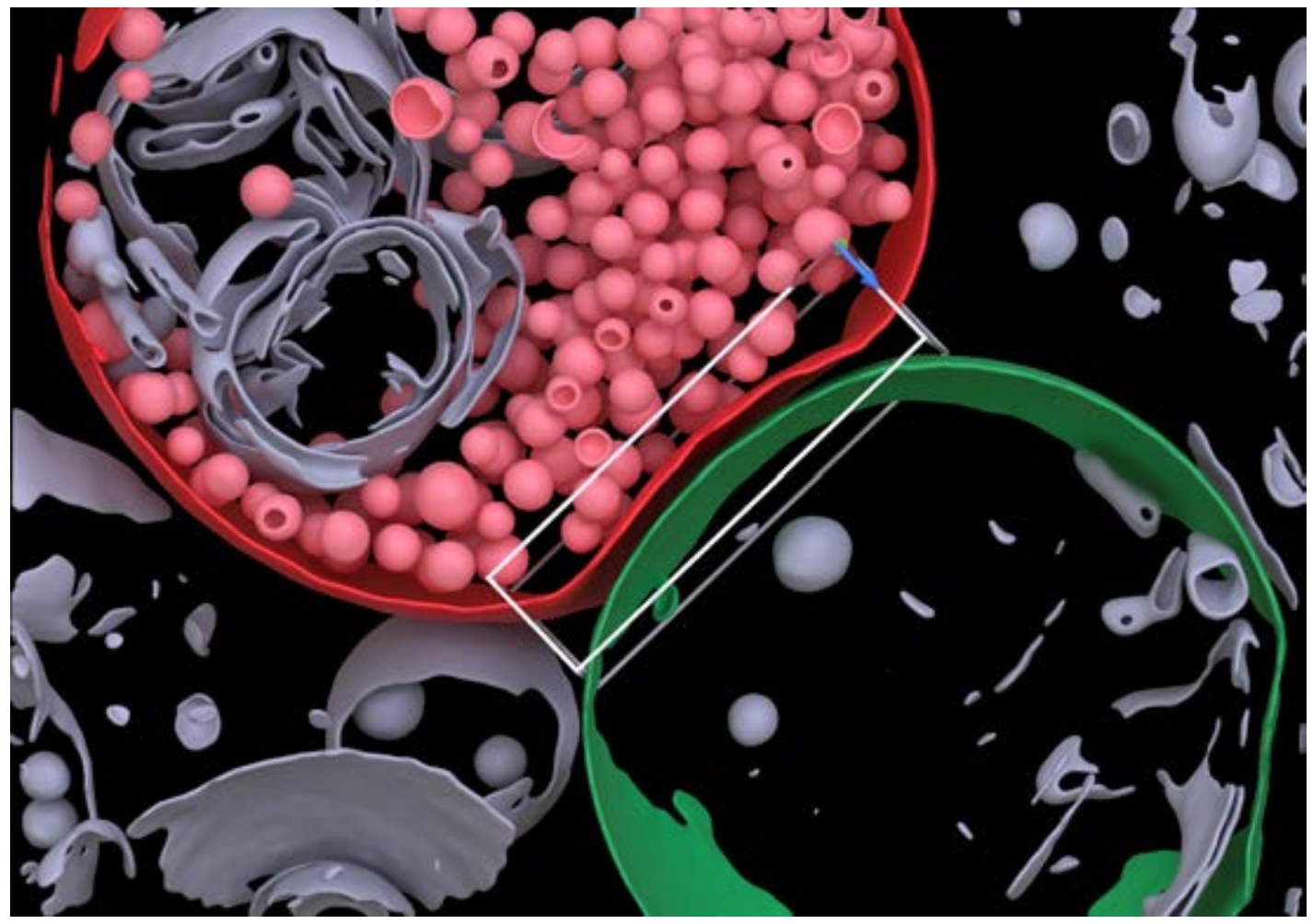

Cleft MIP

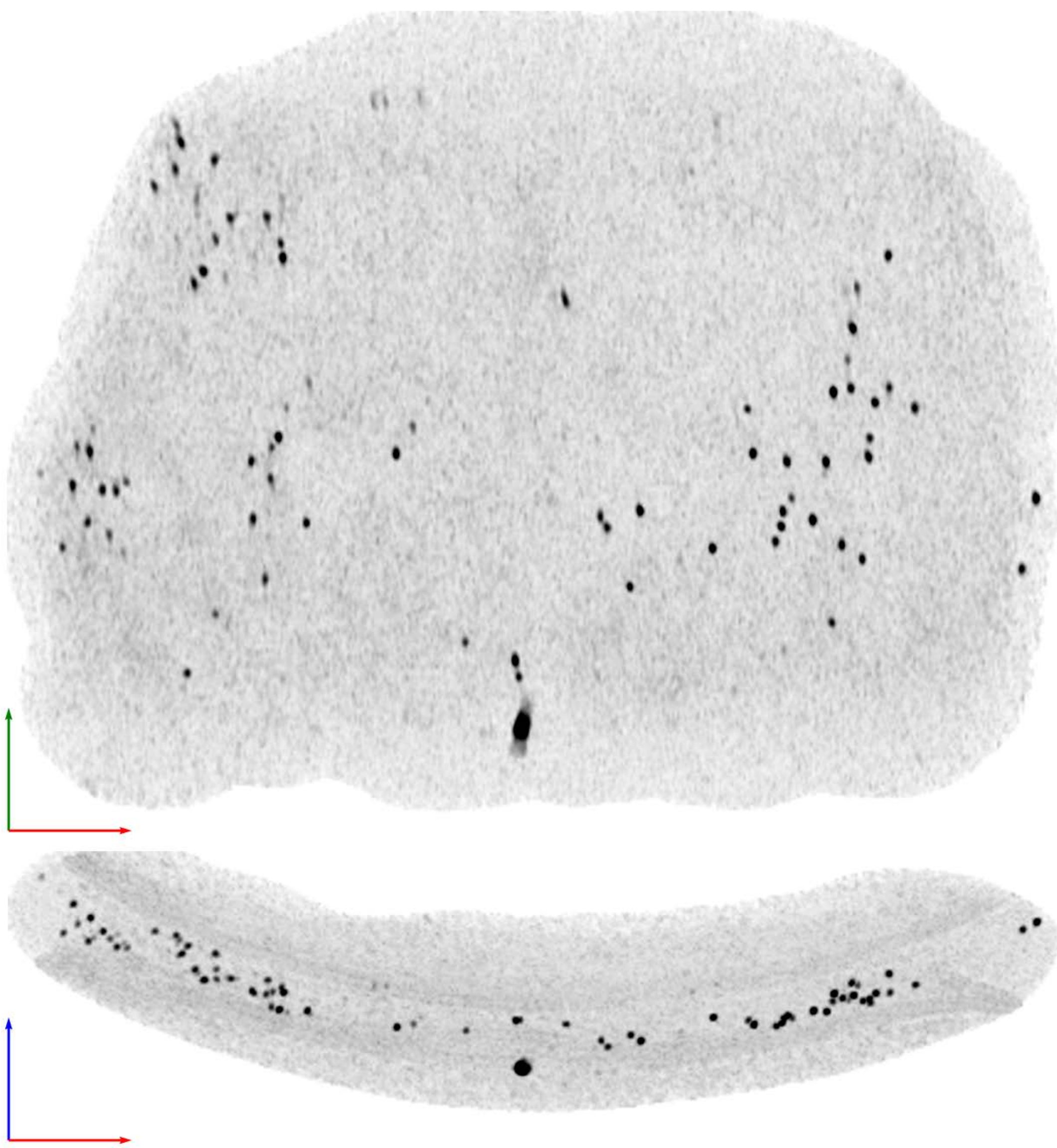

Tomogram ID: 20240920_HippWaffle_113 | **SET: anti-GluN1 5F11 "C-term"**

Cleft ID: 0 | Type: synaptosome

Denoised Tomogram Slice

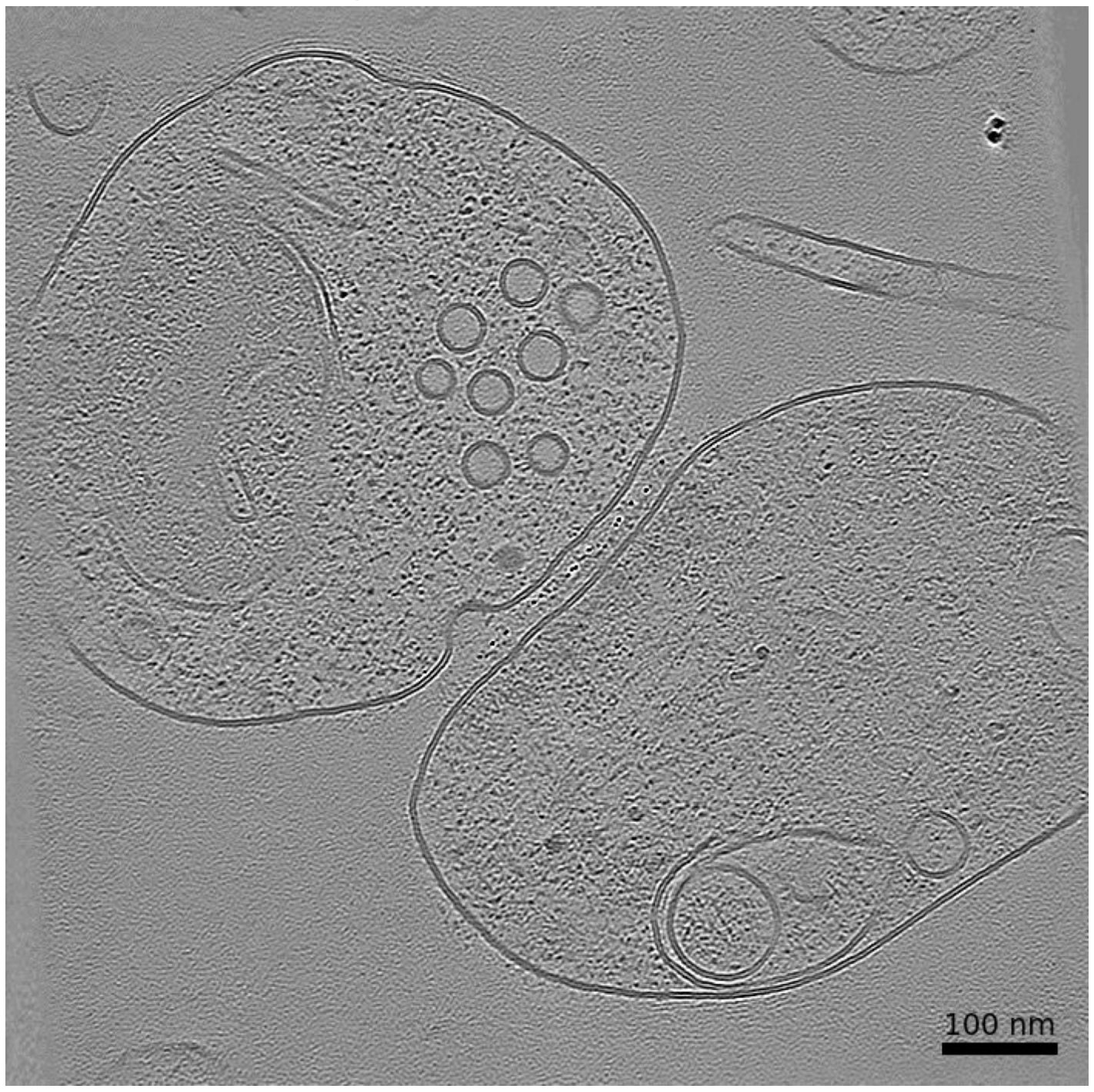

Membrane Segmentation & Cleft Position

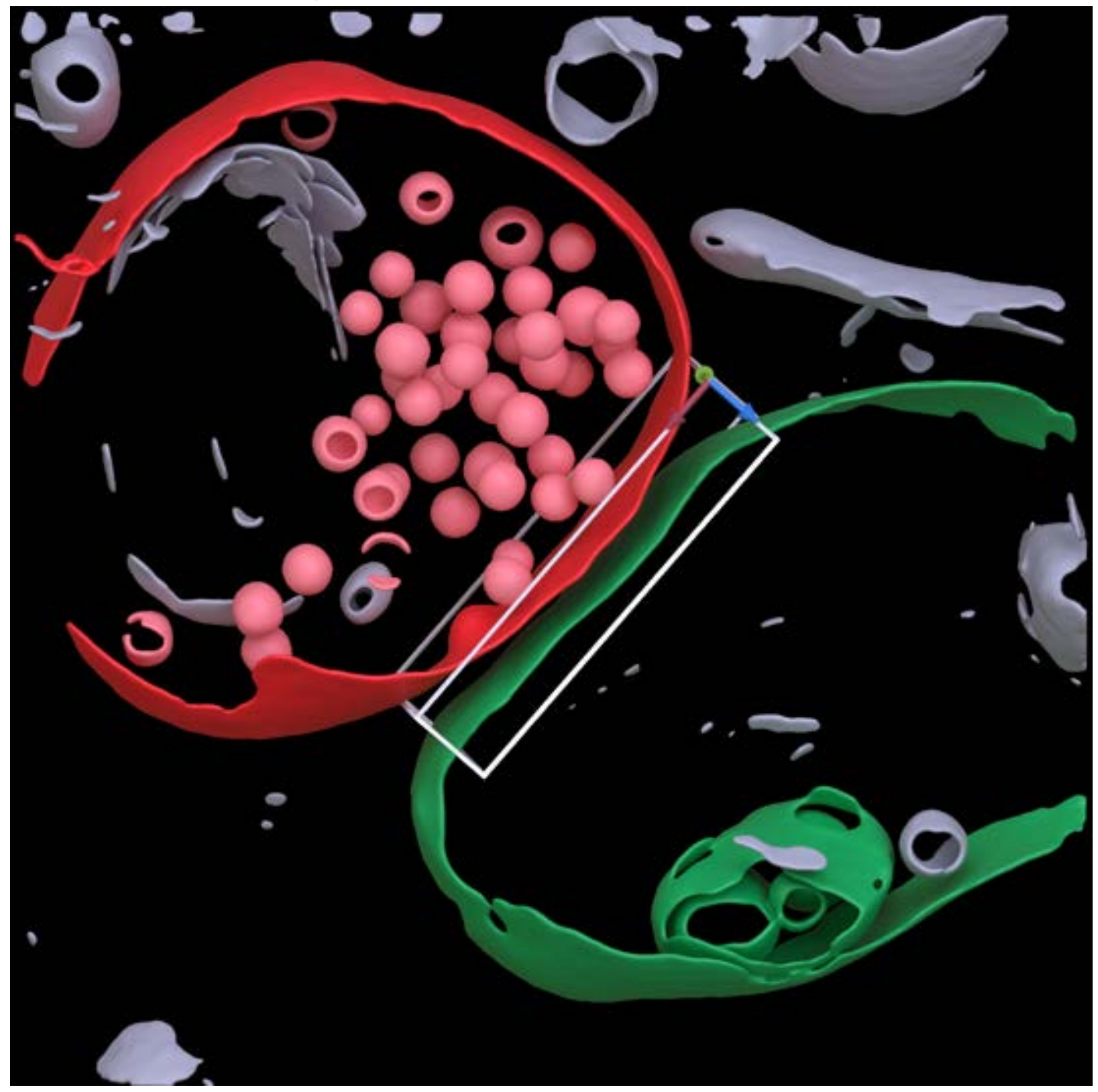

Cleft MIP

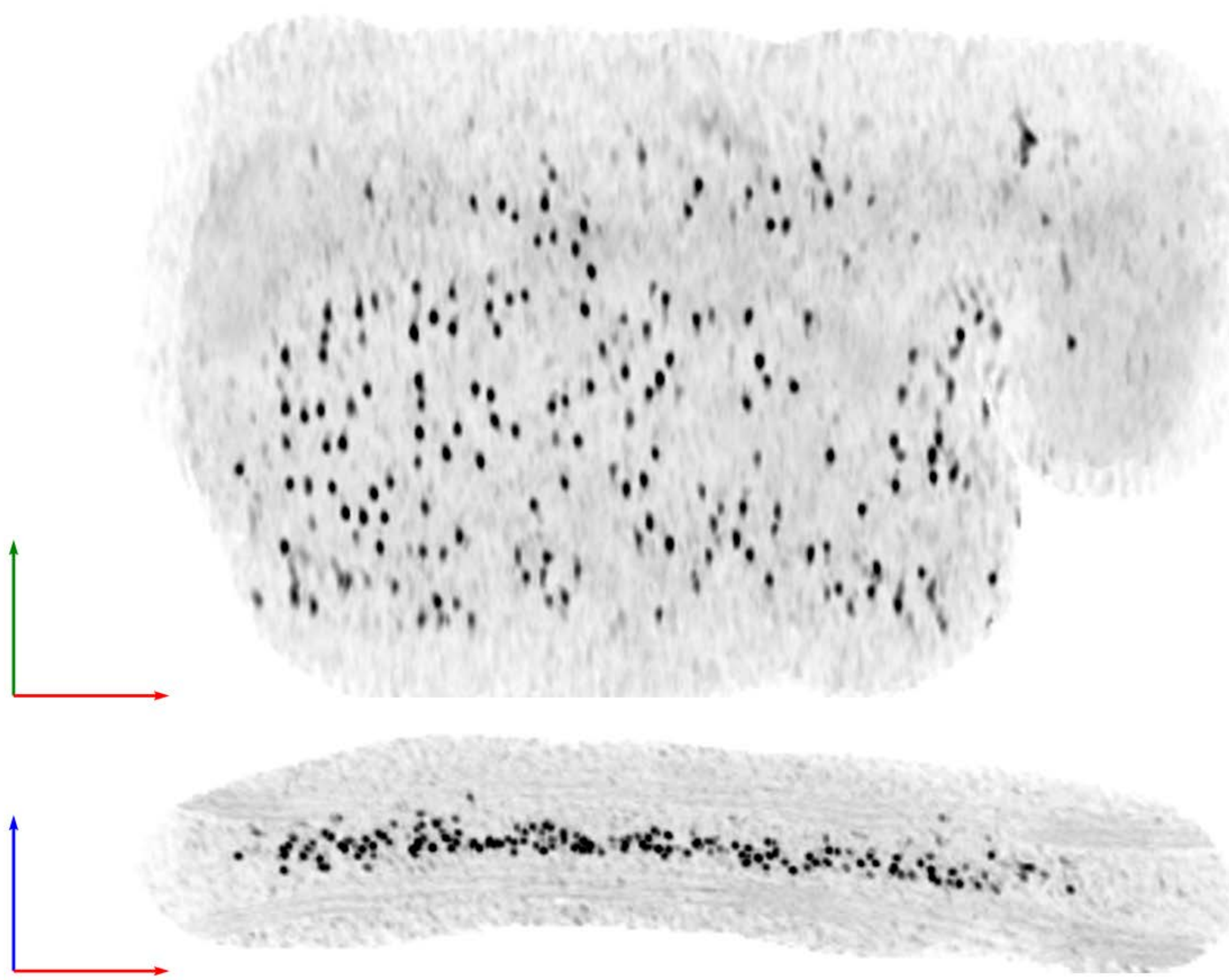

Tomogram ID: 20240920_HippWaffle_129 **SET: anti-GluN1 5F11 "C-term"**

Cleft ID: 0 Type: semi-tissue

Denoised Tomogram Slice

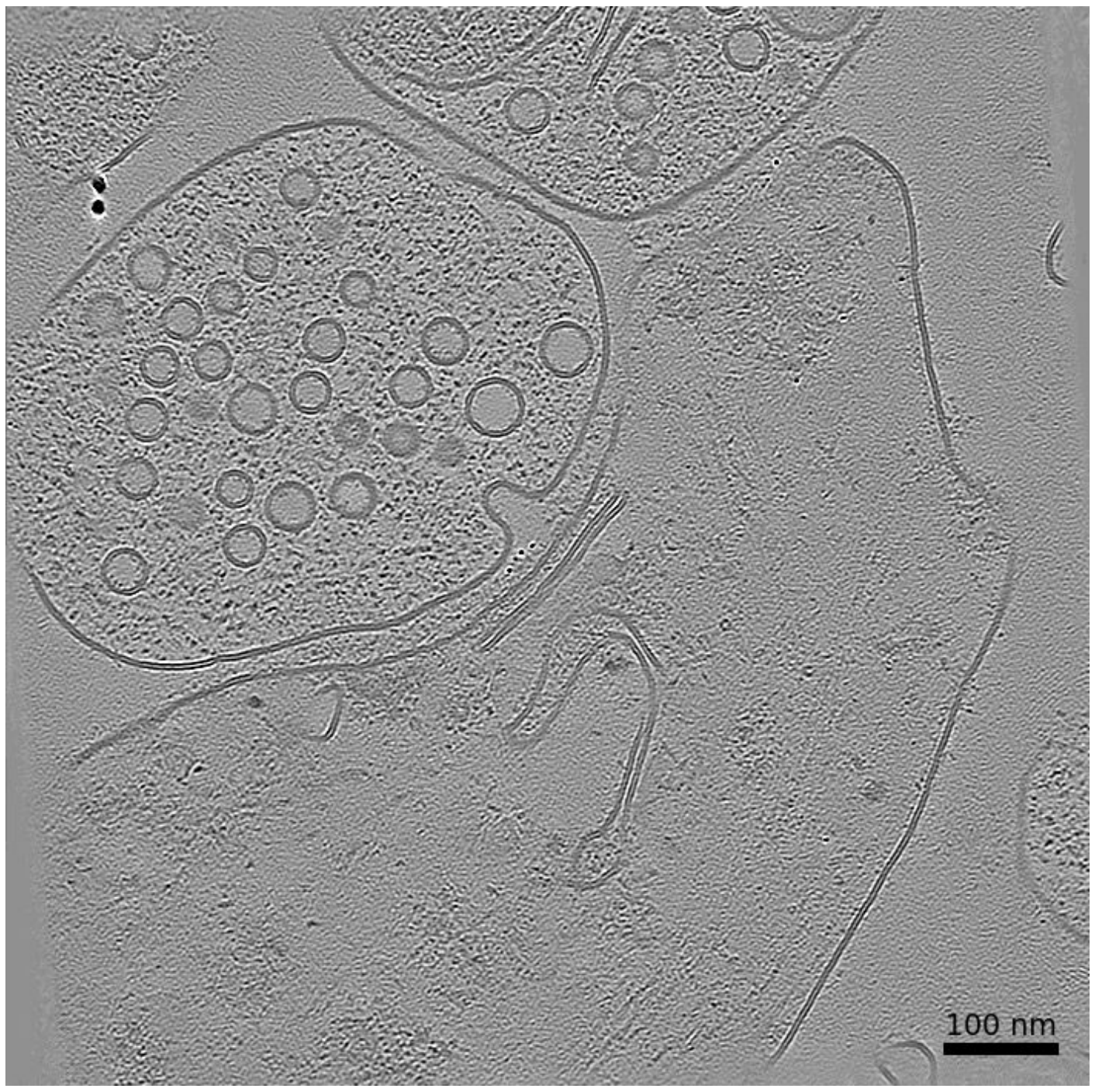

Membrane Segmentation & Cleft Position

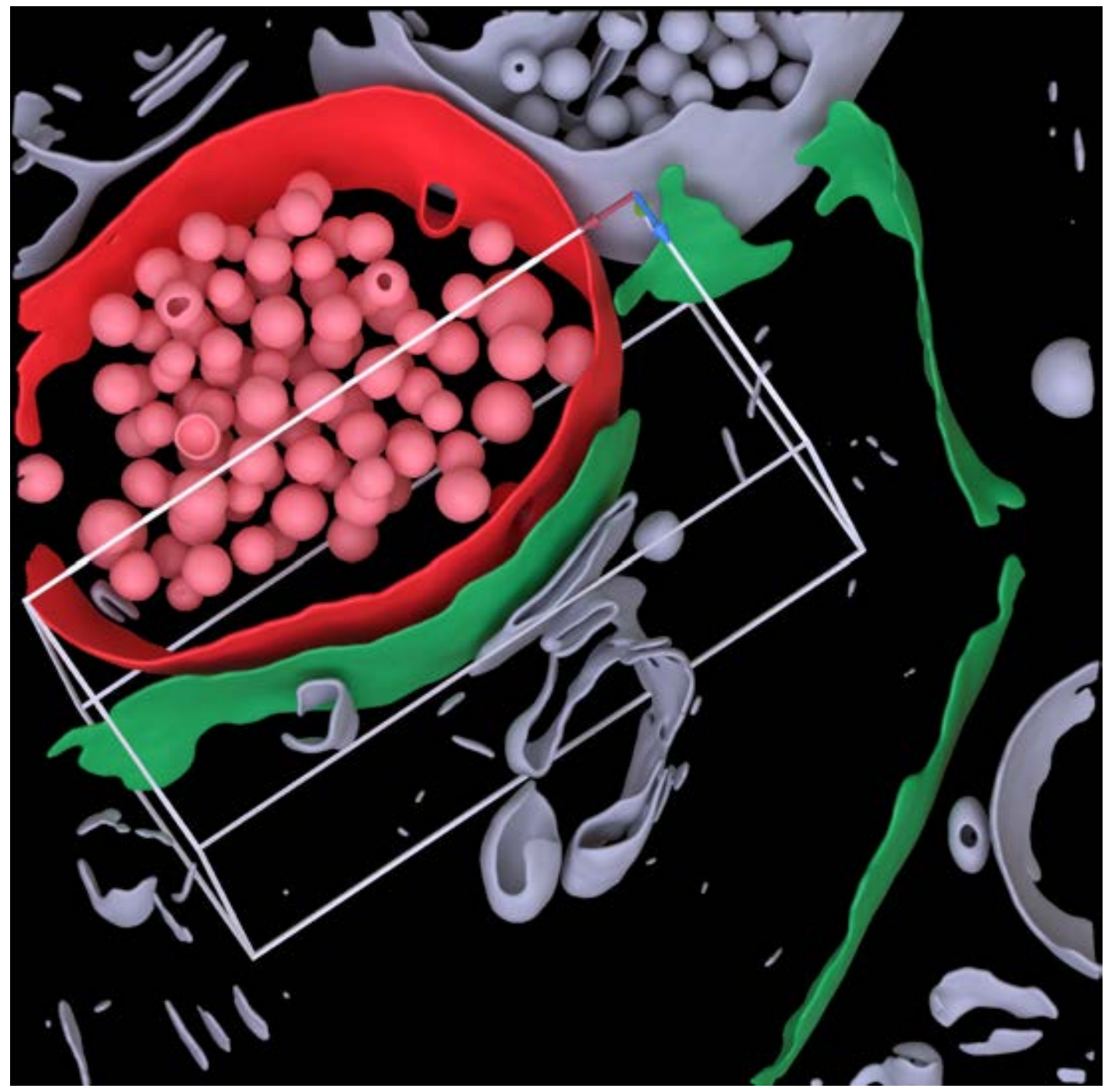

Cleft MIP

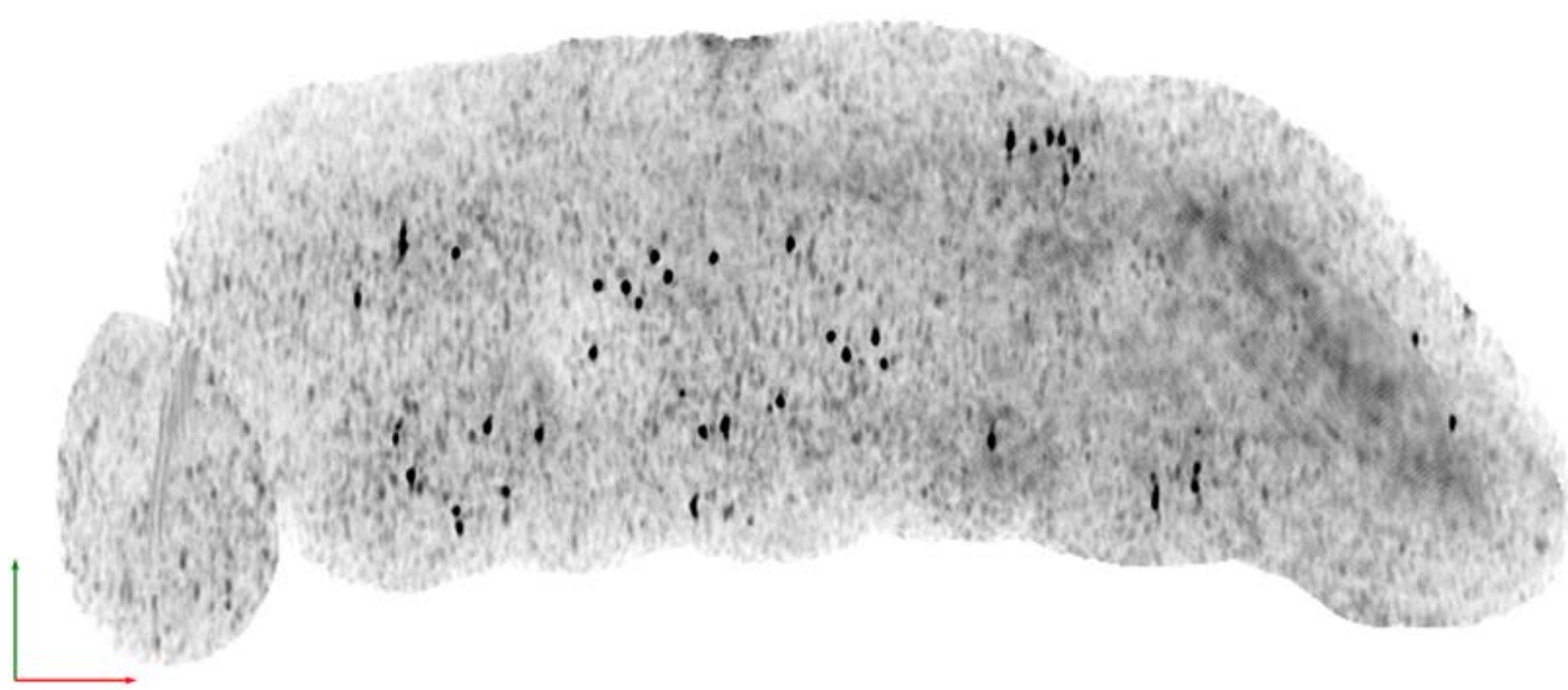

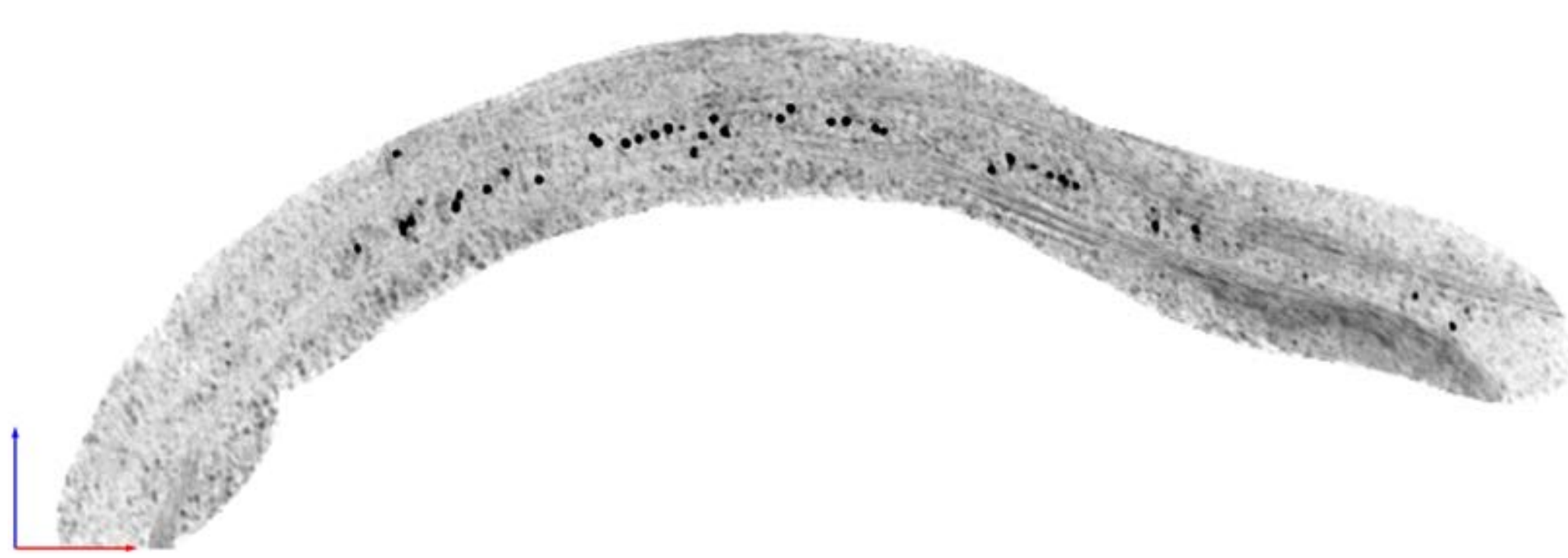

Tomogram ID: 20241206_AMmilled24-3_58	SET: Dual 15F1 monomer & 5F11 dimer
Cleft ID: 0	Type: semi-tissue

Denoised Tomogram Slice

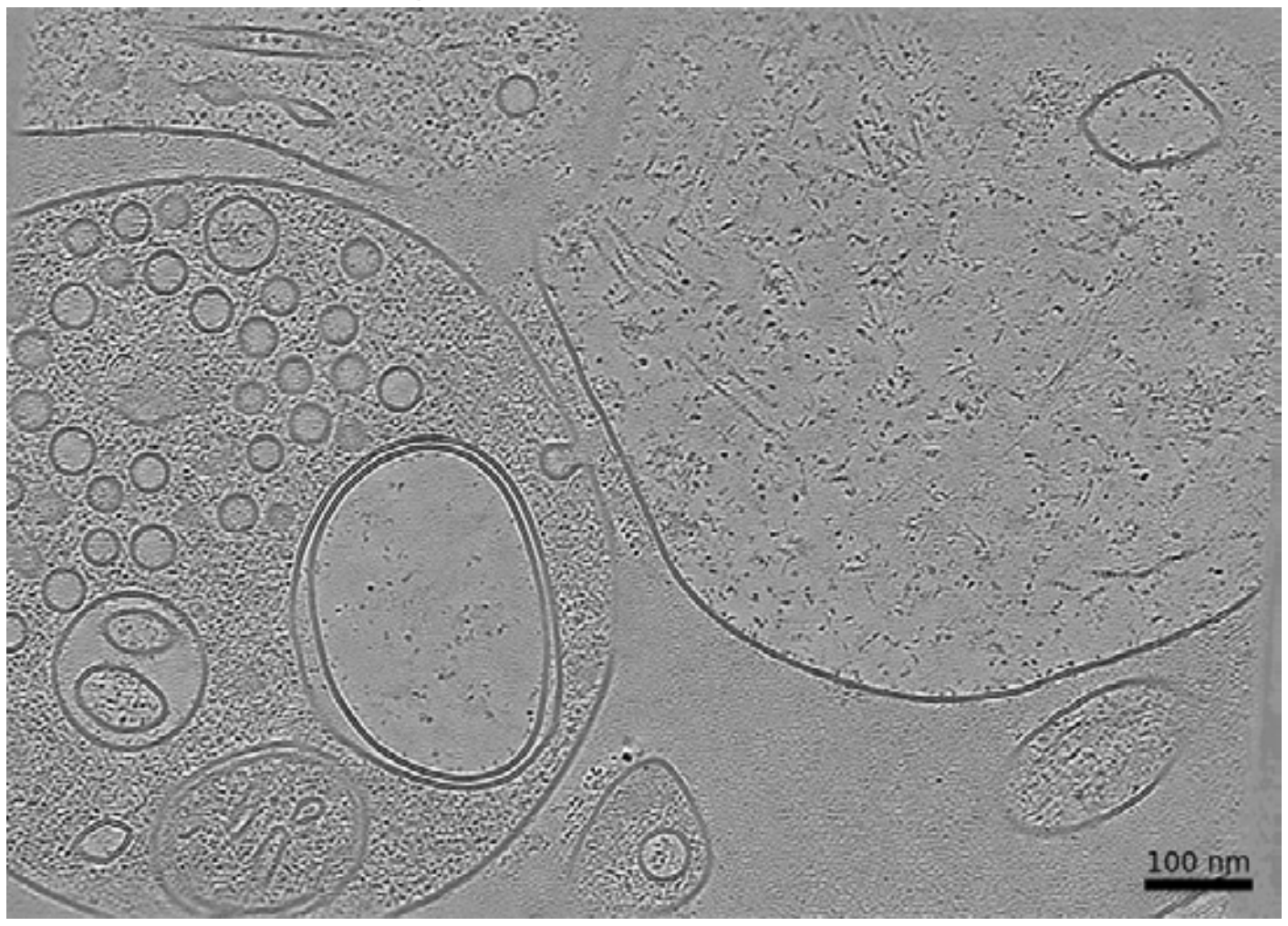

Membrane Segmentation & Cleft Position

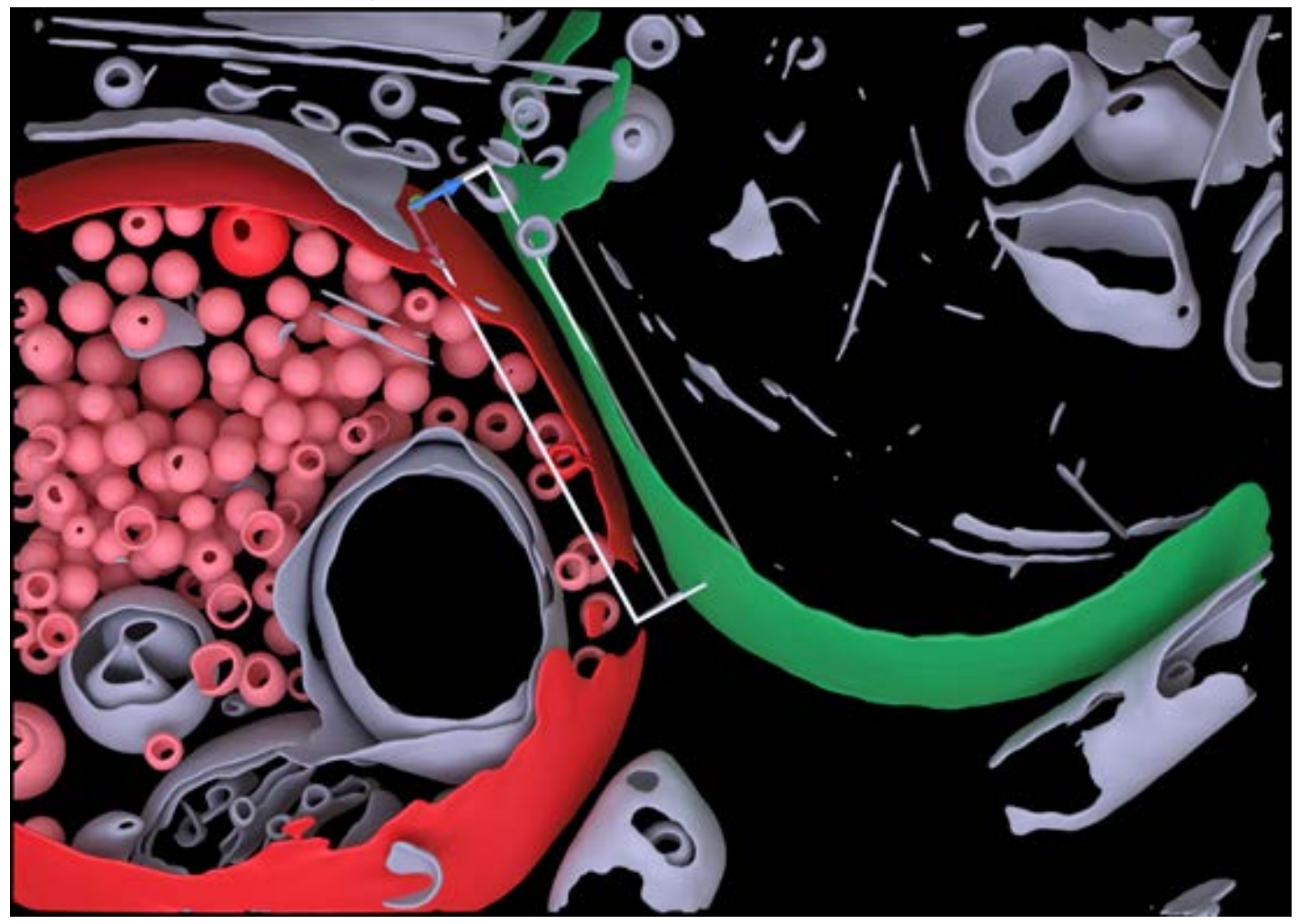

Cleft MIP

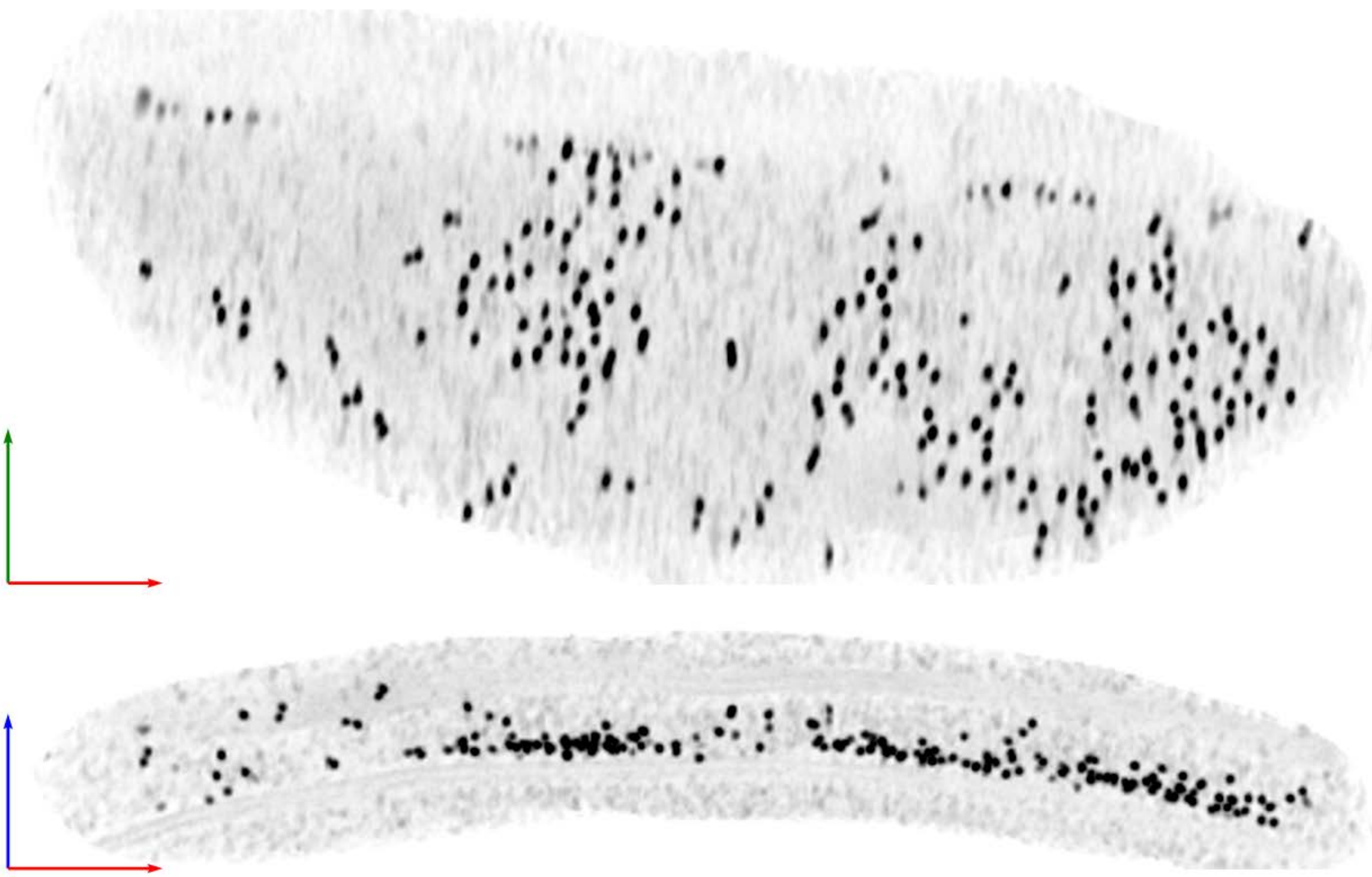

**Tomogram ID: 20241208_AMmilled24-1_64**	**SET: Dual 15F1 monomer & 5F11 dimer**
Cleft ID: 0	Type: tissue

Denoised Tomogram Slice

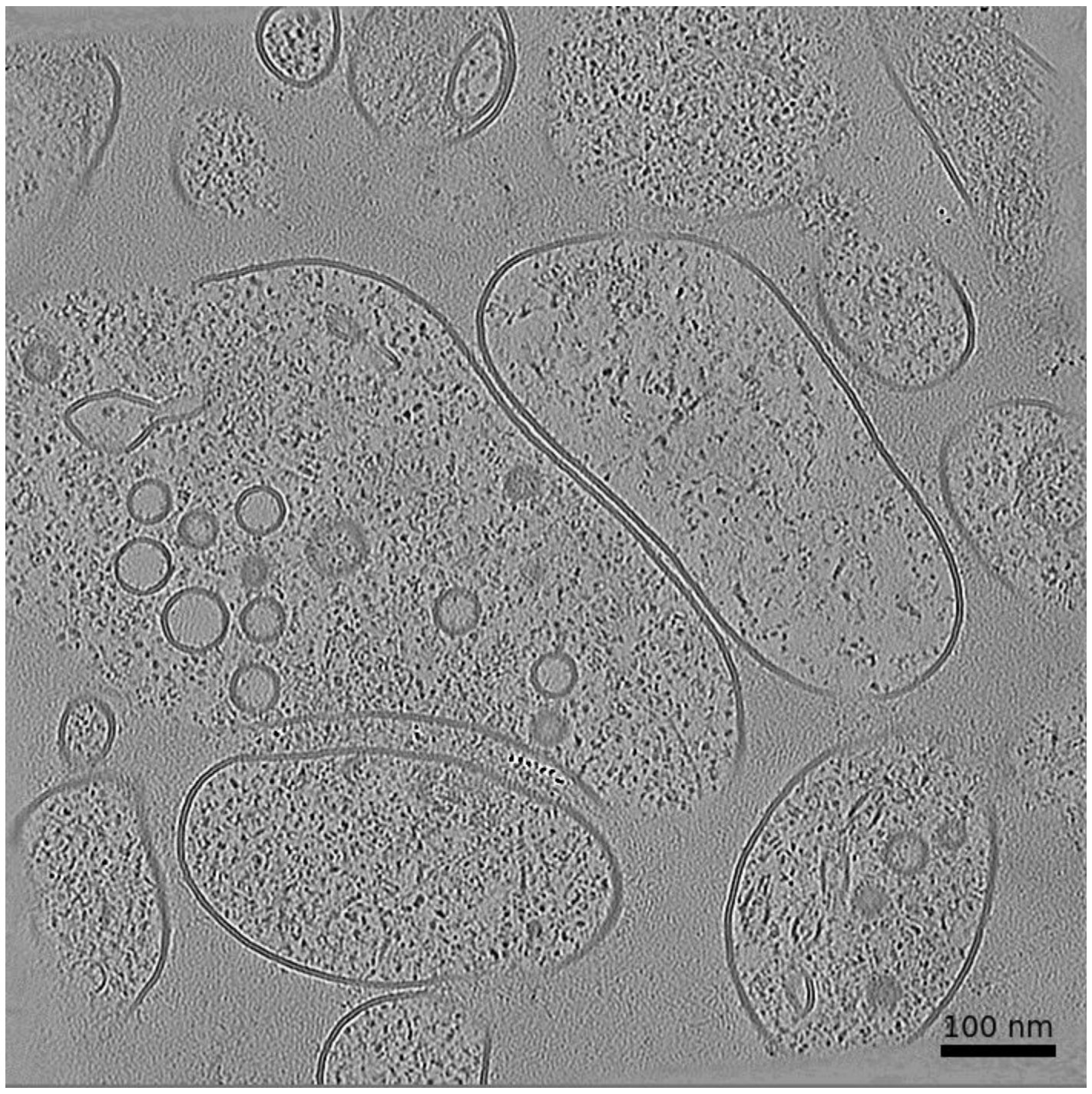

Membrane Segmentation & Cleft Position

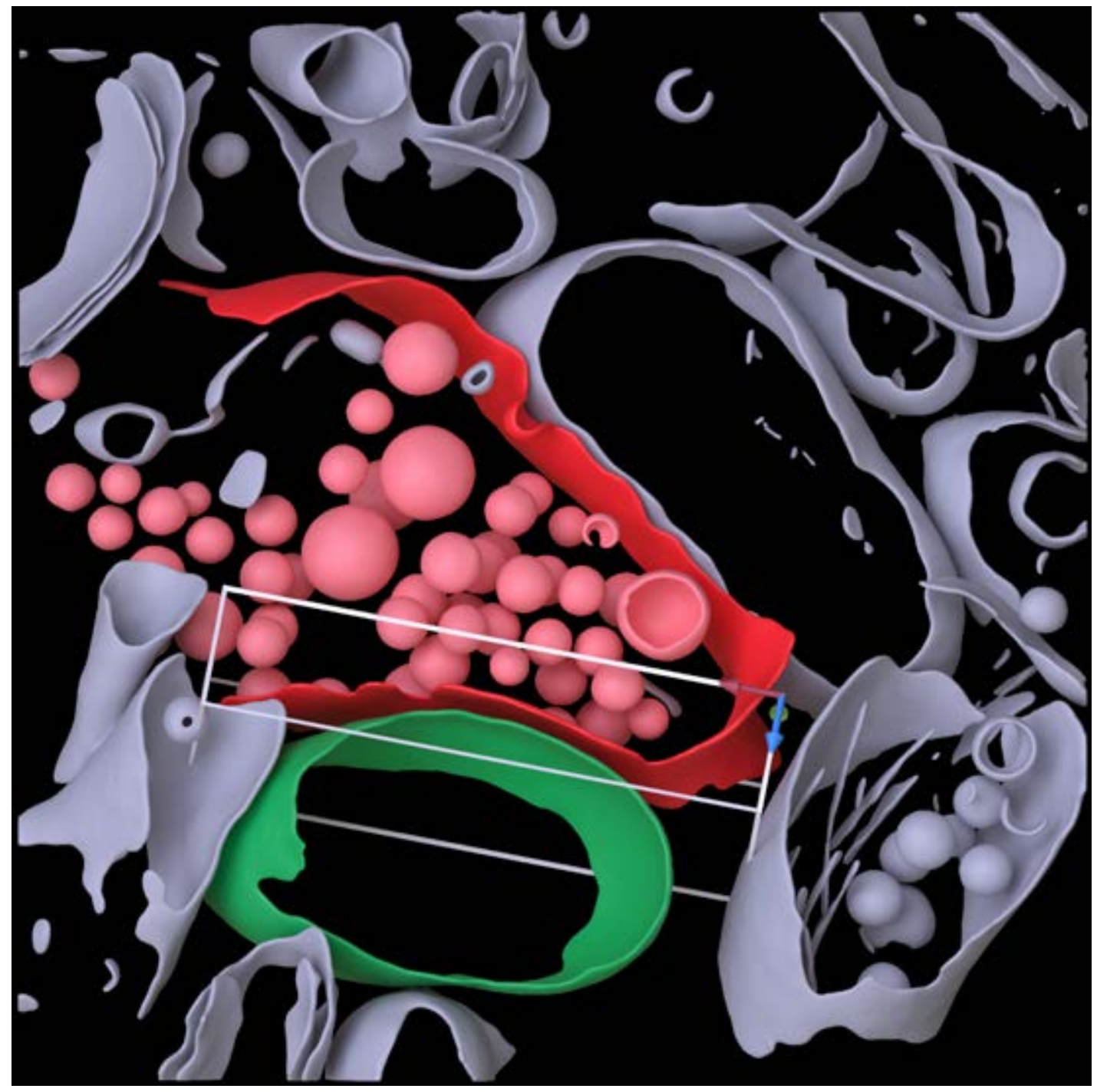

Cleft MIP

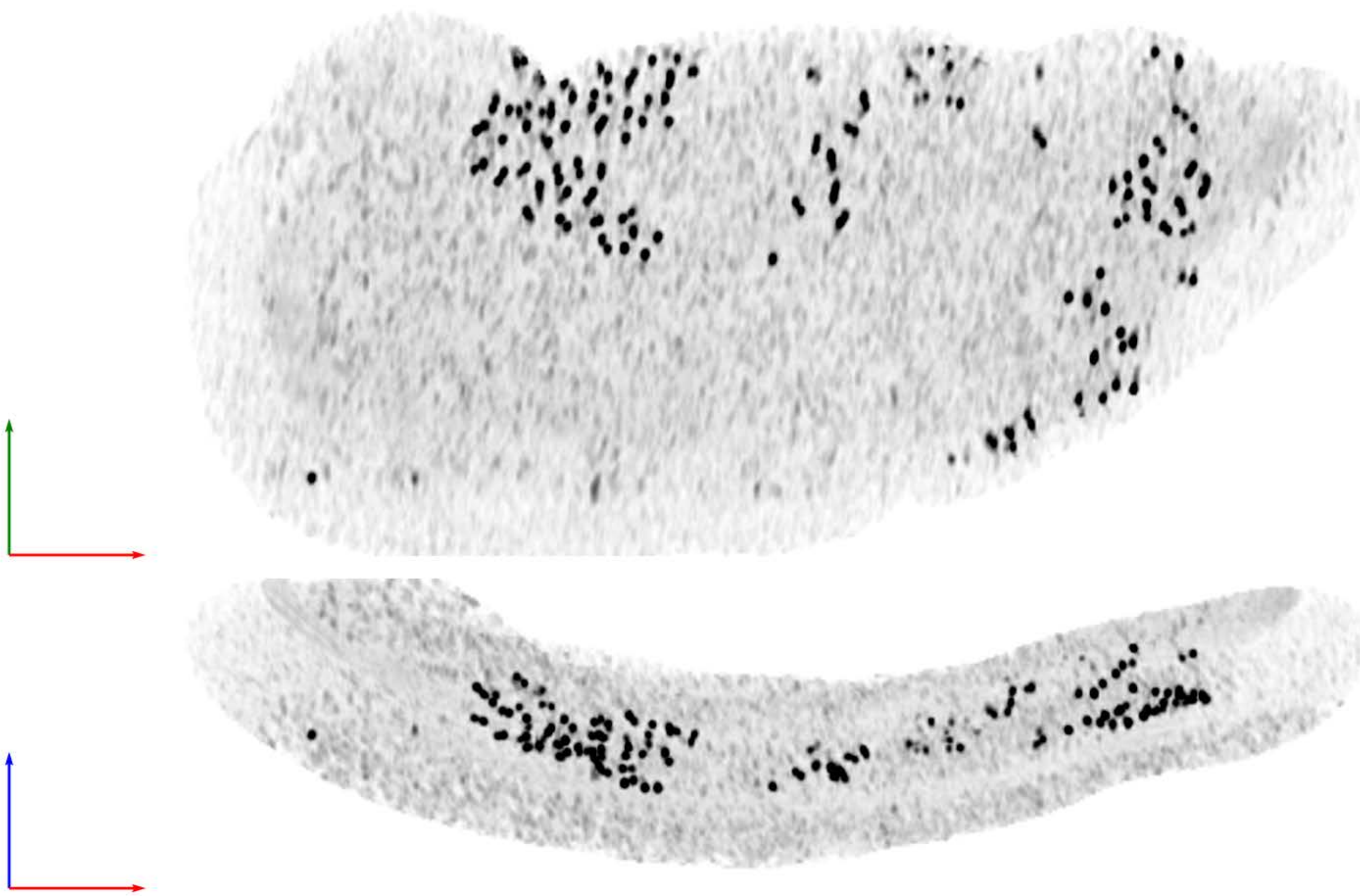

Tomogram ID: 20241208_AMmilled24-1_65	SET: Dual 15F1 monomer & 5F11 dimer
Cleft ID: 0	Type: semi-tissue

Denoised Tomogram Slice

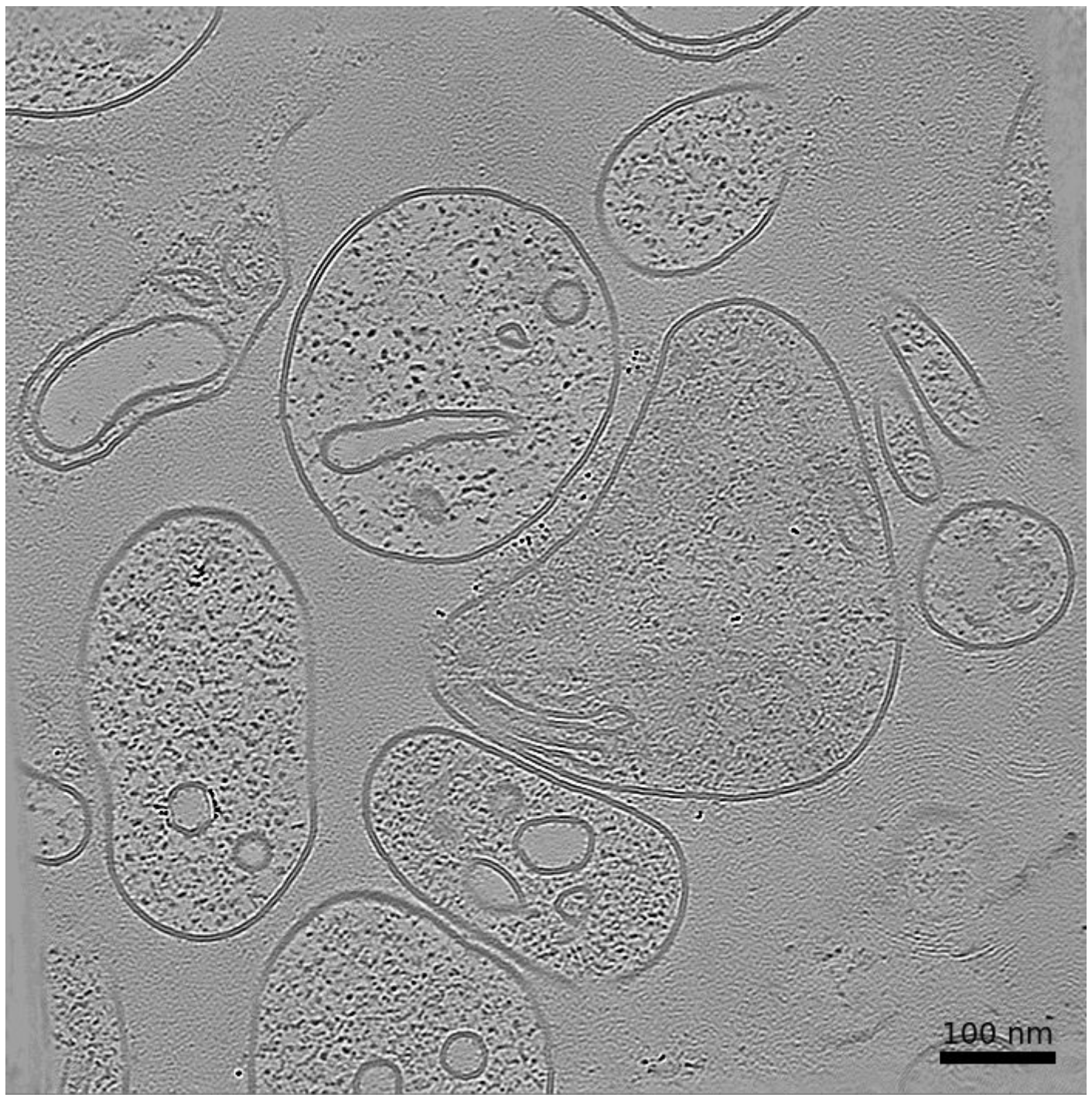

Membrane Segmentation & Cleft Position

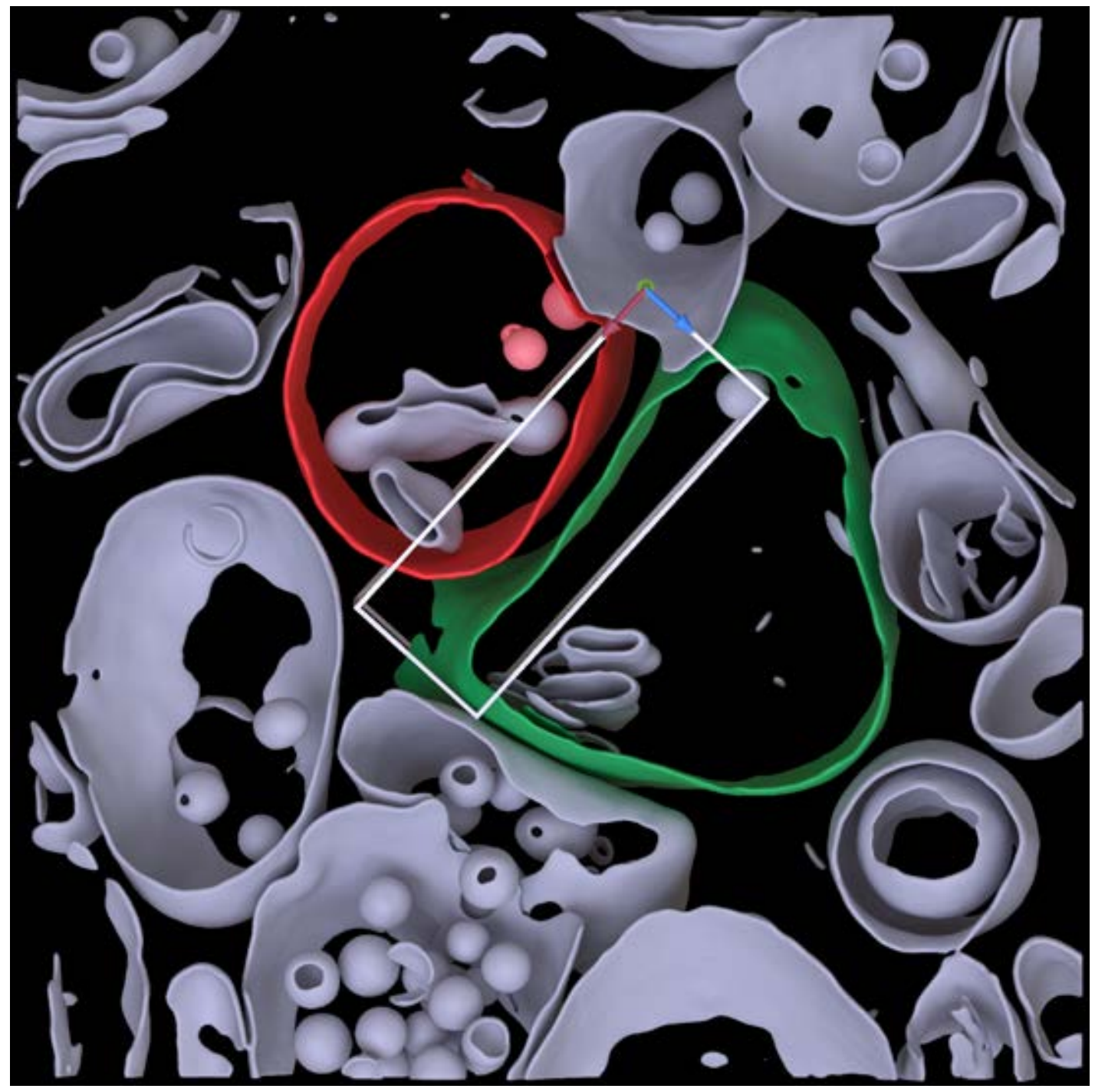

Cleft MIP

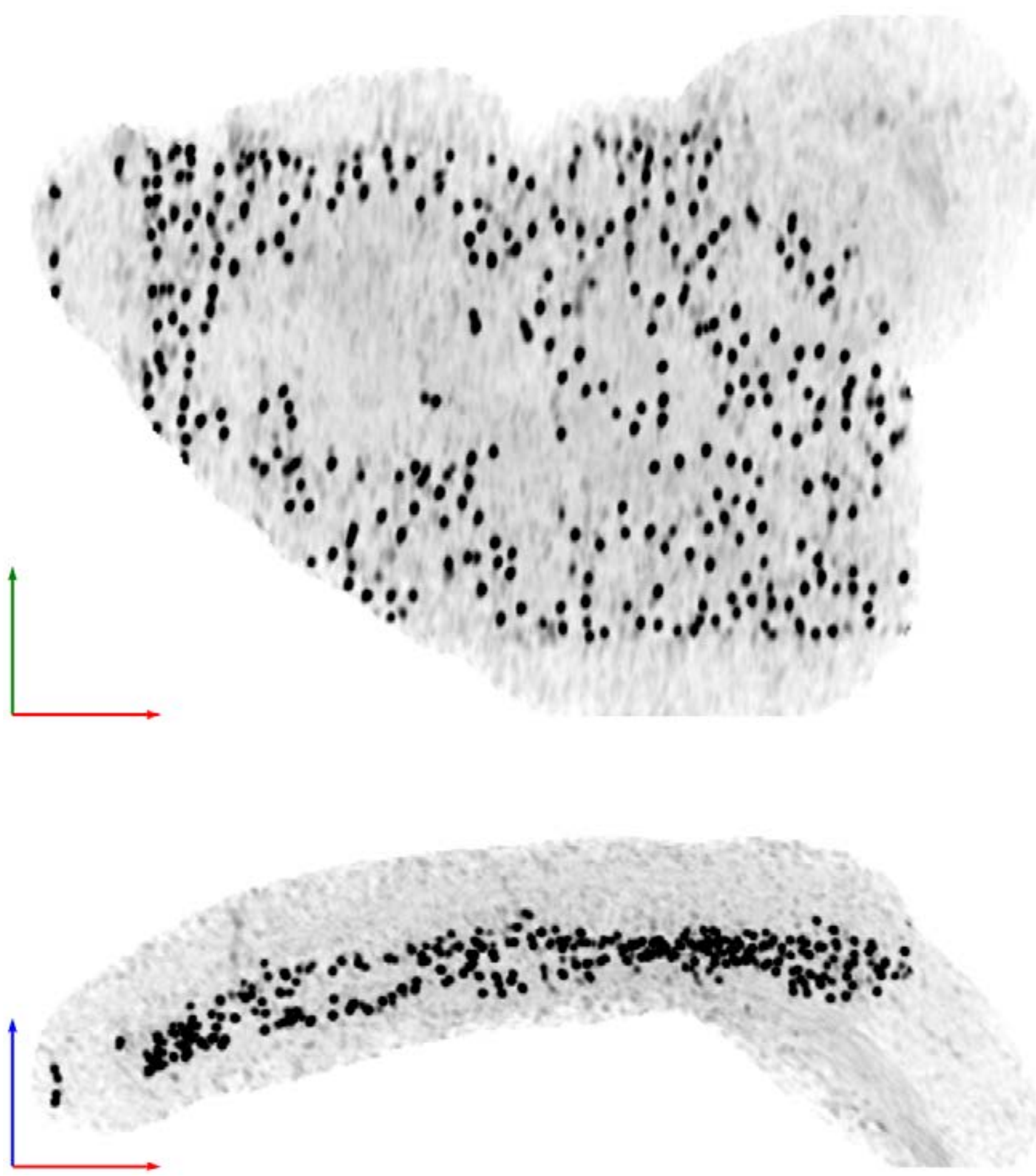

Tomogram ID: 20241211_HippWaffle_49	SET: Dual 15F1 monomer & 5F11 dimer
Cleft ID: 0	Type: tissue

Denoised Tomogram Slice

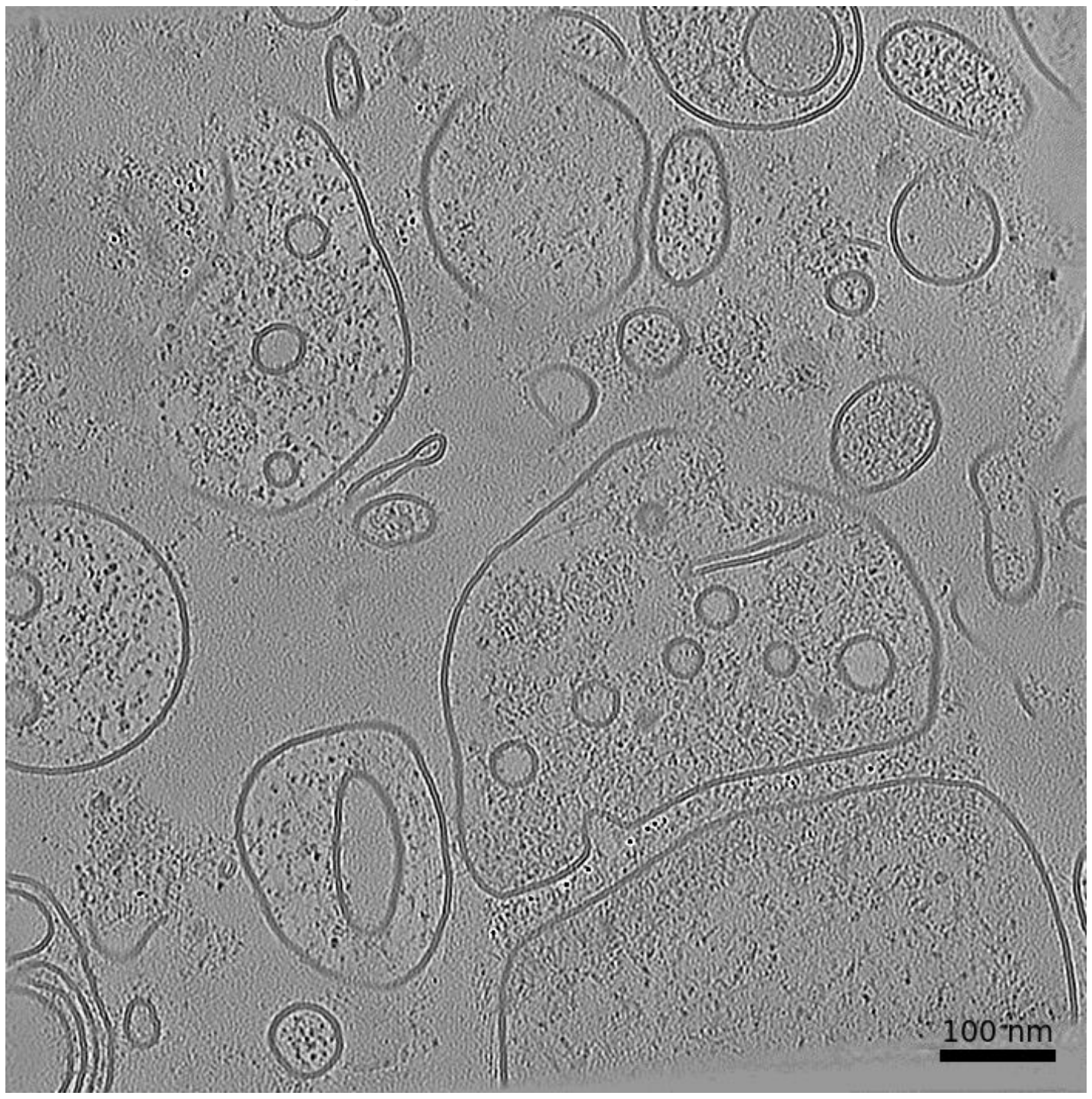

Membrane Segmentation & Cleft Position

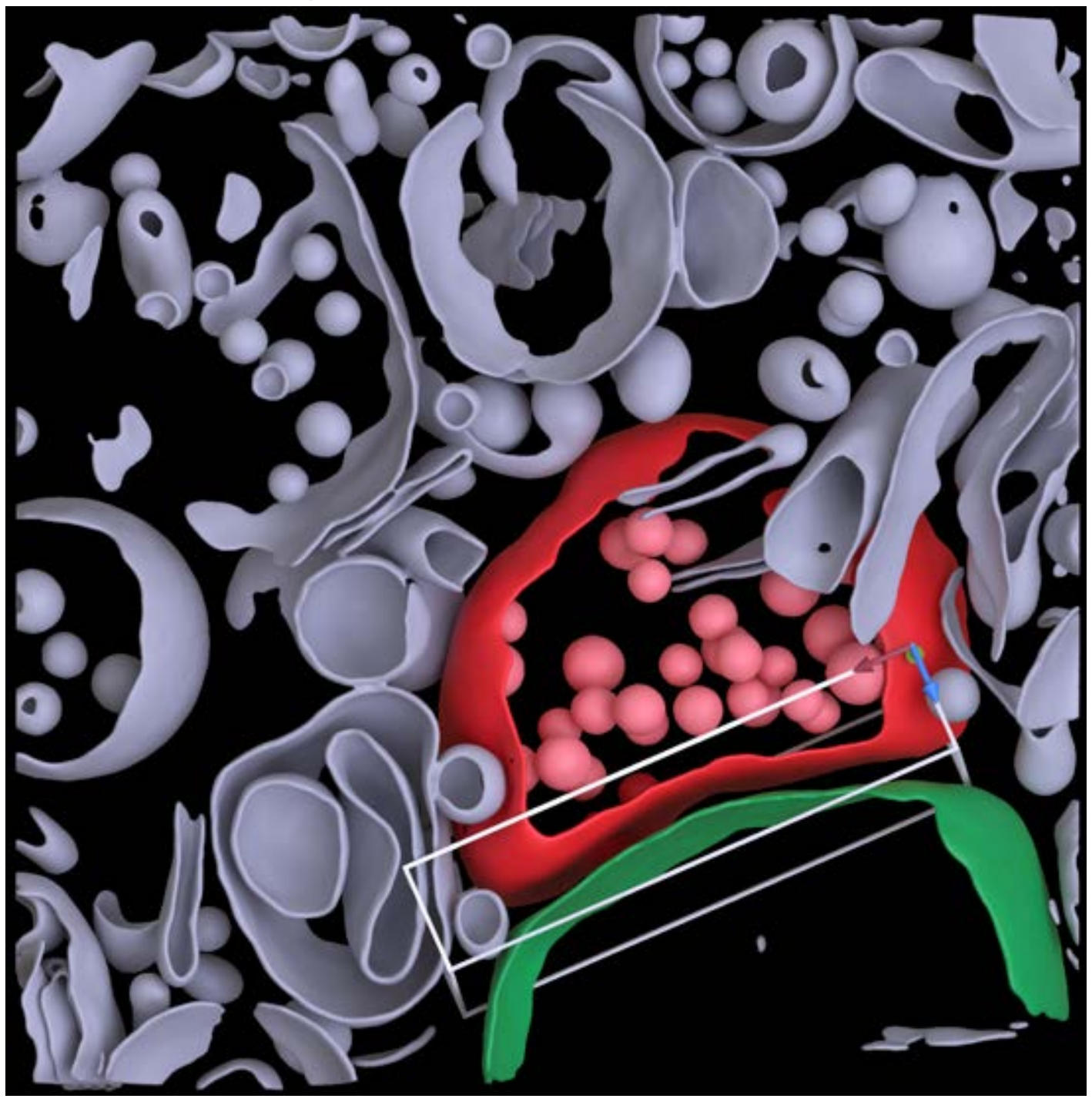

Cleft MIP

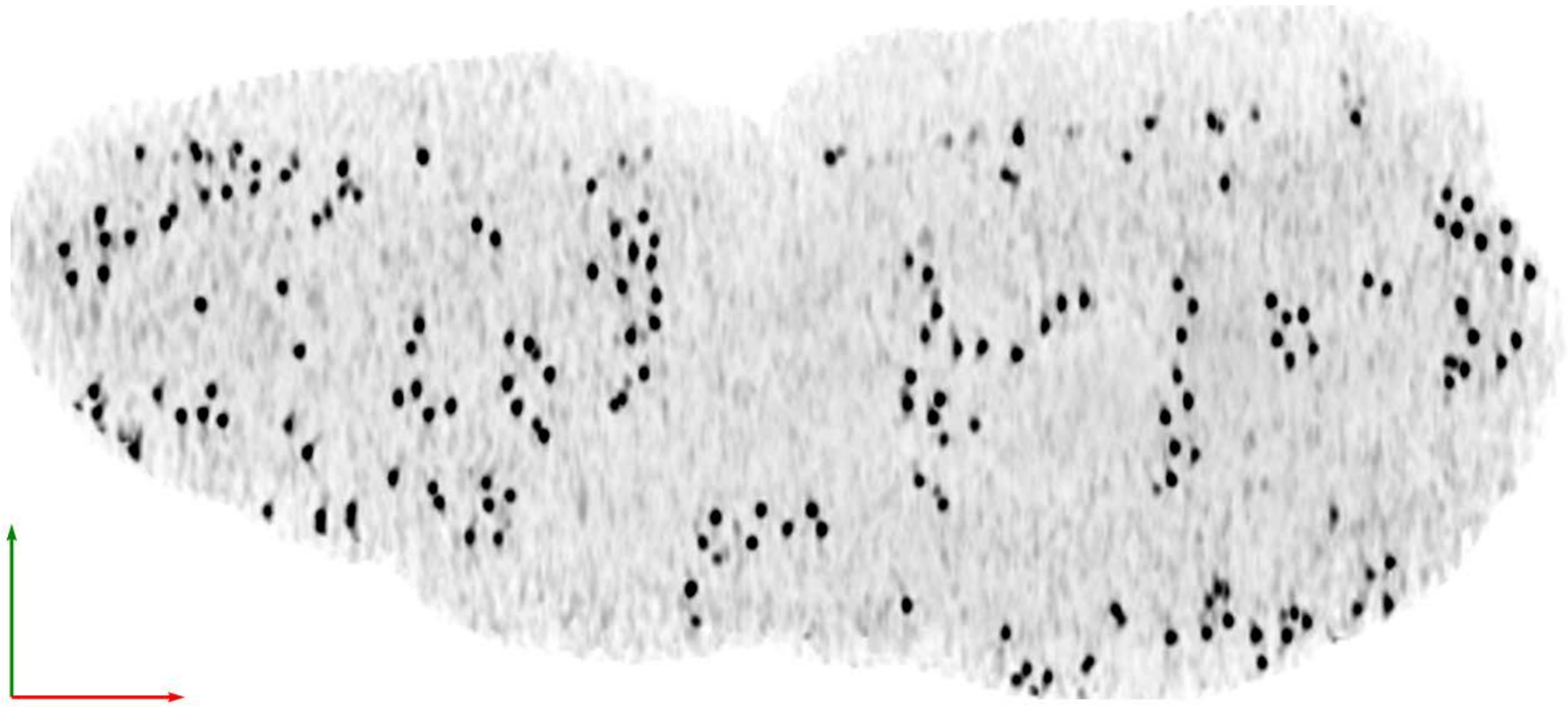

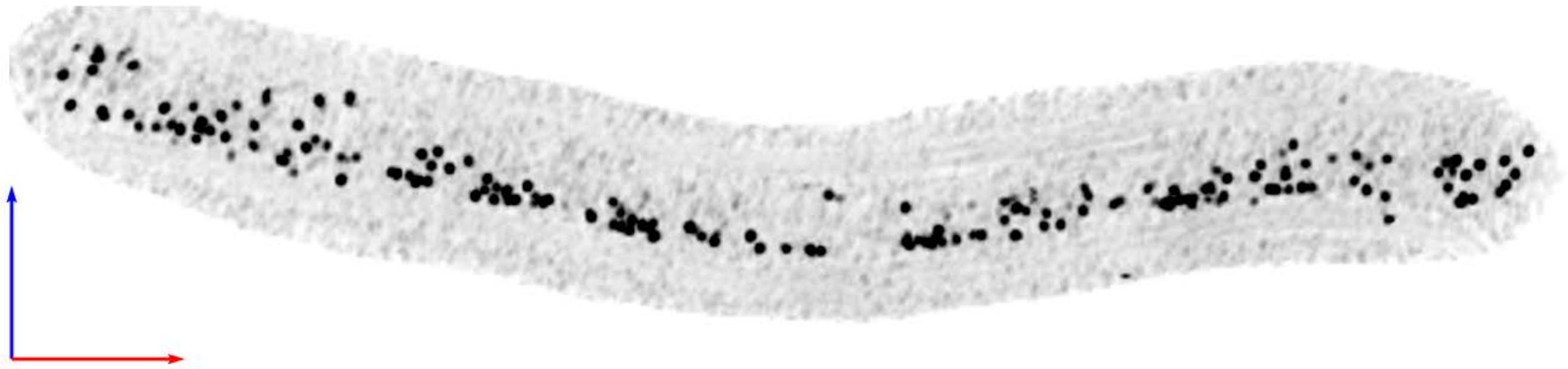

Tomogram ID: 20241211_HippWaffle_50 **SET: Dual 15F1 monomer & 5F11 dimer**

Cleft ID: 0 Type: semi-tissue

Denoised Tomogram Slice

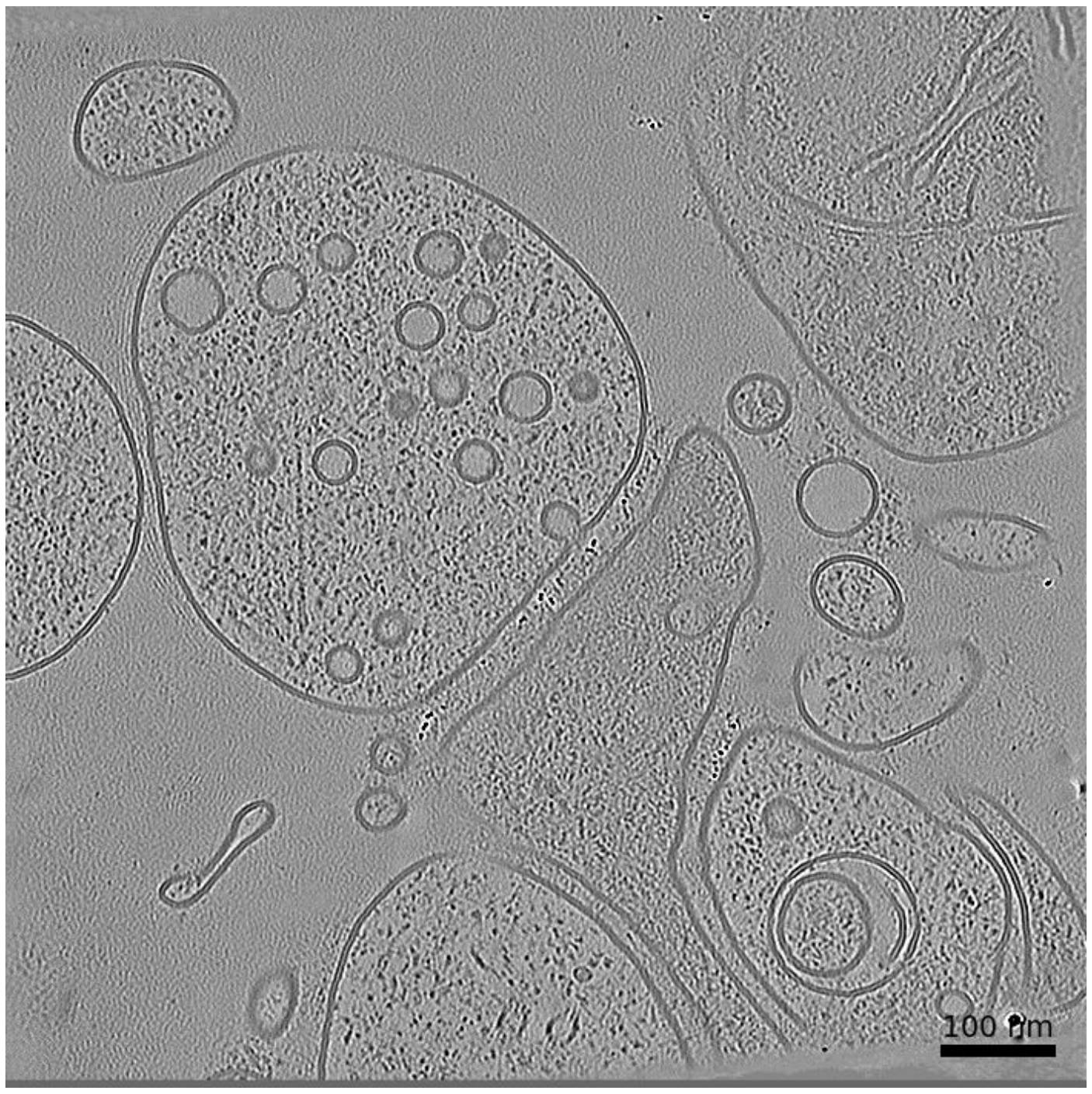

Membrane Segmentation & Cleft Position

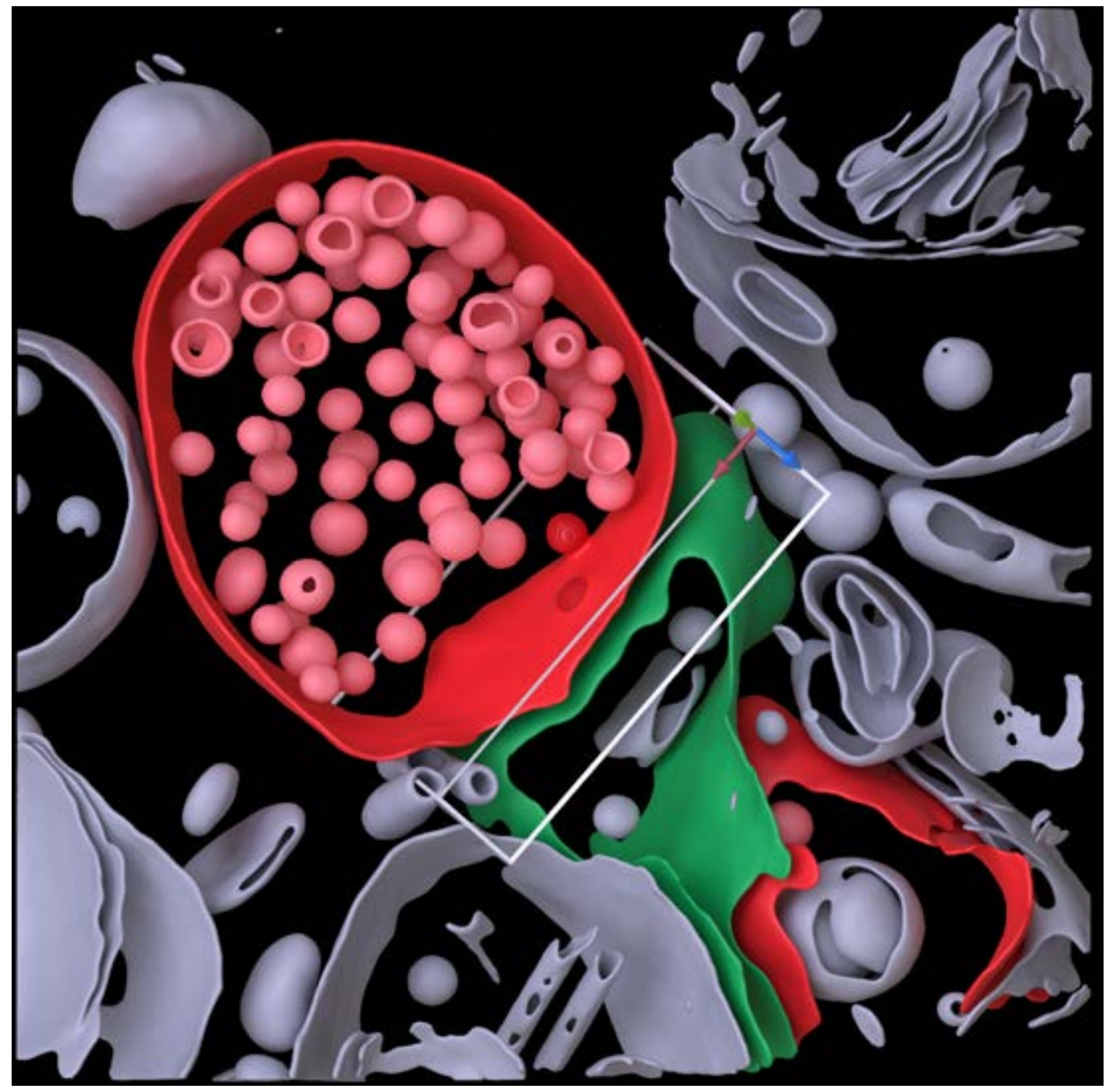

Cleft MIP

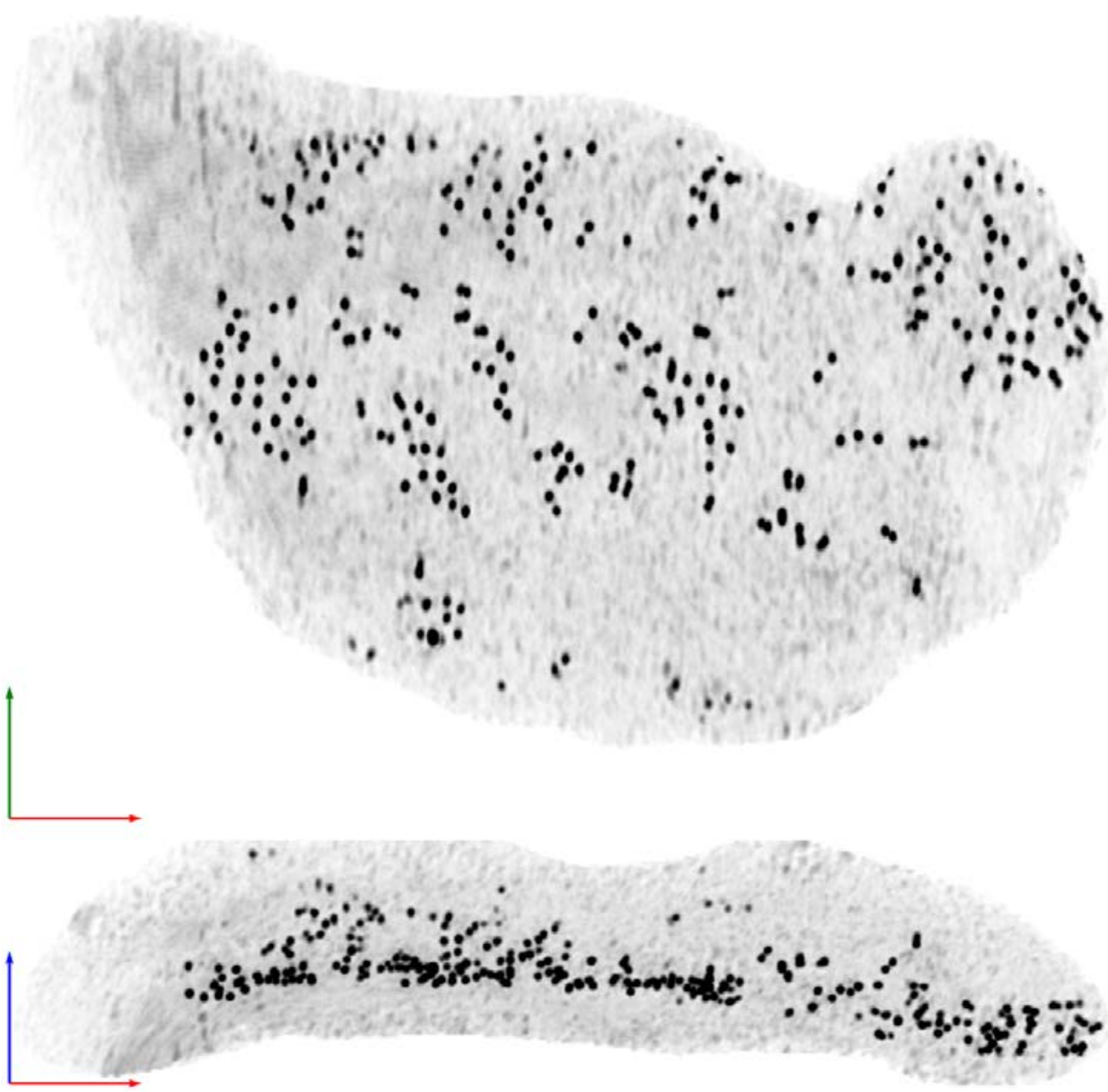

Tomogram ID: 20241211_HippWaffle_50 **SET: Dual 15F1 monomer & 5F11 dimer**

Cleft ID: 1 Type: semi-tissue

Denoised Tomogram Slice

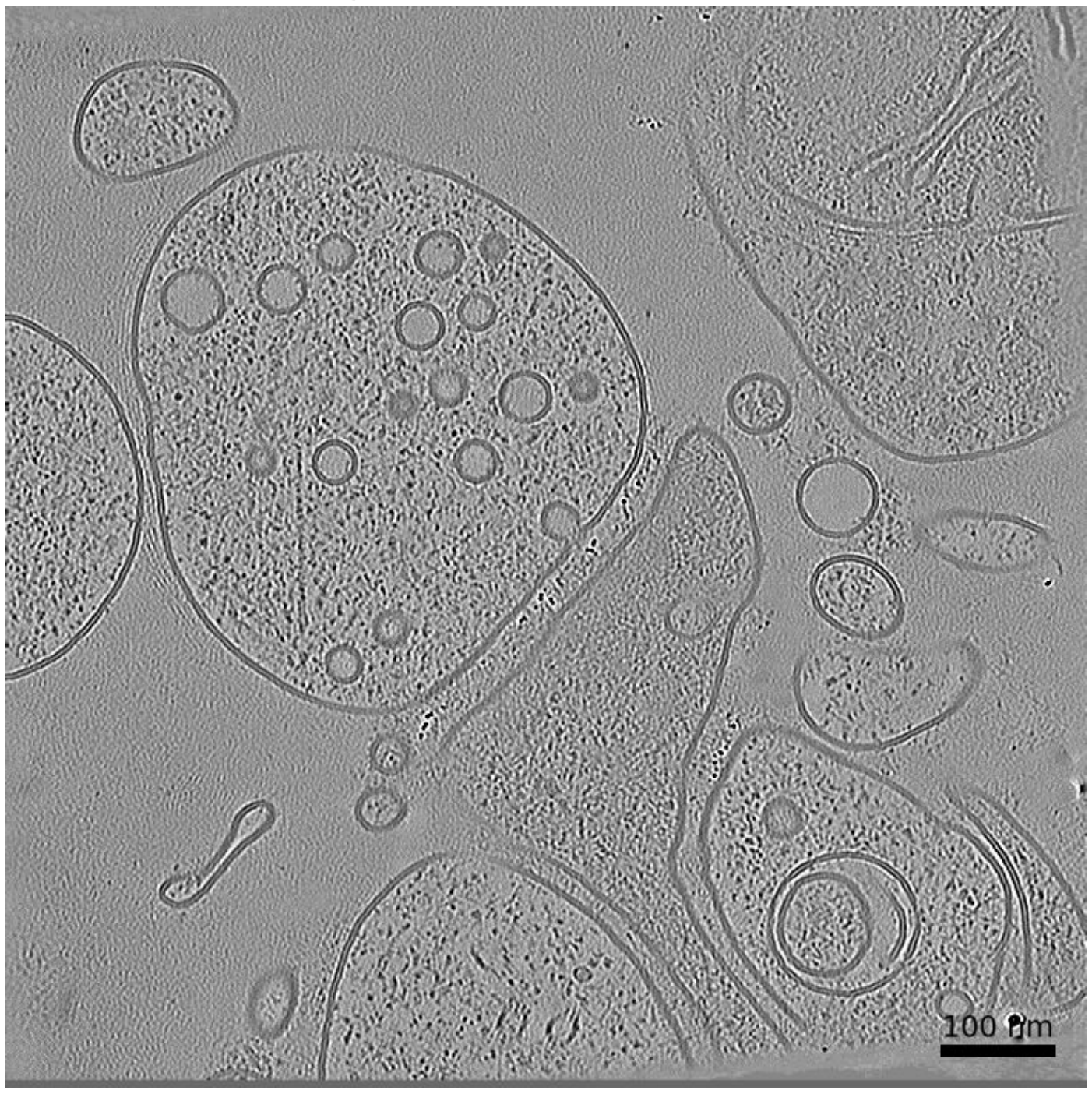

Membrane Segmentation & Cleft Position

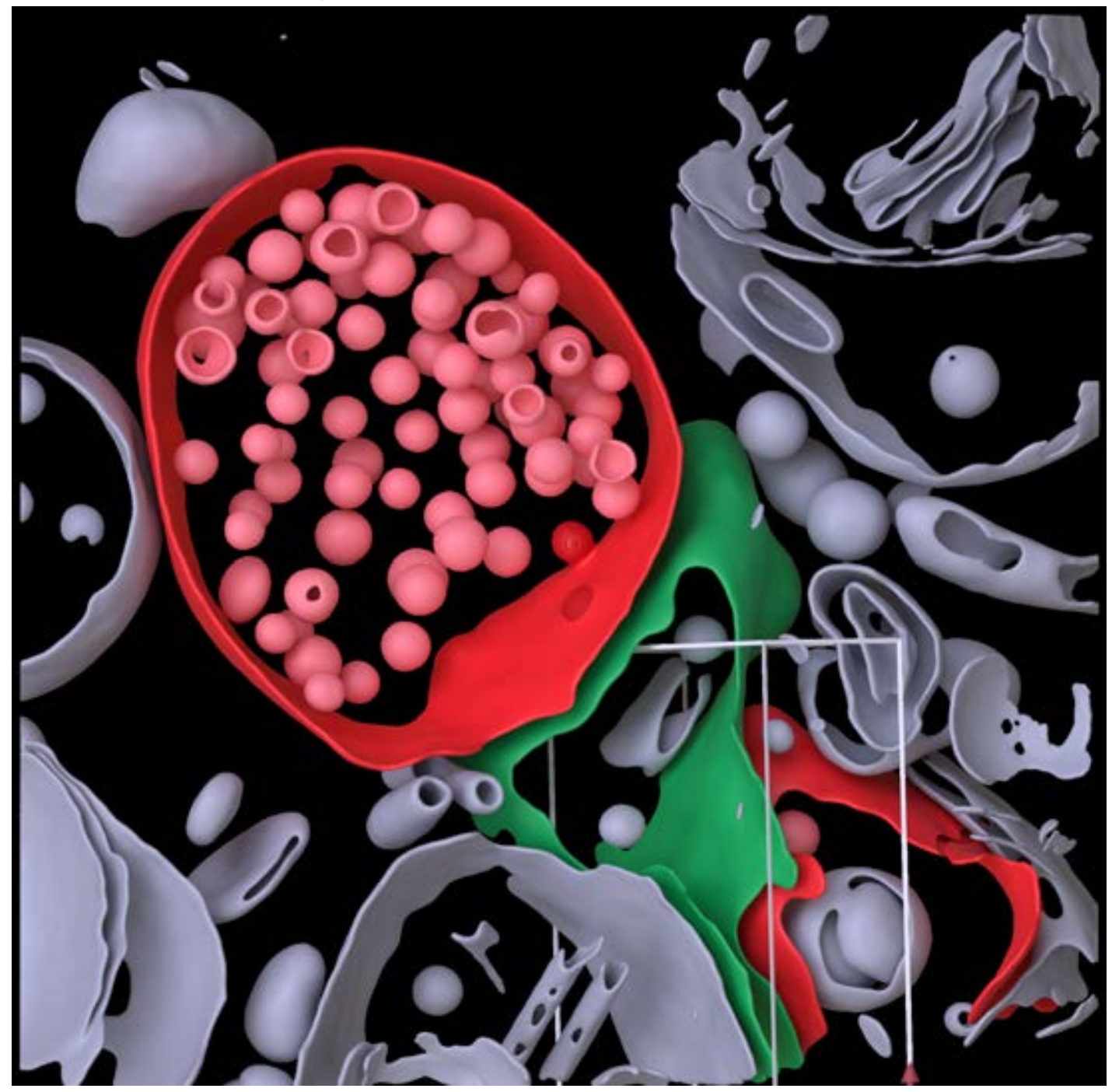

Cleft MIP

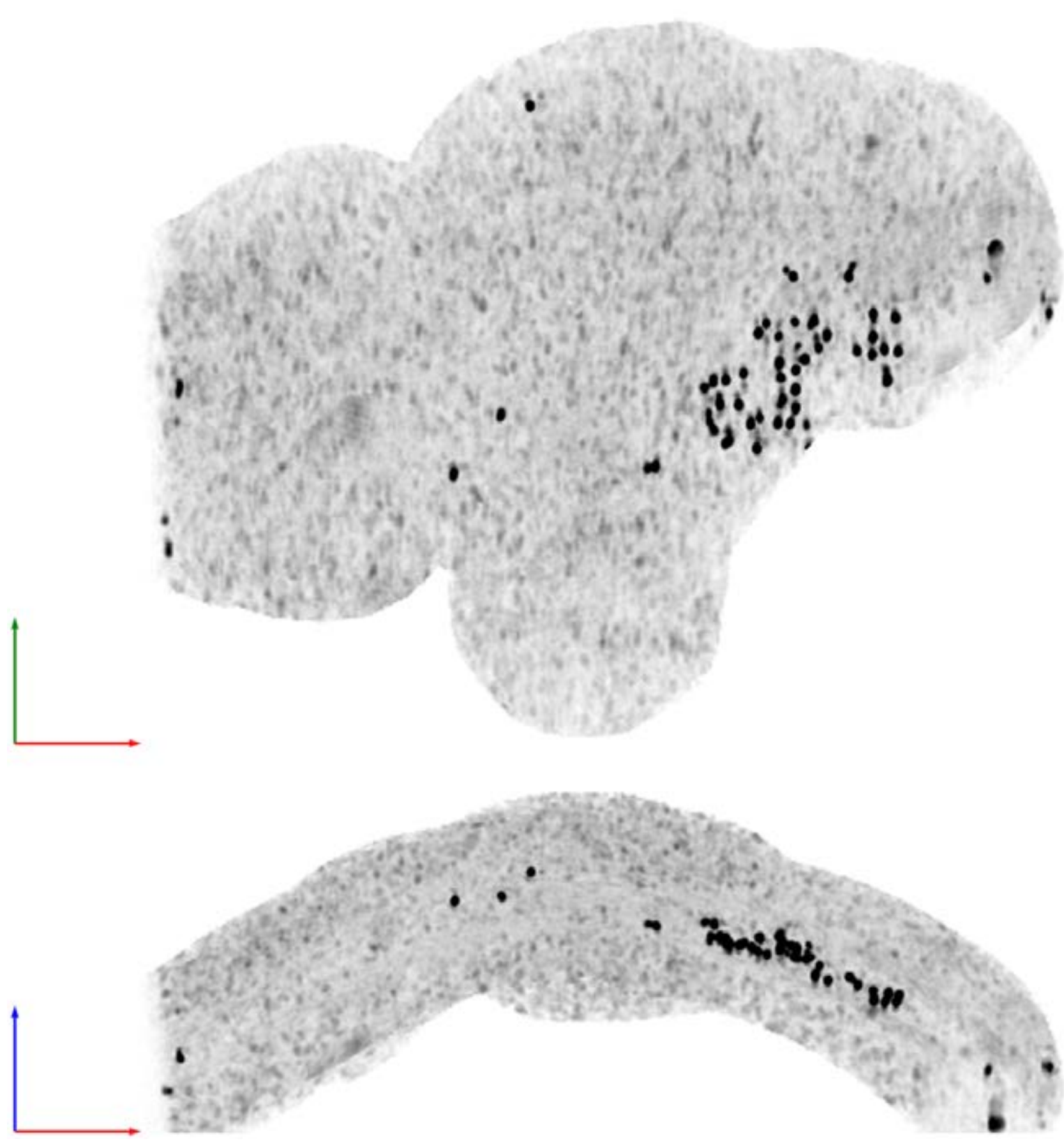

Tomogram ID: 20250418_AMmilled29-2_Position_87 **SET: Dual 15F1 monomer & 5F11 dimer**

Cleft ID: 0 Type: semi-tissue

Denoised Tomogram Slice

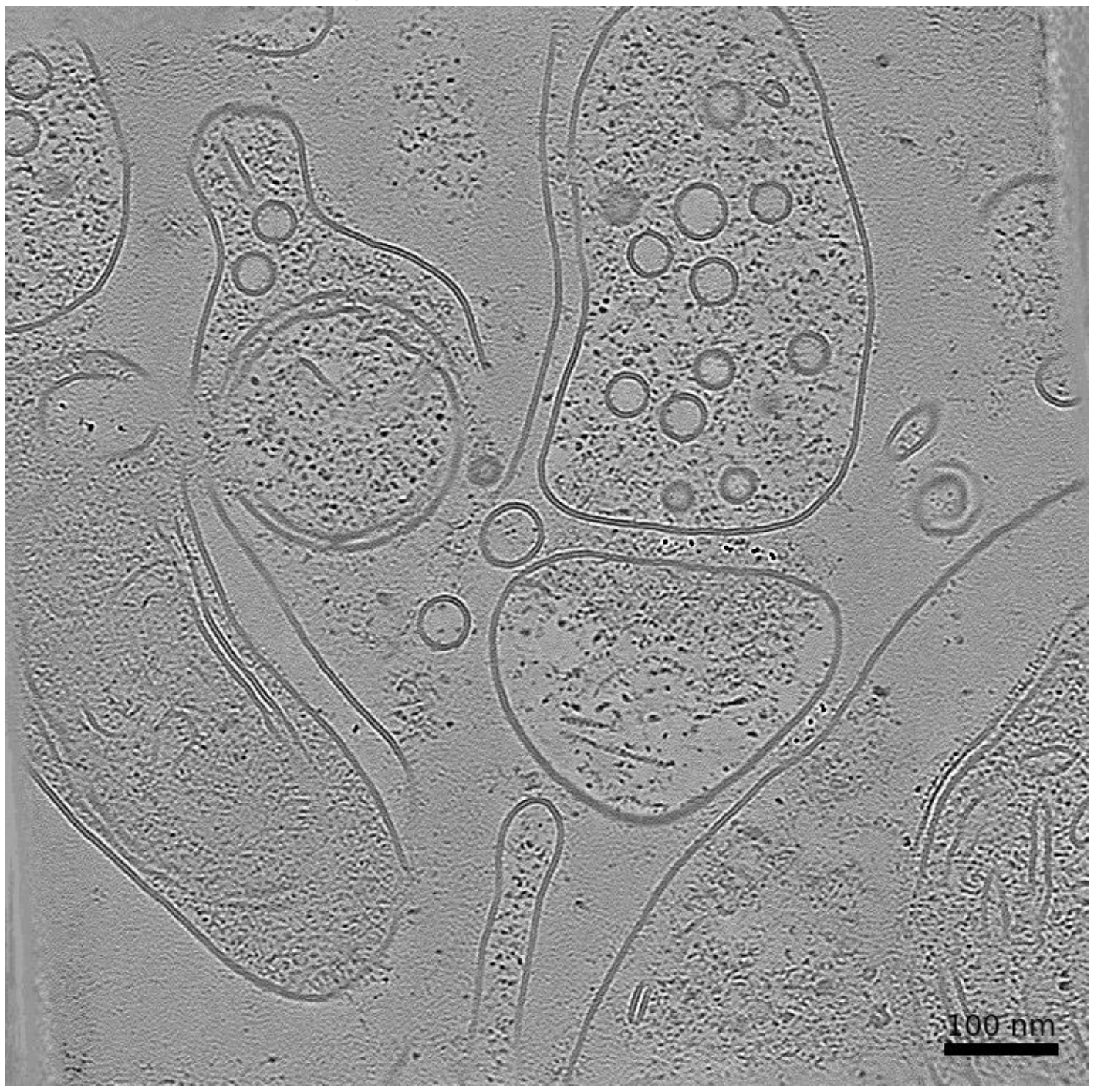

Membrane Segmentation & Cleft Position

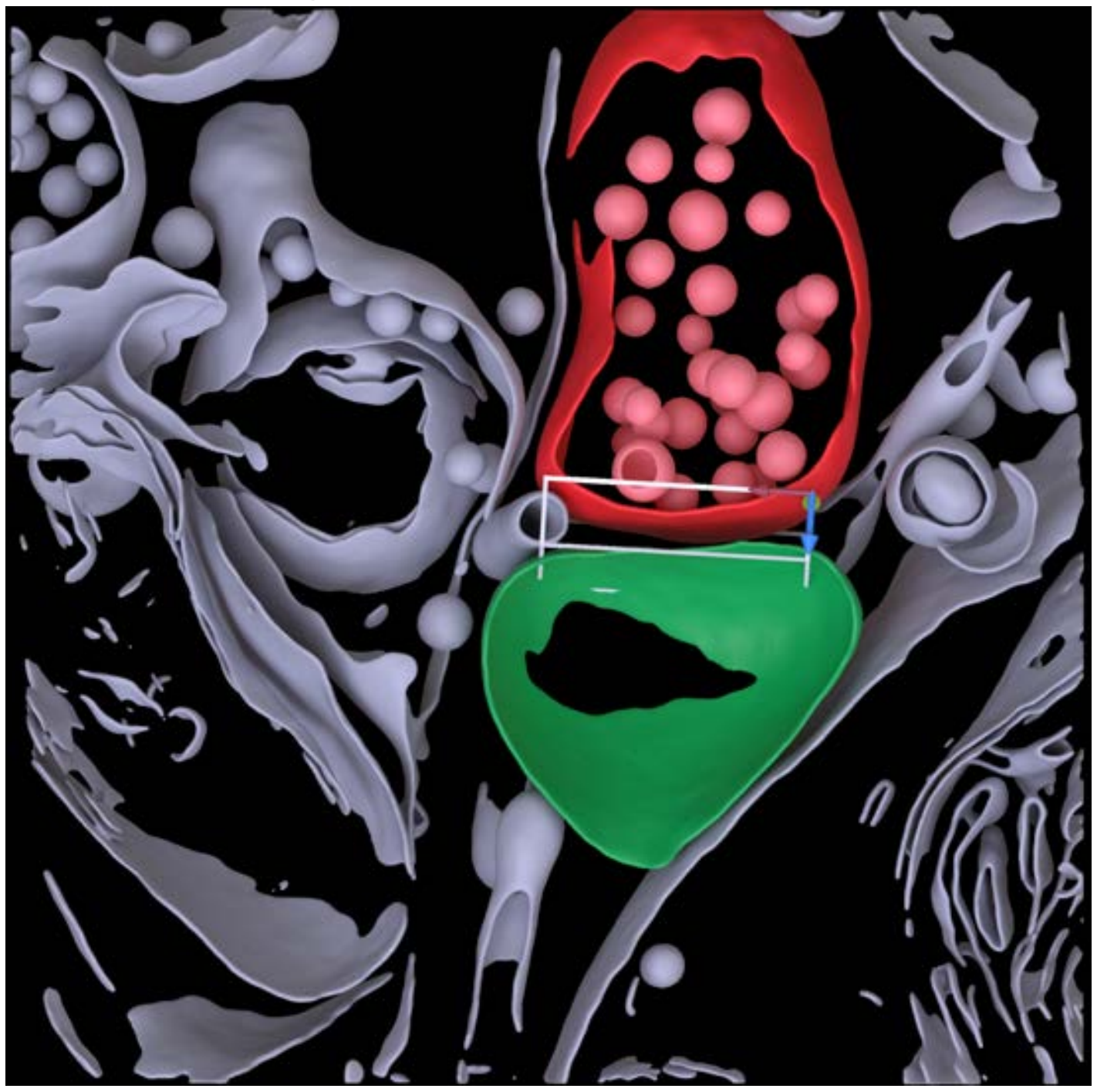

Cleft MIP

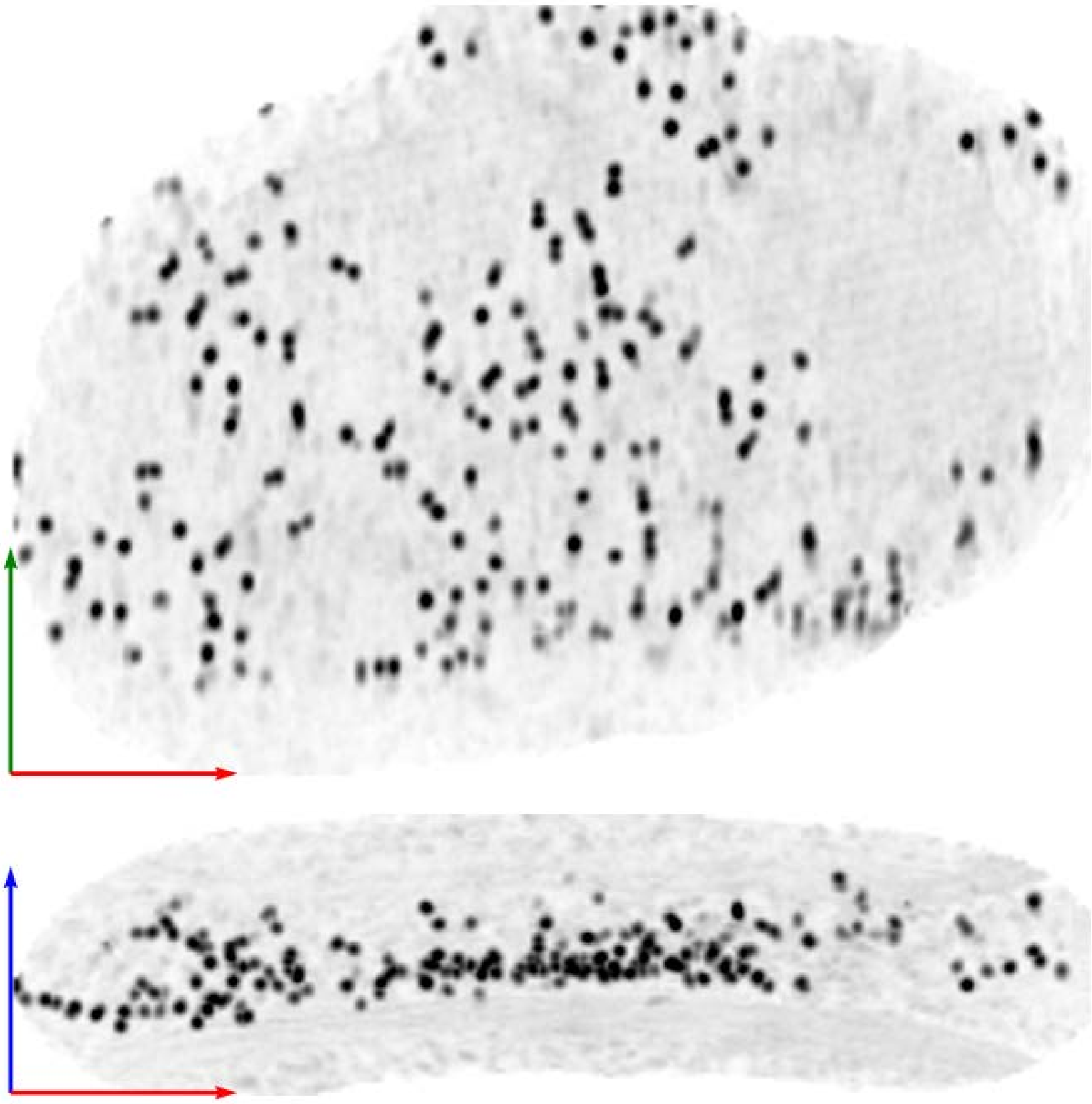

Tomogram ID: 20251117_AMmilled40-2_Position_148 | **SET: Dual 15F1 monomer & 5F11 dimer**

Cleft ID: 0 | Type: tissue

Denoised Tomogram Slice

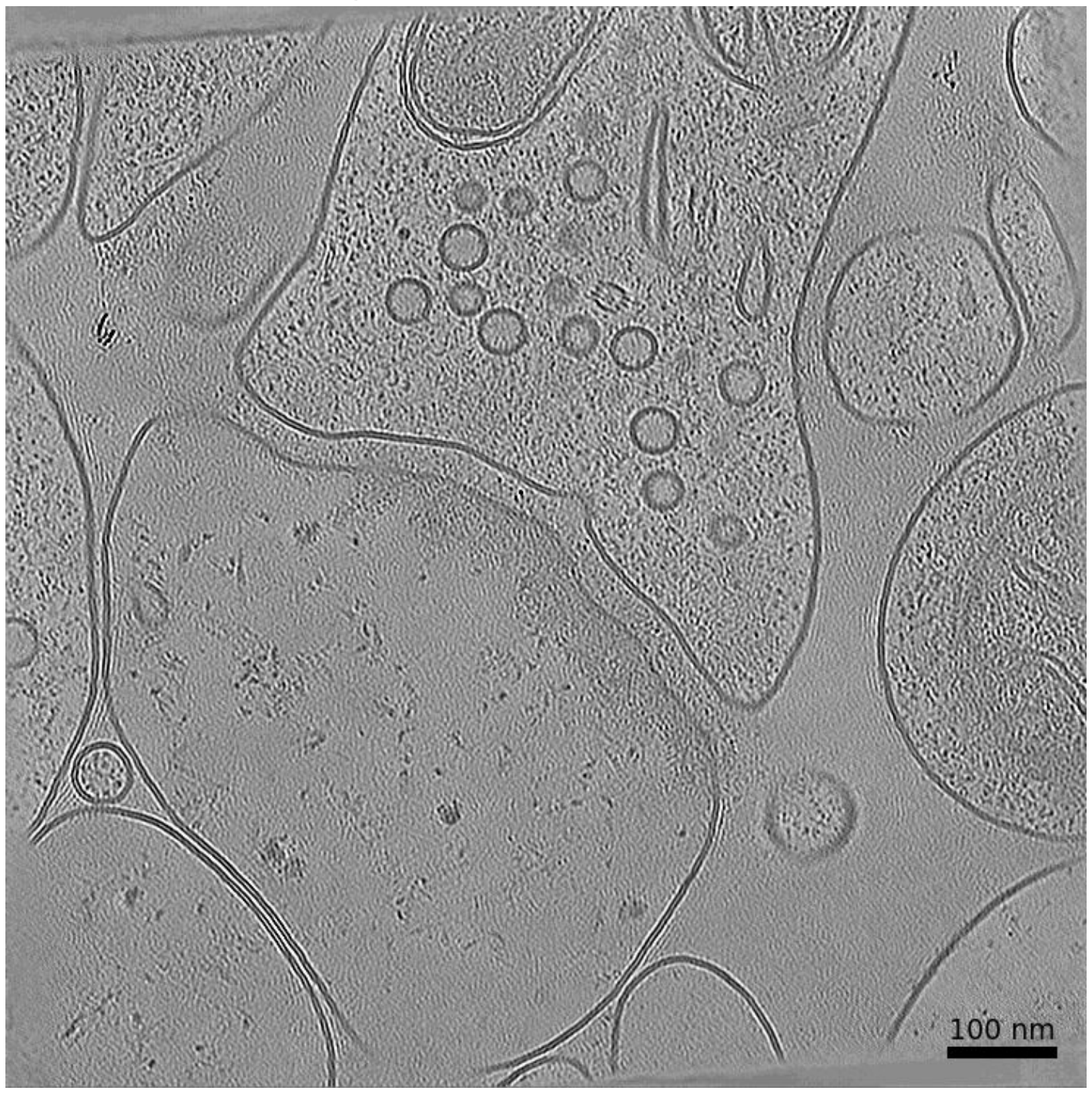

Membrane Segmentation & Cleft Position

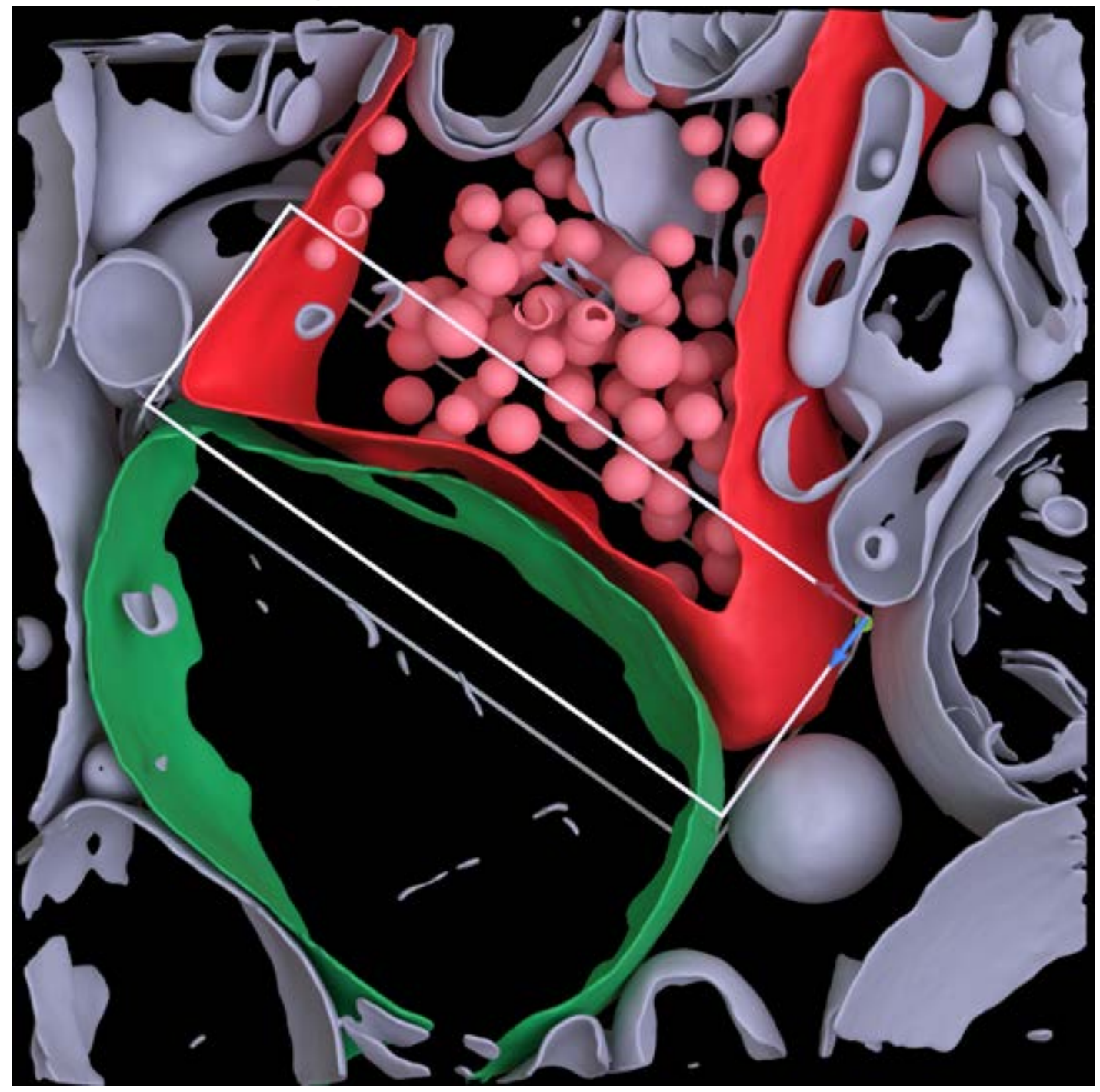

Cleft MIP

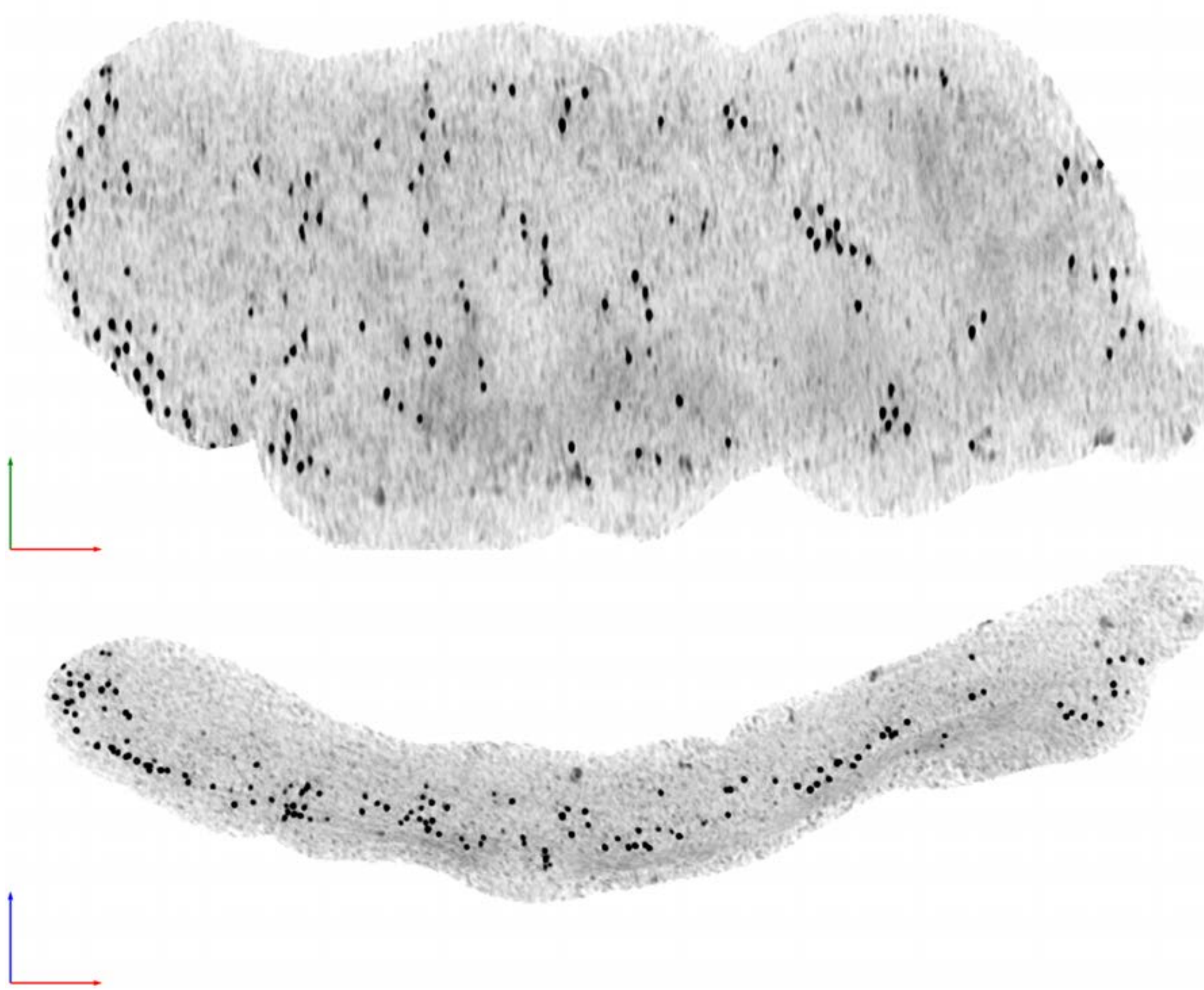

**Tomogram ID: 20251117_AMmilled40-2_Position_148**	**SET: Dual 15F1 monomer & 5F11 dimer**
Cleft ID: 0	Type: tissue

Denoised Tomogram Slice

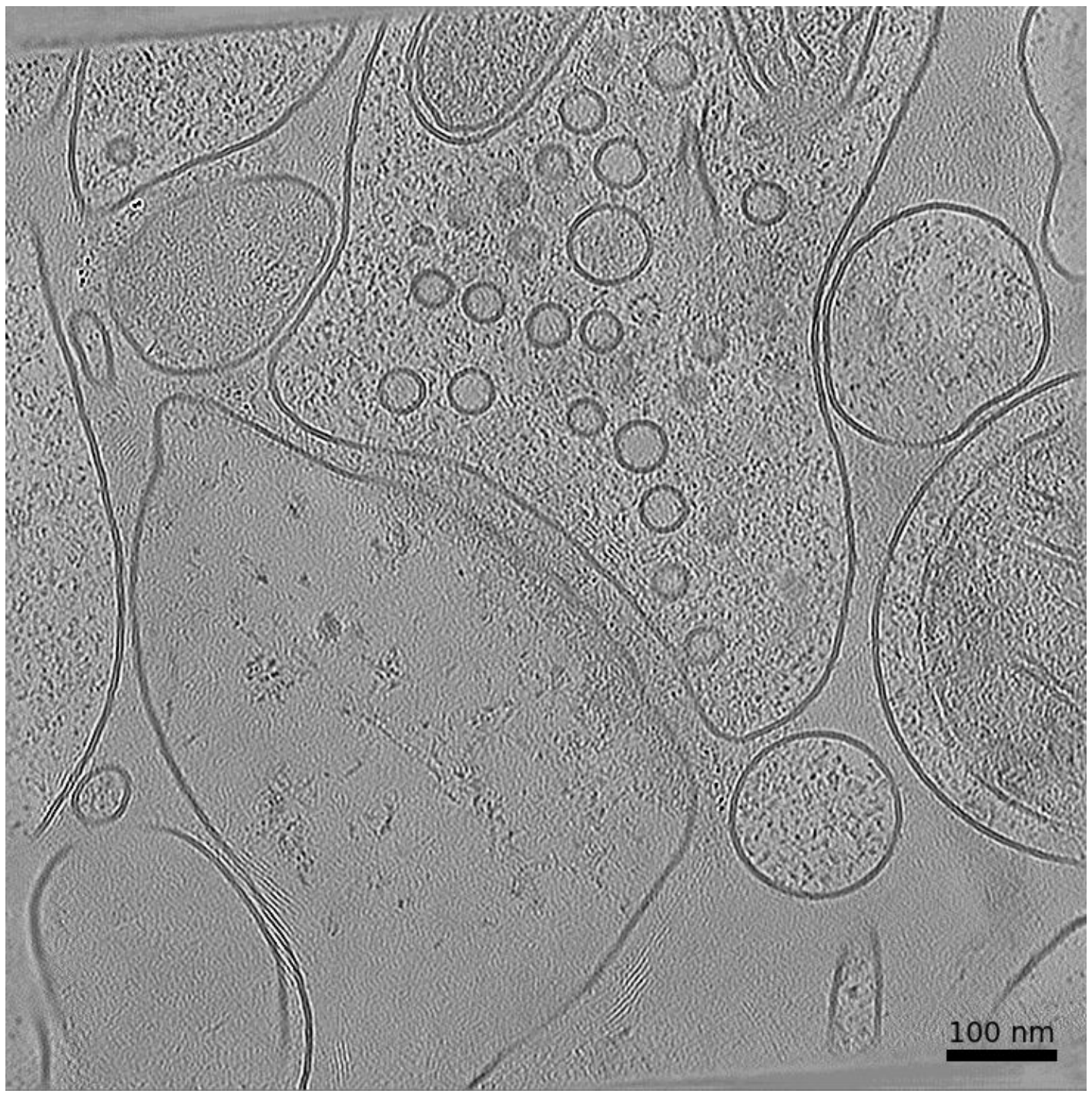

Membrane Segmentation & Cleft Position

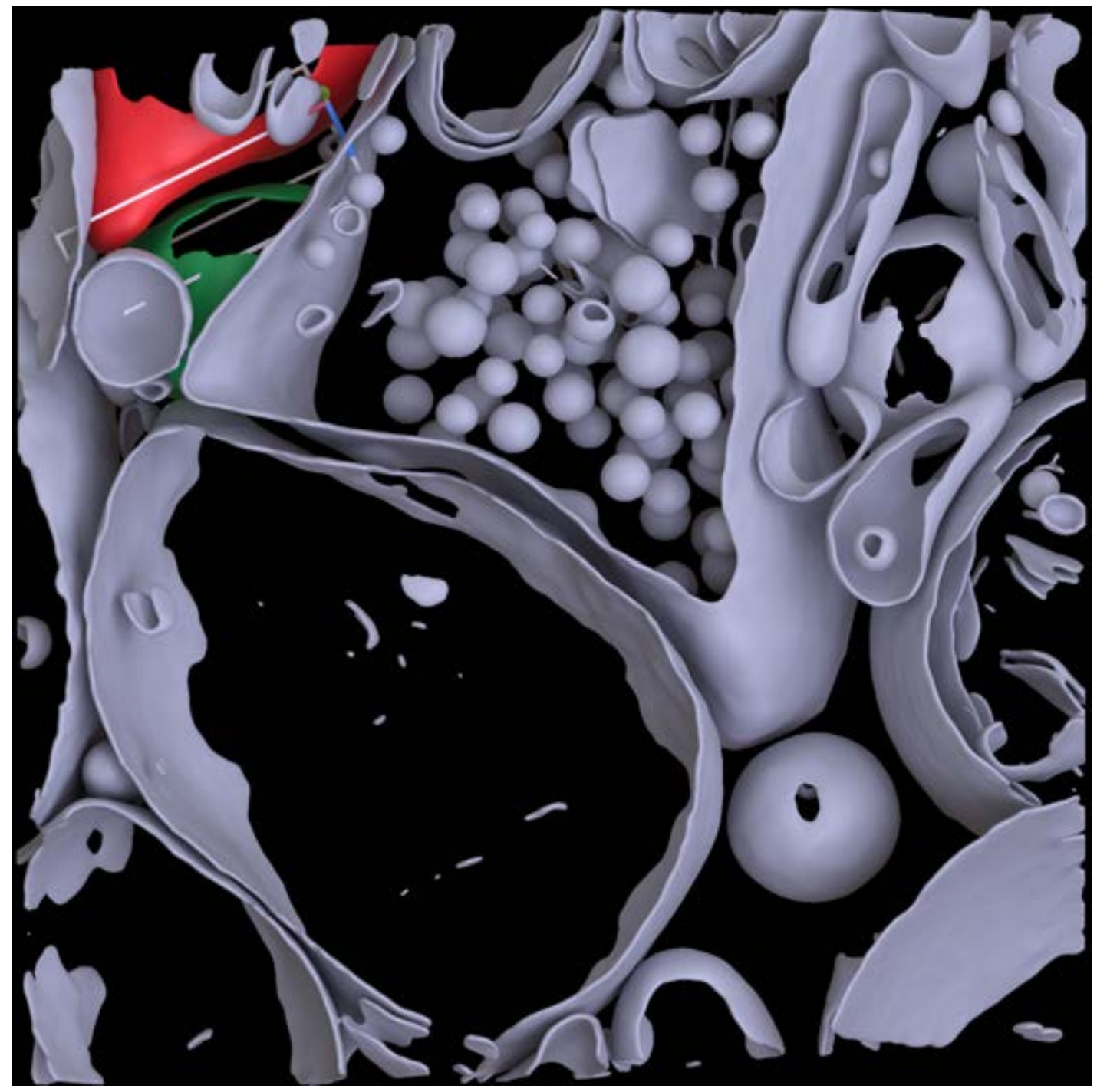

Cleft MIP

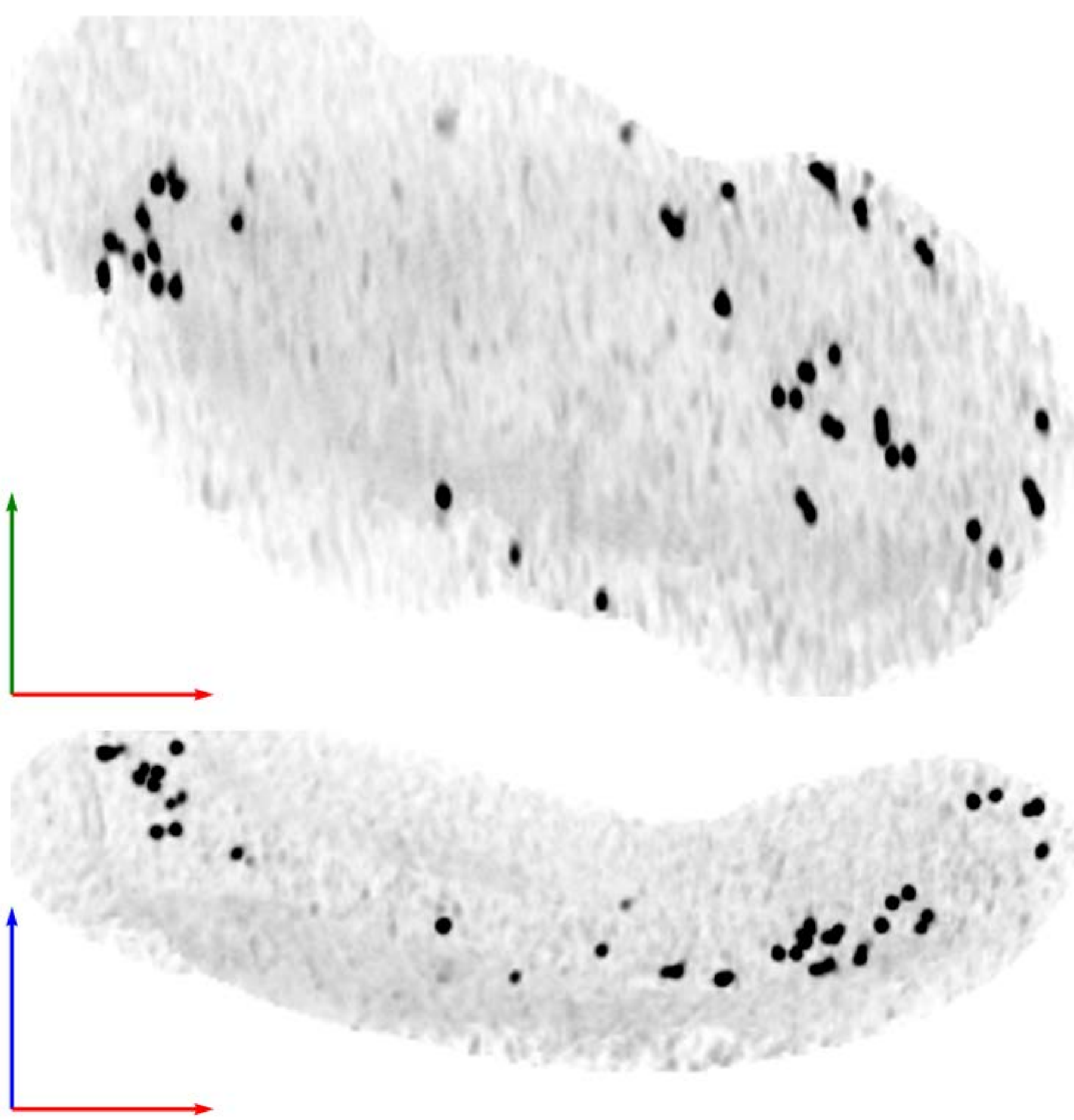

Tomogram ID: 20260127_AMmilled50-3_Position_13_3	SET: Dual 15F1 monomer & 5F11 dimer
Cleft ID: 1	Type: tissue

Denoised Tomogram Slice

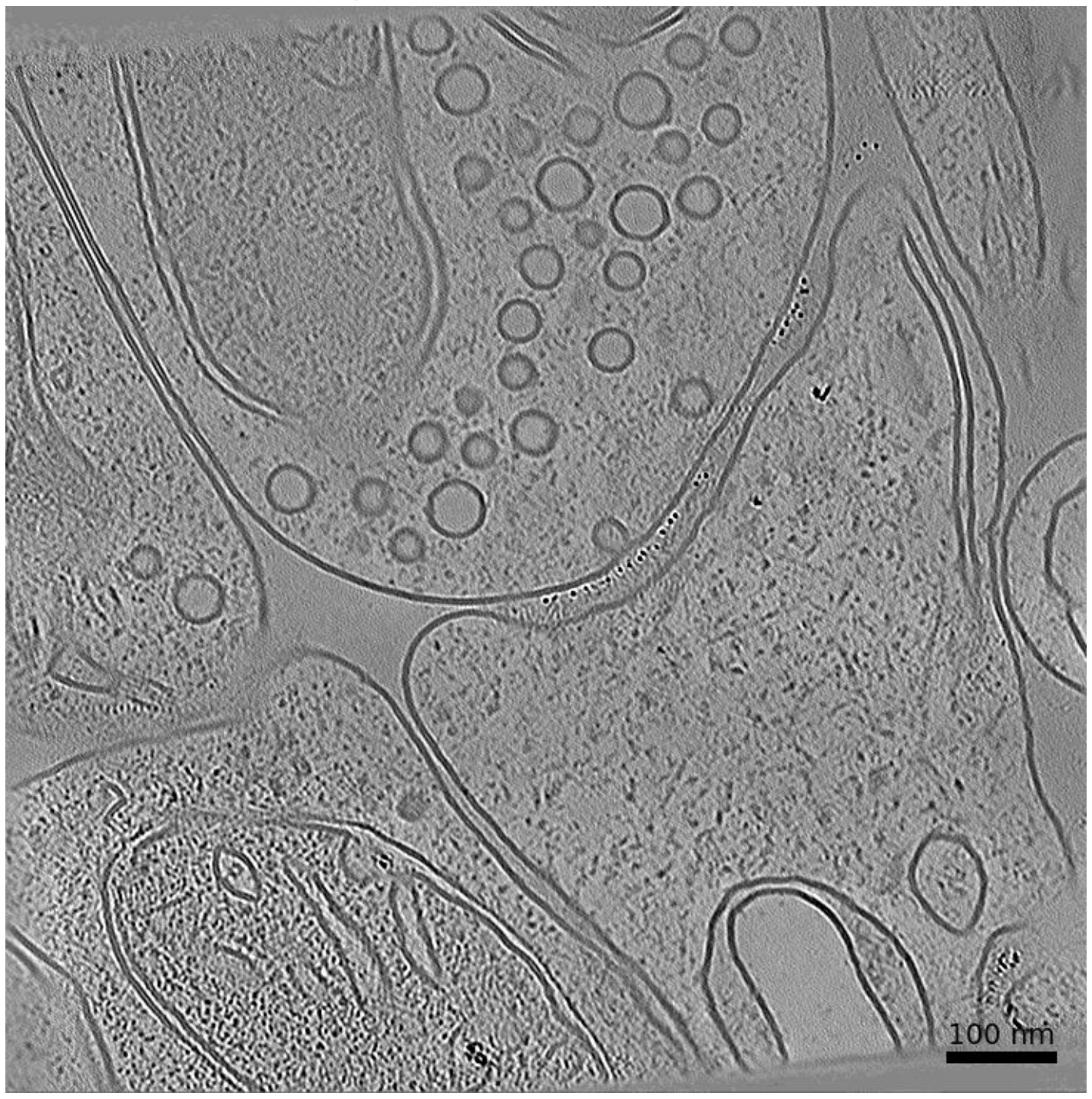

Membrane Segmentation & Cleft Position

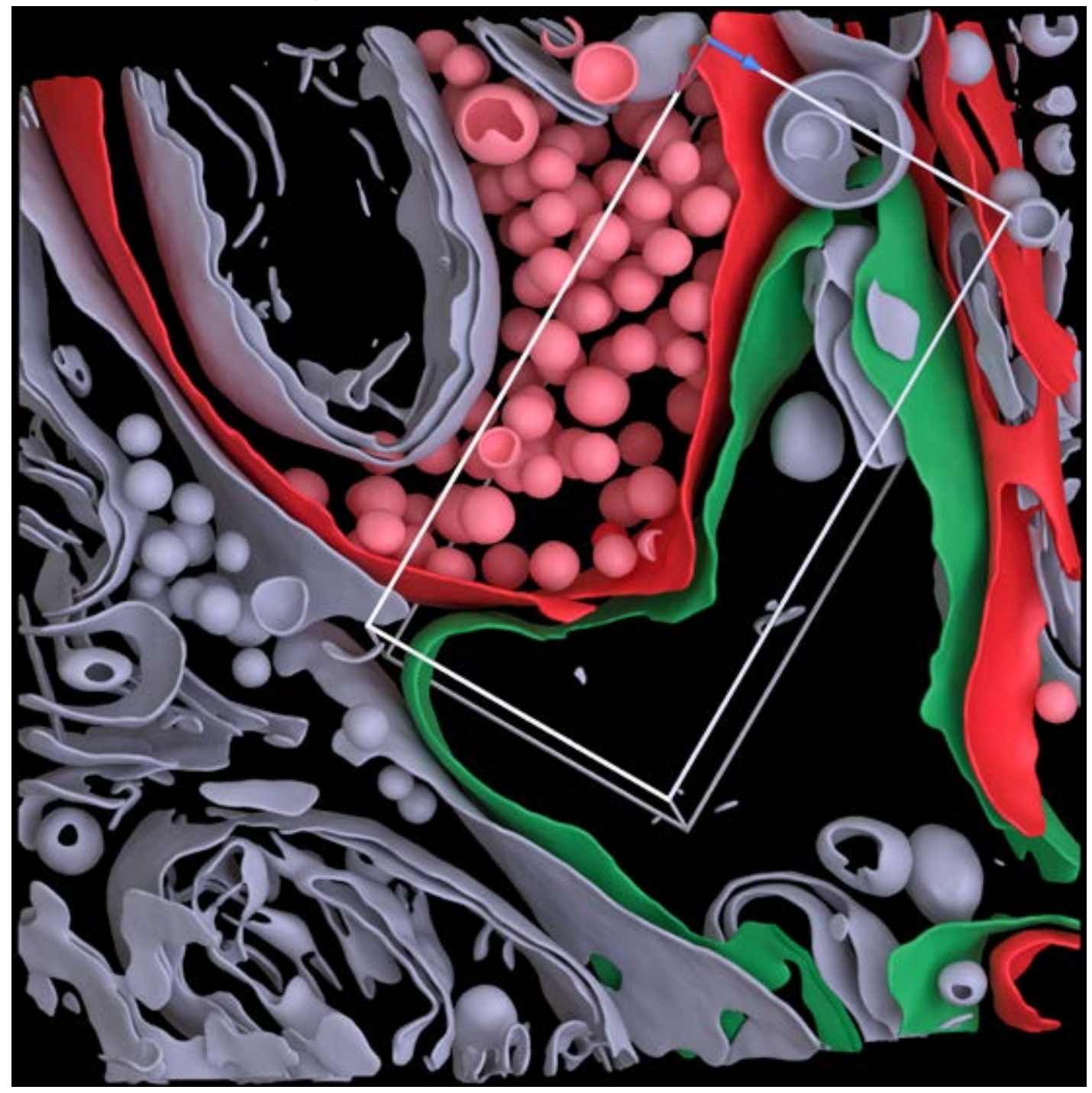

Cleft MIP

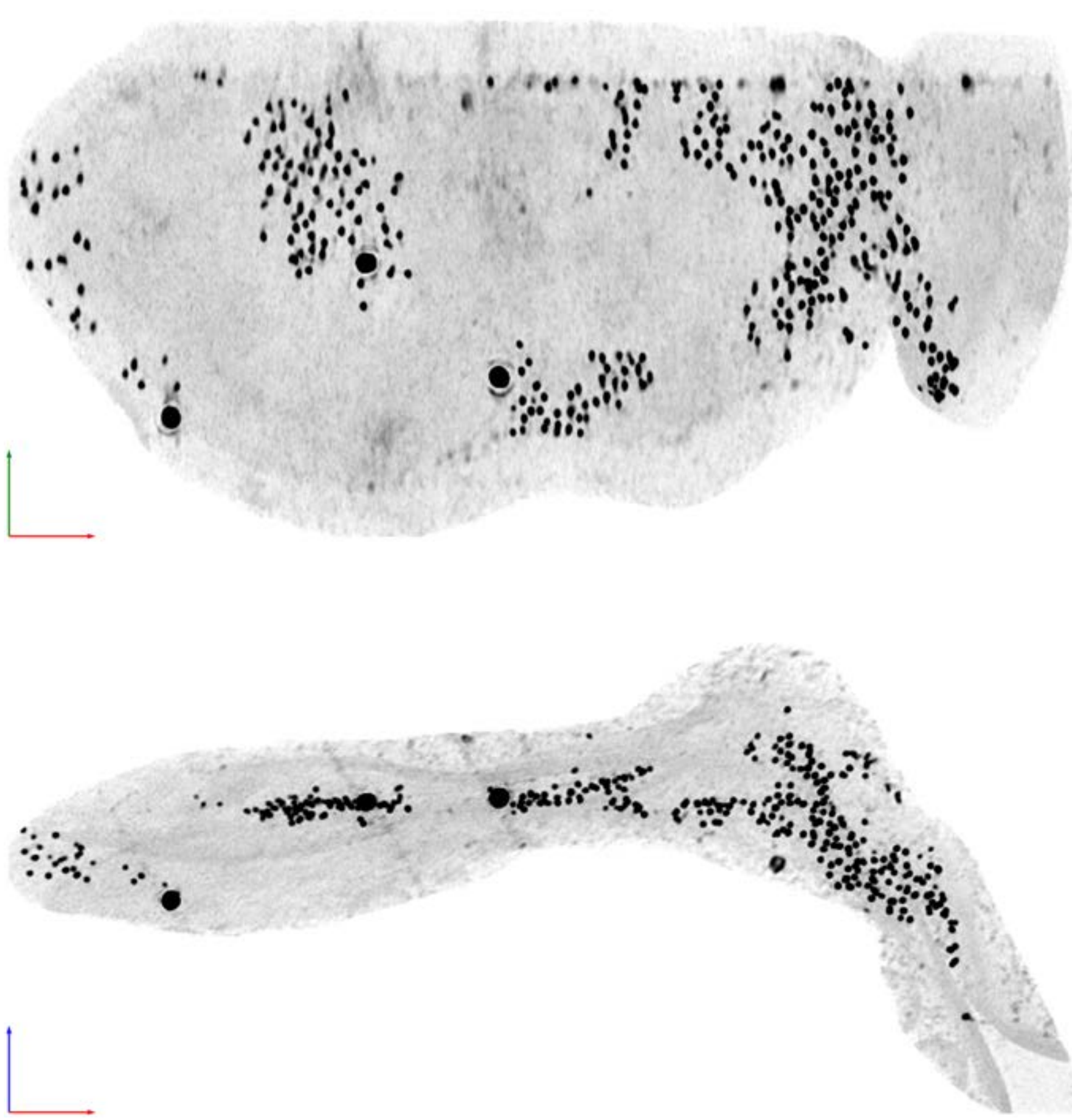

Tomogram ID: 20260127_AMmilled50-3_Position_13_3	SET: Dual 15F1 monomer & 5F11 dimer
Cleft ID: 1	Type: tissue

Denoised Tomogram Slice

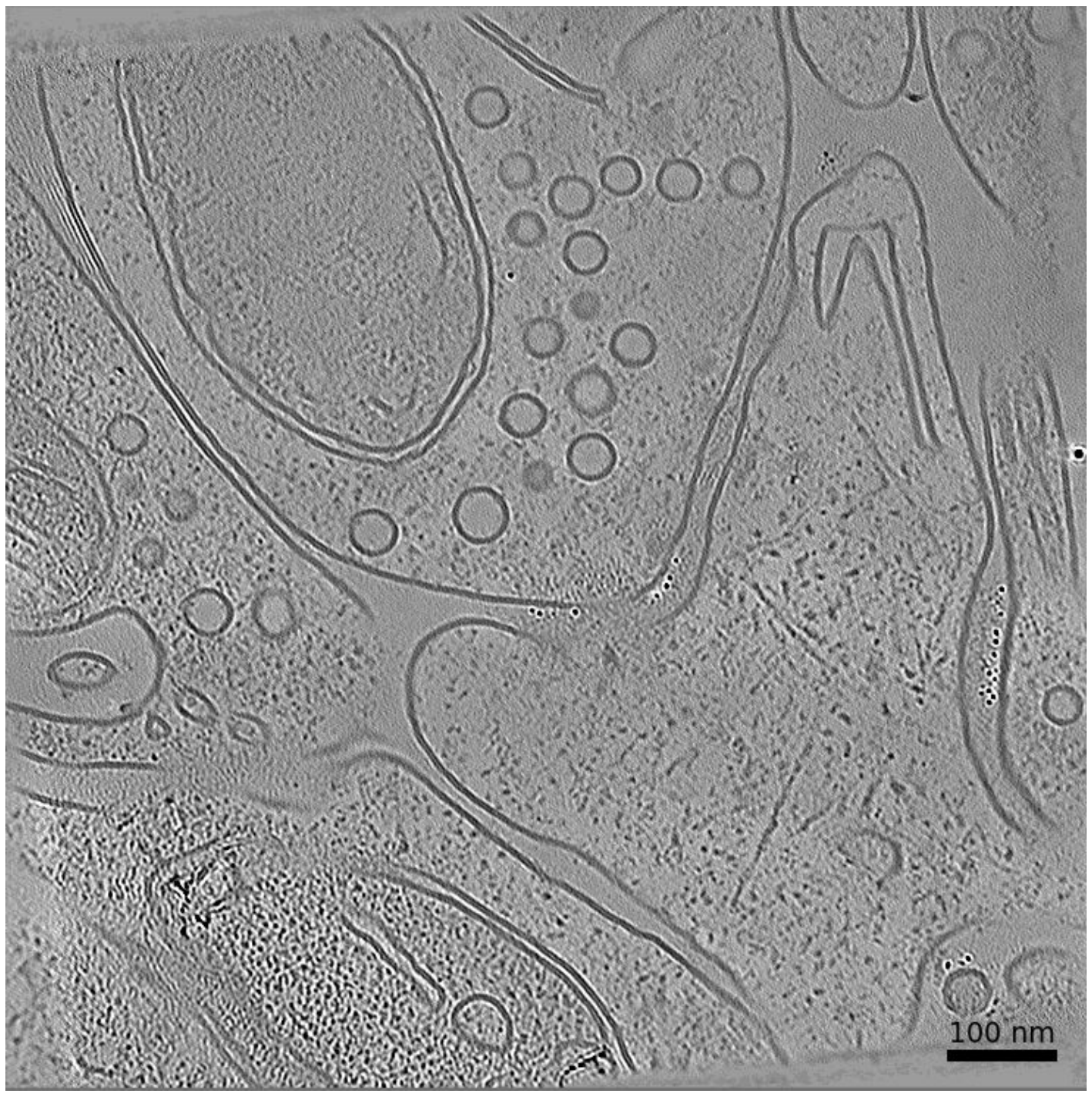

Membrane Segmentation & Cleft Position

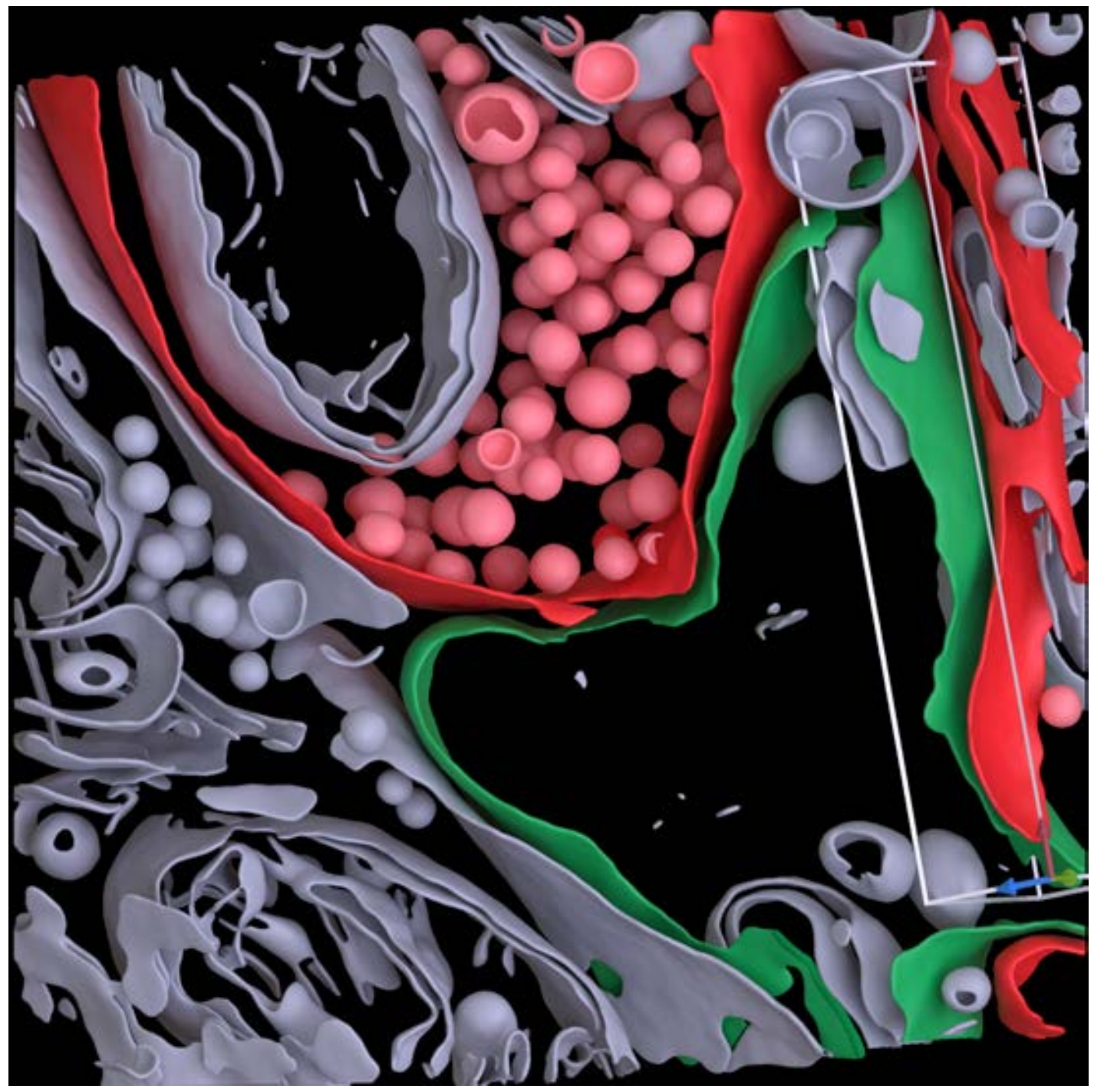

Cleft MIP

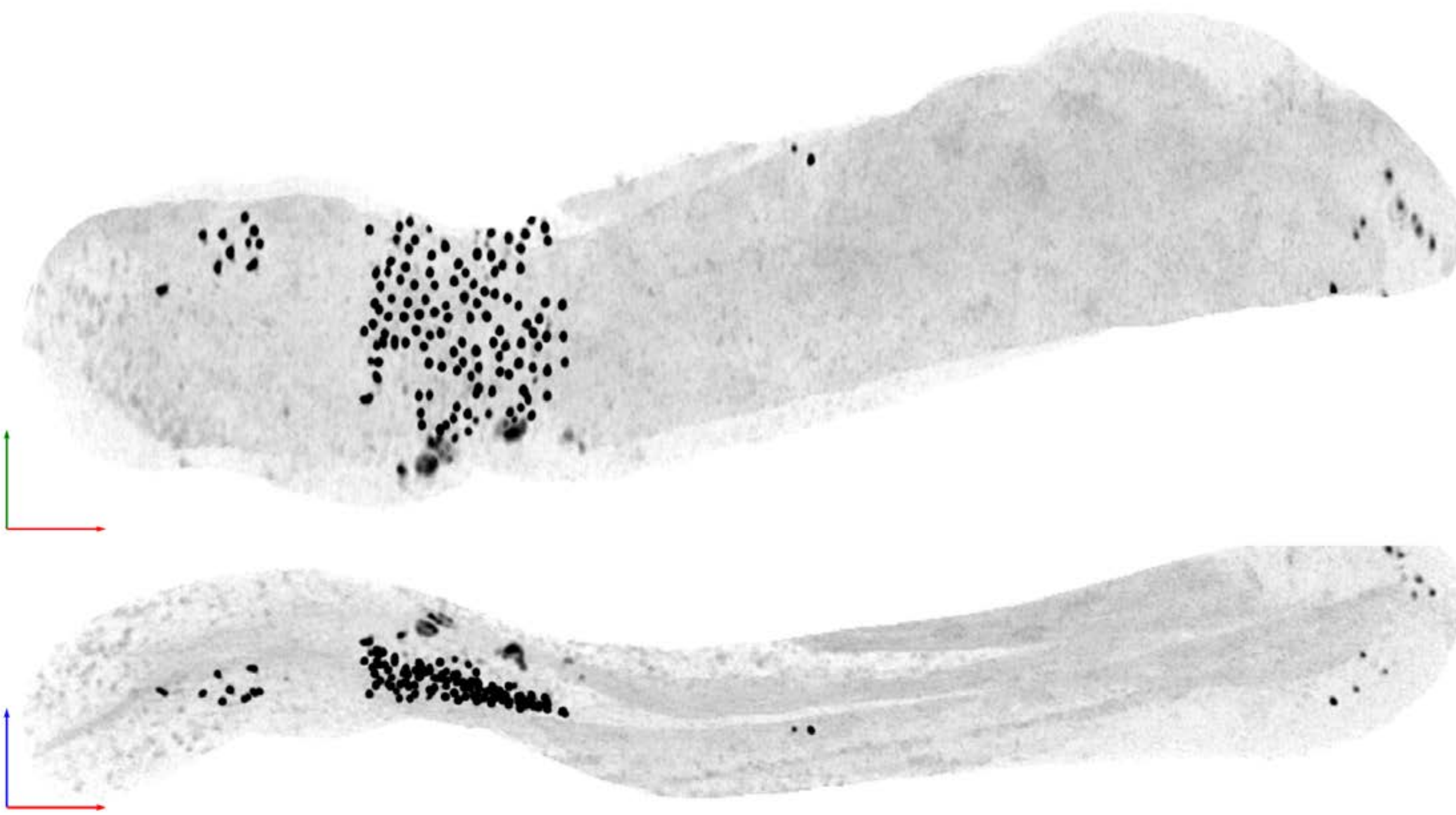

Tomogram ID: 20260127_AMmilled50-3_Position_13_3 | **SET: Dual 15F1 monomer & 5F11 dimer**

Cleft ID: 1 | Type: tissue

Denoised Tomogram Slice

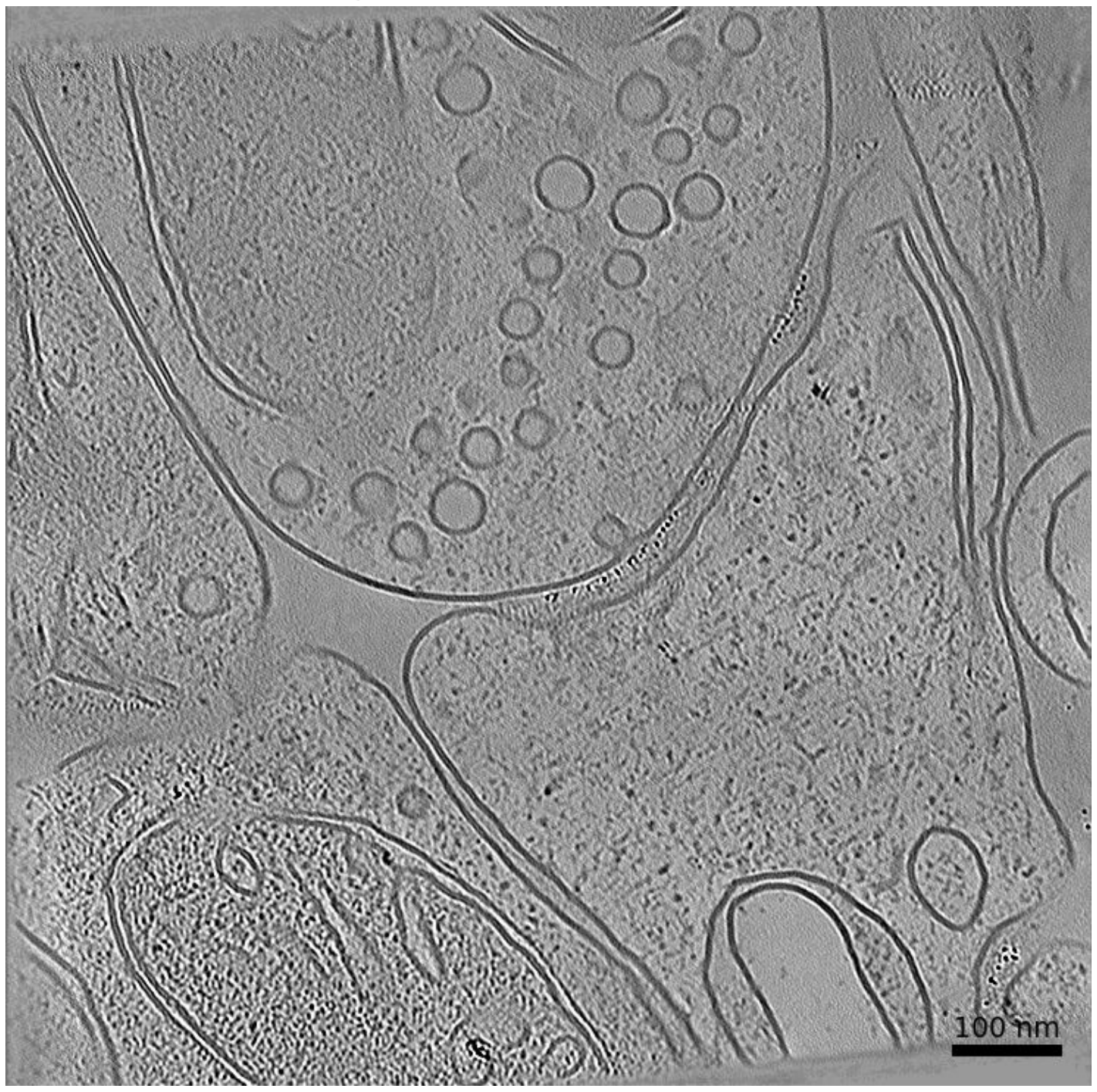

Membrane Segmentation & Cleft Position

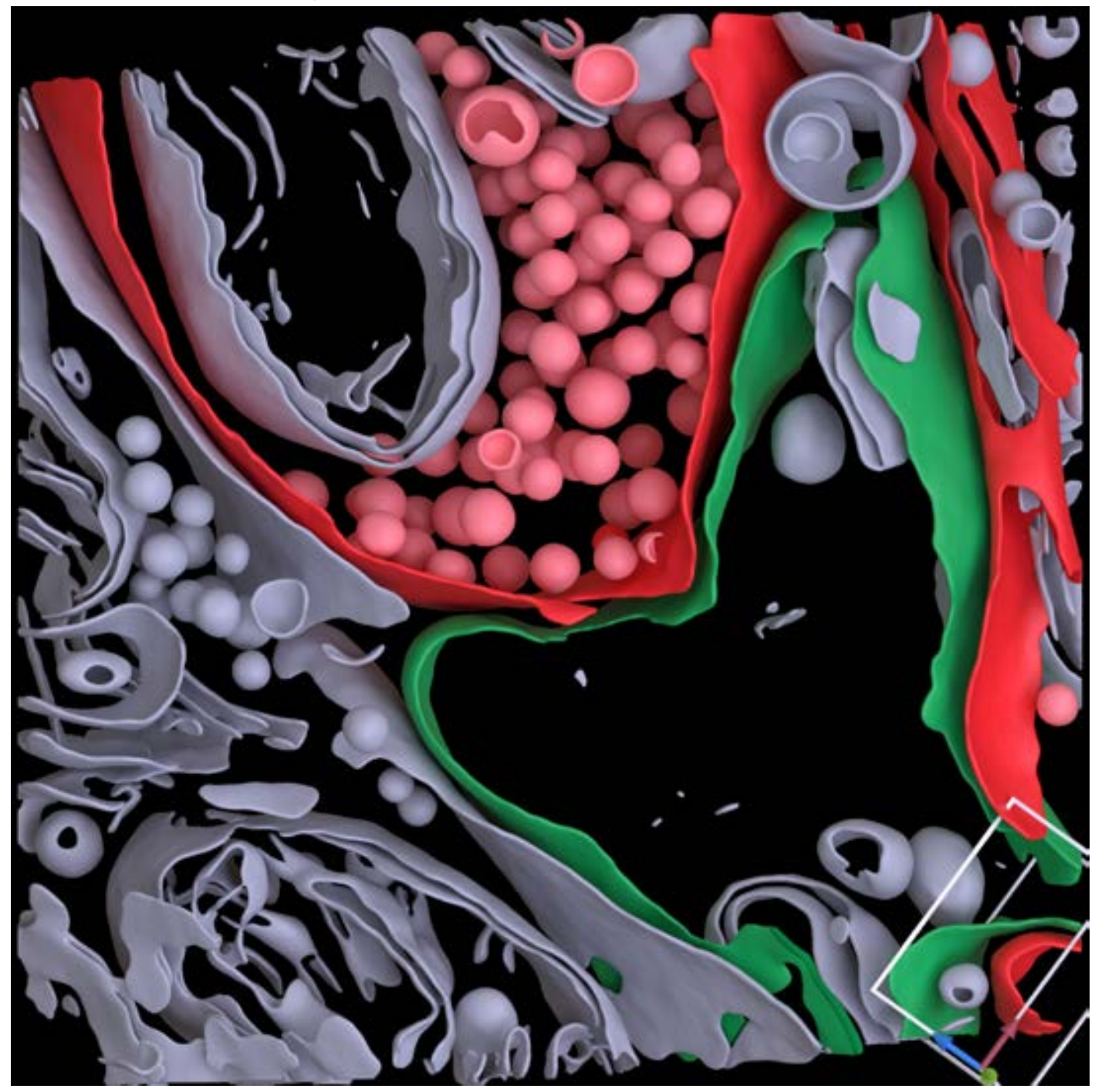

Cleft MIP

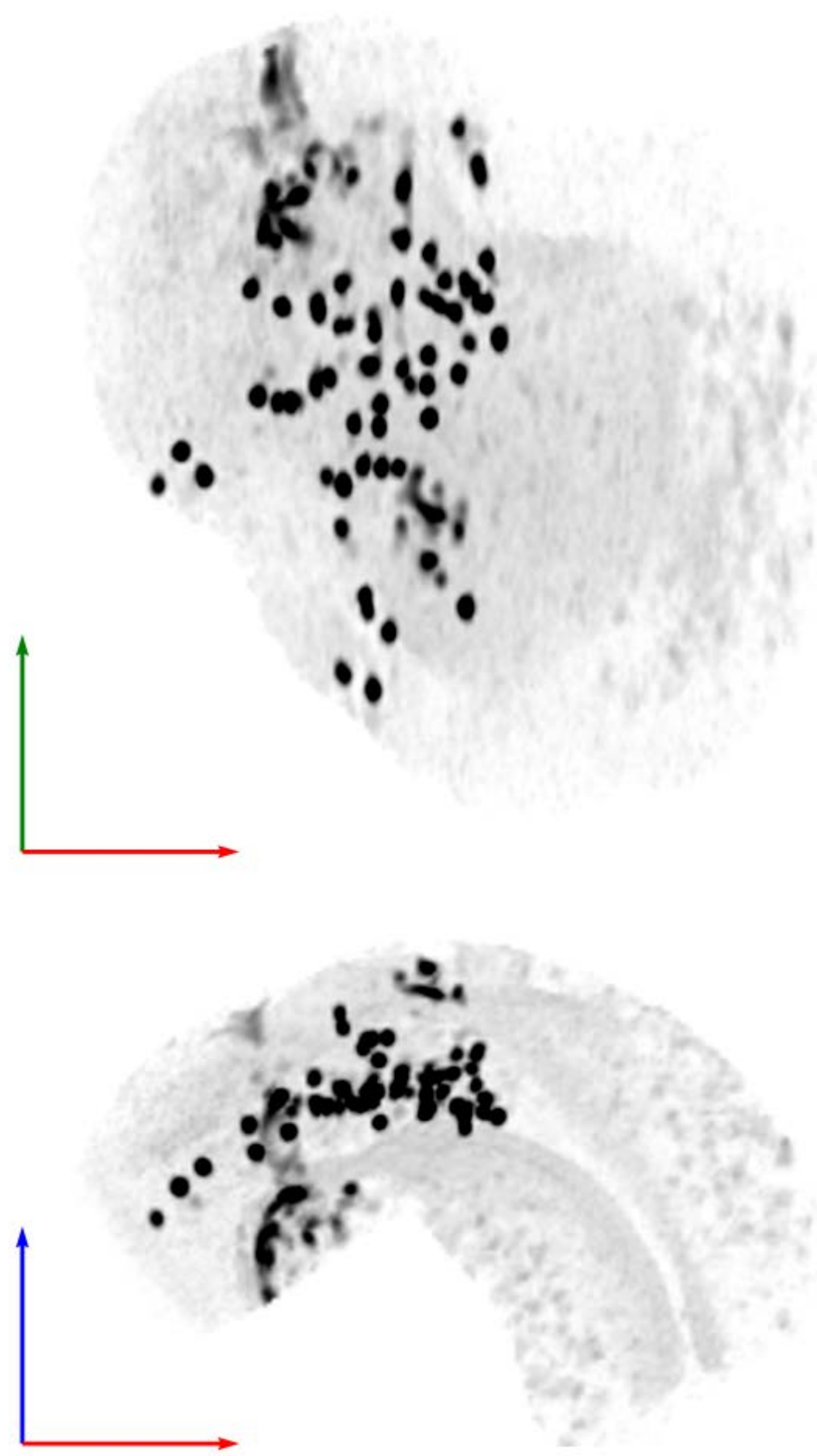

Tomogram ID: 20260127_AMmilled50-3_Position_40 **SET: Dual 15F1 monomer & 5F11 dimer**

Cleft ID: 0 Type: tissue

Denoised Tomogram Slice

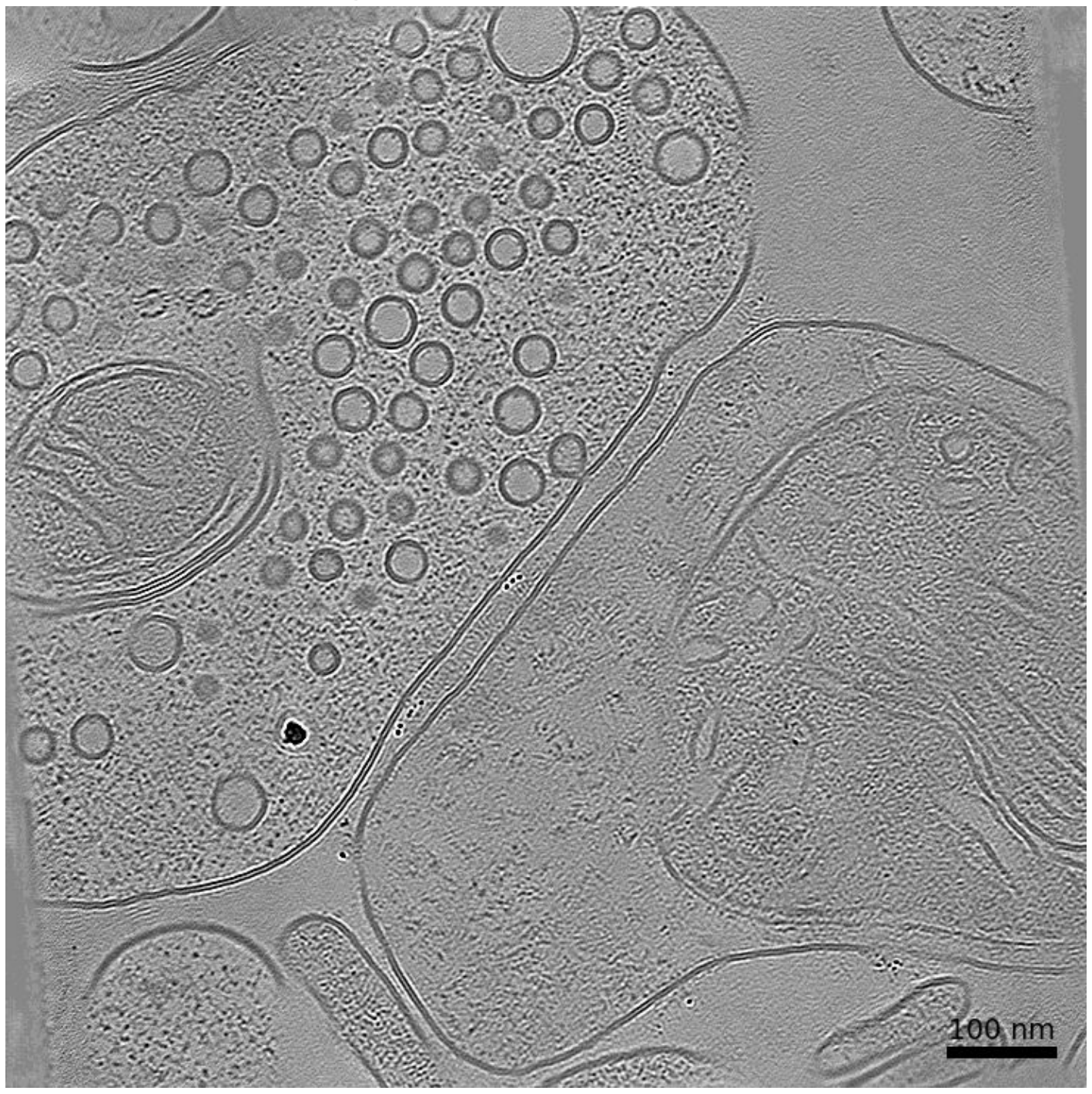

Membrane Segmentation & Cleft Position

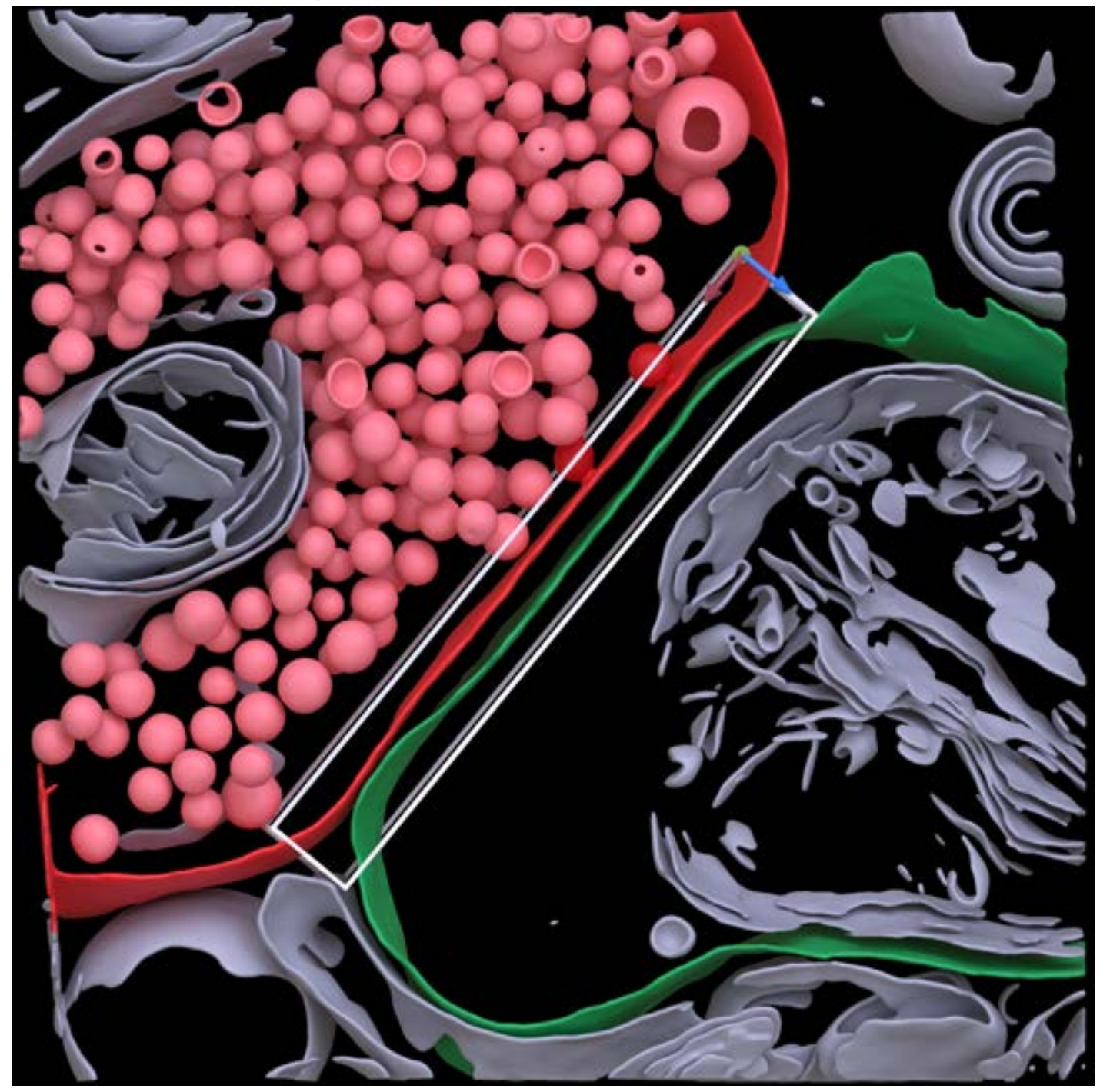

Cleft MIP

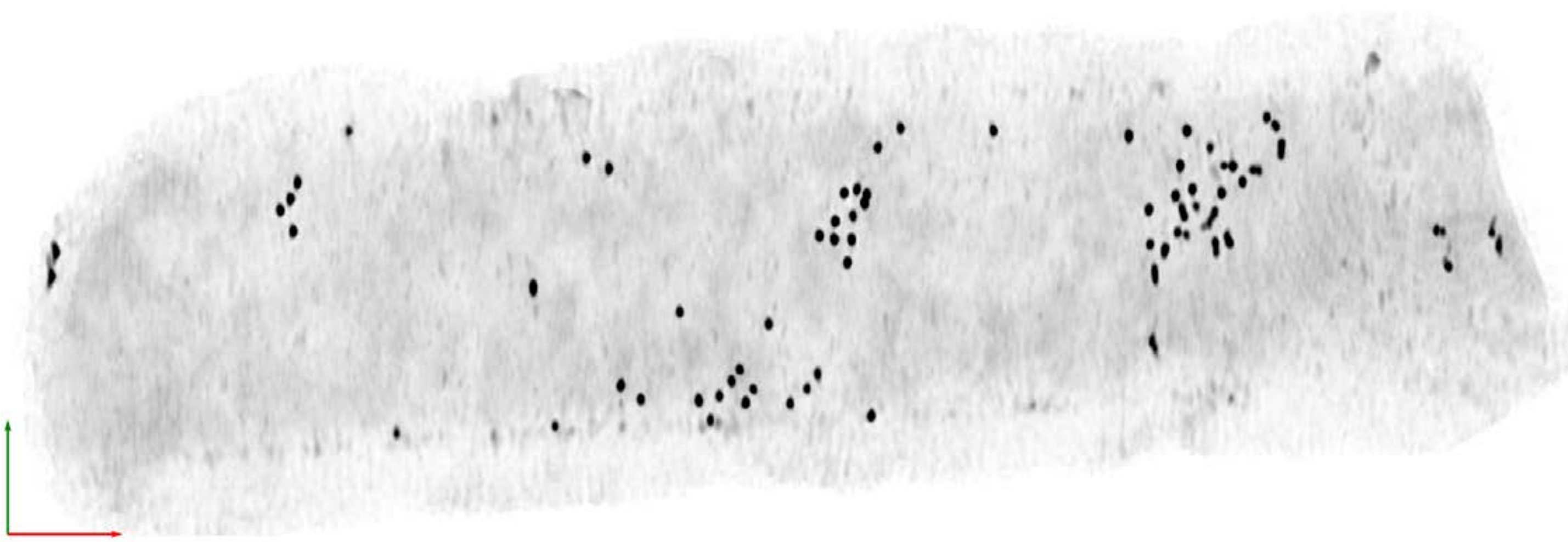

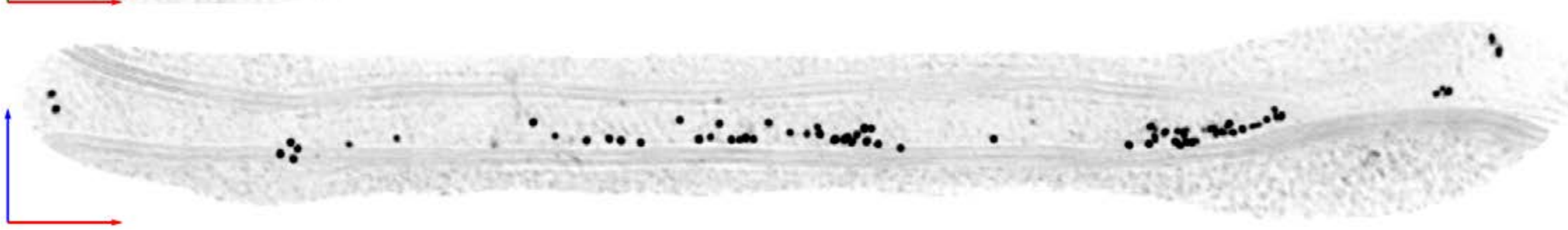

Tomogram ID: 20260127_AMmilled50-3_Position_57_2	SET: Dual 15F1 monomer & 5F11 dimer
Cleft ID: 0	Type: semi-tissue

Denoised Tomogram Slice

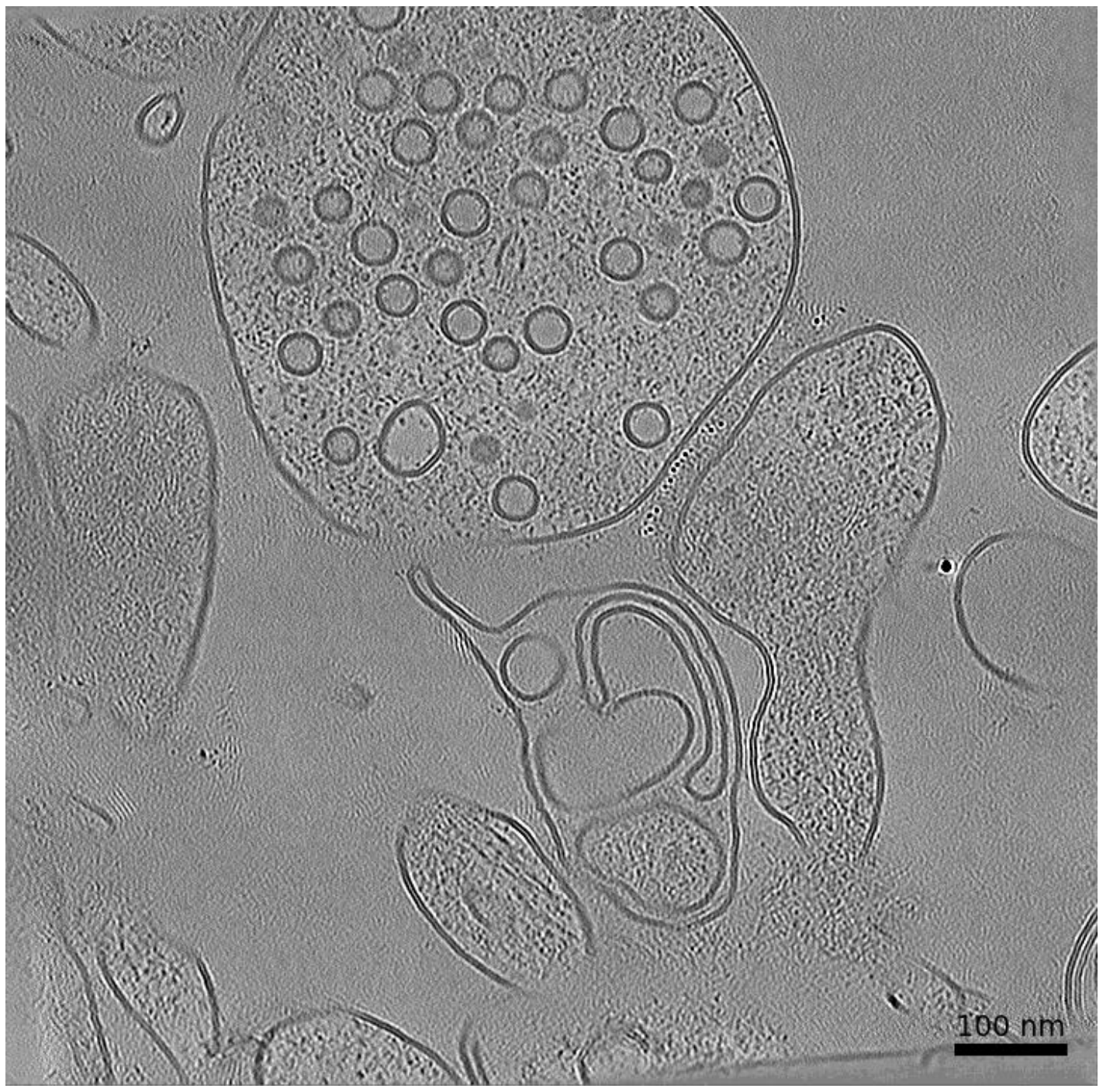

Membrane Segmentation & Cleft Position

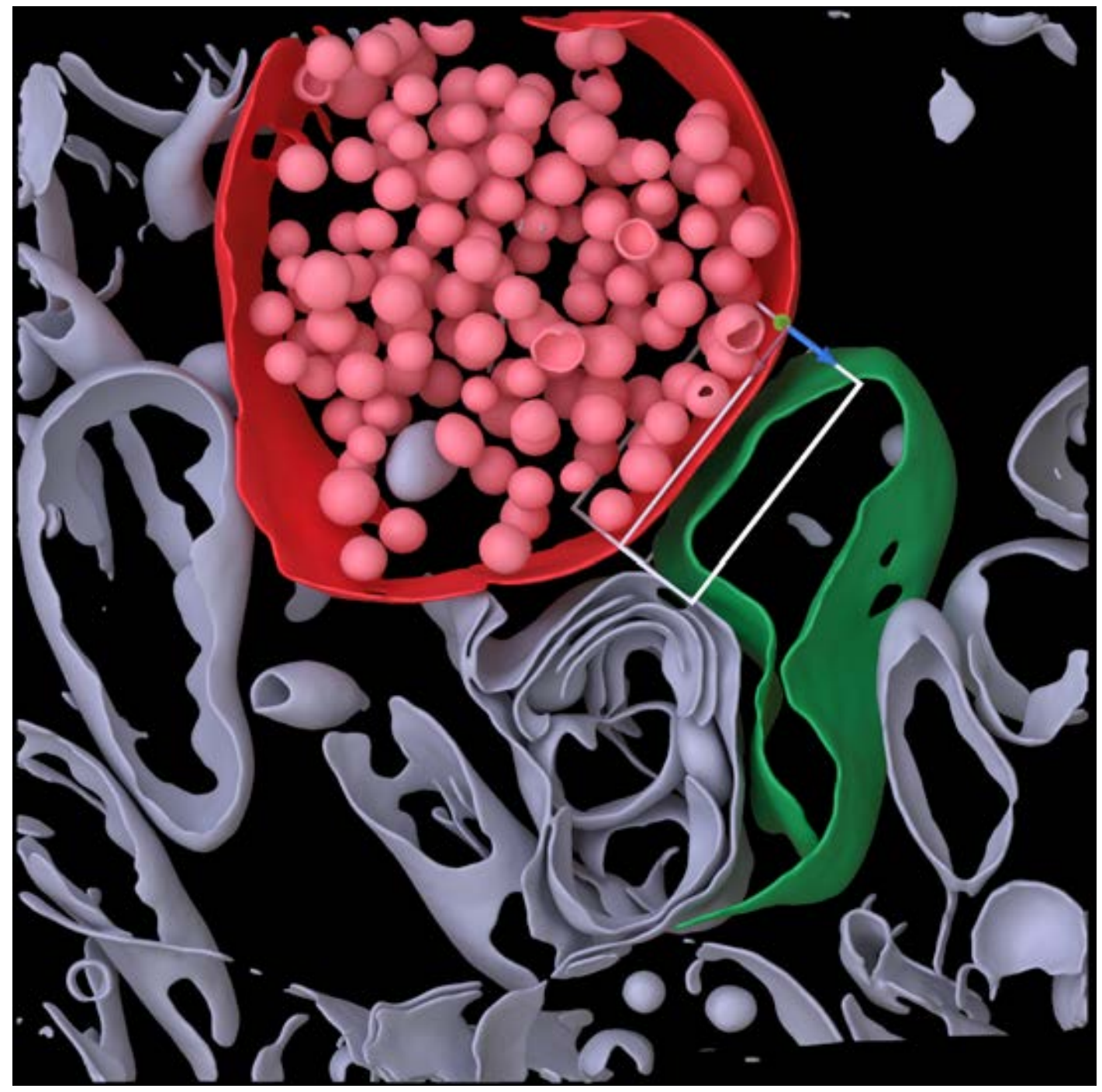

Cleft MIP

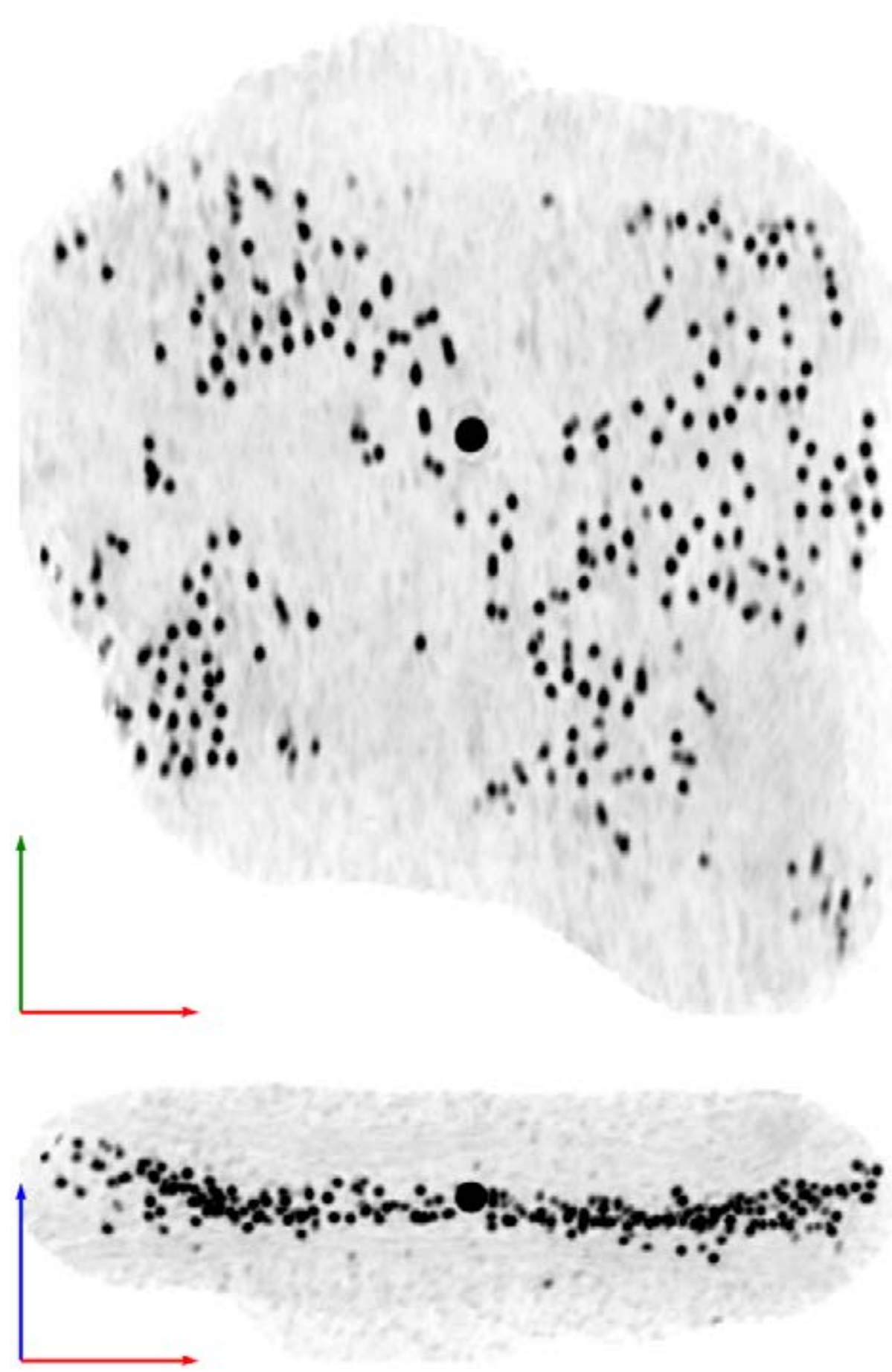

Tomogram ID: 20260128_AMmilled50-2_Position_2_2	SET: Dual 15F1 monomer & 5F11 dimer
Cleft ID: 0	Type: tissue

Denoised Tomogram Slice

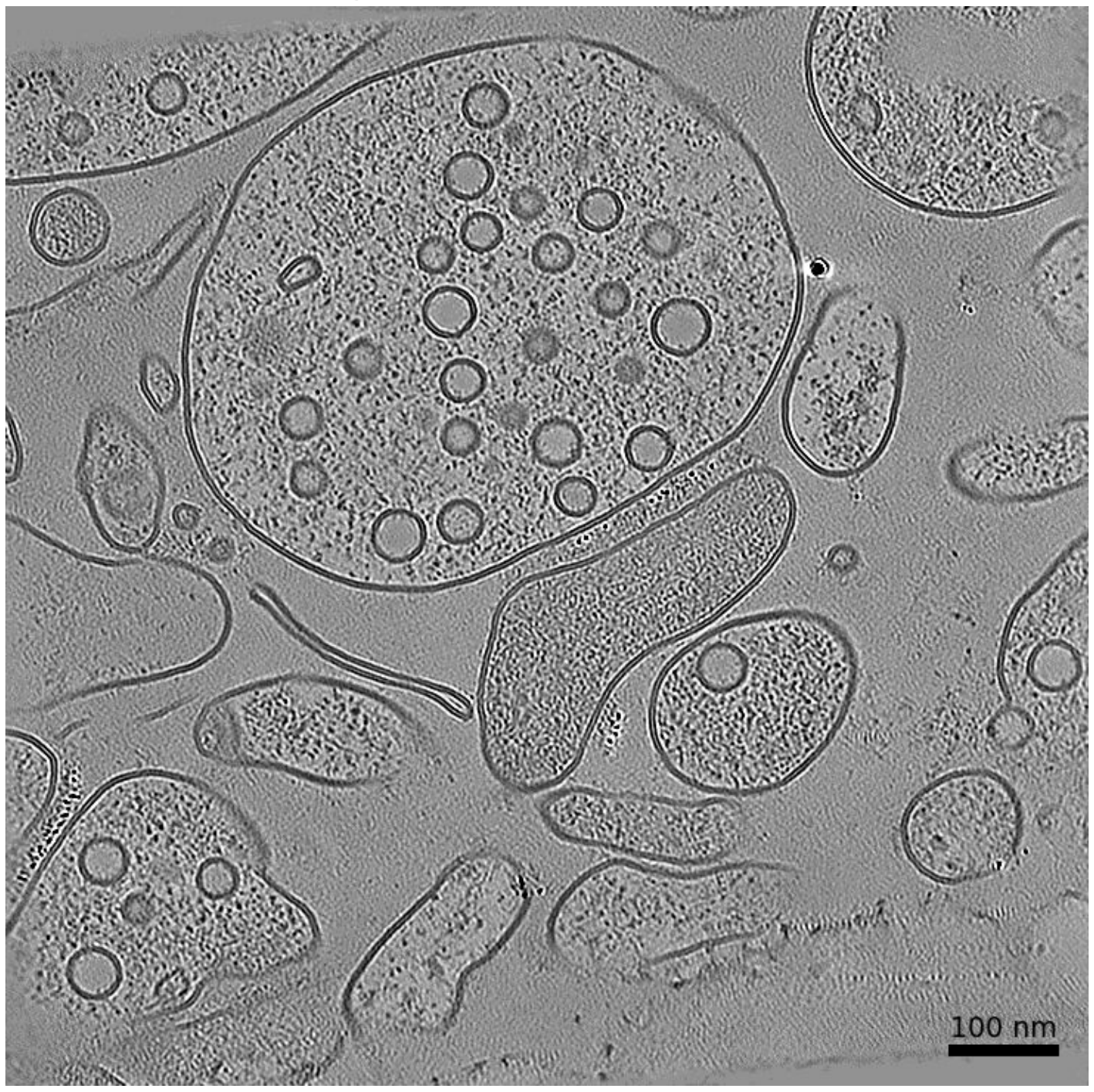

Membrane Segmentation & Cleft Position

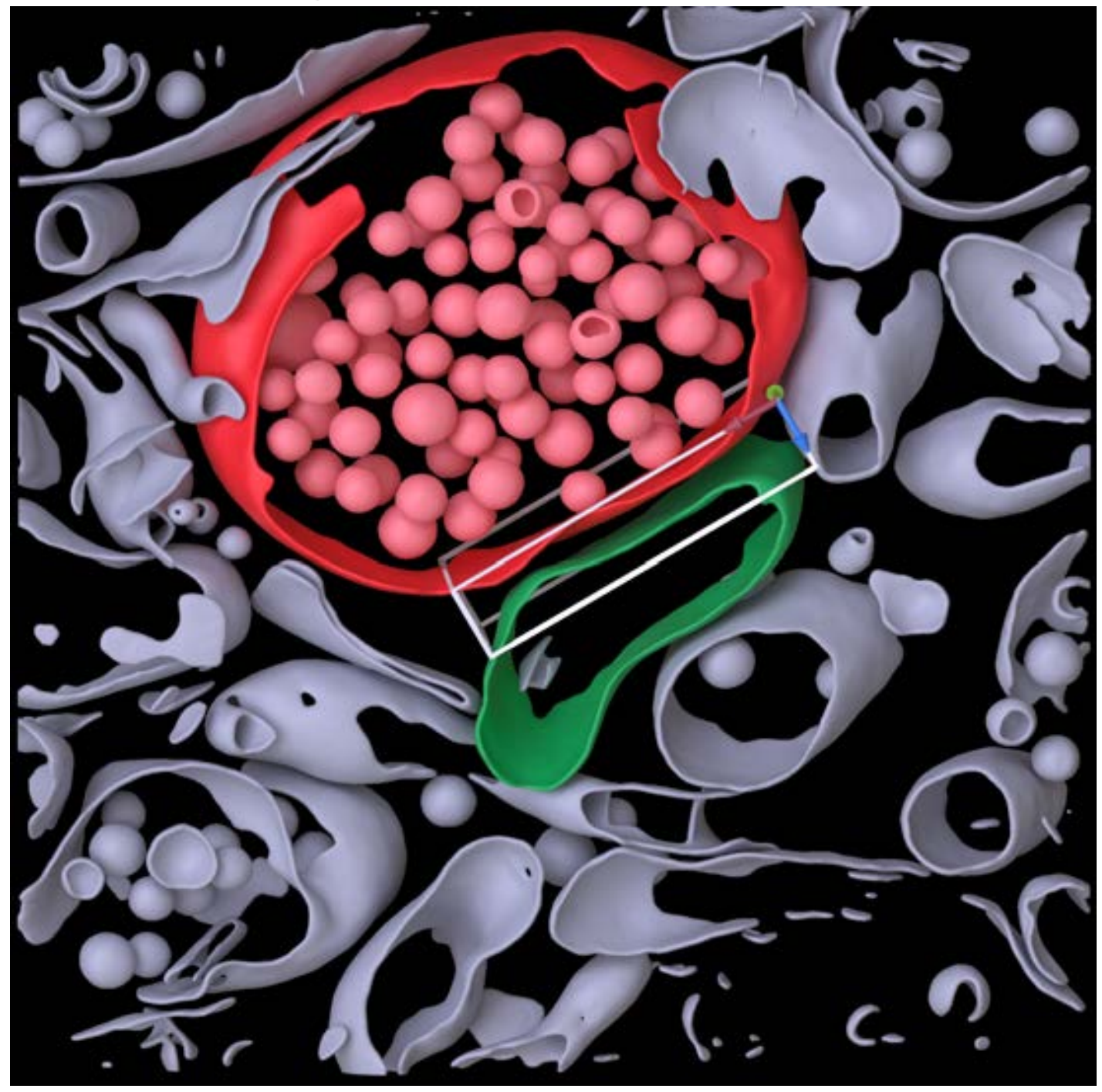

Cleft MIP

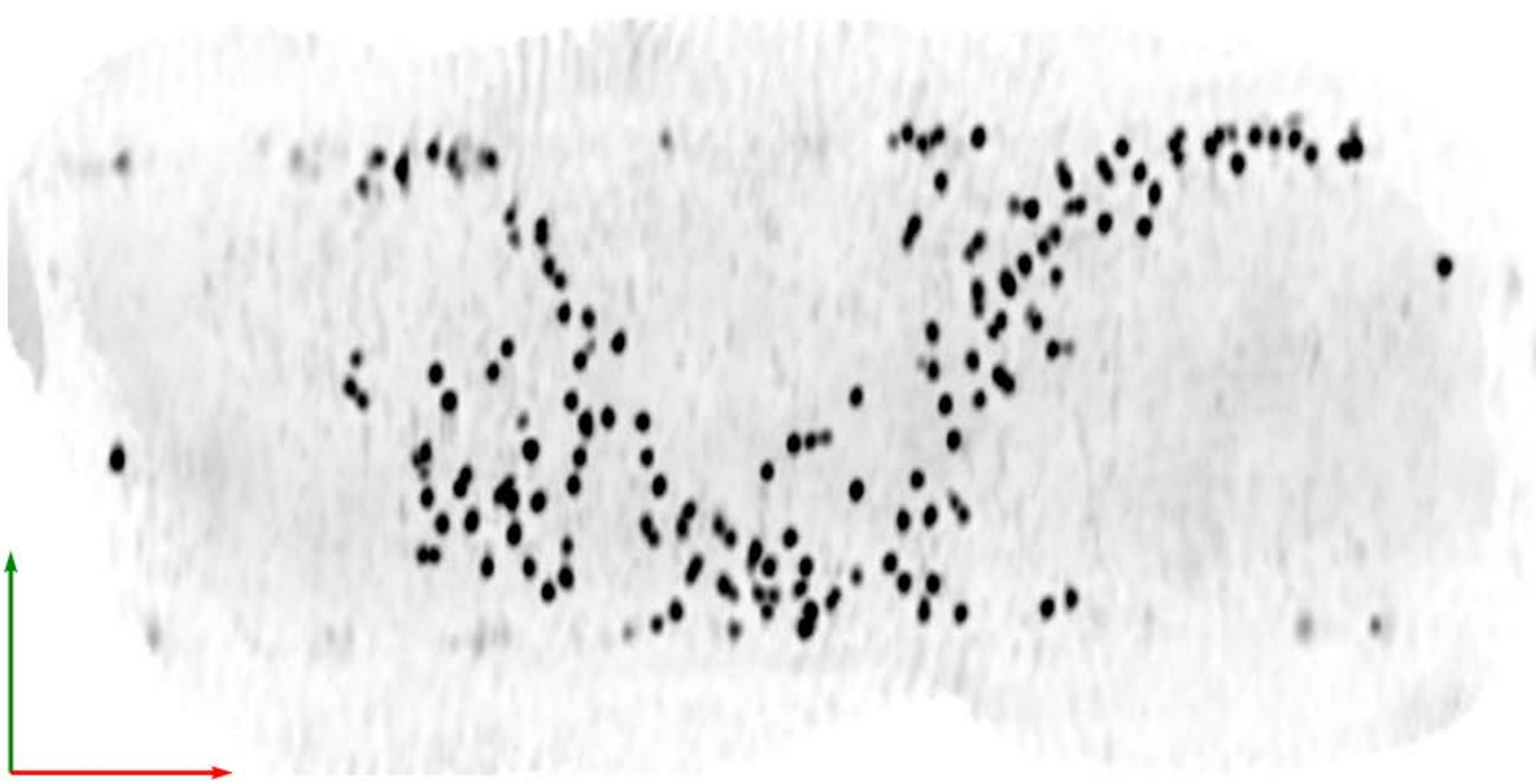

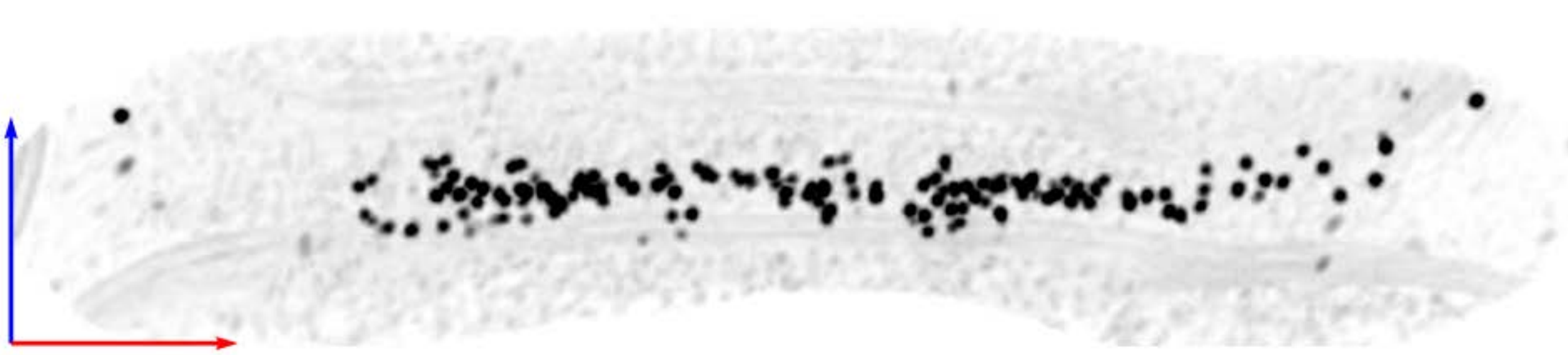

**Tomogram ID: 20250227_AMmilled25-3_Position_16**	**SET: no labeling**
Cleft ID: 0	Type: tissue

Denoised Tomogram Slice

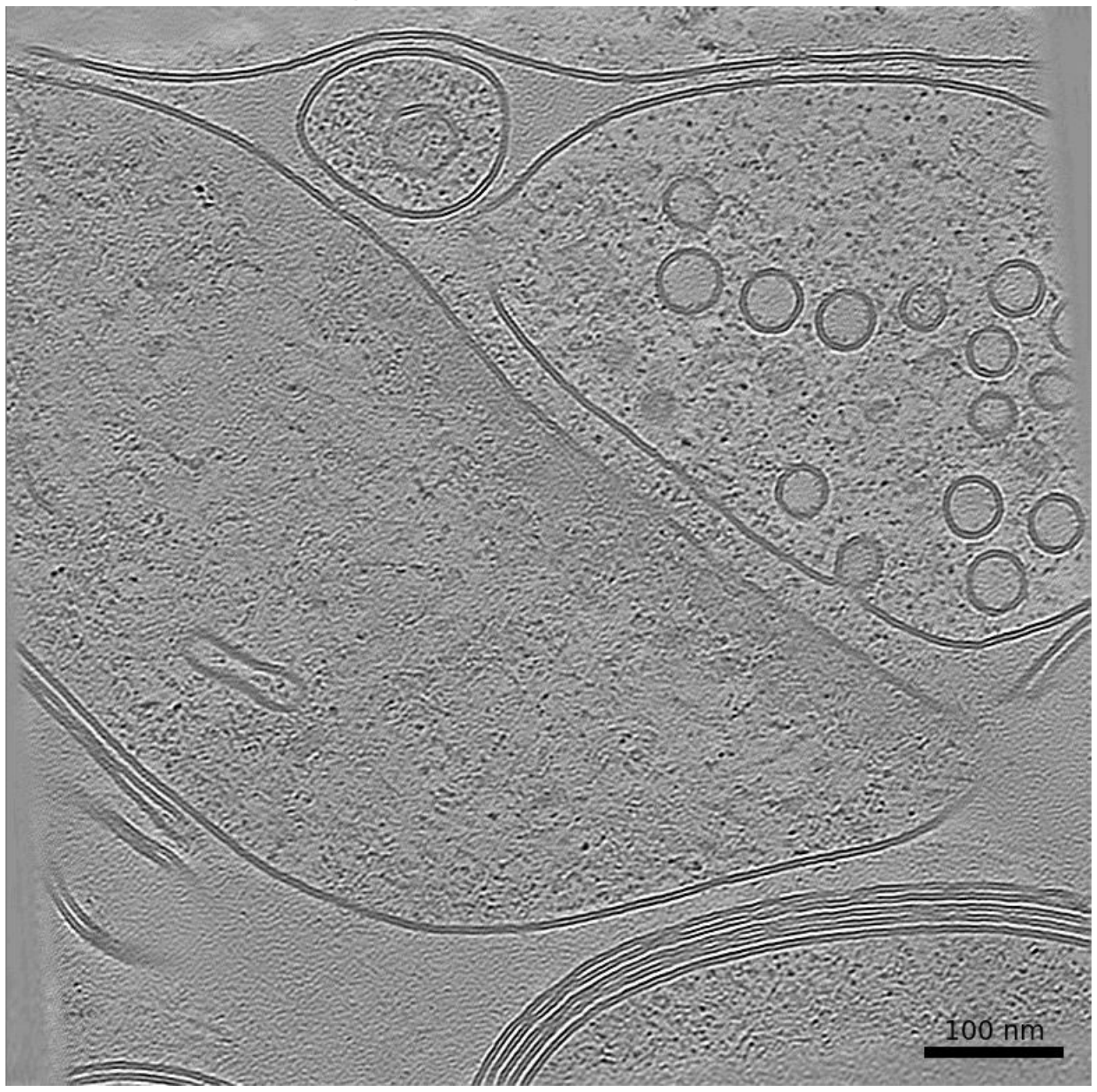

Membrane Segmentation & Cleft Position

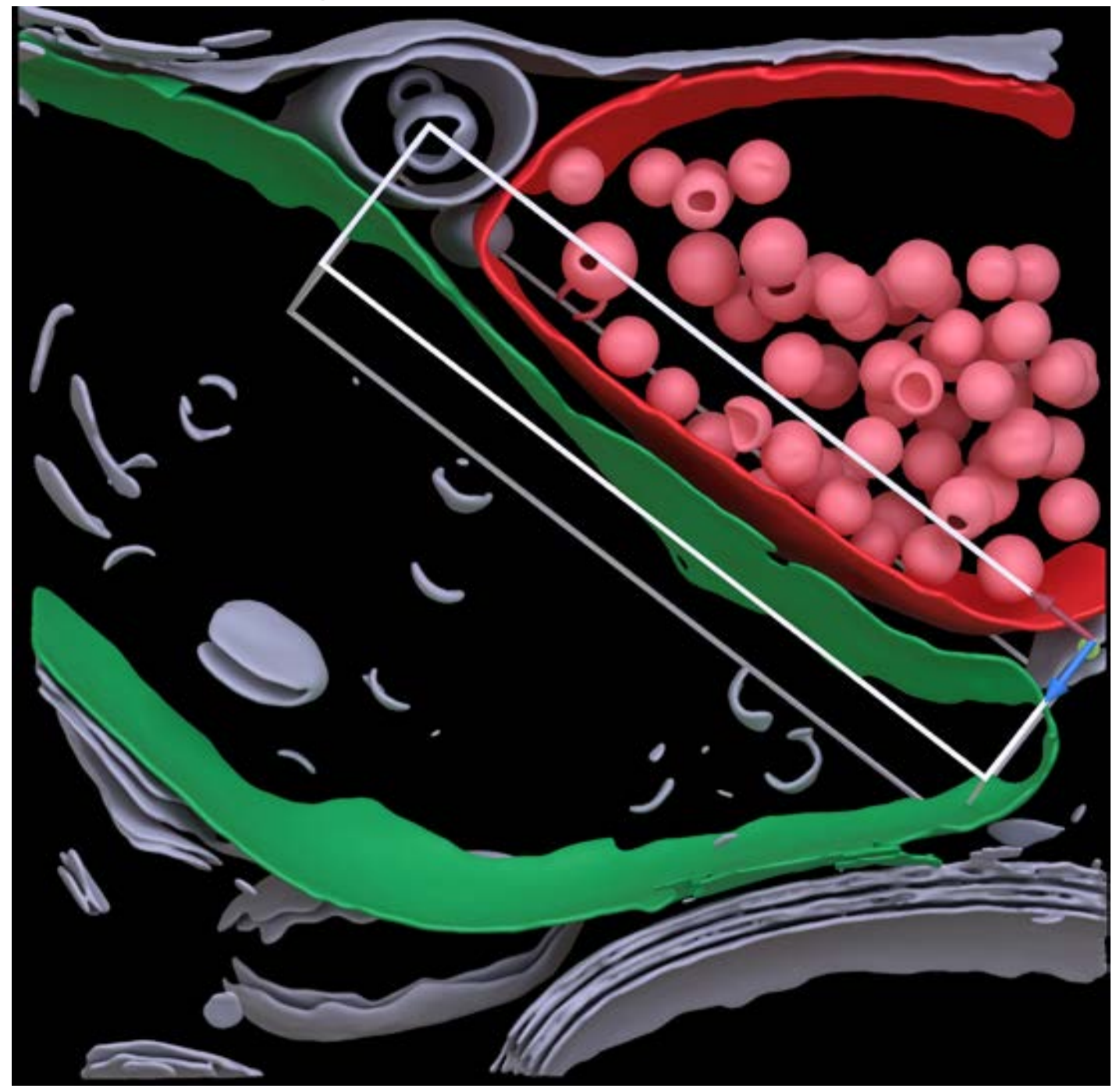

Cleft MIP

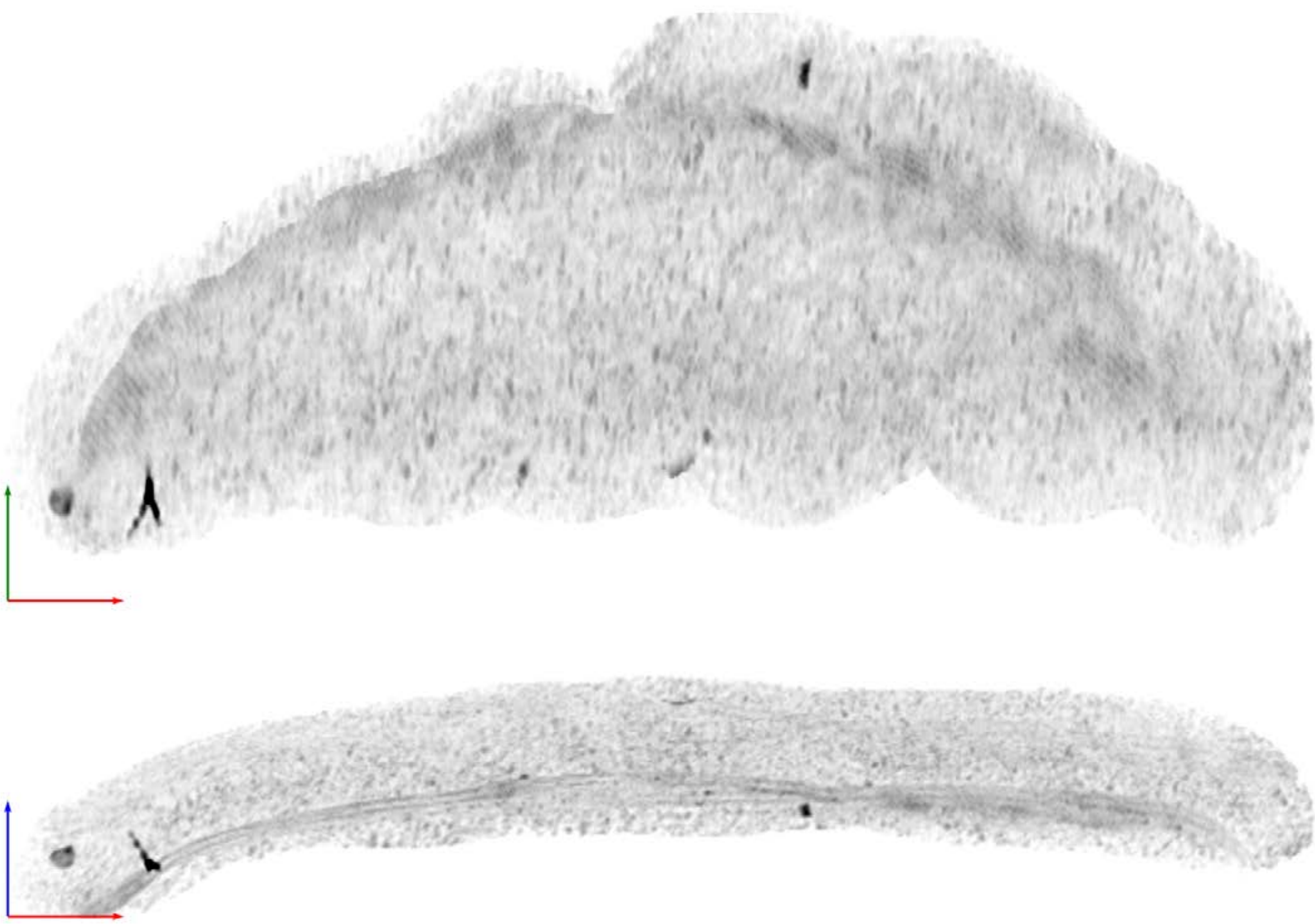

Tomogram ID: 20250227_AMmilled25-3_Position_64 **SET: no labeling**

Cleft ID: 0 Type: tissue

Denoised Tomogram Slice

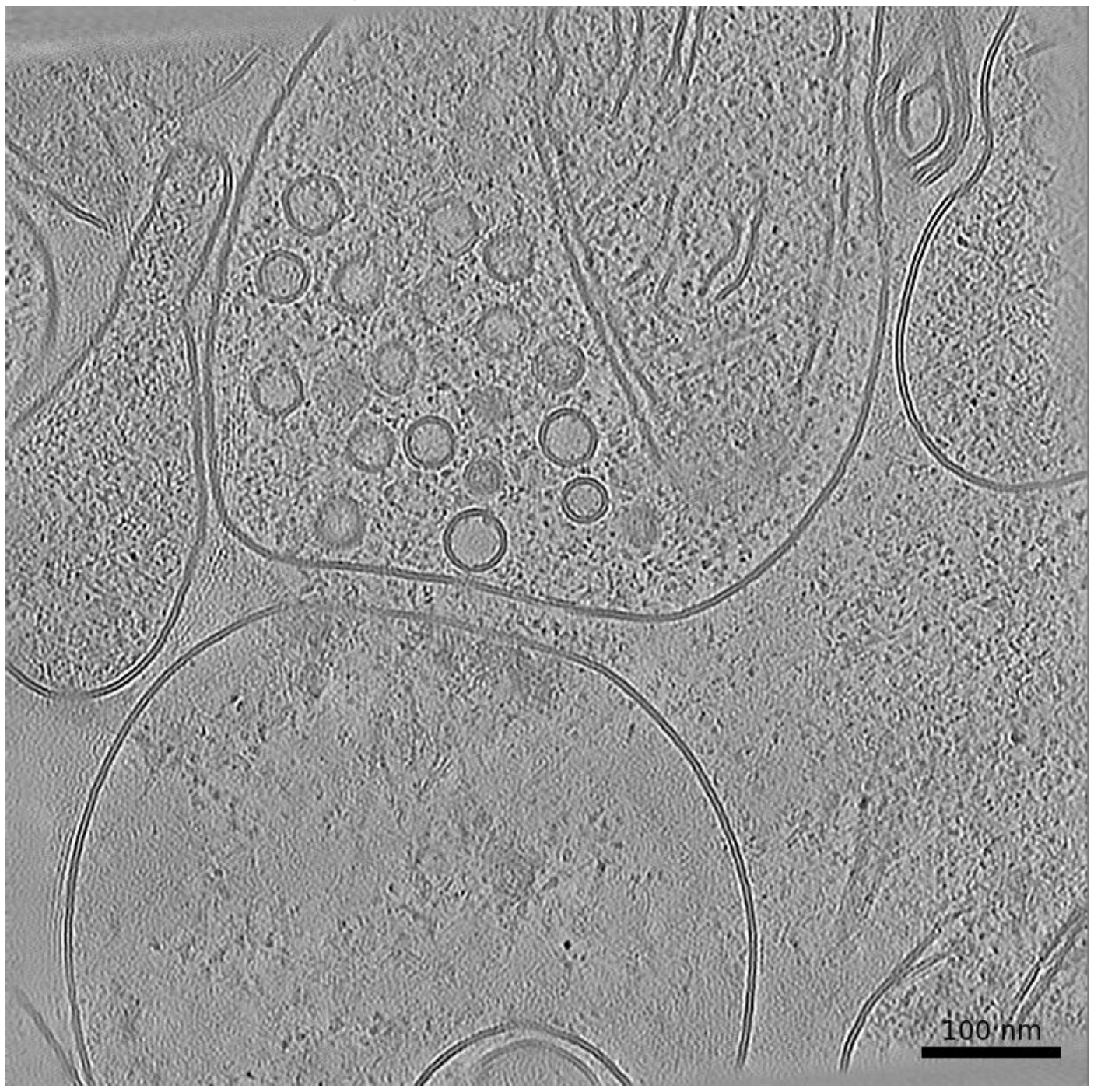

Membrane Segmentation & Cleft Position

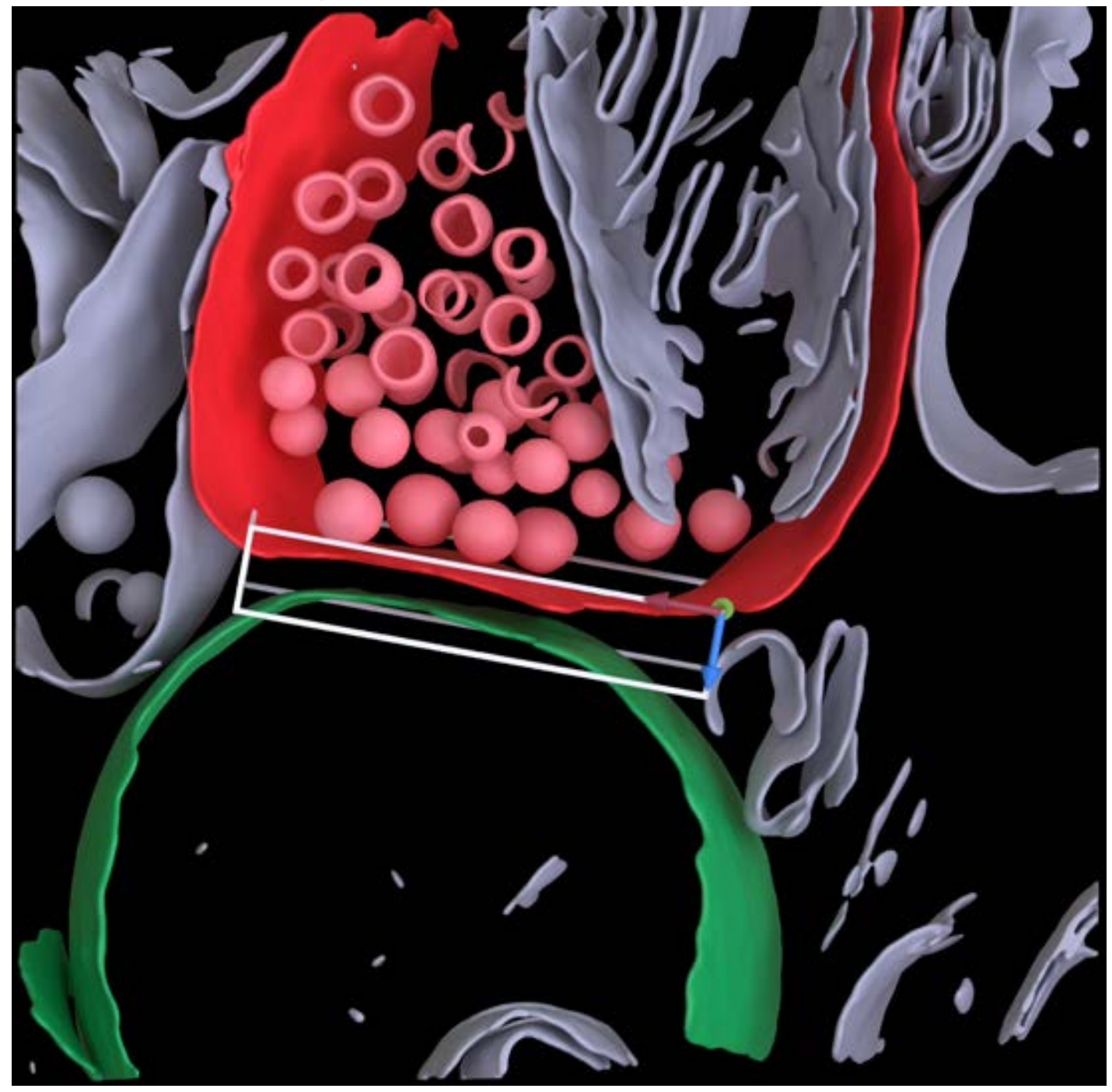

Cleft MIP

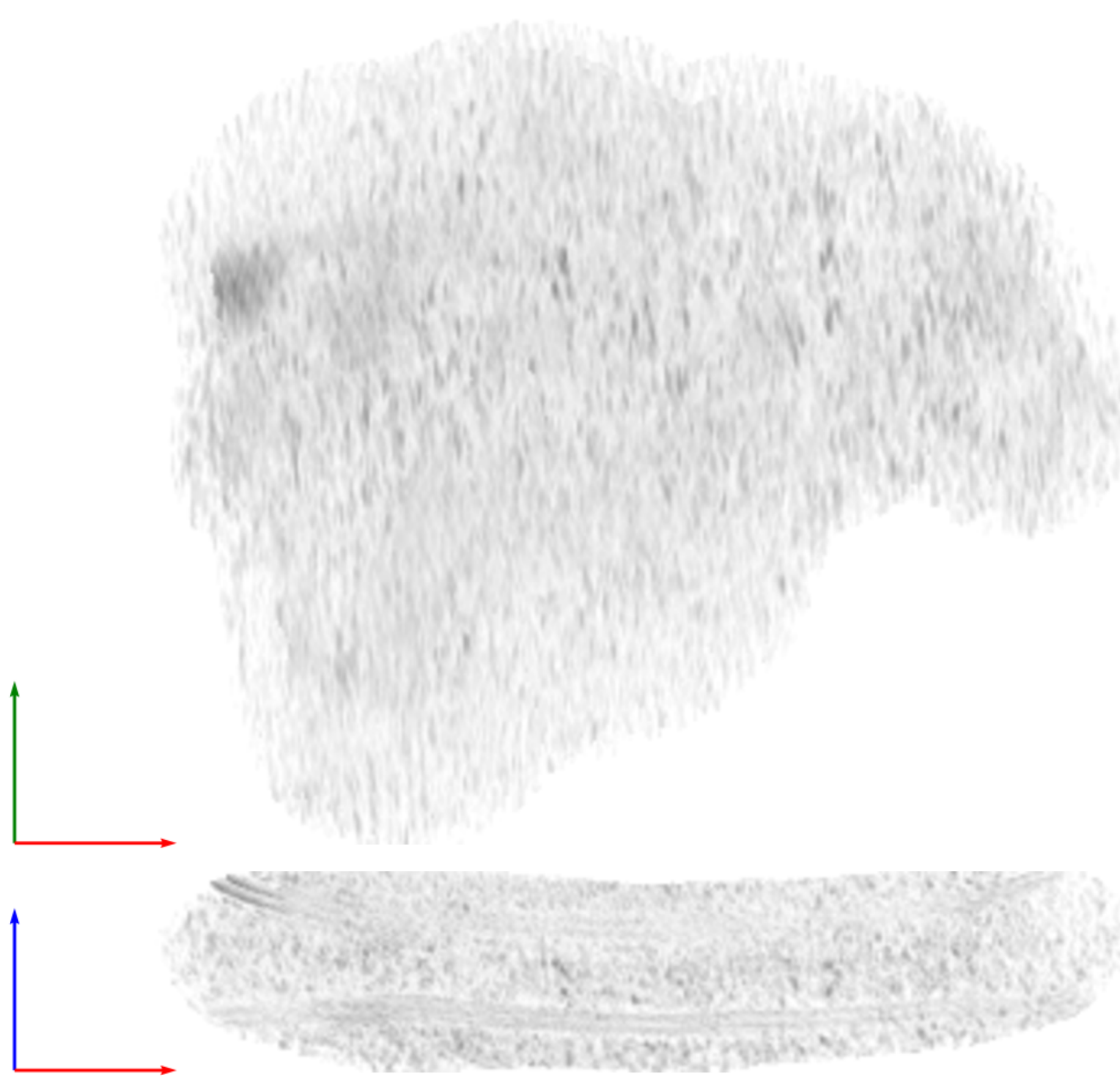

Tomogram ID: 20250227_AMmilled25-3_Position_69_3 | **SET: no labeling**

Cleft ID: 0 | Type: tissue

Denoised Tomogram Slice

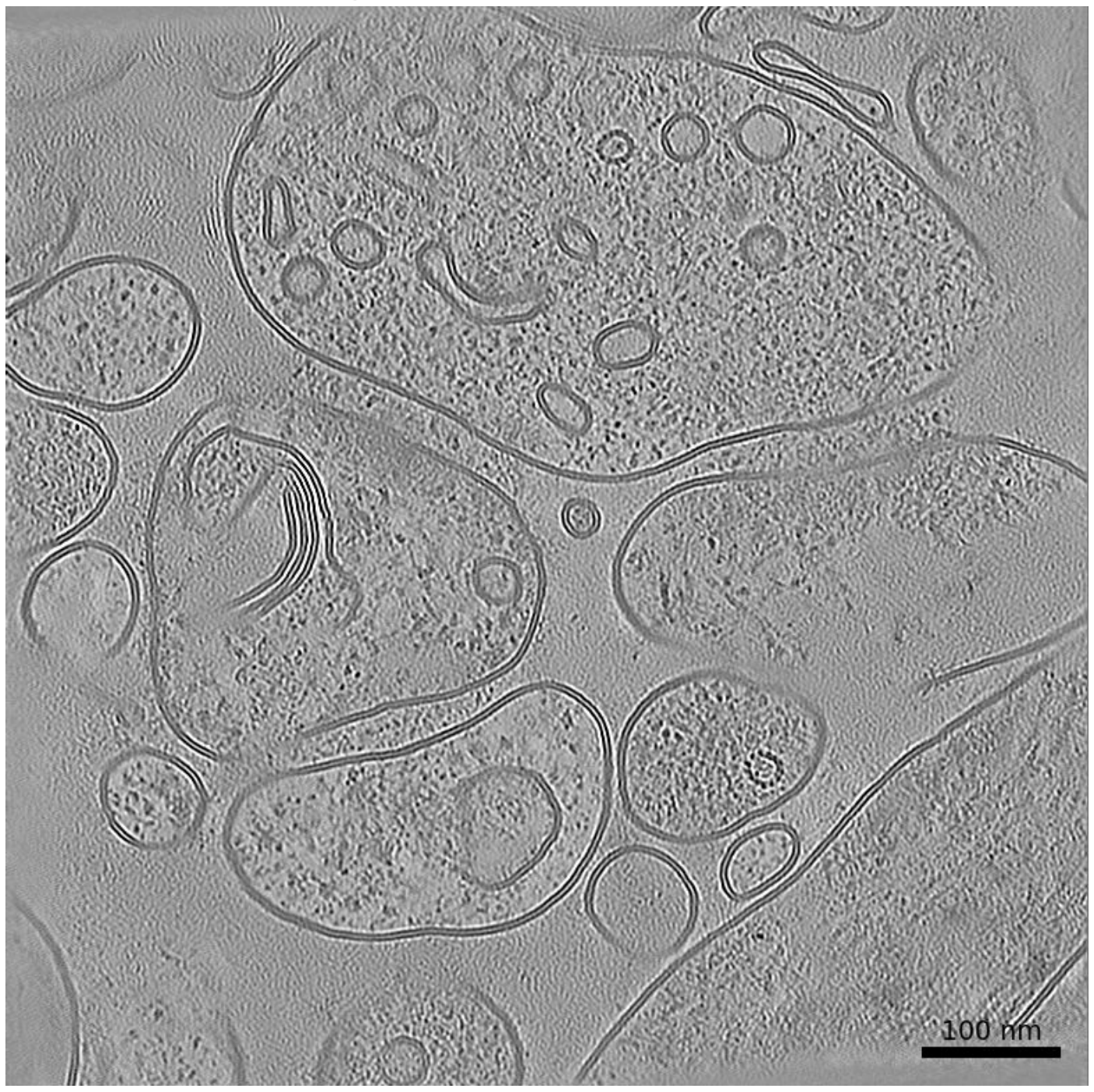

Membrane Segmentation & Cleft Position

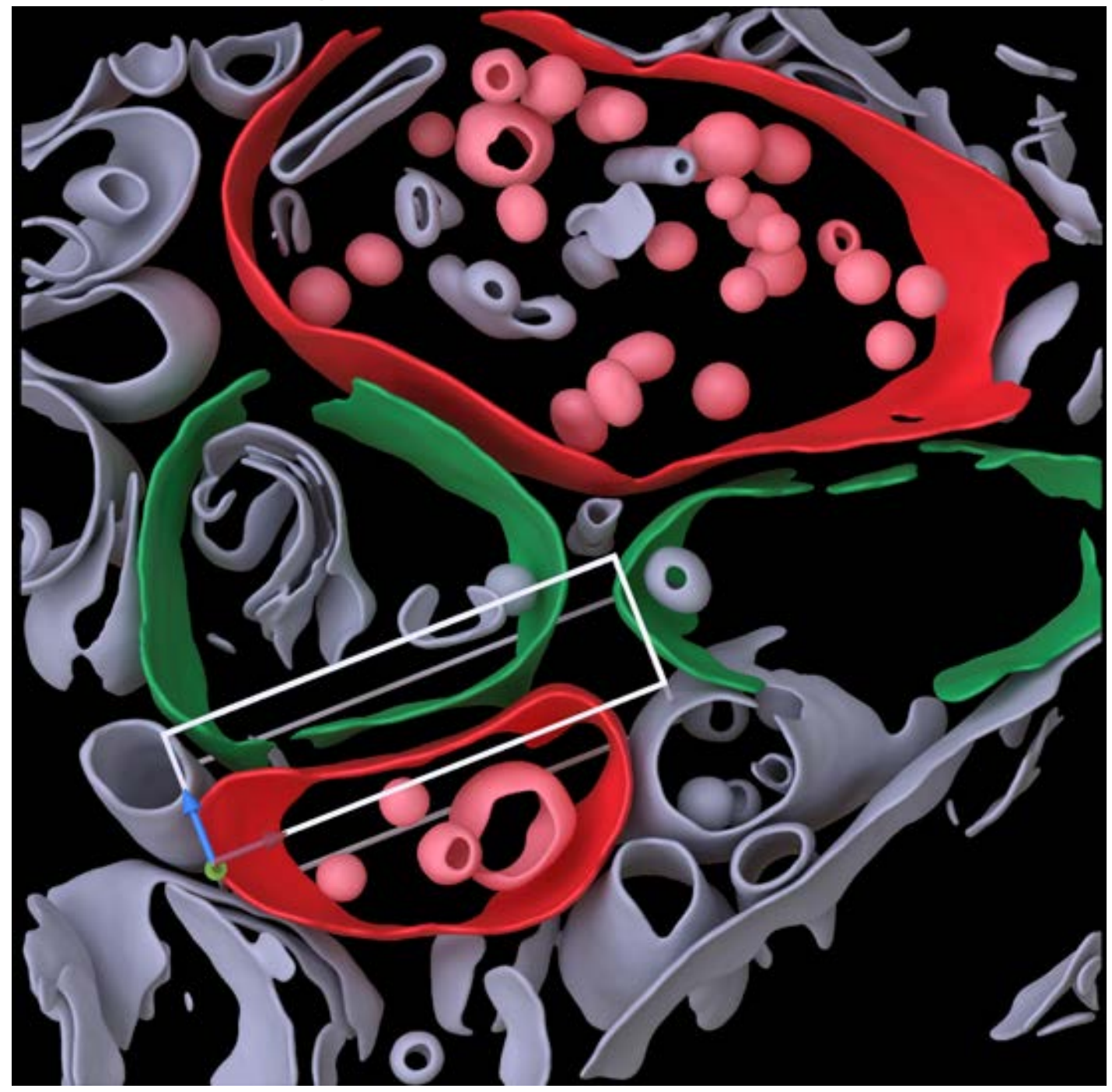

Cleft MIP

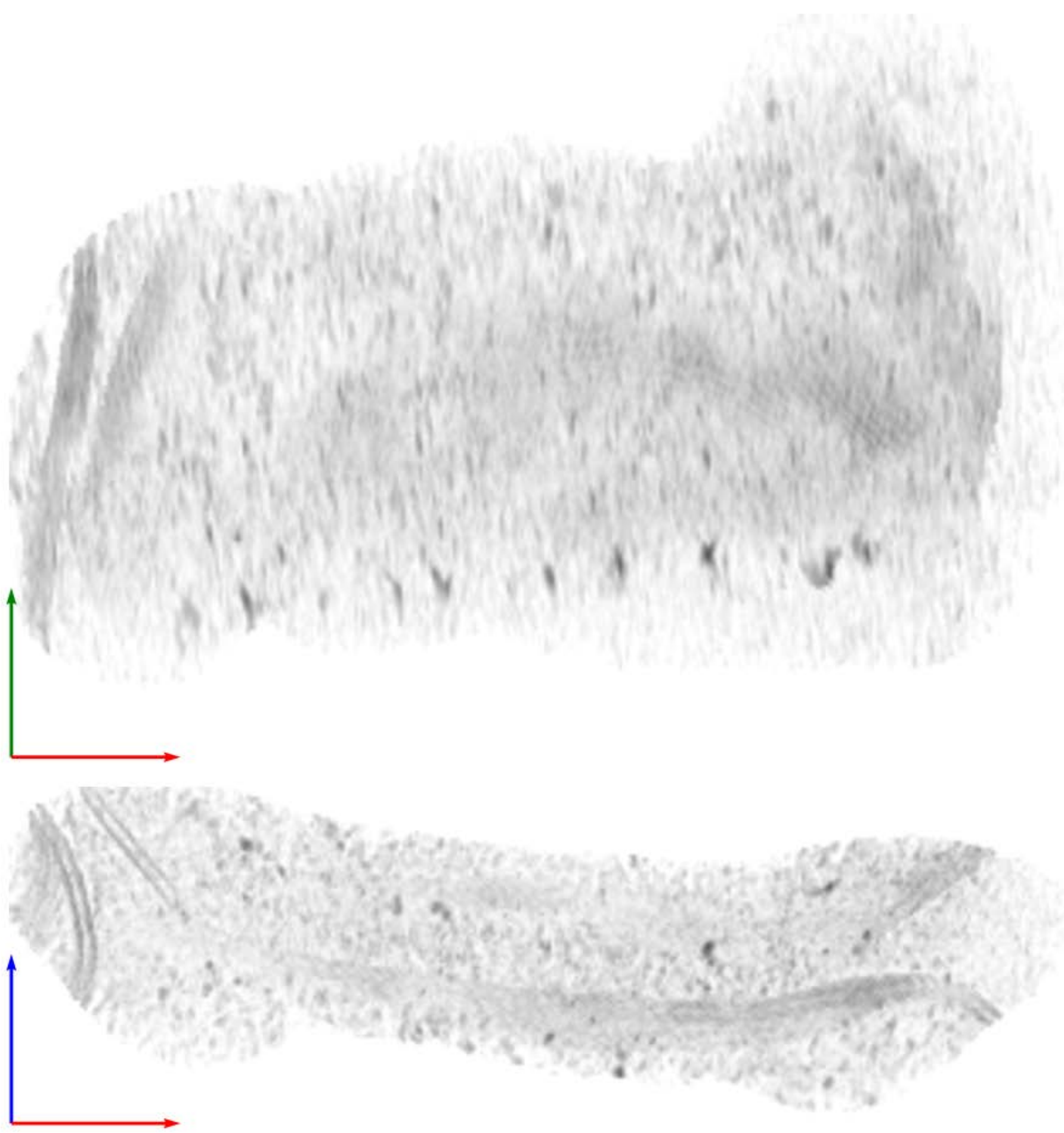

Tomogram ID: 20250227_AMmilled25-3_Position_69_3 **SET: no labeling**

Cleft ID: 1 Type: tissue

Denoised Tomogram Slice

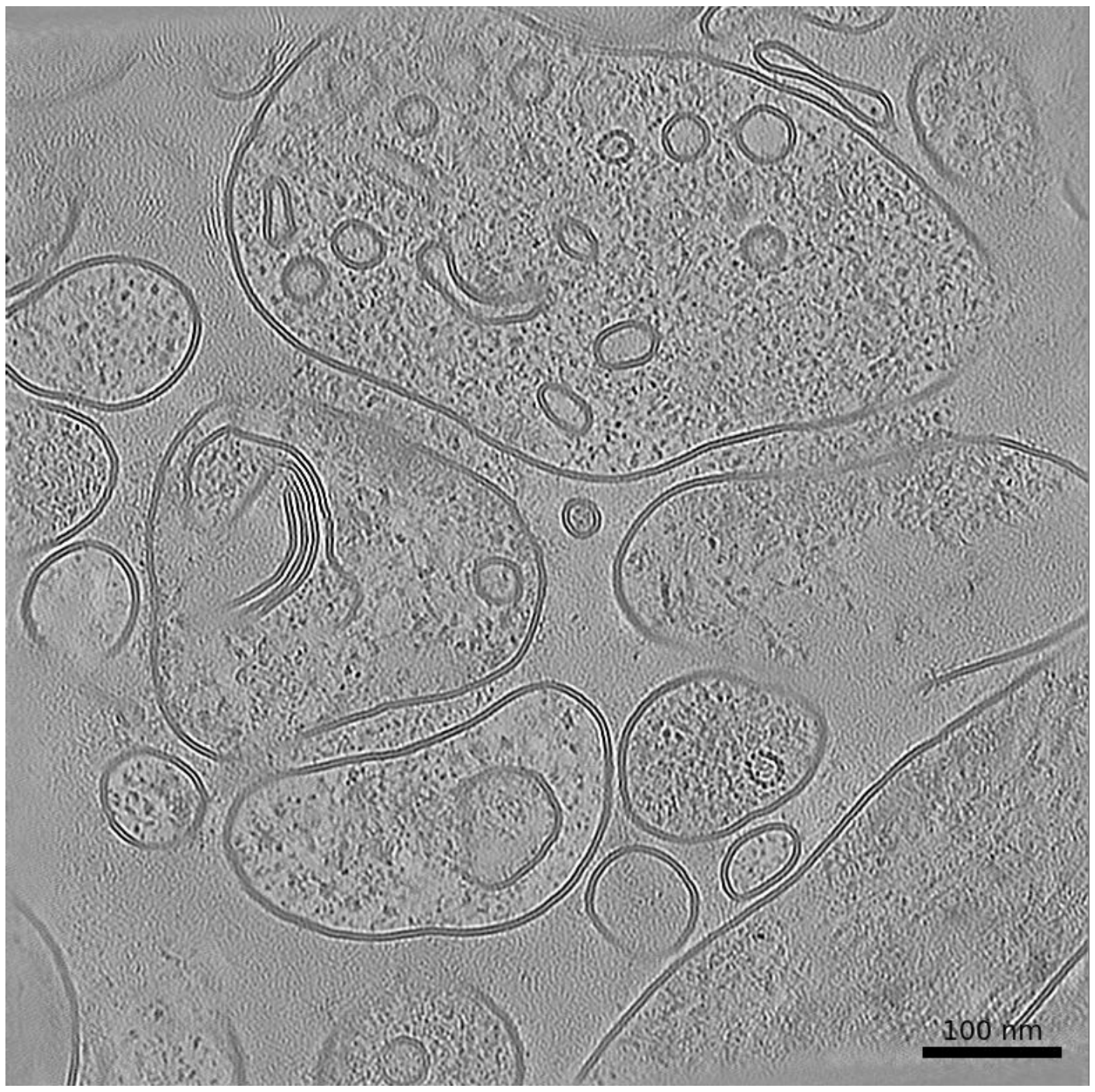

Membrane Segmentation & Cleft Position

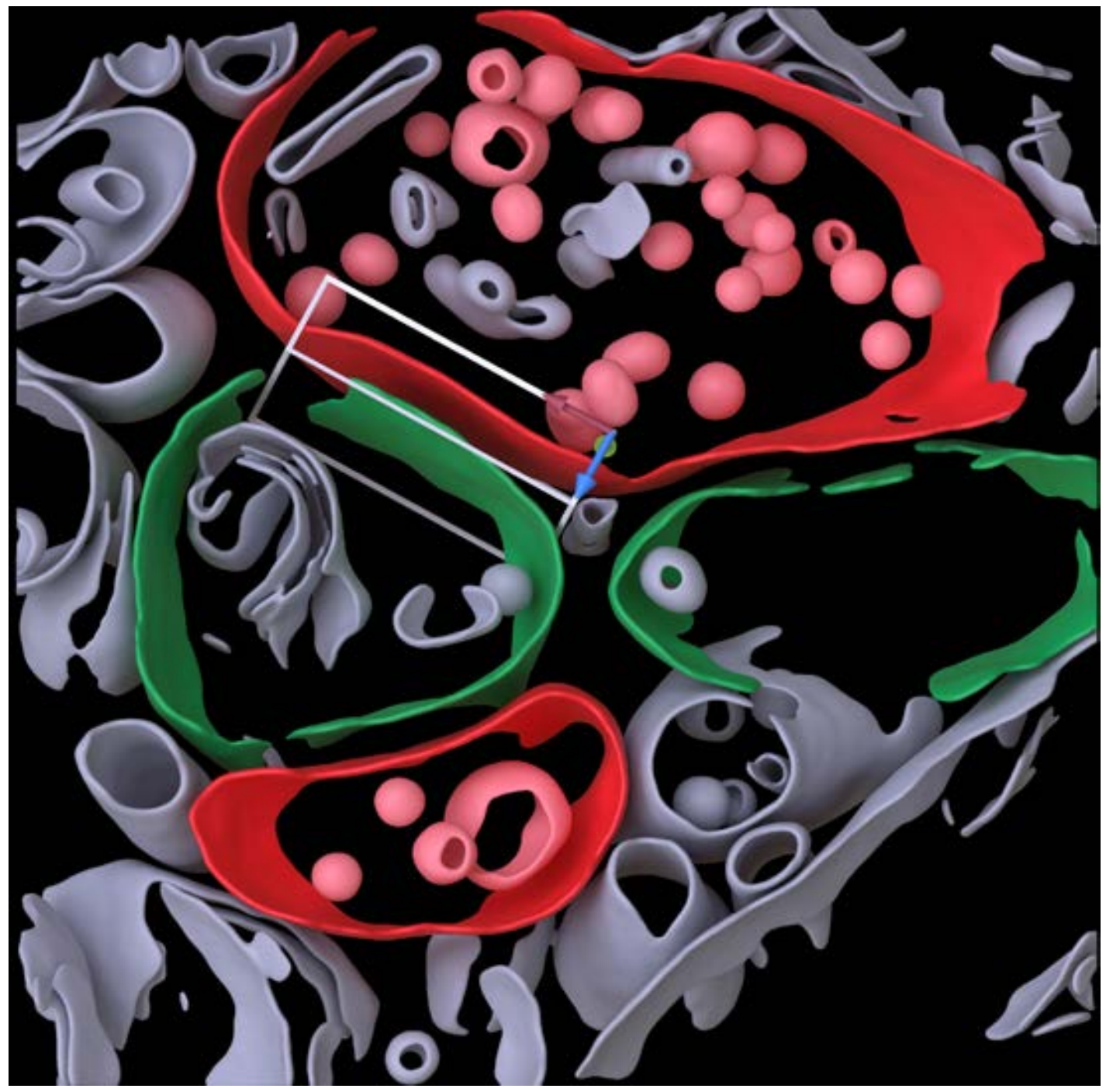

Cleft MIP

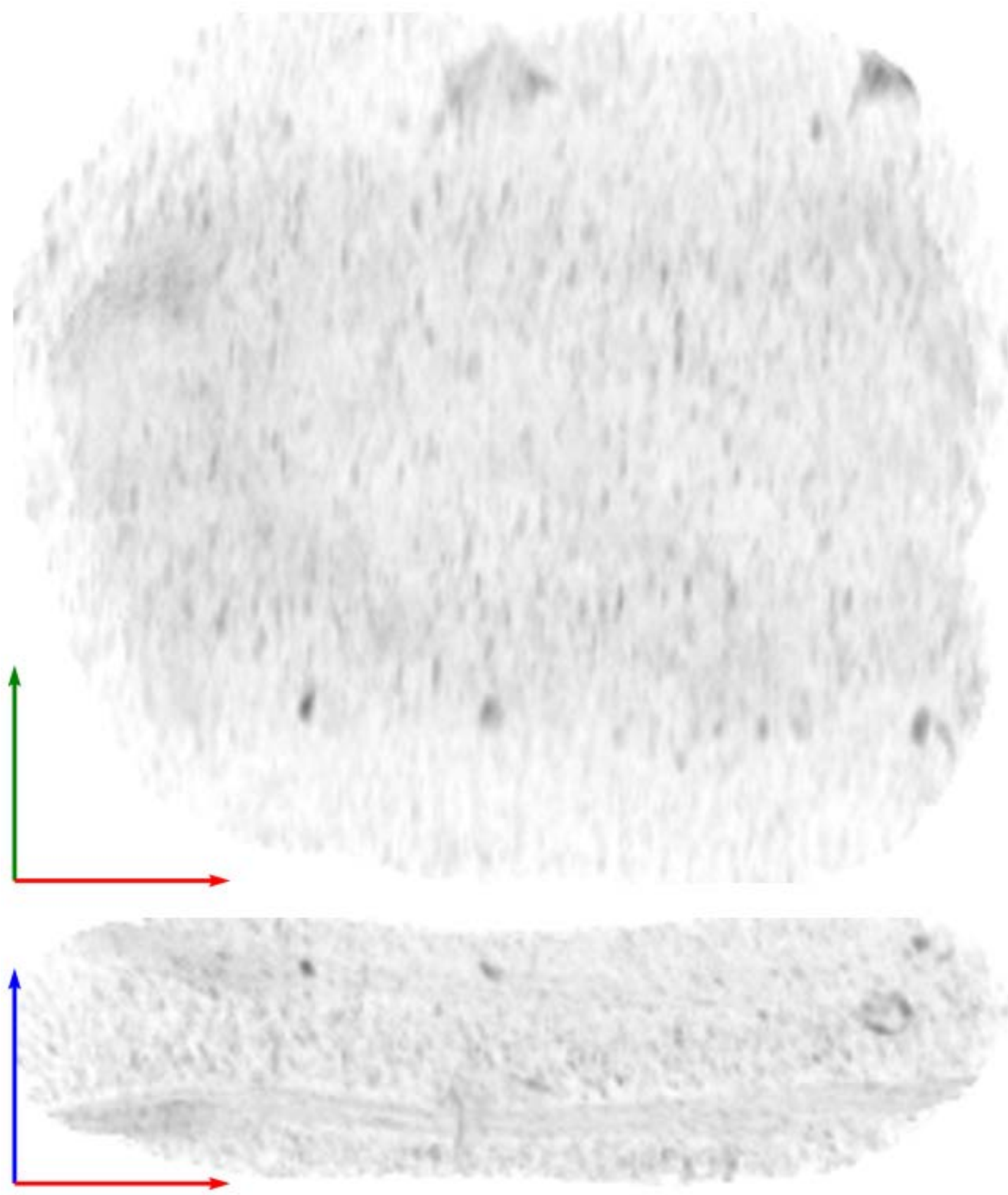

Tomogram ID: 20250227_AMmilled25-3_Position_69_3	SET: no labeling
Cleft ID: 2	Type: tissue

Denoised Tomogram Slice

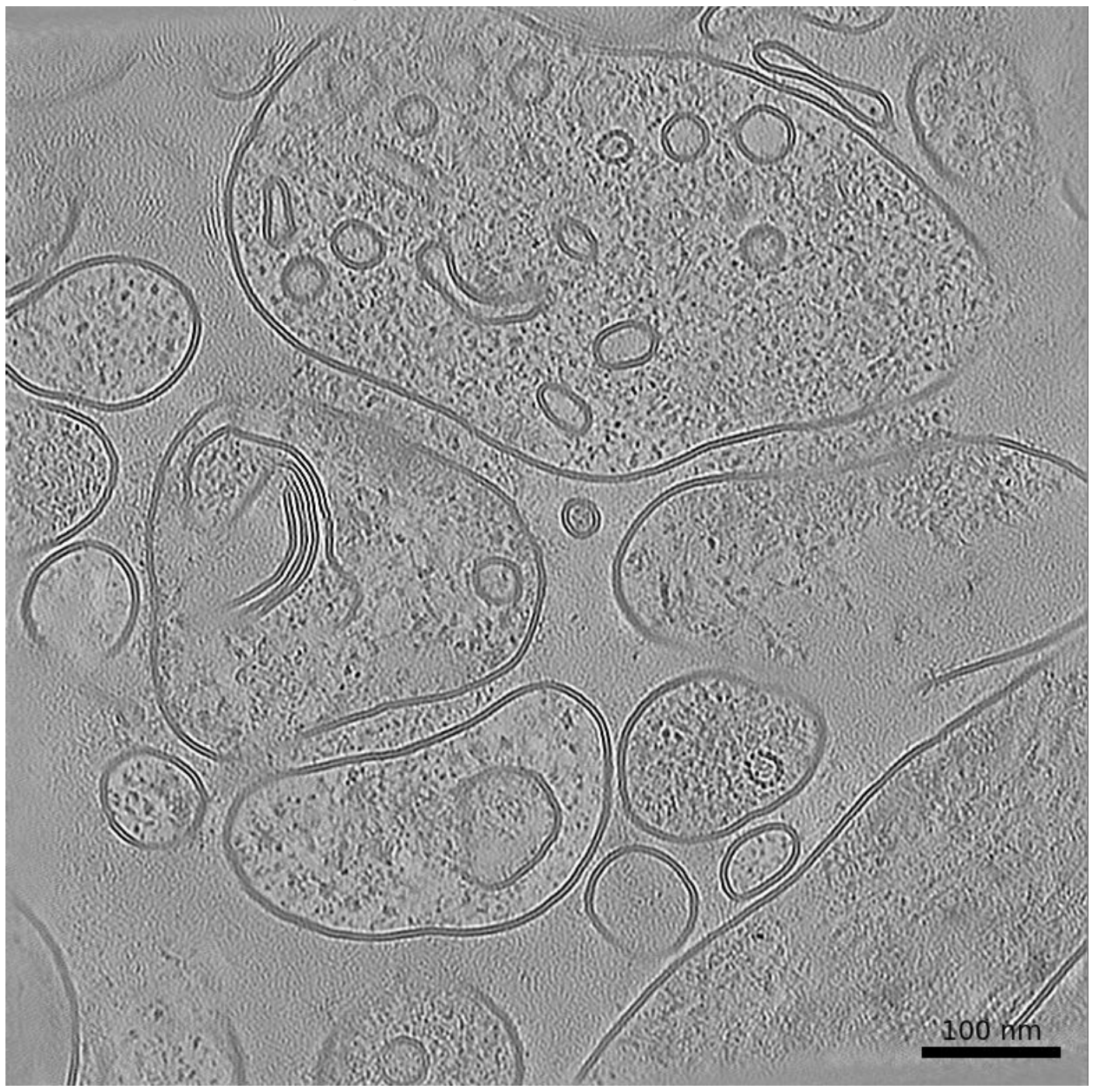

Membrane Segmentation & Cleft Position

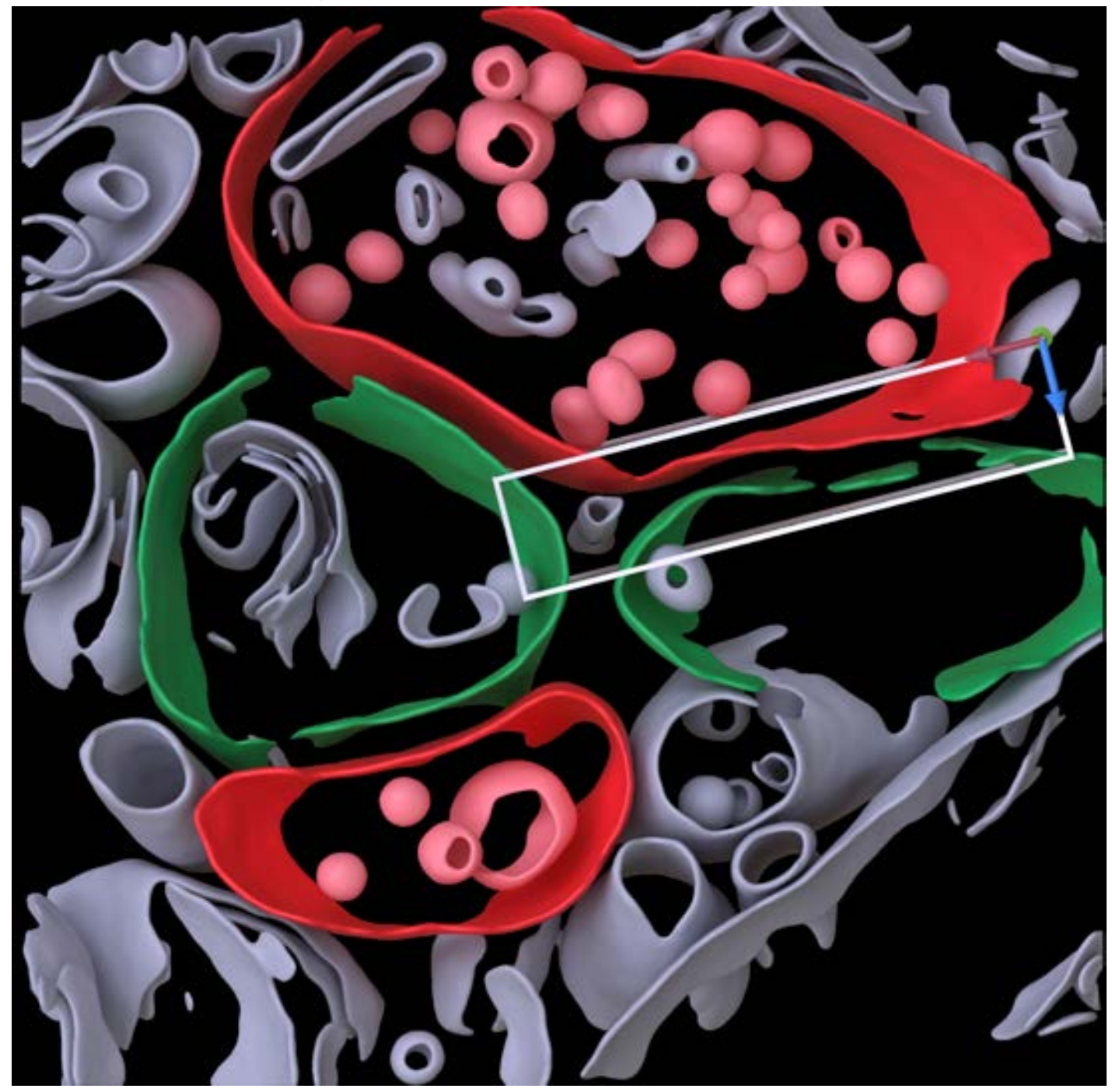

Cleft MIP

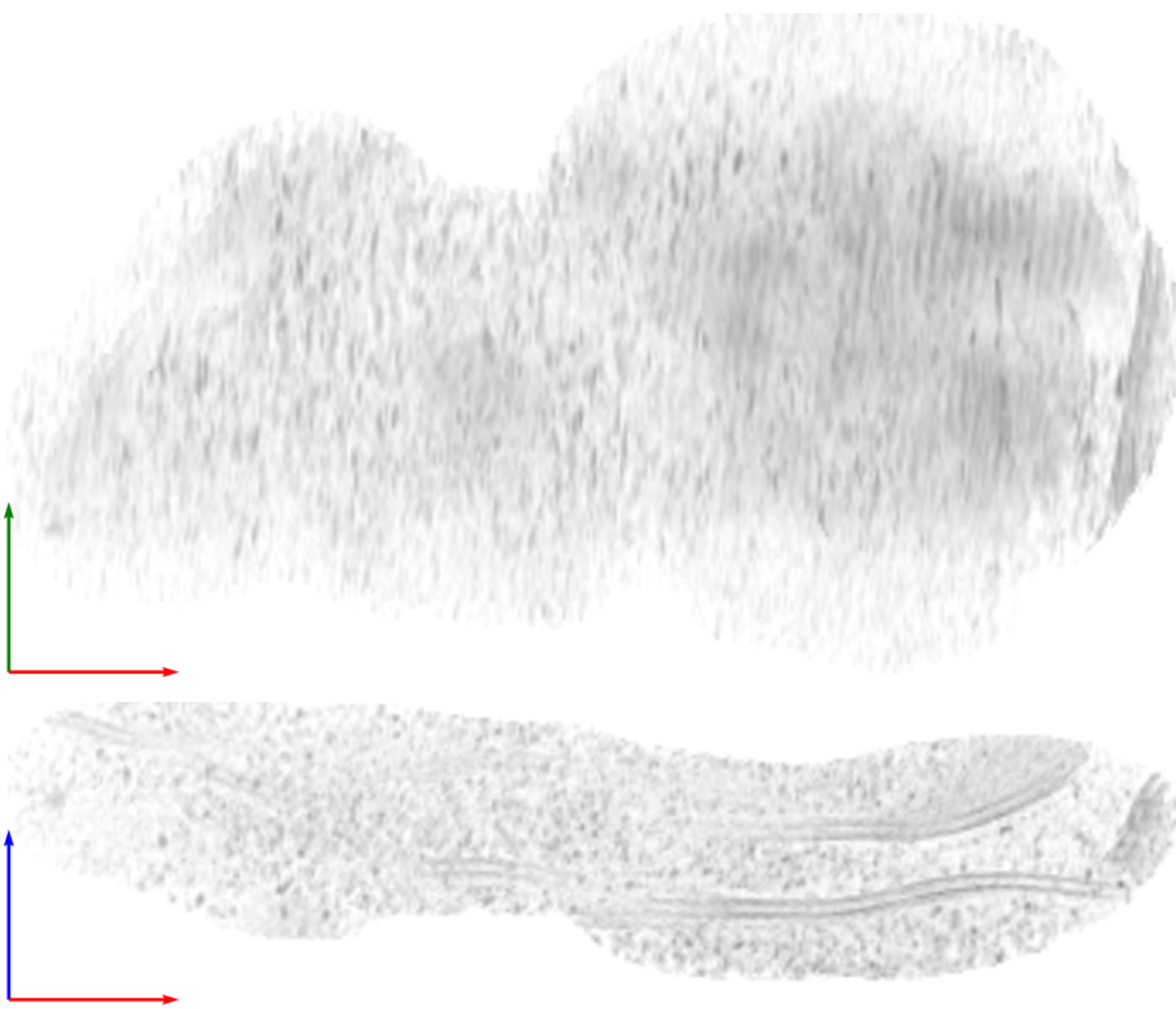

Tomogram ID: 20250227_AMmilled25-3_Position_79	SET: no labeling
Cleft ID: 0	Type: tissue

Denoised Tomogram Slice

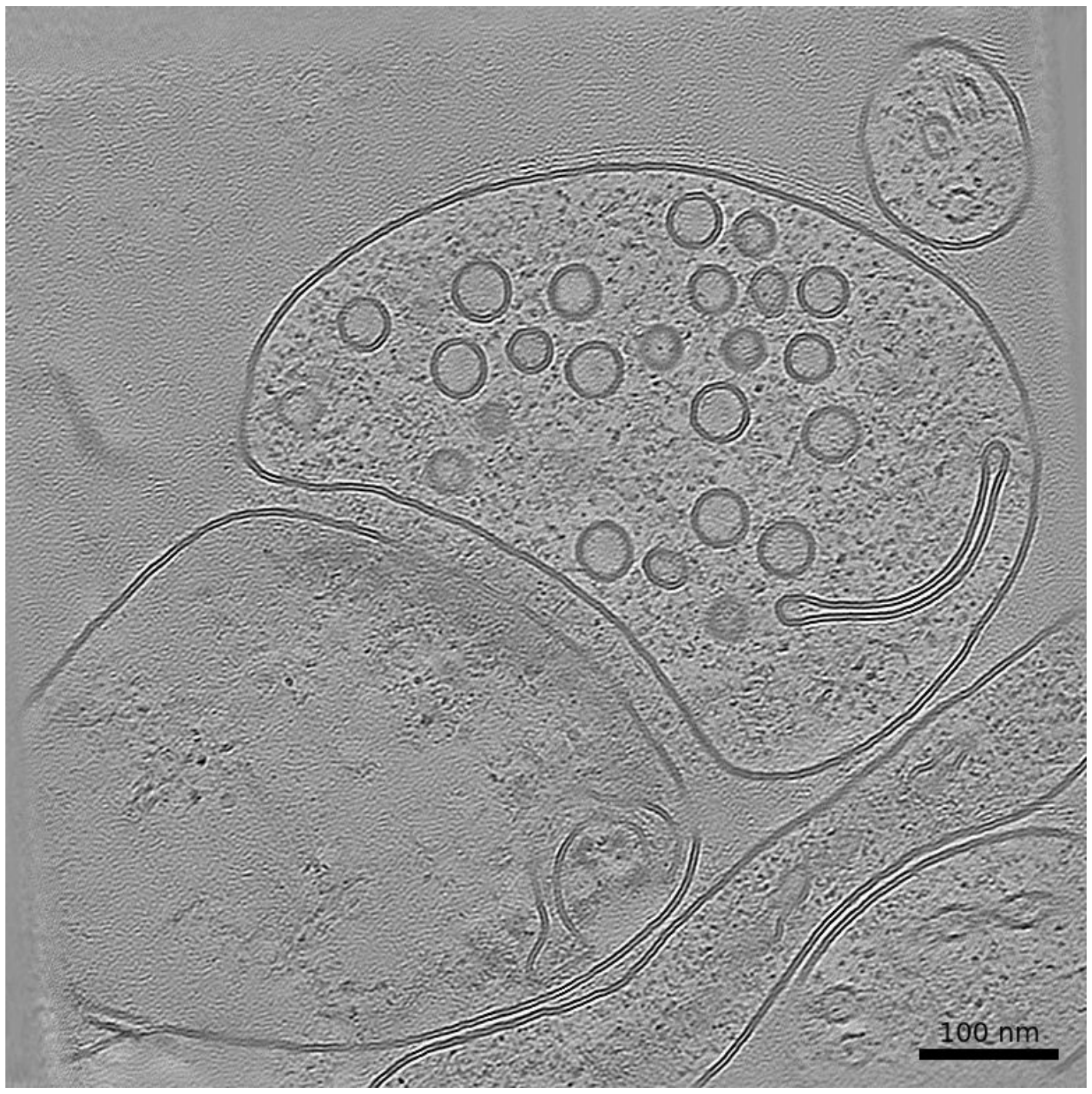

Membrane Segmentation & Cleft Position

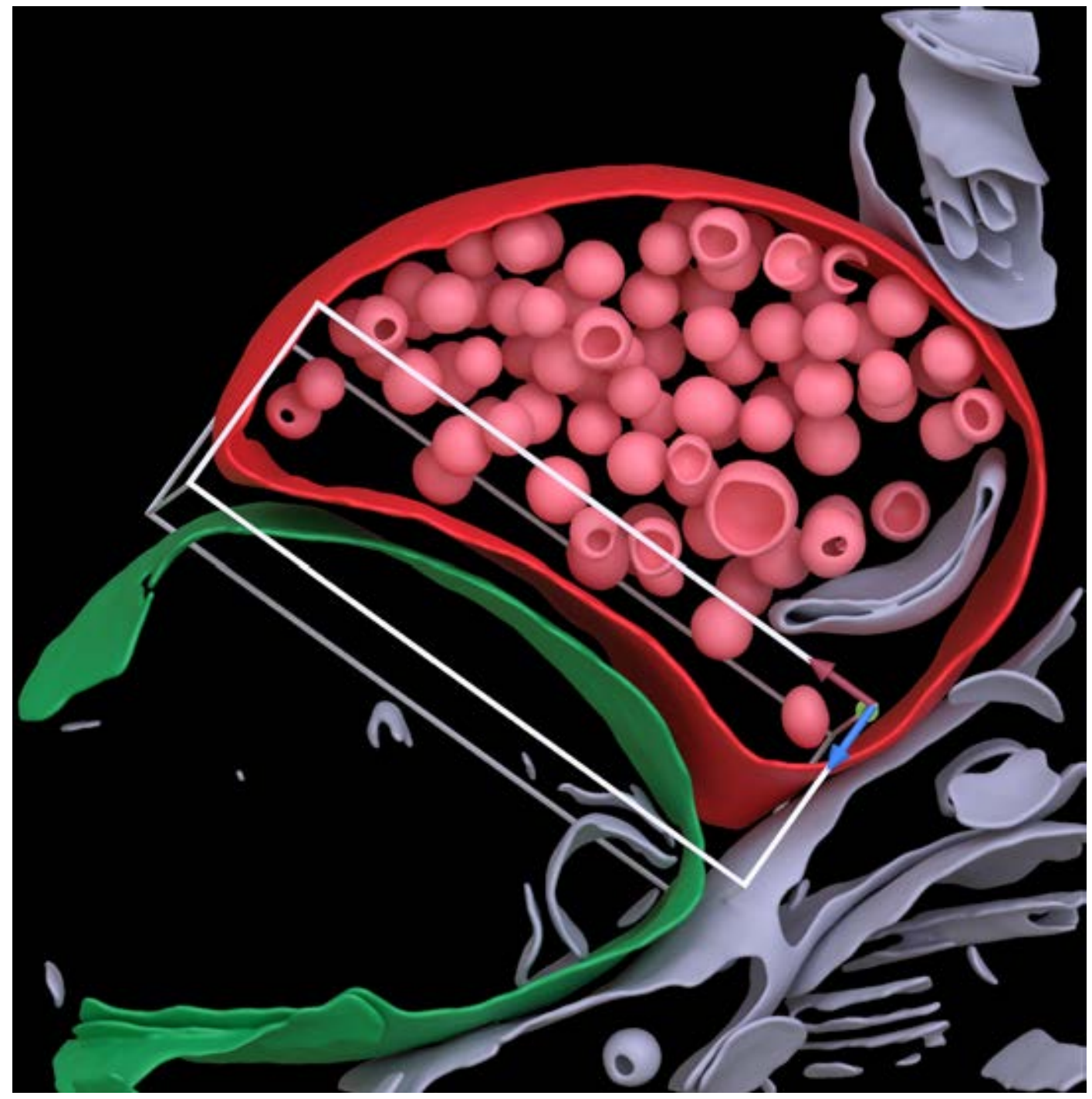

Cleft MIP

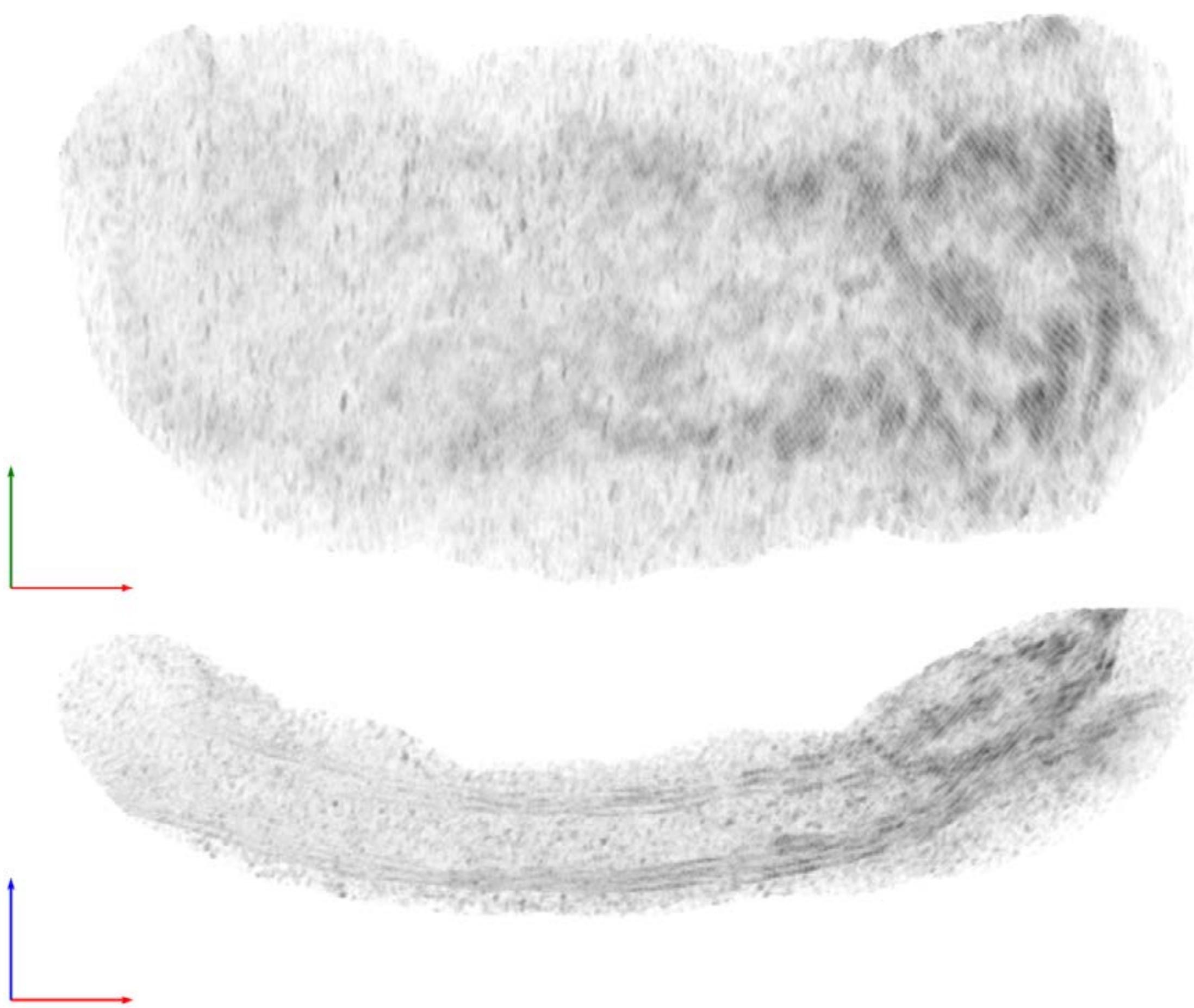

Tomogram ID: 20250228_AMmilled25-2_Position_8_2	SET: no labeling
Cleft ID: 0	Type: synaptosome

Denoised Tomogram Slice

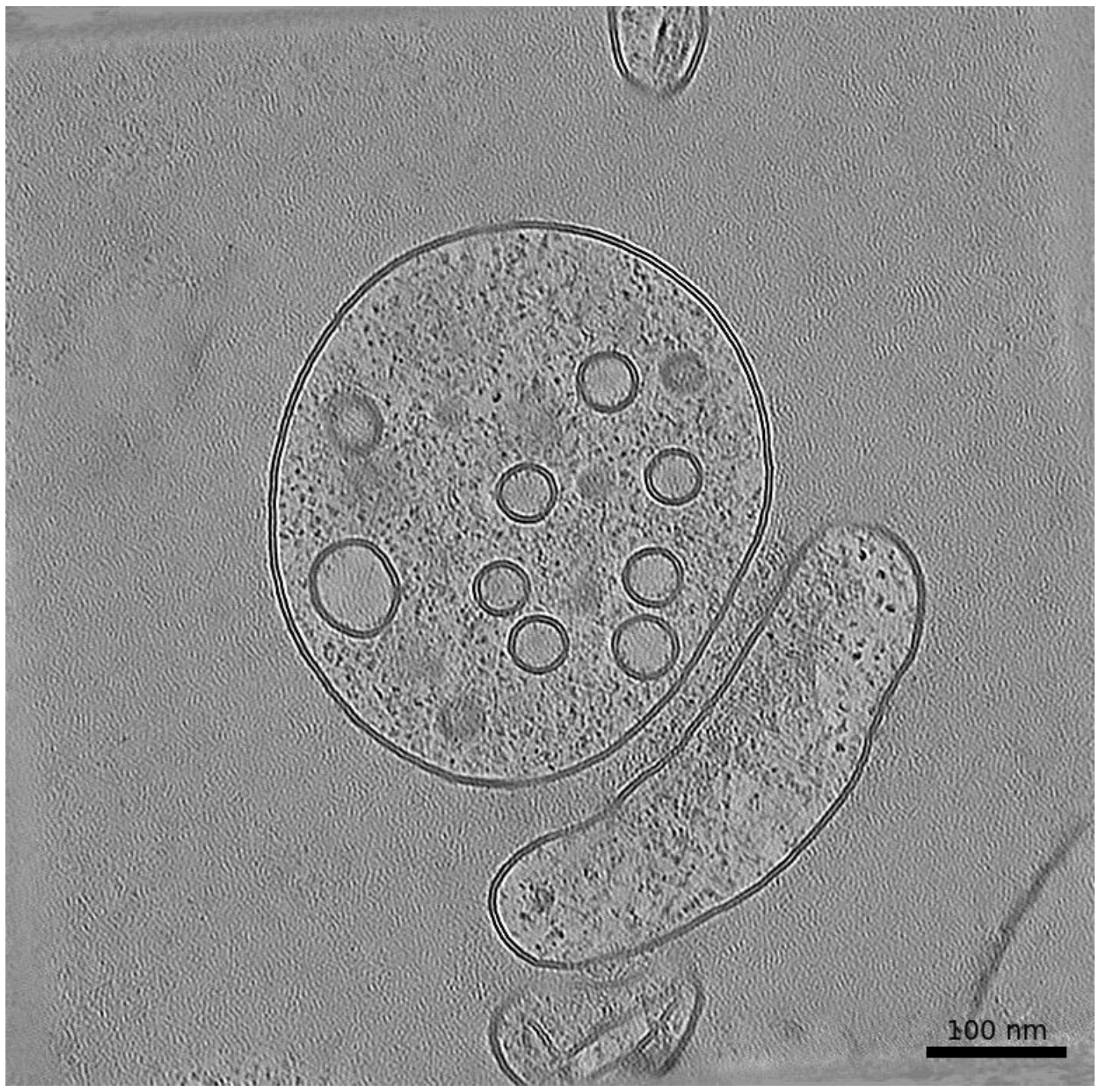

Membrane Segmentation & Cleft Position

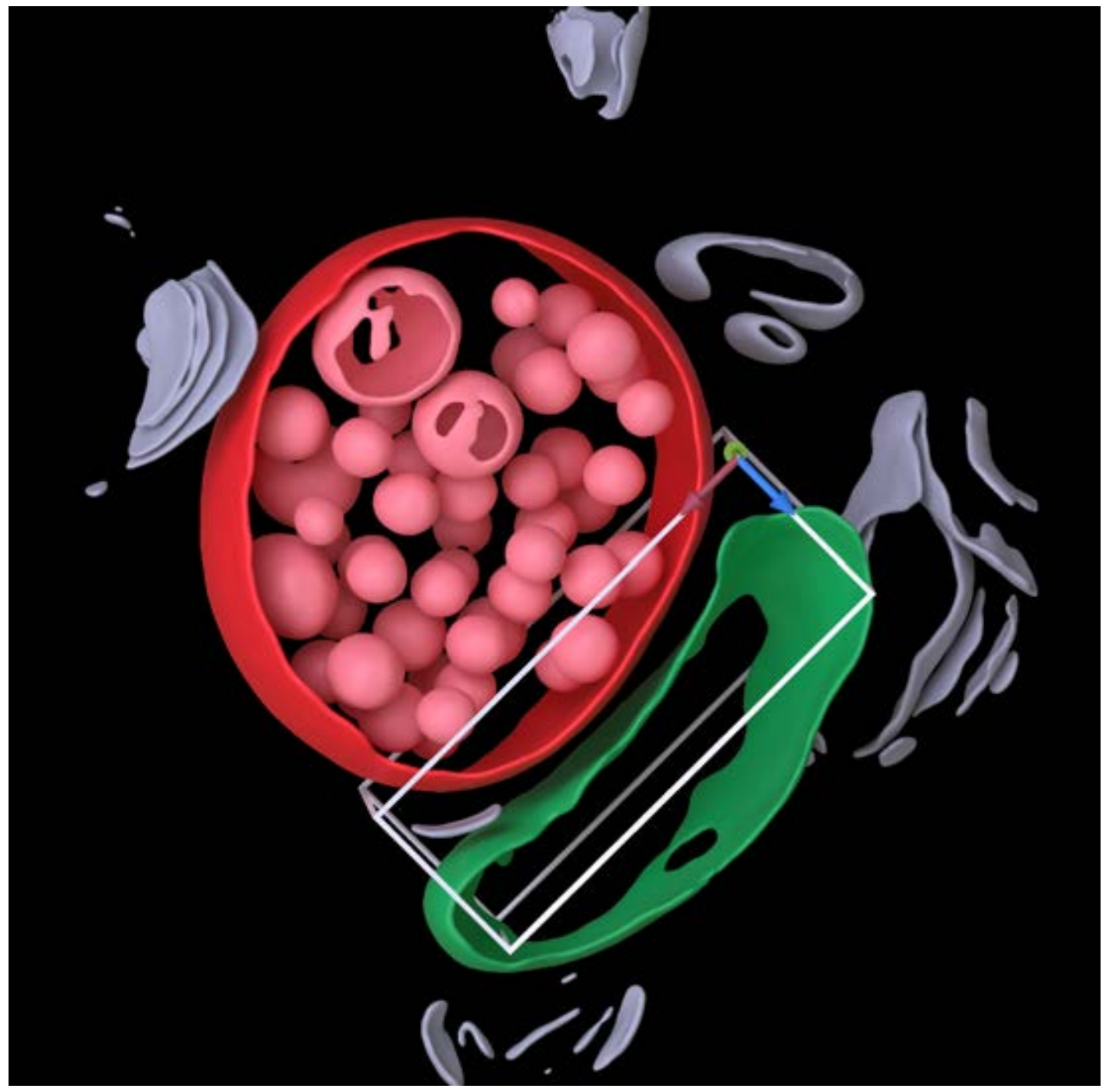

Cleft MIP

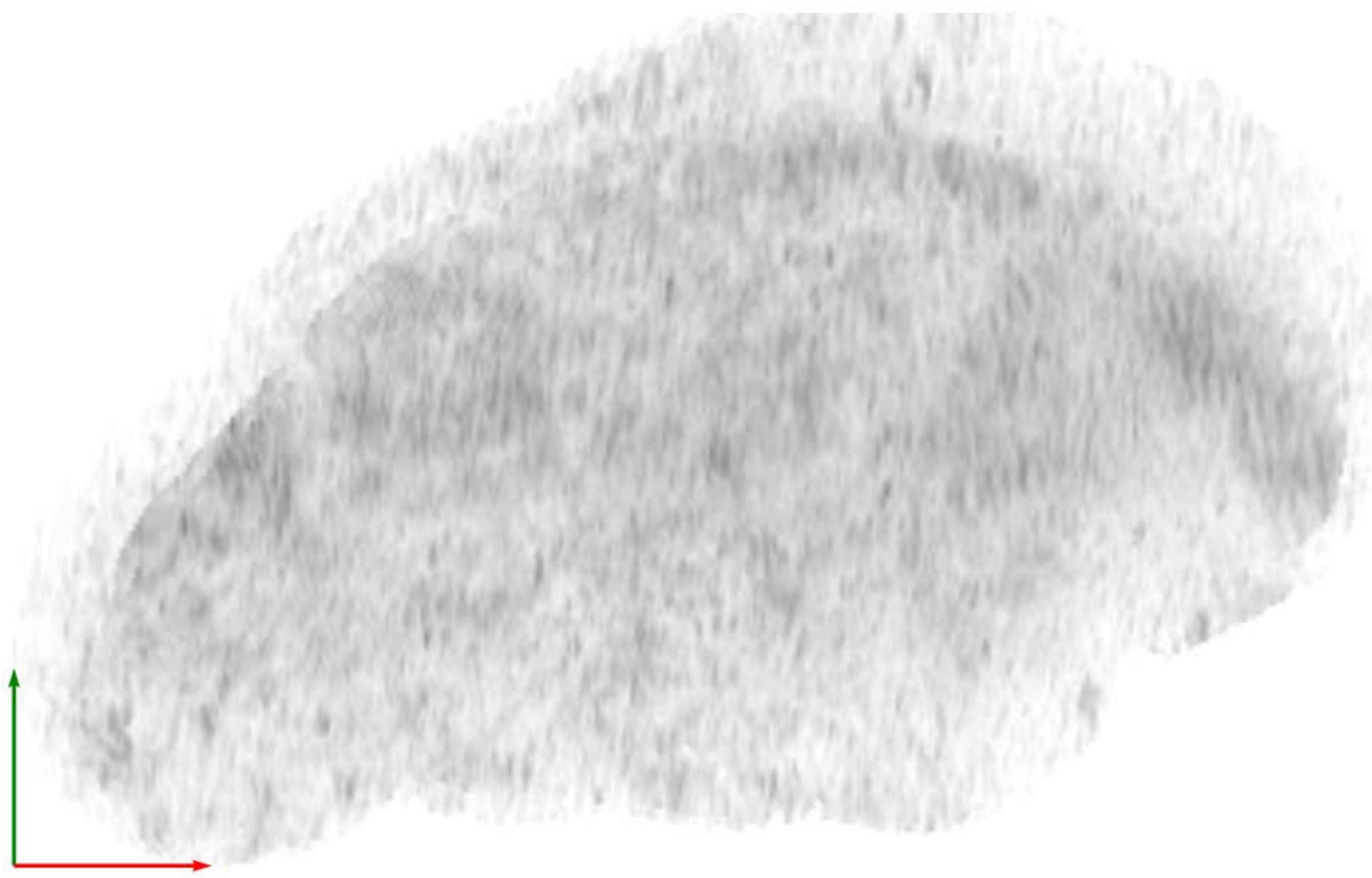

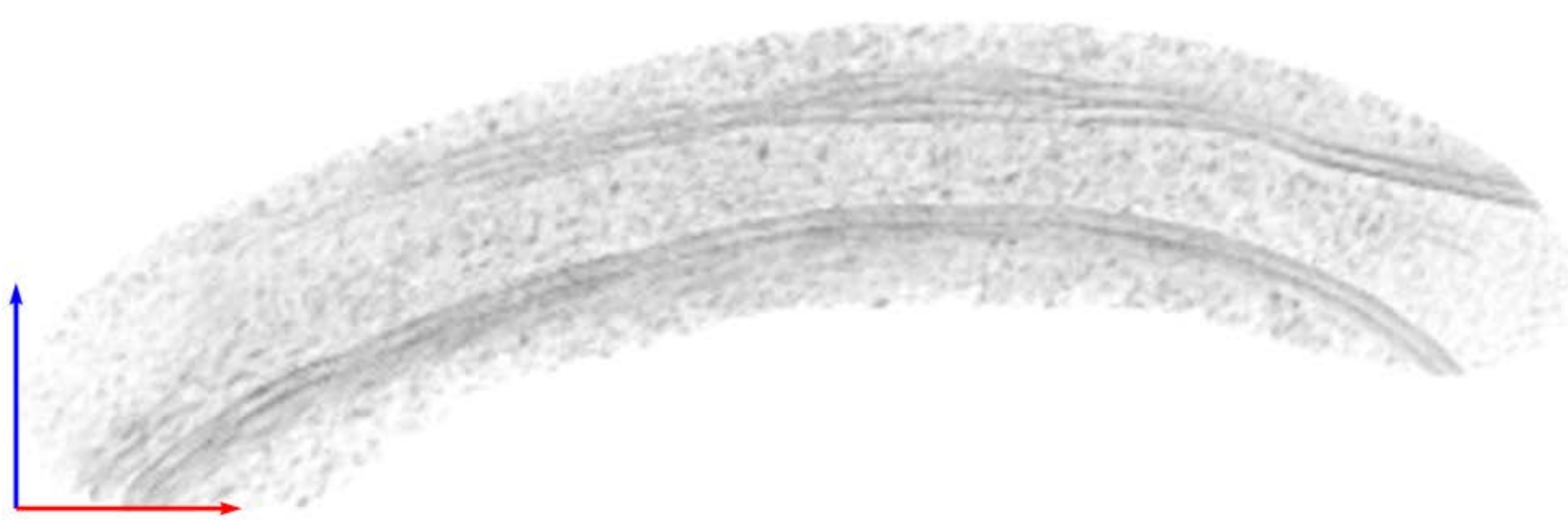

Supplement: Supplementary file 4 [file media-4.pdf]
